# Genome-Wide Identification and Characterization of Long Intergenic Non-Coding RNAs in *Ganoderma lucidum*

**DOI:** 10.1371/journal.pone.0099442

**Published:** 2014-06-16

**Authors:** Jianqin Li, Bin Wu, Jiang Xu, Chang Liu

**Affiliations:** Center of Bioinformatics, Institute of Medicinal Plant Development, Chinese Academy of Medical Sciences and Peking Union Medical College, Beijing, P. R. China; National Center for Biotechnology Information, United States of America

## Abstract

*Ganoderma lucidum* is a white-rot fungus best-known for its medicinal activities. We have previously sequenced its genome and annotated the protein coding genes. However, long non-coding RNAs in *G. lucidum* genome have not been analyzed. In this study, we have identified and characterized long intergenic non-coding RNAs (lincRNA) in *G. lucidum* systematically. We developed a computational pipeline, which was used to analyze RNA-Seq data derived from *G. lucidum* samples collected from three developmental stages. A total of 402 lincRNA candidates were identified, with an average length of 609 bp. Analysis of their adjacent protein-coding genes (apcGenes) revealed that 46 apcGenes belong to the pathways of triterpenoid biosynthesis and lignin degradation, or families of cytochrome P450, mating type B genes, and carbohydrate-active enzymes. To determine if lincRNAs and these apcGenes have any interactions, the corresponding pairs of lincRNAs and apcGenes were analyzed in detail. We developed a modified 3′ RACE method to analyze the transcriptional direction of a transcript. Among the 46 lincRNAs, 37 were found unidirectionally transcribed, and 9 were found bidirectionally transcribed. The expression profiles of 16 of these 37 lincRNAs were found to be highly correlated with those of the apcGenes across the three developmental stages. Among them, 11 are positively correlated (r>0.8) and 5 are negatively correlated (r<−0.8). The co-localization and co-expression of lincRNAs and those apcGenes playing important functions is consistent with the notion that lincRNAs might be important regulators for cellular processes. In summary, this represents the very first study to identify and characterize lincRNAs in the genomes of basidiomycetes. The results obtained here have laid the foundation for study of potential lincRNA-mediated expression regulation of genes in *G. lucidum*.

## Introduction

*Ganoderma lucidum* is a white-rot fungus belonging to family Ganodermataceae, order Polyporales, class Agaricomycetes and phylum Basidiomycota. It is best-known for its ability to produce numerous bioactive compounds, such as triterpenoids and polysaccharides [1], [2]. Annual sale of pharmacological products containing materials from *G. lucidum* is more than 3 billion US dollar. Due to its pharmacological and economic importance, elucidating the genetic basis for the production of its bioactive components is an active area for research. Previously, we have sequenced the complete genome of *G. lucidum* [3]. Through bioinformatic analysis of the genome sequence, we identified genes responsible for the production of secondary metabolites, mycelia mating, and wood degradation. For example, we have identified genes involved in the biosynthesis of triterpenoids. These include genes coding for enzymes belonging to the mevalonic acid (MVA) pathway and cytochrome P450 (CYP450) genes, many of the latter may be involved in the chemical conversion of ganoderic acids. In addition, we have identified genes involved in wood degradation, such as carbohydrate-active enzymes (CAZy) and ligninolytic oxidoreductases. While the majority of protein-coding genes have been identified, the non-coding genes of *G. lucidum* have not been studied in detail.

With the advancement in technologies such as RNA-Seq [4] and tilling arrays [5], it is discovered that a large proportion of the total transcripts of eukaryotes are non-coding RNAs (ncRNAs) [6]. ncRNAs can be classified into housekeeping ncRNAs or regulatory ncRNAs according to their functions. Housekeeping ncRNAs include rRNAs, tRNAs, snRNAs, and snoRNAs. Regulatory ncRNAs include small RNAs, such as microRNAs (miRNAs), small interfering RNAs, and long ncRNAs (lncRNAs). lncRNAs are usually defined as transcripts that are longer than 200 nucleotides, and do not carry an open reading frame longer than 100 amino acids [7]. lncRNAs are present in both lower forms of organisms, such as yeasts [8], and higher forms of organisms, including mice [9] and humans [10]. lncRNAs can be subdivided according to their positions in the genome into natural antisense transcripts, long intronic ncRNAs, and long intergenic ncRNAs (lincRNAs). lincRNAs are usually transcribed by RNA polymerase II, capped, polyadenylated and spliced [11].

Previous studies have suggested that lncRNAs play critical regulatory roles in cellular processes in eukaryotes. In fungi, lncRNAs regulate the synthesis of serine [12], [13], galactose [14], [15], and nucleic acid [16]. In animals, lncRNAs are involved in development [17]–[19] and disease response [10], [20]. In plants, lncRNAs have been systematically screened from *Arabidopsis thaliana*, *Triticum aestivum*, *Digitalis purpurea* and *Medicago truncatura* [7], [21]–[28]. These lncRNAs are important in phosphate-starvation response, gender-specific expression, nodulation, and cold-stress response. For instance, the expression of the *TPSI 1/Mt4* gene, an lncRNA, is induced under phosphate stress [29]–[34]. Three lncRNAs, namely *Zm401*, *CCLS96.1*, and *CR20*, are gender specific in maize, campion, and cucumber, respectively [35]–[37]. The *Enod40* gene is involved in nodulation [38]. In *Arabidopsis*, *COOLAIR* and *COLDAIR*, which are required for vernalization, silence the *FLC* by epigenetic suppression [39], [40]. Two subsets of lincRNAs in the human genome, named enhancer RNAs (eRNAs) [41], [42] and enhancer-like RNAs (or activating RNAs) [43], have been the focus of recent studies. Most eRNAs are bidirectional, relatively short (<2 kb long), and predominantly nonpolyadenylated transcripts [41], [42]. The expression of the eRNAs is correlated with that of their nearest protein-coding genes [44], [45]. In contrast, the majority of enhancer-like RNAs are unidirectional and polyadenylated. It is reported that they function to enhance gene expression [46].

Although a large numbers of lncRNAs had been discovered, only a few have been studied in mechanistic details [47]. For example, lncRNAs can regulate gene expression by recruiting epigenetic complexes at a molecular level [48], [49]. The regulated gene expression directly affects the process of transcription [50], [51], and also functions at various steps of the mRNA processing and stability control [52]. lncRNAs may function in *cis* to regulate the expression of genes on a neighboring loci; or they might act in *trans*, which regulates the genes located in other distant domains or chromosomes [47]. Previous studies showed that the location of lncRNAs in genome is not random, and lncRNAs tend to act in *cis* with neighboring protein-coding genes. For example, in mouse testis, many lncRNAs either overlap with or are adjacent to the key transcription factors and other genes involved in spermatogenesis [19]. Brain-expressed lncRNAs are preferentially located adjacent to the brain-expressed protein-coding genes involved in the transcriptional regulation or in nervous system development [18].

In this study, we carried out a genome-wide identification of lincRNA genes in *G. lucidum* using data generated from RNA-Seq technologies. A total of 402 lincRNAs were identified. Due to the technical difficulties in gene knock-in and knock-out in *G. lucidum*, we used co-localization and co-expression regulation across the three different developmental stages as criteria to identify functionally related genes. A modified 3′ RACE method (MRA) and real-time quantitative PCR (qPCR) were then used to determine the transcript orientation and expression abundance for a selected set of lincRNAs. This study, for the first time, described the presence of lincRNAs in basidiomycete genome. This study also provides information with regard to the potential lincRNA-mediated regulation of genes involved in triterpenoid production, mycelia mating, and wood degradation in *G. lucidum*.

## Materials and Methods

### Materials and data availability

*G. lucidum* dikaryotic strain CGMCC5.0026 was cultured as described in our previous paper [3]. The complete genome sequence of *G. lucidum* has been deposited at GenBank with the accession number PRJNA71455. The Illumina RNA-Seq reads have been deposited in the short-read archive at GenBank with the accession number of SRA048015 as described previously [3].

### Identification of lincRNA and analysis of apcGene

**Figure 1:**
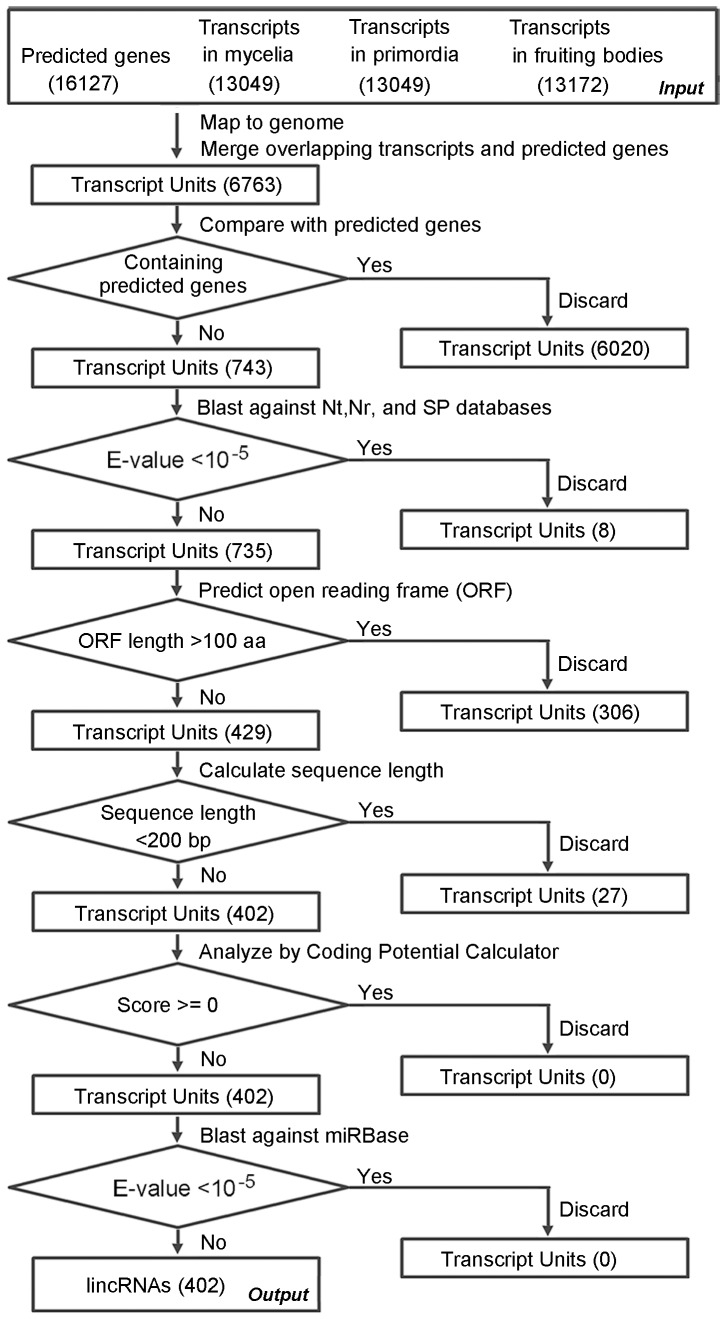
The bioinformatic pipeline used to identify the lincRNA candidates. The predicted genes, transcripts and transcript units (TUs) before and after each filtering step are shown in rectangle. The number of TUs is shown in parenthesis. The filtering process is shown next to the arrowed line above each diamond. The criterion used in each filtering step is shown in diamond.

We developed a computational pipeline for the identification of lincRNAs from RNA-Seq data. The details of the pipeline are shown in Figure 1. Briefly, the reads from the three developmental stages were assembled separately using the cufflinks with default parameters and the whole genome sequences as reference [53]. The resulting transcripts and predicted genes were mapped to the genome sequences. The overlapping transcripts and genes were merged into a transcript unit (TU). TUs that do not overlap with predicted genes were compared with nucleotide (Nt), non-redundant protein (Nr), and Swiss-Prot (SP) databases using a cutoff E-value of <10^−5^. The remaining candidates were subjected to ORF prediction by ESTScan [54]. Any TU that encode ORFs longer than 100 aa [55] or are shorter than 200 bp were discarded [56]. The remaining TUs were compared to sequences in the miRBase (Released 19, http://www.mirbase.org) [57] by BLASTN using a cutoff E-value of <10^−5^. The protein-coding potential of TUs was analyzed by Coding Potential Calculator (CPC) software. Any TU was deemed to be non-coding if the coding potentials of both strands scored less than zero [58].

In this paper, the nearest protein-coding gene located on the 5′ upstream or 3′ downstream of the lincRNA was named as adjacent protein-coding gene (apcGene). Based on this definition, one lincRNA should have two apcGenes that locate on the 5′ and 3′ respectively, a 1∶2 relationship. On the other hand, two lincRNAs might correspond to the same apcGene as the two lincRNAs locate on the 5′ and 3′ of the apcGene respectively, a 2∶1 relationship. In a special case, several lincRNAs are arranged consecutively and share the same apcGene, a n:1 relationship. A total of 697 apcGenes were analyzed, and categorized using the Gene Ontology (GO) [59] and Kyoto Encyclopedia of Genes and Genomes (KEGG) database using a cutoff E-value of <10^−5^ [60]. apcGenes were also compared with the sequences in the Functional Transcription Factor Database (FFTD) to predict transcription factors using default parameters [61].

### Determination of the transcriptional orientation of lincRNAs

**Figure 2:**
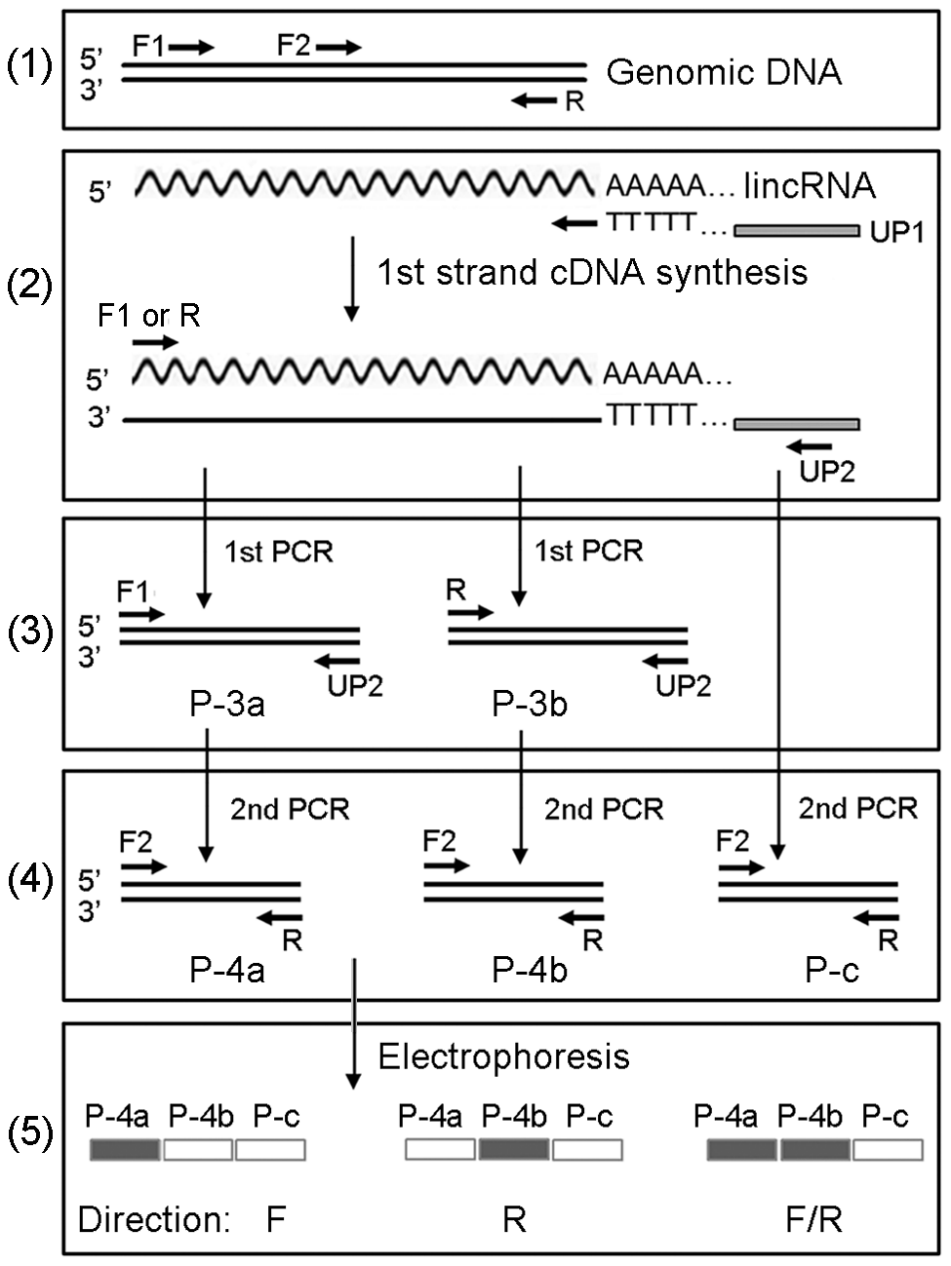
Schematic representation of the MRA method. (1) Relative locations of primers F1, F2, and R on a target locus; (2) First-strand cDNA synthesis using primer UP1; (3) First-round PCR amplification. The gene-specific primer F1 or R was used as the forward primer and primer UP2 was used as the reverse primer, producing PCR products P-3a and P-3b, respectively. The relative positions of the primers on the PCR products are shown; (4) Second-round PCR amplification. The gene-specific F2 was used as the forward primer and R was used as the reverse primer, producing PCR products P-4a and P-4b, respectively. The relative positions of the primers on the PCR products are shown. For the control, the first-strand cDNAs were used as the template, producing products P-c; (5) A graphic representation of the expected electrophoresis results and the corresponding transcriptional orientation they represent. The filled rectangle indicates the presence of a band and the hollow rectangle indicates the absence of a band. The name of the PCR products are the same as described above in (3) and (4). The transcriptional direction determined based on each pattern is shown as F (Forward), R (Reverse) and F/R (bidirectional).

We developed a method, named MRA, to determine the transcriptional orientation of lincRNAs. The details of the method are described in Figure 2. Briefly, total RNAs were extracted from mycelia, primordia, and fruiting bodies using an RNeasy plant mini kit (QIAGEN, USA). Genomic DNA contaminant was removed using an RNase-free DNase I (Promega). Pooled and un-pooled RNA samples were reversely transcribed to produce cDNAs using a GeneRacer kit (Invitrogen) according to the recommended protocol. The first round of PCR amplification was performed under the following conditions: 95°C for 3 min; 35 cycles of 95°C for 15 s; 60°C for 15 s; and 72°C for 3 min. The second round of PCR amplification was performed under the following conditions: 95°C for 3 min; 25 cycles of 95°C for 15 s; 60°C for 15 s; and 72°C for 30 s. The primers are listed in Table S1. The PCR products were separated by electrophoresis with 2% agarose gel.

### qPCR and data processing

Total RNAs were extracted and processed as described above. Reverse transcription, qPCR, and data analysis were performed as described by Wu et al. [27]. Gene-specific primers are listed in Table S1. The expression data for the genes were normalized against that of the glyceraldehyde-3-phosphate dehydrogenase (GAPDH) gene. For those stages in which the lincRNAs or apcGenes were not detected, the corresponding threshold cycle numbers were set as 40. In correlation analysis, we normalized the expression levels in each stage to the maximum expression level of the three developmental stages to compare the expression profiles among these stages. The Pearson correlation coefficient of the expression profiles was then calculated.

## Results

### Computational identification of *G. lucidum* lincRNAs

We developed a bioinformatic pipeline to identify lincRNAs based on RNA-Seq data generated in our previous study [3]. The pipeline, shown in Figure 1, is similar to those used to identify lincRNAs from *A. thaliana*, *T. aestivum*, *D. purpurea*, *M. truncatura*, and *Drosophila melanogaster* [27], [62]–[67]. The transcripts identified from the three developmental stages and predicted genes were mapped to the genome sequences. Any overlapping genes and transcripts were treated as a TU. A total of 6763 TUs were identified. And 6020 of them were subsequently discarded because they contain predicated genes. The remaining 743 transcripts were compared with Nt, Nr, and SP databases by BLAST with a cutoff E-value of <10^−5^. Eight TUs showed significant hits. The remaining 735 TUs were subjected to ORF prediction, in which 306 TUs encoded putative peptides longer than 100 aa. Among the remaining 429 TUs, 27 were discarded because they were <200 bp long. The remaining 402 TUs were then subjected to analysis with CPC software, which assess the protein-coding potential of a transcript based on six biologically meaningful sequences features, and none of the TUs were found to have protein-coding potential. Finally, the 402 TUs were compared with the sequences in the miRBase, and no TU was found significantly similar to any sequences in the database. Since very few lincRNAs in fungi have been described before, the failure to identify homologous lincRNAs might simply reflect the fact that very few fungal lincRNAs are in miRBase [68]. Because lincRNAs have been found to be precursors of miRNAs [22], failure in detecting any lincRNA genes as host genes in *G. lucidum* can not excluded the possibility that some lincRNAs are indeed precursors of miRNAs. Additional experiments are needed to identify miRNAs from *G. lucidum*, which will then be compared to lincRNAs to determine if any lincRNAs serve as the precursors of these miRNAs. The 402 TUs were considered as lincRNAs and were characterized further.

### Length distribution of lincRNAs and functional classification of their apcGenes

The sequences of the 402 putative lincRNAs are shown in Text S1. Detailed information including chromosomal locations, expression levels, length of the lincRNAs, and apcGenes are shown in Table S2. Analysis of the size distribution showed that many lincRNAs ranged from 200 bp to 1966 bp in length, with an average length of 609 bp (Figure S1). This range is significantly longer than that of *Arabidopsis* (200 bp to 300 bp) [7] and *D. melanogaster* (approximately 443 bp) [62]. About 11.7% of the lincRNAs were longer than 1000 bp in *G. lucidum*. This kind of longer lincRNAs has actually been identified in mammalians, which are conserved and associated with chromatin-modifying complexes that regulate gene expression [66].

Previous study showed that lncRNAs may function on linked genes in the neighboring gene loci in *cis* [18]. We then named the nearest neighbor protein-coding genes located on the 5′ upstream or 3′ downstream of the lincRNA as apcGenes. A total of 697 apcGenes were identified and categorized using GO, which described gene products with regard to their associated biological processes, cellular locations, and molecular functions in a species-independent manner [59]. One or more GO terms were assigned to each of the 212 apcGenes. The GO assignments of the 26 subcategories under the three categories are shown in Figure S2A. “Metabolic process”, “protein binding”, “cellular process” and “catalytic activity” were four most assigned subcategories. We also categorized all of the apcGenes using KEGG (Figure S2B). About 176 of 697 apcGenes were mapped to KEGG pathway. More genes were found to belong to pathways of “Metabolism” and “Genetic Information Processing” in the first layer of the KEGG pathway terms. For the second layer, “Replication and Repair,” “Folding, Sorting, and Degradation,” “Translation,”, “Transcription,” and “Cell Growth and Death” were the top 5 most assigned terms. The protein sequences of these 697 apcGenes were also compared with those in FFTD using default parameters [61]. A total of 228 apcGenes were annotated as transcription factor based on the annotation of the best hit from FFTD. These genes can be further divided into 27 families of transcriptional factors (Table S3). In summary, it is evident that many apcGenes might be involved in the metabolism and gene expression regulation.

### Identification of 46 lincRNAs of interests and determination of their strand specificity

**Figure 3:**
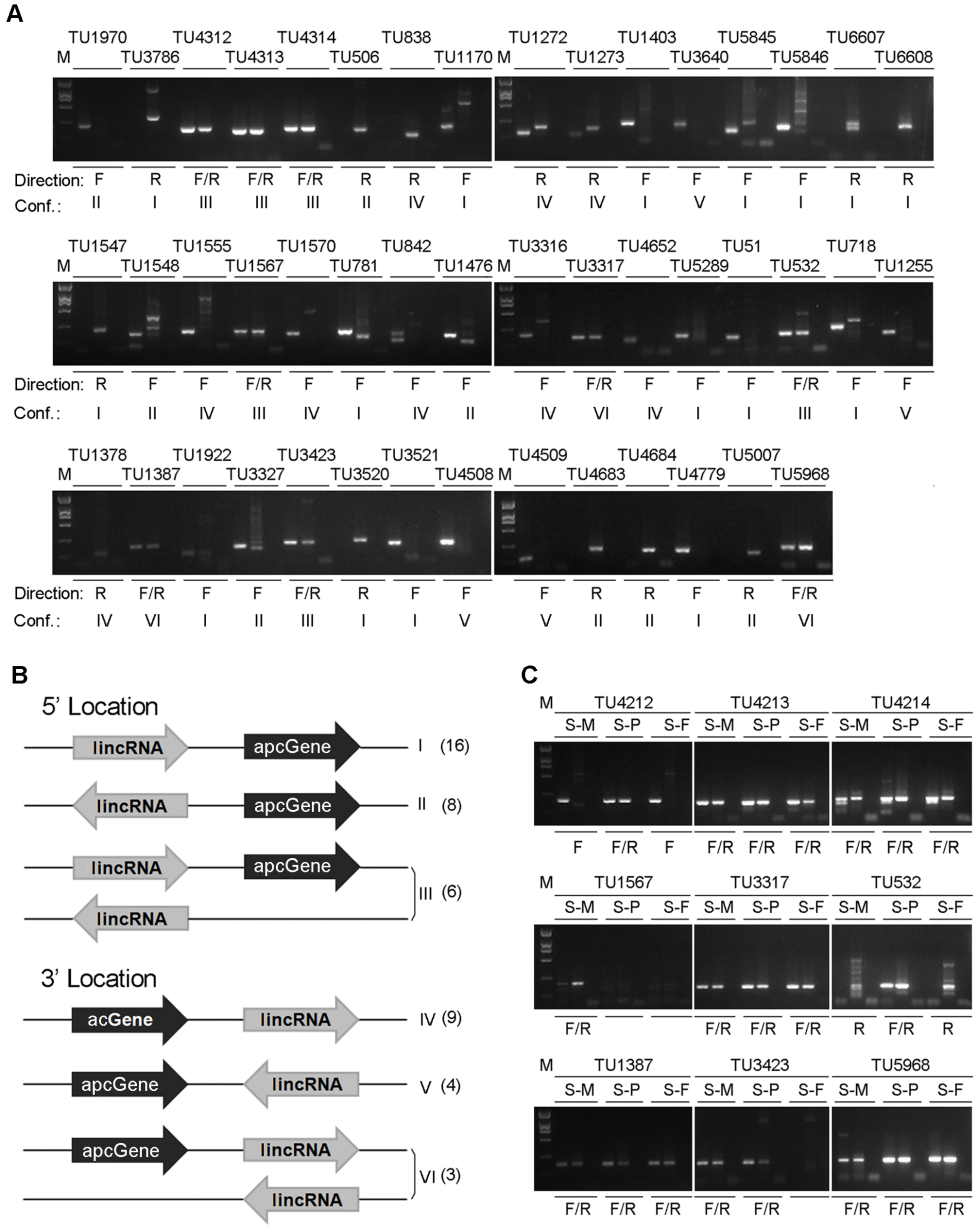
Determination of transcript orientation for 46 lincRNAs by MRA. (A) Electrophoresis analysis of the products amplified from pooled RNA samples. The products from the second-round PCR were separated by electrophoresis with 2% agarose gels in the order of P-4a, P-4b, and P-c as described in Figure 2-(5). The IDs of lincRNAs are listed above the gel picture, and the transcriptional direction and lincRNA/apcGene configuration are listed below the gel picture. The explanation of each configuration is shown in panel (B) M: DNA marker DL2000; F: forward; R: reverse; F/R: bidirectional; Conf: lincRNA/apcGene configuration; (B) Schematic representation of the various types of lincRNA/apcGene configurations. The lincRNA is shown as the gray arrow, whereas the apcGene is shown as the black arrow. The IDs for the configurations are represented by roman numerals. The number of lincRNAs belonging to each configuration is shown in parenthesis; (C) Gel electrophoresis of the nine bidirectional lincRNAs using RNA samples isolated from three developmental stages separately. S-M: RNA sample from mycelia; S-P: RNA samples from primordia; S-F: RNA sample from fruiting bodies.

As we are most interested in genes belonging to either pathways of triterpenoid biosynthesis and lignin degradation, or families of CYP450, mating type B (*mat*B) and CAZy, we first identified apcGenes falling into these categories (Table S4). We then identified lincRNAs that are adjacent to, or co-localized with these apcGenes for detailed characterization, hypothesizing that they are potential expression regulators of their apcGenes. Forty-six lincRNAs were identified in this way and subjected to MRA analysis. It is shown that 37 lincRNAs were transcribed in one direction only, as indicated by “F” or “R” in Figure 3A. The other 9 lincRNAs were transcribed in both directions, as indicated by “F/R” in Figure 3A. For four lincRNAs, such as *TU1272*, *TU718*, *TU1476*, and *TU781*, multiple bands were observed, suggesting non-specific PCR amplification. In this case, the strand specificity was determined based on the band having a size similar to that predicted from the lincRNA.

The 46 lincRNAs were classified into six classes (Figure 3B) based on their orientation and location relative to their apcGenes. The numbers of lincRNAs belonging to particular classes are shown in parentheses after the class number. Among the 46 lincRNAs, 30 lincRNAs located on the 5′ end of the apcGenes. Among these 30 lincRNAs, 16 and 8 lincRNAs were transcribed in the same (Figure 3B-I) or opposite (Figure 3B-II) directions as those of their apcGenes respectively. And 6 lincRNAs were transcribed in both directions (Figure 3B-III). The remaining 16 lincRNAs located on the 3′ end of the apcGenes. Among them, 9 and 4 lincRNAs were transcribed in the same (Figure 3B-IV) or opposite (Figure 3B-V) directions as those of their apcGenes respectively. And 3 lincRNAs were transcribed in both directions (Figure 3B-VI).

In the above experiments, RNA samples obtained from three developmental stages were pooled together before they were subjected to the MRA analyses. To determine if the 9 bidirectionally transcribed lincRNAs are indeed bidirectionally transcribed in each of the stages, the RNA samples from the three developmental stages were subjected to MRA analyses separately without pooling. The results showed that five lincRNAs were transcribed bidirectionally in all three stages, whereas four lincRNAs (*TU4212*, *TU1567*, *TU532* and *TU3423*) were transcribed bidirectionally in one or two stages only (Figure 3C). The differential transcription of these lincRNAs at different developmental stages might reflect their stage-specific functions.

### Comparison of the expression profiles of 37 unidirectional lincRNAs and their apcGenes

**Figure 4:**
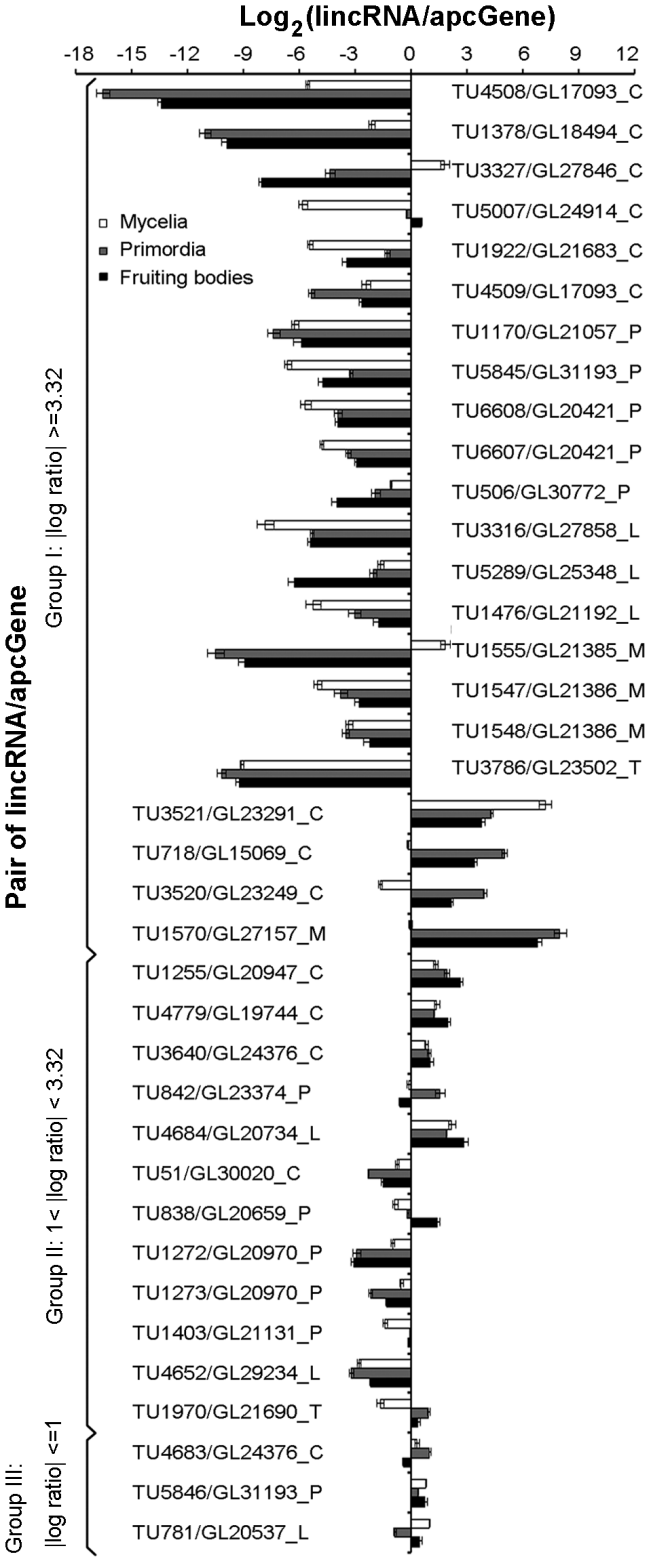
Relative expression levels of lincRNAs and their apcGenes across the three developmental stages. The X axis shows the log ratio abundance for lincRNA/apcGene determined by qPCR. The Y axis lists the pair of lincRNA and apcGene. The lists are sorted based on the log ratio abundance and divided into three groups (I, II and III). The IDs of the lincRNAs and their apcGenes are shown to the right or left of the graph. The postfixes indicate the families or pathways to which the apcGenes belong. C: CAZy protein family; P: CYP450 protein family; L: lignin degradation pathway; M: *mat*B genes; T: triterpenoid synthesis pathway. Error bars denote the standard deviations of three qPCR replicates.

We then used qPCR to quantify and compare the expression levels of lincRNAs and their corresponding apcGenes. Due to the potential complexity of interactions between bidiretional lincRNAs and their apcGenes, we only studied the 37 unidirectional lincRNAs and their apcGenes (Table S5). The log ratios for the expression levels of lincRNAs to apcGenes were shown in Figure 4. The expression of three lincRNAs (*TU1378*, *TU1555*, and *TU4508*) and an apcGene (*GL27157*) were detected only in the mycelia stage, whereas the expression of *TU3327* was detected in mycelia and primordia stages. The expressions of the remaining lincRNAs and apcGenes were detected in all of the three stages.

We classified the pairs of lincRNA and apcGene into three groups based on log ratio of their expression levels. For group I, the absolute value of log ratio for lincRNA/apcGene is > = 3.32 in at least one developmental stage, which is equivalent to a 10-fold difference. For group II, the absolute value of log ratio is between 1 and 3.32 in at least one developmental stage, which is equivalent to a 2 to 10-fold difference. For group III, the absolute values of log ratios are < = 1 across all three developmental stages, which is equivalent to a less than two-fold difference.

Group I has 22 members, including 18 and 4 lincRNAs that showed lower and higher expression levels than their corresponding apcGenes, respectively (Figure 4). Groups II and III have 12 and 3 members, respectively. The expression levels of 34 of the 37 lincRNAs were significantly different (>two-fold changes) from their apcGenes in at least one stage. About 25 of the 34 lincRNAs were expressed at levels lower than those of their apcGenes. Similar expression profiles have been observed in *A. thaliana* and mammalian lincRNAs, in which the majority of these lincRNAs are expressed at approximately 30 to 60-fold lower than the mRNA levels [7], [66]. In contrast, 9 of the 34 lincRNAs were expressed at levels higher than those of their apcGenes.

### Correlations between the developmental expression profiles of lincRNAs and their apcGenes

**Figure 5:**
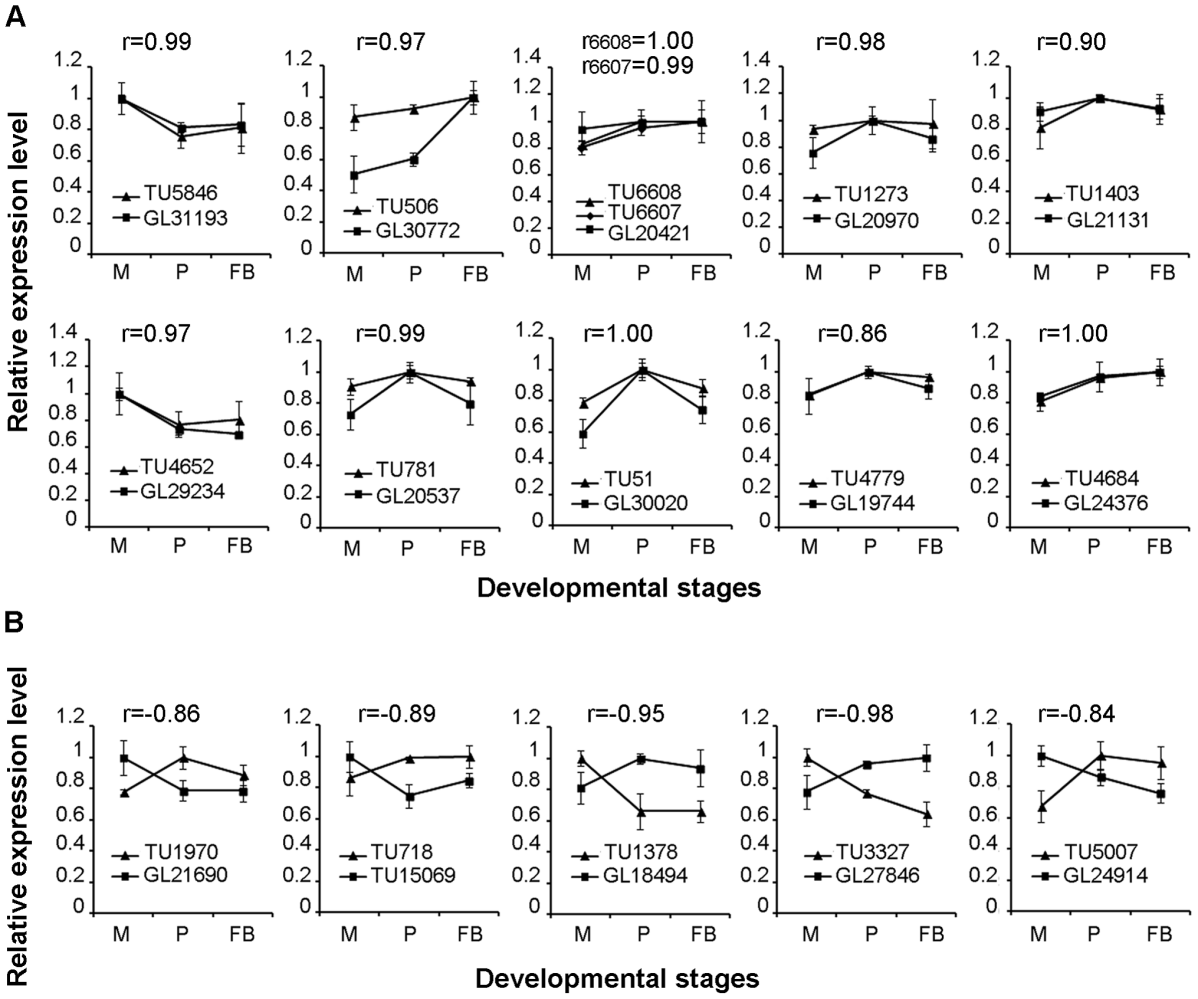
Expression profiles of lincRNAs/apcGenes across the three developmental stages. (A) Expression profiles and Pearson correlation coefficients for lincRNA/apcGene showing positive correlation. (B) Expression profiles and Pearson correlation coefficients for lincRNA/apcGene showing negative correlation. The X axis shows the developmental stages. The Y axis shows the relative expression level, which has been normalized to the highest abundance levels across the three stages. Error bars denote the standard deviations of the three qPCR replicates. M: mycelia; P: primordia; and FB: fruiting bodies; r: Pearson correlation coefficient.

Our objective is to identify those lincRNAs that may regulate the expression of apcGenes. We hypothesize that interacting lincRNA and apcGene should share highly correlated expression profiles. Hence, we calculated the Pearson correlation coefficients (r) of the expression profiles of 37 unidirectional lincRNAs and their apcGenes (Table S5). The expression profiles of 11 lincRNAs were positively correlated with their corresponding apcGenes (r>0.8; Figure 5A). The apcGenes of *TU5846*, *TU506*, *TU6607*, *TU6608*, *TU1273*, and *TU1403* are all CYP450 genes. The apcGenes of *TU4652* and *TU781* are a laccase 3 (*GL29234*) and a glyoxal oxidase precursor (*GL20537*), respectively, which belong to the lignin degradation pathway. In contrast, the apcGenes of *TU51*, *TU4779*, and *TU4684* are an alpha-glucosidase (*GL30020*), a putative hydrolase (*GL19744*), a chitinase 1 (*GL24376*), respectively, all of which belong to CAZy family.

On the other hand, the expression profiles of 5 lincRNAs were negatively correlated with those of their apcGenes (Figure 5B, r<−0.8). The apcGene of *TU1970* is *GL21690*, a squalene synthase belonging to the triterpenoid biosynthetic pathway. In contrast, the apcGenes of *TU718*, *TU1378*, *TU3327*, and *TU5007* are a putative hydrolase (*GL15069*), a Barwin-related endoglucanase (*GL18494*), a putative hydrolase (*GL27846*), and an alpha-amylase (*GL24914*), respectively, which all belong to CAZy family. In summary, in the 16 lincRNA/apcGene pairs that showed significantly positive or negative correlation, 9 apcGenes are involved in wood degradation and 6 apcGenes belong to the CYP450 families, suggesting genes involved in these biological processes be potentially regulated by lincRNAs.

## Discussion

### Genome-wide discovery of lincRNAs in *G. lucidum*

lincRNAs are a subset of lncRNAs located in the intergenic regions of a genome and participate in various cellular processes. Previous studies have described lincRNAs in fungi, animals, and plants [47]. However, no study has reported the presence of lincRNAs in basidiomycetes. In this study, we performed a genome-wide identification of lincRNAs from RNA-Seq data obtained from samples in three different stages in *G. lucidum*. A total of 402 lincRNAs were identified. We hypothesize that some lincRNAs would regulate the expression of their adjacent neighboring protein-coding genes, which we called apcGenes. Hence, we selected 46 lincRNAs whose apcGenes belong to pathways of interests for further examination. We determined the strand specificity using MRA and expression abundance using qPCR for these lincRNAs across the three developmental stages. Thirty-seven of these 46 lincRNAs were transcribed on one strand only and 9 were transcribed on both strands.

Due to the lack of efficient transformation system, we could not directly validate the functions of these lincRNAs by direct knock-in and knock-out. In stead, we used an indirect approach to determine if lincRNA and apcGene are functionally related by examining the correlation of their expression profiles. Among the 37 unidirectional lincRNAs, expression profiles of 11 lincRNAs and apcGenes showed significantly positive correlations (Figure 5A). In contrast, the expression profiles of 5 lincRNAs and apcGenes showed significantly negative correlations (Figure 5B). While correlated expression profiles do suggest possible functionally association, additional experiments are needed to test this hypothesis.

### Potential roles of lincRNAs whose expression are significantly correlated with those of apcGenes

Previous studies suggest that similar spatio-temporal expression profiles for pairs of lncRNA and apcGene are indicative of positive or negative co-operativity of their transcriptional regulation [18]. Our results revealed several pairs of lincRNA and apcGene, whose expression profiles were either positively or negatively correlated. The expression profiles of 11 pairs of lincRNA and apcGene exhibited a significantly positive correlation (Figure 5A). In this group, 9 of these 11 lincRNAs located in the 5′ region of the corresponding apcGenes (Figure 3A and 3B), in which seven lincRNAs (including *TU5846*, *TU6607*, *TU6608*, *TU1403*, *TU781*, *TU51*, and *TU4779*) were transcribed in the same direction and two lincRNAs (*TU506* and *TU4684*) were transcribed in the opposite orientation. The two remaining lincRNAs (*TU1273* and *TU4652*) were located in the 3′ region of their apcGenes and transcribed in the same direction as their apcGenes.

Positively correlated lincRNAs/apcGenes have been reported in several organisms. In the fission yeast *Schizosaccharomyces pombe*, glucose starvation causes the chromatin of multiple non-coding loci to open at the promoter of a gluconeogenesis enzyme gene *fbp1^+^*. This leads to the removal of TUP co-repressors and the binding of transcription factors (Rst2) to the promoter, which ultimately induce the expression of *fbp1^+^* gene [69]. In another example, chromosomal looping transports a lincRNA/activating factor complex into close proximity to target genes, thereby inducing histone H3 lysine-4 trimethylation and gene transcription [70], [71]. By analogy, we can hypothesize that positively correlated lincRNAs could possibly activate apcGenes via a *cis*-transcriptional regulatory mechanism. However, we can't exclude the possibility that lincRNA/apcGene might under the control of a similar transcriptional regulatory mechanism.

On the other hand, the expression profiles of five lincRNAs/apcGenes pairs exhibited significantly negative correlations (Figure 5B). In this group, four of five lincRNAs located in the 5′ region of their apcGenes (Figure 3A and 3B), in which three (*TU1970*, *TU3327*, and *TU5007*) were transcribed in the opposite direction. *TU718* was transcribed in the same direction. The other lincRNA (*TU1378*) located in the 3′ region of its apcGenes and was transcribed in the same direction (Figure 3A and 3B).

Negatively correlated lincRNAs/apcGenes have also been reported in yeasts, plants, and mammals. In the first example, the upstream non-coding locus SRG1 negatively regulates the expression of a key enzyme in serine synthesis (SRE3) in *S. cerevisiae* by directing nucleosome assembly. The transcription of lncRNA in nucleosome assembly may contribute to nucleosome positioning on a genome-wide scale and negatively regulate the transcription of adjacent genes [72]. In the second example, lincRNAs can also recruit chromatin modification complexes to downregulate the expression of target genes. In humans, lincRNA *Xist* binds to the PRC2 complex, thereby inducing histone H3 lysine-27 trimethylation and transcriptional silencing of genes on the X chromosome [73]. How lincRNA in this group regulated the expression of their apcGenes should be interesting topics of future studies.

### Potential functions of bidirectionally transcribed lincRNAs

Recent studies have identified two groups of lncRNAs, called eRNAs and enhancer-like RNAs. Both of them may function to enhance gene expression [43], [44], [74]. We have identified nine lincRNAs that are not only transcribed bidirectionally but also have poly-A tails, as they were identified from samples amplified using oligo-dT primers (Figures 3B, C). As a result, these lincRNAs seem to possess characteristics of both eRNAs and enhancer-like RNAs. Whether they belong to eRNAs or enhancer-like RNAs needs further investigation. Among them, six located at the 5′ end of their apcGenes. Three (*TU4312*, *TU4313*, and *TU4314*) of the six lincRNAs were adjacent to the gene *GL24088,* encoding a hydroxymethylglutaryl-CoA reductase (HMGR). HMGR is involved in isopentenyl diphosphate production in the MVA pathway, which is the crucial precursor of triterpenoids. Interestingly, these three lincRNAs also showed distinct expression profiles. While *TU4313* and *TU4314* are expressed bidirectionally across the three stages (Figure 3C), *TU4312* was expressed bidirectionally in the primordia and expressed in the forward direction only in the mycelia or fruiting bodies. As HMGR is a rate-limiting enzyme in the MVA pathway [75], the presence of these three lincRNAs with distinct expression profiles may reflect a novel regulatory mechanism of the MVA pathway.

For the other three lincRNAs located at the 5′ end of their apcGenes, *TU1567* was adjacent to a pheromone B alpha 3 receptor gene. *TU1567* was expressed bidirectionally only in the mycelia, consistent with its possible involvement in the mating process (Figure 3C, Table S4). Two bidirectional lincRNAs (*TU532* and *TU3423*) were adjacent to the CAZy family genes and showed developmentally preferential expression among the three stages (Figure 3C, Table S4). *TU532* was expressed bidirectionally in the primordia and expressed in the reverse direction in the mycelia or fruiting bodies (Figure 3C, Table S4). For *TU3423*, bidirectional transcription was not detected in the fruiting bodies (Figure 3C, Table S4). These two lincRNAs may be involved in carbohydrate metabolism as indicated by their expression profiles.

### Some technical considerations for this study

Several limitations might have affected our ability to accurately identify lncRNAs in this study. These include (1) the lack of well-defined gene models in *G. lucidum* genome and (2) the use of non-strand specific RNA-Seq dataset. The gene models used in this study were reported in our previous study [3]. However, the 5′ UTR and 3′ UTR of the gene models have not been determined experimentally. In similar studies, a distance cutoff was used to distinguish separate transcripts [7]. However, the lack of well-defined gene models has precluded us from selecting any proper distance cutoff to distinguish separate transcripts. In stead, we used differential gene expression as a criterion to determine if two adjacent transcripts might come from the same transcript (Figure 4). Our rationale is that if two adjacent transcripts represent distinct transcript, their expression levels should differ significantly. As shown in Figure 4, the majority of lincRNAs/apcGenes under study has shown significantly different expression abundance, suggest that they indeed represent unique transcripts.

On the other hand, the use of non strand-specific RNA-Seq dataset has prevented us from identifying natural antisense ncRNAs. In stead, we can only identify lincRNAs. Future studies using stand-specific RNA-Seq dataset would allow us to differentiate overlapping transcripts that are transcribed from different strand, consequently, to identify natural antisense ncRNAs.

## Supporting Information

Figure S1 **Size distribution of the 402 lincRNA candidates.**
(TIF)

Figure S2 **Classifications of apcGenes.** (A) Gene Ontology (GO) classifications for the apcGenes. (B) The KEGG function annotation of the apcGenes. Distribution of apcGenes in different KEGG categories: I. Metabolism; II. Genetic Information Processing; III. Environmental Information Processing; IV. Cellular Processes; and V. Human Diseases.
(TIF)

Table S1 **Primers used in MRA and qPCR experiments.**
(XLS)

Table S2 **Detailed information of the 402 lincRNAs and their 697 apcGenes.**
(XLS)

Table S3 **List of apcGenes annotated as transcription factors.**
(XLS)

Table S4 **List of lincRNA/apcGene pairs selected for analysis.**
(XLS)

Table S5 **List of Pearson correlation coefficients of expression profiles between the lincRNA and its apcGene.**
(XLS)

Text S1 **Sequences of the 402 putative lincRNA genes.**
(DOC)
