# Supplementary figures and images for: Genome-Wide Identification and Characterization of Long Intergenic Non-Coding RNAs in Ganoderma lucidum
Source: PLoS One. 2014 Jun 16;9(6):e99442. doi: 10.1371/journal.pone.0099442 (PMC4059649; doi:10.1371/journal.pone.0099442)

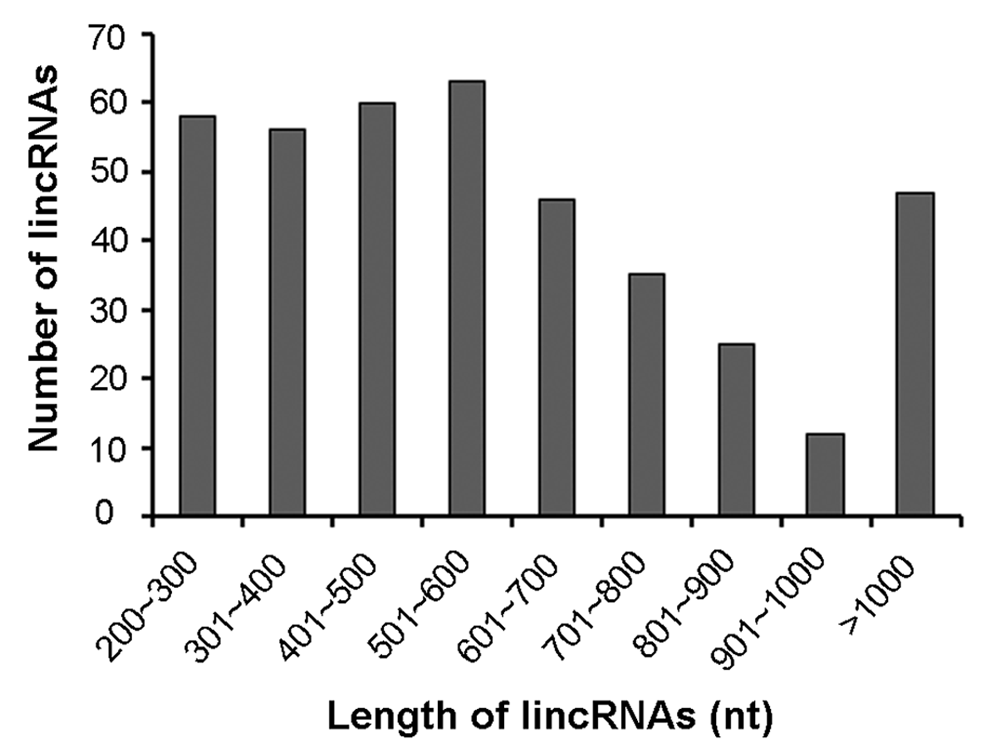

Supplement: Figure S1 — Size distribution of the 402 lincRNA candidates. (TIF) [file pone.0099442.s001.tif]

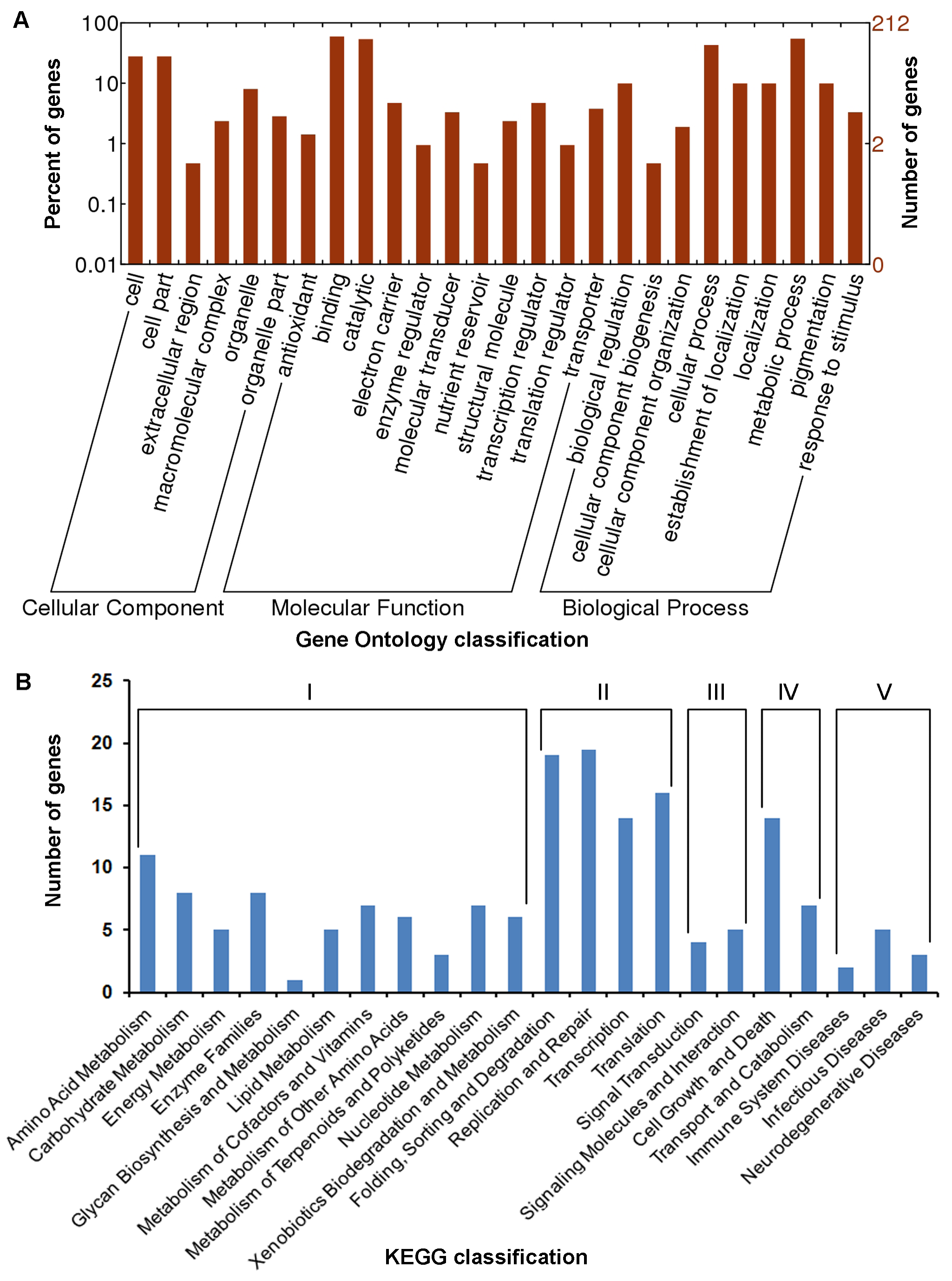

Supplement: Figure S2 — Classifications of apcGenes. (A) Gene Ontology (GO) classifications for the apcGenes. (B) The KEGG function annotation of the apcGenes. Distribution of apcGenes in different KEGG categories: I. Metabolism; II. Genetic Information Processing; III. Environmental Information Processing; IV. Cellular Processes; and V. Human Diseases. (TIF) [file pone.0099442.s002.tif]
