# Supplementary material for: Genome-Wide Identification and Characterization of Long Intergenic Non-Coding RNAs in Ganoderma lucidum
Source: PLoS One. 2014 Jun 16;9(6):e99442. doi: 10.1371/journal.pone.0099442 (PMC4059649; doi:10.1371/journal.pone.0099442)
Supplement: Text S1 — Sequences of the 402 putative lincRNA genes. (DOC) [file pone.0099442.s008.doc]

**Text S1. Sequences of 402 putative lincRNA genes**

**Note: The ID of each lincRNA sequence contains the following information concatenated with “-“: TU ID, scaffold number, start and end positions of the lincRNA.**

>TU10-GaLu96scf_1-39368-39716 ACAGCGAGTCGCTGAAAGGAGTAGTGACGTACACATCCGACTTATATGGTCCTCCCAAGGTCCTCCCGCCCAATGCATGTCGTTCCCGGTCAGGCGGGCAGCTACAAGACGATGTCGGCCTTCGCGGCAGCCCAATCGGAATCCATGTATGTAATACCAGTTGCGGATTCGACGAAATGGCTTGTCATGATCAGTTGCTAGTCGTTTTGTGAGGCGTTGAAGGCGTGAGGGCCGGTTTCAGTATAGTGAGACACAAGCAGTAAGATAAAGAGTGGCAGCAGATGATGCGGAAAGAAAGCCATGCTCGCGACAAGTCGAAGGAAGCAAGGGTCCACCCTTGGCACCACCT

>TU102-GaLu96scf_1-707676-708210 GCGCGTGCATATATGCGACTTCTAAAAGCCGGCGGGGCGTTTTAAACCATCTGACCGCAAGGCAGTTCTGATGCTGACGCTACGCCCATCTAGATCCCATATATCCCCTCTCAAAGACCCAGATATCACACCTTGATCAGCCGCTTGTCGAATAAGCGTGTGGACATGGGAATCGGGCTCATTTATCCGGGGCTCATGACGACTGTGCCTGCAGCTGTGCGCGCTTGCGCCTGTATATGGTTGGGTTCCCTTCTATTCTTCTGGGCCCGTTCTCATGGTGCCAGTGAAGGTCTTCTTAGTTGGCGCTTCGCGAAGCATCGTCACATGCAAATATGGTGCGGTATCCGTTTCTTTCACTCACCACCGTTTCATGAGGTGCACCTAACCTTTCTTGAGCATAGGTAATAGACTAGGTCGTGCGGGGTCGCTGGCTCTGAGGATCATCCCTCCCCAATGCGAGAGAGCGCGCCGTTCCCGCTTCGGGTTTCCTTAGCGTTGTAGCAGCGCAGCGCGCGCACACTTGACACCGTTAGGG

>TU1055-GaLu96scf_11-1218312-1218928 CCAAGGCCTCCAACATGTCGGTCCTGTCGTCGATGGAGGACGGAGGAAGAAAGGTGTCTAAGAATTGCTGGACTGGCATAGGACCCACAATAGACCGTTTGCAATCTTCGAGGAGATTTAAATTTTGATCCGTCGCGAAGTGGGGGTCCTGCCTGCCAGTCTGGAGGAGAGTATGGTTGAGAGCCAAAGGGGTGTTGTATACACAAGACGGAACCTCGAGAACGGGAGCACTATTAGGCATGACTGAATACGATGGAGGAACTTGCAAGAAGAAAAACAATGGGGTATGCGTAGAAAGTGAAGGGGGAAGGAGGTTTGTGGACTGTTTGTGGCACTCGCGCGCCGACTGGATATGTCGGGCCAGCGGCGTGGCGTTGATTTTGTGGACACTACAGGCATGTAATTATCGCGTCATGAAACTGCTCTGACTTCAGGGTGGTAGGACGGGGGATAAGTTAAAAAAATGCTGCTGGCGTATAATACTGTAAGTCGCTGCCGGTGGCTCAGTCCATAATAGTAGTCGGTCTCATCTCTGGGACATTGAGAATGGTGCGTTTTTTGTTGCTGCTGAGCCGGGAAGACACGGTGGGGCCATGCCCCGGAGCAAGGCTGCATGA

>TU1059-GaLu96scf_11-1224701-1225155 GAAAAATACTGGAAAGGCTCGATATCTTCAAAAGAAGCCTCGATCGCGCCGGTCGACCGCGTTTATTCGGTACACTGAATACTTCTATTGGGGTCCAAAAATAGCTACACAGGACGGGCGTATGCTACTACTCGCGCGCTCGCGCGCGGGACTGTACGGGCTCGCGCCCGACCCACCCGCCGCGCGCACACCAAAGTGTTGCGCGCGCCTTCCCGCTCGTTCTGCCCTGGCGCCCACTCAATCCGGCTGCGCTCCTTCCCACCGCGCGCTCCTCTGATTGAGTTCCGCGGACCCGTCGCGTGCGGTCCTAACGATGACGACCATTAGATGACGTCATAGAGCATGCGTCCGCATCACATACCTGTATTGCGCGCGAGGCGCGCGCCCGAGAGAACAAAAGGGAGGGAGGAGGCAGAGGGTAGCGGAGGTCGCACAGTGGGGAGATGGTGGGGGGA

>TU106-GaLu96scf_1-716235-716476 CACAAACGCCCTCTCCCCGCGCCCCGCGACCCGTGCACCGGGCACCCAGCCCGGCCACGTCACGTCATCGAGGAATGGTCGATGTCGTATCGCAAGCCTCGTTGCAACCCCAGTGACGTCCGTCCGGCCGCACGCTGACACGATGACATCGAGACGAAGACGAAGACGAACCGCAGGCCGCGCGCTGCTGATTGGCTGAGGGGAGGGCCCGTCCGGATTCGGAACACCGAGCAAGCCACGCC

>TU107-GaLu96scf_1-716567-717262 GTGGCGCCGCTGACAGTGGCGTCGTCCACCAGCGTCCACCGTGTCCAGGACCCAGTGGGCCGGCGACCTGATCGCACCGCGCGCCAGTCACTAGGCCTCAGGCTCGTCCGAATCAGGCTCGCGGCTCCACAGTCGATGTTCAACGTTCATTCCCGCAAGGGGCAGCAGTTGGGGCCTCGGCGGCCGATCCCATCGATACCAGACGAGCGCGCGTGGCGGGAAAGTCCTGGAAGCGGAAGCCTGGAACGGGAACACTTTGCGGTTCCCCACTTCGAAGTTCTCAGCAGCATGATCAAGCGGGCCGTGGAACGCGAACCATGGCGGACCTTTCACCTTTCACGGCATCTGGCCGGCATCTGCGCACAGTGGTCGCTTCTCGCCAGTTTCTCGCCCGATACCCGCTGCATACTTAACTTATGCGCGATGCGATGAGATGCGACGCGTATCAAATCGGCCGCGCGAGCGTTCGGCTAGCGACGCGGACATCTCGAGAACGTTCATCCGTGCATCCGAGCCGTGCGAGGTGTTGAGGGCGAGCGCGGGCCAGCTGCAGTGTGCAGTGGGCGCATGTCTGGACACACTCAATCAGTCTCAGTCGCTAAAGTCCGAGTCAATTGCCCTCGCCATCTTTTCGAACTCTTTCTTACGTGCGCACGGCGGACCGAGCGAAAGCCGAGTGTTCGCCCTCGTAATTGA

>TU1119-GaLu96scf_12-99812-100388 GGACTGGAACGTCGACATAGTGCTCGTCACTCGATGACTCTCGAGCTCGAAAGAGGATATCAGATAGTACAGGCCTGGCTCGCAGAACGCCAACGACGCCGAATGGACGATCGGGATCCTAAGACCCCCCAGAGTGAATTCAGACTCCCCGCGTTCGTCAACTTCCATTTCCTCGACCCCGGCTCCGACTGCGATGAAATATTCTGGCAAGGTTCGTTCTCTTCTCCCCATACCCTTTGCTTTTTACCATCTCGCACAATCCATGATTCCCCTTAGATCCCCTGACATTCACCCCCAGGCGCGGCAGAAACGGTTATGTCGACTCTTGGGGCATATATCCCGTTCGTCACTCTCCTTTGCTCCAAACTCACCCCCATTCCTTACCTCCAACACATAGCATGATGACGCCGGCTTCAGAGACATCGATATACAACGTATCATGACACCTGTGTCAGCCAGGGCAGTTTATCTTTCCCCTTTACTTACTGAAGTTCATAGATCATCTTCTCCAGTAAGGACAGACGCTGTATGTATGGATTAACGTGTGTTAGCTCTAGTTAACCTTCGAACAAGCTCT

>TU1131-GaLu96scf_12-140962-141538 ACATAATTCATTCGTTGCACAGGGATAGGGACTACAGCTACAATAATCTCAAGGGTCCGTATGATATCGATACATCTCTGTGCAAGCGGCTCGTGGTACGGTATCTGTTGGATATGAGAAGACATATGCATGAGTGAGCCCACACAGATGTCCACGATTTGAGCAAGTAGTGCGTACCCTTCTGCGTCCTAGCCCATGCTAACAACAGCAACACAGTGGGATGGGTATGATGTAACAGATACTTCCGTCCTAGAACATAGTTAATAAGCTGAAACAGATTTCGGAGATCGGGCACATACAGGACATGGGATGCAGCCCCCACGAATCCGAGCAGCAGGACCTGACGCGTTAGGTTGGCCAGAAGGGAAGGACATGGCTGCAACTGGCTCAGGTTGTTTGGATTGTACGAGAGGGGGGTTTTGGTGGGCCGCAGGGACGTGTGCAGGAGCAGGACGGCTAACGATGTGCTCTTGAGACATTGCGAGGAATTAAAAGGAGACTTGAATGCACCATGGCCGTTCCGATGGGTCTTTATACGCCCGCGGATGTGAGAGGCTCAAGGAGTCTGATCATGAGT

>TU1133-GaLu96scf_12-143655-144209 CTAGTGTCACCGACACCGAAGACCCCGGTCGAGTGTGCGACAACTCGAACGTTGTTGTTCCCGGCTCGGCTGCTGTGCCTCGCTGTCCATCCACTCGGAATTCAAGGCCATGTGCATGAAGGGGCTCGATTTCCGCCTGCCGCAGACAGATGCCTCACCCTCGTCCTCCCGTCGGCAATCCCCCTCCCATTCCAATCGGGCACTAGTACTGGCAGGTCAAGCTAGCGCGCGCGGTGGGTGGTGAGTTGTCTTTGTTTCAACTCCTCTGGCCAATCTGCTGCTGCTATGTCTCGTTTCCAAGAGGGCTTTATCGTCAGAATCAAGCAAGGCGCTGGGAAGAATGAGCCTTGGGTCATTGGAACTCGGTGGGCAGACAAGTAGACGAATGAATTCGAAGGAGCCTACCGCCGGGCGGACGGGAGCTGCGGGAGCATGGGAGTCGTAGCTGCGGTCTCGACGTCTCGACGAACAGACTTGTCGTTCTTCTTCTTCTTCCAGGCCCCGCCCCCACATTGCATGCTCTCACTTTCCCTTTCCGTTTGTGGACCGCGGCGA

>TU1158-GaLu96scf_12-355917-356680 GTTCACAGACAGCTGACAGCCATCCGGTCCGCCAACGTTAACGTCGCGCGGTACGTCACTATCTGCAGCGCTCGGAGCGAGGAATGGCGCATTGACAAAATGCGGAGTGGGCCTAAAATTCATACTTATGAGGCAACTGTCAGCGCGAAACAGTGTGGAGTGACAGTACTTACTTGAACGACGACGAGGGGGCCTGCGCGGTCAGGCGGCGGCCGCACTTGCTCGGCAAAGTAAAACAGTAACAGGGGCGTGTTCCGAAGTTGGTAGAGTCCAGCGCTCGACGTCGGCGTCCGAGGGCATAGGGAAAGCGGGCCGTGAGTGCGGGGGACACATGTCGCTTGTTTGGCCGTTTCAAGGAGAGAAAACCATGCGCTCGCCCTCGTTGGAGATATACGGGTGGCGATTATCGCGATCTGCACGGAACTGGCGATCGGTACGTAGCAGATGAGAGGCGCCCCGGGACAGTCCGAAGGGGGGTGGTCAACATCCTATTCAATGTTGGCATCGAGCGCGAATCGCGAGAGAAAGCCGAGGACGAAACATCTGAAAGGGAAAGGAGTTCATGGGACTGCGGCGCTGACATGAGCTGACAAGAGGTGCAGCTGAAGATGAGTGAGATTTGTTAGACTTAGGAATCTGCGGGCGGTATGTTGGAGCGCGGTGAGGAGGGCCCGAGGAATGGGGGAAGAAAGAGGTTTTCGGTGAGAAGAAAGAGCCATAGGAAGGAGGACACGTGAGAAACGGACAGGTTAAGTCAAAGGGGG

>TU116-GaLu96scf_1-794208-794677 TGTTTACTCAAATCGTGTCCACGCCGCCAGCGGATTGTCTCTCAACATTTTCTACAGTTTTCAATGCCGACAAAAGCTGTCAAAGGCCAGATGTATGCTAACTTTGTCGAATACACCTTCAAAGTTGTCTTCCCTAACCGATGCTCTCGTTTCATTTTCCATCCTTCCCGAGTTCGTCGAGGGCAAGGAGCTGCGCCTCTTTCTTTCTAGTGTCTCCTCGTCCTCAGATACAGGGCACCGATACTCAAGGCATTCGGTCCGTTCACCCTATCCCCTGTCCTGCTTGTCTCGCCCGCTCAGCCCTTCTCATCGTCTGATGGCCAAGCCTCCATTCCAGGTAGCATGGTCCTCAAACTGTACGACCGGTGCTGTCTTGTGAATGCGCGCGATAACTATGATGAGGGCAAGGCATGGTCCCCCGACAAGGAGCCCGAATACCGGCAGTACCTCAAGGATGTAGCGACCGGCAC

>TU1170-GaLu96scf_12-401863-402267 GTCAGGGTCCACTCCGAGAATCGGTGCGTTGTCGGGGCTTTAGCGCACGTCTAACACATTTCTGACTTCTCGCCTTCCTTCCCGCGCCCATCGTCTGAACGGCCGCGAATACAGGTGTTCGACGGTTTCCGCTGCGTTGTCTGACGGGATTCCGATGCGGCCGCTCAATCACTCGAATAAGCAATGAGTGTAAGCCTCGGGTTCGAGGGCCTTGTATCCGATCTCGGTTCCTGGGATTTCGTATTCTGACGGCATATATACGCATGAAATGATTTTGCAGTTCAAAGATGAATAATGTCGTTCCCTCTGACCCTTCTATCAGCCTCGGAGCAATTCGATGAGGAAGCGAAGCCAACGCTGAGCGTGGAGTCCTACCCAAATCCGCGGATGCCGTGGGTGTCCTTA

>TU1187-GaLu96scf_12-508246-508763 AGCGCCCACCCCAATATCTGTGTCGGAACGGGACGGGAATGAGTATGATATTACCCCCAGTGAAGCGAGTCGTGAATGAGCTTACCGGGTCAATGCAGCGGCTGCGCGATCCGAGACATGTTCTTTCAGAAACTTTGGACCTTGGGCTCCACACCTTTCCTGCCATGGTGAGCACATCCAGGTCTATGGTGCATGGATCAAGTGAATGTATACGTACGTGGCGTCGACCGTGGCAAGGGCTGCCGTAAGGTGCATACTGAGCGATGCAGTGGTTGAACAAAAGAAAAAAAAATCGATATTTTCATCAGCCATATGCAATGAGGGTGTTCGGCGTCAGAAAGACCATGGTAGAGGAAGCAGAGTTTGAGACAAATCCAAGATGATAGGCAAGGAAAGGCGGAGGGAGGCGGAGGGAGGCAGAGGAAGGTGGTGGTGGATGGAAGTGAAGAAGACGTTGGATAAGCGCCTCAAGGGACCGGGGCGGAAGTCGAGACTCGAGGAGGACGGGGACTTGTCGA

>TU1255-GaLu96scf_12-791636-792193 CGCGTGCTTGCCGGGCATTTCCGACGTAGGATACGAGCCGCCGTTGTACATTTTCGGACCCATCCGTTTGCCTCTGTACCACGACGACCCCCGTACAGGACAACCGGAATCTATCTCTTTGCTTCACTCGGACGACATTTTTCGCTTGCTCCAACATACTCGACCGGACTCGGCGCGTTTTCCGTCTGCAGACCATCTAGATCTGGCAAGTGGCCATTACCATCCTAGTGTTTGACTTATTGGCGGCTGAAACCTCACTATACACAGGCTGAAACAAGATACCCGGTTCACACCCCATAGATTGTGTCAACTGACGTTGCAGAGGCCACTGTGGGAGTAATAGTCAGCAAAGCGAAACAGTACAGCGTTCCTAAATTTCTGACAACACTTCACCTCCCGTGCTATAGAATAATACCAGTATATCCGGCGCACTGCGGCCCATCAATTCATCGGGCGGGTCTTGGAGCGTCCTGCTCAGCTCAAGGACCGCCATTTGTCCGTAAATTGCGGAGTGCCCAAGTTCGTCCGTAGGTAGGTAGTAGGGAACCCCACCAAGTT

>TU1272-GaLu96scf_12-986870-987643 CACAATTAGCAAGAGGATCACAGAGGATCACTCACCATCAGCTTGGGACGTCCTTTCTCTTTTCCACCTTCACTTCCTTTCGACATTACCGCGACATGCTCGCCAAACACCGTGTGCACAAATCCTGCGGAAGTAGAATTCCCGCGCATTCCTTGGGGGTGTCTGGGTCGAAAGTACTGTATGTCACAGGCACAGGTTGGAAAAATGGTGCTGACACGTTCCTATAGGTGCAGCGCATGGTCCCGGCGAAGTGGCCACCATCGGCCAACTCGTGCGACCGACTGACGACAAAACCCTCTATAGCGGTCCCCGCCTGCCAGGTATGTAGTGTGCACACGCTGCGTCCTTGCGTTTCGCTTACGCGACATCCTAGCGCATGCGGTATGCTGGAAATACTGAACAGGCTCTCTAGATCTCGCCCGTGTGAGCTTTGAATTTGTCGTCTAGTAGGTCTGGCTCGTGTCCGAAAAATCTTTGAGATTTACAGACAATGTTGCAGAACCGTGGGGCCTTTCGAGGCCTTTCGGCGTCCCTCCACGCCTCGTGTCTTTCTCAATCACTGTGCACTGTGCCACCCGGGCACTGCCTGGATGTGCGCTTCCGGCGTCCGCTCATCTCCGTGTCCCAACGAGGCCTCGGTGGCATGGGGTGGTGGCACGGCACAATCGACTTATACTCACACGTGGTCCATGACGCTAACTTGTTTGTCCTCCGGTGGCAGTTATGAACTTCACATGACACAAGTGCGTACGTGCACTCGTAAGCCAGCTGGGT

>TU1273-GaLu96scf_12-987803-988339 CTGACGTGTGGGGTACTGACCGTGCTCCAAGGACCAAGGCAGAGTCAATGGTCGAGACTCGAGAAGAGACAATCTGAGATGAGCAACGCGTATGTGGCCTCGATTACGTCGCGTTTCTCACAGAGCCAGGTGTATTTTCTAATGCTTCGAACGTTCTATACGATGAAGATAATTAGGATGACCCTCCGCCTAAGTGCGATGACGACCTGGGAACAAGTCGAGGACAAAGGGAAAGACGAACGCTCCAGCCAACGTCCAGCTGTCGTGTGTTGCGCTTGACTGCAACAGAACACAAGTTGGAGCAGAGGAGTGAGAAGGACAGCACGTCTGAAACGTCGGTGAGATAACTGCACACTTCGCGCAGTTCGTCCAAGTTGAGTTGAGCCATGTTAGTTGGCAGATTGTAAGGCGGTCTGCCCGAGAAATGTCAGGTGGTTAGATTCGAGACACGAGAACAGTAGGAGATAAAAGGAAGACATTCAGCGAGTGTAACGAGCCTCTATCCGGTCCAGTTCCGTTGGTAAAGGACTGAGAGAA

>TU130-GaLu96scf_1-877849-878226 TACTATCAGTATTCTAGGTAATAACCCGCCGTCAATCCTTCCCCTCTCTGGCGGACCAAGTCTTTCTGAATAAAGTTAAGTATTTTACTCATTGACAGTCACGACTCACAATGCGCCGAGGACACTGGAGACTGGAGGGCTCATCCGCAGTCACTGGAAGGTGTCGAGATGTTGGTATGTCGGATTGGTGAGATGATCGTGGCTAGAGGGTACTGTAGCCGTGATTCGACCGTGCTGGAAGCGGATTTGGATGGGTCTGTGTCGTCGAAAGTGAGACTGGATCCTTATCCTACAATACCTGTGGCCAGGAGAGGAGAGGAGTTAATGGAGAGGGTTGATGGAGAGGGAGATGAAGAAAGTTTAAGGAAAGAGGAGGAG

>TU1331-GaLu96scf_13-86495-86872 GCGTGCTGAGTTGTGGCGGCCGCAAGGATTGGTACGGGCGAATCGACCGGTTGTGGTGGAGGGAGGCGGTGGCTCGCGAGTGACTTGGTTTCCGGGGGAATCGGAGGACGCTGCTCGCGGCAGCTCGCCGCTCGGCCGGTACTTGGATGGACTGCACGCTGTGAGTGGAAGGGGACTGCGGGTGGCCGTTGACGTTGATGGTAGGCATGGGGTTTATGGTTTGTGATTGTCGCGTGCGGGAGGATGTTGGAGCGAGAGGATGCGTACCTGTACTTAGACCGTTATGACAGACCGCGAGCGCGGCCACTGTAATAGGGAGGAGATGATGCAGAAATGTACGACATGTTCAAGGCTTCTGTGCTGATGATCAGGCCTCAG

>TU1338-GaLu96scf_13-154365-155182 TAACCTGTACATTCAGCCATCCGTAGGCACACGTTACAAGTGAAAATTCGGACCCGCCAAACGACGGCAGCCGAATTCCAGCACACCTGATATCTCGTCAGCGACACTTCGGAAGCCTTGGCGCCATCTACTGCGTGTGATACGTACAAGCGCTATCCGCTAGTCCGGCGATATATAAGACCAATAGGCTCTTAGTGCACATGTGGCCGACCTGTATCTTCAGTAGTGGGATATCCCTCATGAACCTCAGGGCATCTGTAGCGTCTTGACGGCATCCGTAGCATGAGGTATGATCCTACACACGATGCGAGATATCTCACTCACAGCGTCATCAGATTGTGGTCAACGTTCTCCTCGTTTTGACTCTATGGAAGGATCACGGCGCCAAAGGCGGCTTCATCTTTGCTGAATTCGATGGATCGTCGCTGGTGCATGTCCGGGAGAGTAGCCGTCTAGTAGACGACTTGTATCGGCCCCATGCAGGGCACCAGGAGGTGATTTGGATTGATTTGTTAGAACTAGCCGAGGATATTCAGGAAGAAAGGGTCCGTTACCGGTACCGTAGCACAGCGGGATATCCCAATGATCACATAGGCAGGACCACATCAAGCCCGGGCTTGAGATATCTAACTGTAAGCCTTCCGGGTGACGTCCGAAAGCCTACTCCGCGCCTTCGATGCAGCCGGTGGAGTGTCTTCCAGTTCCTCGTTCTCGTAGTGAACCCTCCTTCCTTCAACCACCTCACCGTTAGGGCGATGTTCAGGTTCAGGTTGCATACCCACCACCGCAACCCCCGCCCCCGGGGCAAGCCGATTGCA

>TU1361-GaLu96scf_13-274260-274463 GTACAGATGAAACCCTCTTAACCCATACCCACTATGACCCGGCGCTGTCCCCCATGTCAAGTCCGTGGGTCCGCAAAGCTATGGAGTATCGTAGCTTATTCATGCAGCTCCATAATTCCCTCTTACCCCATACCTGTTACCTTGCGGACCCCAGAGTTATTGGCTAAGCGCGCGTATGGGTTAAGAGGGTTTCATCTGTACCTG

>TU1364-GaLu96scf_13-459055-459766 ACTCACAGCACAATGCTACACCGCTACCTCCATACCGCACCCATCTTATAGACAAATTGCGTAAATTTCCTTCGTACAAGGTCTACCATCTATATCCGCCGGATGTGAGTGAATCGGCCCTACCACCTGACCTTACTCACCGAACTCGTCCTCATGACAGGGTCGACCGCCCTTCCATCCCATCCGACACAAATTCCGTAACCGCAGCCTGAGGACACCGAAACTCGGGACATCTGTATCTTTGAGCGGCTGTTGTGAGCAGACATGATGCCATTCGTCACCCTCTGTGTGACTTCTACCTTGGTTCATTGAAAAATCGTGAGTACCTCCTCGATGACCATATGGCTGGTCATTCGCTGATTCCGTCCGCCCTTATTTTCAGGTCATTAGTATTCCGTACCCTCGGCTACCCTTGGAGGGCTGCGCCCCGGTCCCCGGCAGATAGCCCCCTTTTCTCGACGCCTCGTTATCGACCCTAGACATTACAACTCGAAGACGTGAACACAAGTGGCTCACGAAGTTTGCAACTTCTTGACGCTTCAAGCTTCAAGTCGTCCAGTCGAGCATCCACTTCATAGCGGTCAATTCACGAGAATTTTAACTTCAAATTAAATGTGCATCAGCAATGGAGTATTCACTAGCATGAATTTGAACATTTCACCAGCATGGTTGGGCGCGAGAGAAGACCTGGAGGGGCAAACTTGAACCTGAT

>TU1376-GaLu96scf_13-490287-490970 AGCCCTATCTTCTTCGCAGACAGCCAACCCACGTACTATGCGTGCATATCTGTCGGATATGCTCCCGTCTCCGTAGAACCCACCCCATCCCCGCACCGCCTGTCGATGAGCAGGCTCCGACCCACCGCGTCGCTATGAATCCGAGACTCAGAATAAGGCAAGCGGACCGGTACCAGGCCTTAAGCTTGTGACTTCTCAAGAGAAGCATATGCATATAGCGGCCTATGGTATGGTACACAACTCTCACCGTCCTGACCGTCTCCCTCCATCTGGTGTTCATCAAGGGAGTCATGTCGTTCACACAGTGAGTGTATCCTTAGCTGCCCAACCTTGAAATTCGGTAGTCAAGCTAGAAGGCCCCGAAACGTGACGAAATCTATCTTATTTAGACCTGGGGAACGCCCTCCGGAGTACCCTCAGTGGTTGTCTCGGCGGCACAGCGGCGATTGACCTCCCTCCGCACGATCACGAGCTGTCAGACTCAATAGAGCTACGGGATATCGATGACGCAGATAACCAGGACACTCCTACGGCGAAGGTGGCTGGAAGCGGTACTACCATGCCTTCACCGCAGCGAACGTCTGCGTCCTCGTGCAACGTGTACTTCTAGGACCTCGCCTCCCTCGCCCAAACTGTGCTCGTATCCCTCGCCGCTGCTGGTCTCTGGATGTTTTCTACACCTGC

>TU1378-GaLu96scf_13-493942-494266 GAATGAACGATCCAAGCCTTACTCGCCACTTATTCCAATGTTACCTCTATGTTCCGCAGTGAACACAACATTGACCTCCATACTCCATAAGATTCAGTCTAGGAGCCTGAATAGTGCCAATGAGCCATGTACTTGGCGTGGGCTGCATGTATGCGTATGGATTCCGACGTGCTAGTGCCATCCAAGCCCTCGAAGGGCTGACGCATATGCATATGATGAGTTGAAAGGTAACTAGATACAGCTGAGGTTATCCTCACCCAACTGGTCTTGGACTCTTGGCATGCGGCGGTCAGCACAGTGGCCCGTGGGGAGATGGGATGGATGG

>TU1387-GaLu96scf_13-620442-620993 CGGTGCCGGTGGCGCCTGCCTGAGAATTAGAACACGACTCGGAGGACCCATTGGGAAGCCCACACTGATGGCGACGGACGGACCCCAGATCCTTGCGCACAATCTCACTCATCCCAAAGGCTAACTCGACACAACGCACACAGGTACCGCTGTTTCACATGTCCCAACCACCAGCTCTTCGGTTAGCCACCGGTTTGTATGTCGGGAGGCAAACACTACACGAGGCACATGGGCGCAATCGACAATAAATGACAATCCCTCGCAGAGCGGAGCAACAAAGCACGGATGCGGAGAGGTGTTCTGTGTGATAGTATACAGTACAATACAACCGGCATGTCCGAGGTAGCAGCGAAAGCAGTAGGTCGGATAGTACAGTACTAGAACGAGCGGCCAGTAGCGTGCAGTGTCCGCTACAGTAAGAACGAGCGACGAGCGACGAGCAGCGTGCACATAACAACAGCAATACCAATACACTTACTGATAATAATCACGAAATCCCCCATATGCGCATGCCTAGAATATCCCAAGACCCCAGTGACCCTCCCCTCATTA

>TU1391-GaLu96scf_13-634442-635050 AACTTGCCGTCGCCGAAATCAATGGTGGTGGGCCAACACCTTTTTGCAGCTCGTATATGCGCTCTGGGATGATGGATGTGCCTAACGGTAAGTGCCCTGGGCTTTCTTATCCACCAGGGTGTGCAAGGAGCAGTTCGAATCAGGCGTTTTGTTCGAGTCTGTTCCTAACCGGCGTACGCTCCGTGATTCGGACTCATTCAAGGATTCTCTCCCCCCATTCACGGTGAGGCGAGAGGATGTATAGCTGCCTAGAGCGCTATATGGTATTCTAGTACCGAGGTATGCTTGAAAGAATCCATCCCCTCGGCCGTATCCGCTAAACGGTTTATTGCCCAATTGCTTTTCTAGAATACATTACGATGCACTTAAGCAAAGCCGCAAGTGAAAGGCATAACATTCCCGGCGTTTTCGGCCTATTGGGACGGGATCGCAAATAATCGGTCAGGAGCGTCTCTCAGGACGCCTCTTCTGAACGACCTCTCCTAACCTCAATCAACCTCGGTAAGCAAGTACTAATTTCTCCTGGATGGACCTGCGGTGCAGCCAGTATTTAATAAGGGTCATCTCTGCCCGCAGGCCTTGAGACCATCGCAAGCGTCGAATGCGATG

>TU1393-GaLu96scf_13-644281-644828 TGTAAGCTCGCCGCTCGAGGGTGCTTCTGGGATGAGCGTGGCGTAGATCTCTGGGGTTCTTACGAGGGTCTTTCTTGTTTCGGCAGCCCATGTGAGCATATTGGGAACTGGGGAAGCATGAGAGAGAAAAAGAGGAGAAGCACGGGGAGTCAGCGGCGGATGACGGGATGATGGCGATTGGGTGTGGATGTGAACGAGGCCGTTTCGTGGATGGGTGGACTTTTGACGTCAGGGTGTGGCCAATCGGATCTAGCGTAGAAGGGTGAAAATTTGGTAACTGAGAACATTGATGAATGCGTCCGAACTAGGAGACTTACTTTCGGGGTCGAGGCGGATCGCCGATTGTGCGAGGGTCGTGCCTCCGATGGAACGCGAAGGGAGCGAATCCTCGATCATATGTGAACCAACAGGGCAAATACACTGGAGATCAGGTCGAACTTTCTCCGGGACTATCCGGAGAAAAGTTCAAATATGCACCAAGTATTCCCAACCTTGTCGTGGCGACTATCCATTGAAAAGCGTGAGAAATGTCCAAGTATTCGCAACAT

>TU1394-GaLu96scf_13-645173-645556 CAGTAGAACATTTCGGAGGACCAGCTATATGCGCATATAGGAGGTGTGTAAGCGCCAACAGGCCGATGTTCAAGTGGCAGAAAAACGTACTGATGGGGCCGCGGCGCAAGCTACCTAATGCGACTCCGAAGGGGACAGCTGTATGCGCCTGAGACTATGCGCGAGGACGAGAAGACGATACGCATGCTTACCATCAGTACGCCACACTACGCCCAGGTCCTCGCCGTAGGAAGGCTGCCGGAATGAGAGGGACGACGATCAGCGCAAACGACAACCTATTAAAGTCCCTGCCAGGACGCACCCAAAATCCCTGGATACTCGACTAGAGGTGGGGGGTCAGTGAGCACCGGGAAACCGACTTGAGGTCAATGAAAGTGGGAGTAG

>TU1395-GaLu96scf_13-646330-647043 TAATCGAGGGATGACATAAGTATATTGGACGAGAAAGCTTCTGGATTAGGTAGGTATCGAAAACGCTGGGAATACACGGGGACAACTTTGAATAGTCTCGGAATTGTCACGAGAAAATTCCGGGGTGATCTCCAGTGATAGGTCATTAGAAGAGCCAAGCAATACTGGATGATGAAGAGGGGACAGAGGTCAGTAAGCCAAGGCTGGGAAGGAAGGGGTTGCTCGCCTTGAGGCAAAGGGCGATTCTTCTGTATGAACGTGGGACATCCGCTGCAATCGCACAGTTGGGGATGAAGCCAAGTAAGTATGTGTCGGTGCTAATGCGCTCTTCGGCATGCTACTGACCTTTGCGCGAGTCTATGTAGAAAGACAACTATAATCTCTTAGCGGAGATGGCCGGATCTGGACGAAGGTATGCGATAGCCCTGTGGGGCCATGGATGATGGCGGCGAAGAAGGCGGGGTTAGTGGGCCTTTCATGTAGATAAGAGGATCATCCTCAGCTTAAAAATTGGAGGCTATGAAAAGGGAGCGAGCTGAGCATTTCGTAGTACGGTACGATGCCATGCGTGCGGATCGTGAAGTTGATTAACAATAATGCTGTGTACTTTCTGAAGGGAAAAAATTCGCACATAGTCTCAGCTACATGCGTGATATGCTTACTCACCAGGACATTCCAAACTGAACCTCTGTAAGTAACTCCCTGGTAACTCCA

>TU1403-GaLu96scf_13-681009-682346 TGCTCCTCACCCCGACCATGATCGGATTTTTGTTACTTTAATACTTATTCCTGTCCAGCTCCATCGATCCAAGGAGCCACACACCCATCACTTCCCCCCCTTGTGGAGCGCTTCACTCCATAGATCAGGGCCGACGCCCGAGTCGAATGTCGACCGACCGAATTCGCAATGTTTGAAATGCATCCAAACACAATCCGTGCAACTTGGTTTTCCCATTGCAAGAAAACGGGGTGTGCTAATTATTTTTGCCCATTTTATCGCCCCCTTGCAGTTGTAGTACTTGCCAATGCGCTGATGATCATGTCCAGATGTTCTTCCGCACCAACATCTCATCTGAACCATGCGATCCCAACAGAGGACTCACGTTTTCGGGGAGCATGGTGGGGACGTAGGTTCGTACCAACCCCTCCCACGTCCTCGCGGCACGTGGCGGGTTTGGTATGAGACCAGGTGAGGATGAGAATCGGGATTCAAATATTCAACCGTGACGTCCATCTCGAAGAGTCCGACCAGTTAACTGCTGCCACCGATATAGTCCATGCCAGTGCAGGCTGTGCAGATACTGCCATTATGCCTATTCCATGCCAGGGCAGCATGATGGCTGAGCGCTGAGCGCTGATCGTCTACTCGTGGACCCAGCGTGGACCTTTCGGACGCAGAGCCACGAAGAGGCACGCCGCAAATTTCGCGCGGTAGCTGGCGCCTGAGCACCACTTTGGCTGGGGGCCTACTTATTCTGTATCTGTGTCATGAACTCGTCGGAGATTCCAATCTGTGTAAGTACCCCTTGTCTCGACGTCTTCAGTGCCTCGTGCCTCCGCAGCTCCAGATAGCTCCAAGTCTGTAAATATTCATCCACACCCTTCCGGCAAGCGATATCATTACGACAGGTAGGCACCCGAAACTATCATGACACGATGGTTCTGACAACTGATTTGGTGTATCGGATCATCACTTGAGACAATACAGAGAGGCGACATAGGACGATACTTGTCGTACCGACGTGTATTCTTCTAGCCCAAGTGACTTGAACGGCGAAGCGTCGTAGAAGATCGCAAAGTAGTTCGTAAGATTCAACACGTATAAGGCTCGCGCCGCTTGTCGACGATGACATGAACTCCCCTGGTGTGCATCATGCGCCTCAGAACGGCATCATTGGCATTGGCATGGCGTATGCGTTCTCGCATTGGTTTCTTCTCTTTGCGCTCGAATGTCTCGCTTAGCCCATCTGACTCTCCCCGTTGACTGTACTGCAGATGGACATCATCAAGTTTGCAATTATTGCATGCCTAGCAACGACTGCATGGTTTCTTTTCCGTTTCCTCACGCCAAGGCGGC

>TU1476-GaLu96scf_13-984222-984625 GATTTATTAGATGAGGTGCGTCGTGAAACAATGTGTAGGTAAAAGGCGGACCGAGATGAGACTCCGGGCGTGATCGGGTGCGATCCTGATTATCCCAATCTCCACTTTGTGGCCATTGTGTCGCGTACGGTTTGTAGAAATCGGGTGTAGGGTAGGGTCATGGAAGCCTTGACTCTAAGTCGAGGTTGGGGCTGGATGACGCTGGACCAAGGGTTCCACGACTGGTCACAGCATGGCGTCGACTAGTTACGCTACACGCTGCTGGGAGAGATGTCAGAAAGGACGGAACAGTTGTTAAGTGCATCGATATCCTTACTTGTAGACTCTCGAGTCTCTGGCTATTGTCTGTGGTTGAGTGGAAGGAAGGTGGGCGAGTCTAAACGGAGGAATGCGGGTCAGGGCAG

>TU1496-GaLu96scf_13-1052942-1054286 CGGCTCACAGCCCAACGACTCAACCACTGACCTCCCCAACACCATCTCCCCCCCCCTCGTCCCTTCTGCTCGATAGCCCTCGGTCTTTGACTCCTCAAAATCGTAGACGCGTGGAAGGTCCGCAAACAGAAATTGGACACTTGGAGAATACCTGAGTGAGAGGCATGCAGCCCCGTCCACCGACCATCTACGACGACATTATCCAACACCCCTCCCGGGCTCCTTCCATCATCCGCGCGTGACACCCCCAGTGTTTGAATACTTCAAGACTCGCGAATACCAGTTCAGAGGAGAAGAGCCGTGATAGTGGGGGATGTGGAGAGAGGGGGAGACGTATTCAATCCCGGTGCGCAATGTTGCCACCCCGCTCGGGCCACCATTCTTATGTCCTCAGGCTCCCCACTCTGCAAAGGACTTCACATGATCTCTCTCATCCAATACCATCCCTTCAATGACGTATCGGTGCCGAGCGGCTACCGTACTATTGTCTCCATATTGCTTCGGCCACACTGTGCACAACGACCGCAGCGCAGCGATCCCAAACGCTTGTCCACTCATTGTATCTTCGACGCTTTTCCATAAATTTCCGTTCCATATGCCCCGCAATTTGGAACCCGAACAAGCCCTTCCTAACAATTGGAACGTGGGCGCACTTAACCCCACCACCCCCAATCTTTCAACAACATCATCAACAACGCGGCAGCCTTCCTAATTTCTTTCCTTCGAACCAGTACGATACACCCGCACTCCTACACACCCGCCGCGCGTATGCCAACCGATATACAACGGTATACGGCGAGGTGAGGCTTAACTTCATCGCATCGTCGTAGACCGTCCTCATCGGAACCGTCGCCTCAACATTGTGCTTCCTCCGTCAGGCCATCGCGTTTACTGCTGCAATATGCATAGGACTCTGTAAGTCCACTCTGTTGGTGCCAACCTGCTTCGAGGCTGACAACTAAGTAAGACATCCTGCCATTTCCGTTCGAAGCGTAGATTGACTACTTTCTGCGGATAGCTTCAAGGAGTGCAAATATGTTTGCTATAACAAGCACTATCTCTCTTGTCACGTTCCATTAGATATATGCGGTGAGTACCGTCACCCAACGCTCTTGTTGTGCGACGATACTCATCGTTTTGATGCCCTCCATTTAGGCGCCCATATGCCGGTTGCCGCCGGCAACGACAAAGGAGAACAGGATGGGCATATTCCCTCACAAGCAGTGGAGGCATGGATCACAAAGATTACTGTACGTACCTCAGTACGTAGGATGAGGGTTAACCCATGCTTGACAAGTGGGCCCACAGATGGACACATATTTGCCGTTTCGTGGCTAGGACCCCT

>TU14-GaLu96scf_1-43786-44215 CCTCGTCTCCGCCTGGCGGTCGGTCACCGGTTCTCTCTCTCTCTCTCTCTCTCTCTCTCNNNNNNGTCCGTCCGTCCGTCCGTCGTCGTCGTCGTCTCTCTCTCTCTCTCTCTCTCTCTCTCTCTCTCTCTCTCTCTCTCTCTCTCTCTCTCTCTCTCTCTGCAGCAGTGACTCGGAGCTGACCGCCGTGCTTTCGTTTATTCAGACTCGTCCGACCCACATTCAACAAGCTCGTCCGGCTTCGCGTACCGCCACCGCCGGCCTGCCGCCCCTTCCCGCTCGCGATCGCGACCGTAATGTTGATGTTCAATTTTAATGTCCTTGCCCTCGATTCCGAGAGAAAGGCCCCTTACCCTTAGGCGTCTGGCTTGCCGCCGATCGCGTCGTACGTGGAAGCCCGGGGAAAGCAAGCGCGCCGGGATACAGATAC

>TU1501-GaLu96scf_13-1087975-1088560 GACTTTCTCGGGACAACAACGGGGTACGCGTAACCTTGCACATTCTCGACATATATAAGTACTCACAGCACATAACCGTAACGAGAATGGGGGCCCAAAGATCAGGGTTCCCGCACACGACGGATCGAGCGGCGCAGGCCGAGTATATAGAACGATGACTGTAGTGGCGCGACGGCAACGTGCACCACTATGTCGGCTATGAGTTGACACGGAGTTAGAGCCTGAGGCCTTCATCCGCACCTACACAGAGGACTGGAACGAGGGTGGCGACGCCATGCAAACGAGCTGCACAATATCCGGCCTTTGGATGACATACGGTGAGGACCGAGGGTCATACGCGCAGACAAGCACTCATAGGCGGGGGGCCATGTGAAGTGCTGGGGTGCAAACGAGCTGCACAGCGTCCAGCCTGCGGATGACATACGGTGAGGACCGAGGGTCATACACGCAAGCCAGTTGACGTTGAGCACTCATAGGCGGGGGCCATATGAAGTGCAGGAGTGCTCTCTGGGGCCGTCGGCGCTGCTTATATGCGCATGGGTCAGCCCGTGGGACGATAACTGCCGGTGAGACTCCCGCGAAGTCC

>TU1504-GaLu96scf_13-1095196-1095438 TCTCTCAAACACTTCCTACCAGGTTCCGGCCACGGAACTACCACTGTATGCTGCACGGACGACTACTCGTACGACCAATTACACACCAACACCAAACCCGCATGTCTGCACTGGATGTATGCGCGGGAAGTTGCGCACAATGCACAATTCGAACGCGTCACGGAGTCTTCATCCCGAAAATACCACGTCGTATCATGAAGACCCACGTCGGATGAGCATTAACCGGCCAAGCATTCCAGGTGG

>TU1505-GaLu96scf_13-1096030-1096645 CTCTTTTCCCACCAACGACGTTCGTGGATTGCTGGGCGACGGCCTCGGTTTGGAATCTATTGAGCTAGCTCCCAGCGCTCTCAGTTCGGCTTCCCAATAGTTCCCAGTATCGACACGCTTTCAACGCATGCTGCGCCGGCTTCTATCGCGGGAGCTCCGAGGTCTGTCGCGCATCGGCCTACTCACTCGATGCCATTGCTCGAGTGAAGCGATCACGTATGGTAAGCCACTTCCTCCCCTCATTATCACCTCCATTCTTTTTACGTATGGAACAATCGGGCGCGTACATGGCTATCTCTAGCTAATCCCCGACGGCAATATGCCTATCTCCCGCGTTCATGCCCAAAGCAATGGCTAGTTCTAATGCCGCACCTCGCCTCGACATCCCCGTTCGAACACTCCCCGCGCGTTCTACCCGGAGAAGATCACGACGCCGGCACGCCGGACGAGGCGACCATCGATGAACACGCGCGCGACGAAGGTGGAAGGGAGAAAATAGCCGTGGATGATCCAGGCCTTGTCCCTTCTCCCCGCGTCGACTGCCATTTGATATTAGGGACGATCCTCTTCGAATCCACCGCGGGGCCCTCTCGGACCCCTCCTACGACGCCCGTGG

>TU1513-GaLu96scf_13-1126492-1127264 CCGAGCGACGATGCGCGACTCCCCTCCCGCCCCTTGCCCTTGTTCTTCCTCCACTCAGCGAATACAGTTTCACCGGTGCCGCACTGCACGAAGGCTGACGACTCATTCGTGGGCAAACTGACCTCCCTGGCCGGGGGCCGATCACCTTTCGACGACGCCCACATTCATTTCATAAGTATAGCATCTCCGGAACCACCTTCGCCTTCTCTTACGCCCCCTCTGCCGGTCAGACCTGTTCTCGTGGAGTCGTTTCCTCACTACGGACACTTCCCGAACCGAGTCGCGTTCCTCCCATCGCCCCTAGAACTTAGGACGTGGCCTAAGTTCAGCAGCGCGCATTCCCGACCCTGCGTGCTACTCGGAGCCGGTCAGGCCTCCTGGGATCGCAACGATCTCGAAACGACAGTCGCGCGTCGGACTTTGTCTGGTCTCCGGGACGCGCCGCCTGCCCCTTTGCCTTCCCGCGTCCTTTTGCACGCATCACCCTAGGTCCGCGCGCGGATCGCCTGTGGGTGCTCTCCCATGGTGCAGCGCGCTGGCCTATGGGGCGGAATCGCGCCGCGCTCCATCAGGTGTCGGTGCCTGACGTCAGCGCGTTGCCGCGATGCGGTCGGCGAATGCTGGGGAGACAGGTTGGTATCGGTATAGAACCACAGAGAACCACACGCAACGCTGACGCCACAAAGTCGAAGACGGTGGTCTGCCTCCCAAAGCGGCTAACCGTGGGGTTCCGCTTAGGTCAAGGAGCTCCCGTGCGTGCCTCTGGTCGGACT

>TU1543-GaLu96scf_14-102014-102523 AAAATTGGTAGAAGGATATAGGCATACAGAAGATTGGACACACAAAATTCTTTGAAATAGGCACATACGCATCGGCCCATTAAGCCTCTCCATCTCTTTCTTTCAACCCCTCCAGCAGTCCCTCTAGGCCACCTACGTCGCCGACCGCTGCAAGACCGTTATGCCGATCCACAGCGATAGGCCATTTGAGCGCATTGGATGGCAGGGCACGACACCGTCTAGATCCTGGGGTCAGCTGTCGATGGTAGAAGTGCGAGGACAGGATGTTCCCAGCGGTGGGCTAATCGGCGATACCGCTTAAACCGGTGGGTGACTGAGGTAACTGAGGAAGGGGGAGCAAATGTCAGTACGCACAAGATAGAGATCTCAGACTGACAGGCATACCTGGAATGGTCACATAACATTTTGAAAGATCATGATTGACTTACGATTTCCCGCGTGAAACCCTTGAACGTAGCTCAGTCAGGATGGTGAAGCGCAGGGAGGGTAGTGGCCGATGGTGGTGGTCGG

>TU1547-GaLu96scf_14-110922-111227 AAGCCTCAATAGTACGAAAGCCTTCAAATTGGTGCCGAATGTTGATATATGTCATCAGTCTTGGACCATTTCATGATGCATATCTACTTACCGATGTAGTGAGGCACAAGGGGGCACCGGTGCCAACGCCGCATCCCTCCAGTGTGGGCGCATGTGAGAACCACCGTGACCAAACCGTGACGTTGCAAGGAGGCCAGCGGCTCGCCCGAGGGTCGCCGACGTGTTGTGGGAACCGTCTCGCAACCGTACCGCTGACTCCGAGCGCTGAGTTGAACCAGTTCGCTGCCGATGTATTCGAAGGAAGAG

>TU1548-GaLu96scf_14-111582-112177 GCGTCGATGGTTCCTGGGGACGGTCCACATCCGCGGGCATTCAAATATTGGCTCAAATATGTGATTATTCGCACTTCAAGGCGAGCAAGCTCCTGGGAGATAAGAGAGCGGCAGTTCAAGTGGGGAGCAGTGACGCCAGCAAGGACATCTCGAGCATACGGCGCGTCTGCGTACTAAGTGGGCGCACTCCATGATGACCCTTCGCTGACCCTCTCAAAGCTCACCGGGTGTTGCAATGCAAGCCGACGTGGAAGCATTGTAGATGATCAGATGCTGGGTCGGATCTCGTGAGTGCGCTTTGTGGACGCCGTGGCGAGGCGGTGCGCCGATACTCACTGTGGTACCAATTCTATTCTTTTCGTGGTGATGGATACCTCAAACCATCAAGAGCGAACGCGTGGTCGCTCACGAAATCCACACAGATCGTCTTGGCCGAGCTACTTACGGCCTTTATGTTCGAGGCGTGGCGGAAATTTCTCGAGCATTACGTTTCCGAACGCGAGCTATGGAAGCAGAAAGCCGGAGCTGTAGCTGAGAGTGTAATCAGACCGGACGCCCAACGAGTCCACAACAGTCTCCGCCGAGGCAGTTAATAA

>TU1551-GaLu96scf_14-116661-116985 GTTACGTCCCCGAGACAACTTCCTAGTGTGAGGGCGAAGACGTAACCGACCATATAGCGGCGTTCAAAGCTTCAATTCCGGCCCCACTCGGGACACTTGATTATCGGATGCGTTCTGTGGAACCCCCAGCACAACATCGGCTTCGACCATGCTTATGGACTTTCTTGCACCTTCTGGATCCTATATCATGCTGATGTTAGACAGGAGGGGAGGCAAGACATGGCGATATCGGCTCACCCGCGAGAAAGCGAATGACTCCCGTGACCCTCCGGGGAACGCATTTCGCCGACAAGGAACGCCATGGCGTGTGAGCCACCACGTTGAC

>TU1555-GaLu96scf_14-137566-137933 TCCTACTTCCTCTCGACCACCTTCCTCGCACTTACGTCCTAATCTTTCCCCCTTTGCTGTCCTCCATTTTTCAACCCCTACGCGAACGCCTCTCCTGATTCCAGCAATGACCTCTCACTGGAAGCTATGCTCATGGTTCTGTCGTGTCTCGTGTCTCTACCAGGCCAACTCGTCCTACTCCTCCACTCGACGTCACGGTCGATGATCGCCCGCGCCGGACATGCCTTCTGTCTCGTTTCTTCTCCCTTGGCACCCGGAGGTGTTGAACCAGTCAATACGGCGATCCATGACGTGACTACTCCCCGCTCGCCTGAGCACGTCGTACCGGGCGCGAAGGATGTCAACCCCCTGGGGGTCATCGCGACATT

>TU1562-GaLu96scf_14-153084-153929 GTTCCTTCACTTCGAGCCACCAGCGCGACCATCCCAACACCGCCCCACACCGCGAGTTTCCCCCCCGGACCATGTCTTATTGGATGCAGCAACCTCGCGGTACGGACTTTCCACCTACTTCACTCGGCACCACCAACAACCTTTGTCAGGACCGCGCACTTGAATTGTATCCCGGCGCGCTGGGGAGCATGTGGTACCAGTAAGTTTTACTCTCTTCTCCCGATGTGGACGATCTGAATGGATGGTCTAGGGCCGTATATTCGTATTTTCAACTATGAAACTGTTGACATATTAGCAGGTCATCGGATATAGGCAGGCCCGGGCACACTGGTTCGTAGTGATATCAATCGTATCCCCGACTCATAATGCTTTCCTGATCTGCTAGTGTAGACATGCAGTTATACGCCTCTGGCCTCGAGATCTGGTGAGTTATCCAACTATATGACTGTCTTATGCATACCAGATACATAACCGTCCCCAATTCAGGTCTAGACGTTGCAGGTGGTGGTAACCAGTGTCTCGAAAATCCTCTGTGTGAGTACAATATTCAGTGGCCTCCTACTGGGAAAGTAGCCCATATTACTCAACCATTTGCAGATTACACATGCTGATCAAGTAGTCTAGCTGTCGCCGTCACTGTCTACGCTAAGCAGGGGAATCCCGCCTCCGGGATGTTTGTATGTCTCGTACATTAGATACATGTGGGCGTATGTAGCGCCTGAGGCCTGATTCGCAAAATATATTTTATTGTTTCTATGGCCAATGGGAAGTGGGATCGGAAAGCGCTGACCTCGTGAAGCACACTCGAATGTCCTCGATCTCGCACGATTCCACTCGCAGGGCCCC

>TU1563-GaLu96scf_14-155349-156446 GGTGTGAAAGTTTCCGGGTGGACAGGCGGAAGCTCGCGTTTGGCCTGAGAAGCGGGGGGCGAAGATGACGGTTTCTCGTAGGCAAACGCGTACCGTACACTGTGTATCTACGAGCAATATATACCAATCATTATCGATGGTGGCGCGGGGACGAGCGGAACTGATCACGTAAGCCAAGATGCAACAACACACCCCCTTGAGCAGTGGAGCGAGGACGAGGAGGAGAAGGAGACGGACAAAGGAAGCGAAGACAGCAATGGGGAAACACCCCCTCAAACGAGTGGCGACAAGCAACAGCAAGAAAACCTCAACATCAACAAAACCTATGACCCAAGACCATCCAACATCCGGCGGGCAGGCACCGCAATAAGCCTTGAGGAACCACGCATTCGAGCTCCAGGCAGGCGCGCTAGCGCGTGCGCGGCGGGACTGATGTCCGGGGTTGCCCTCGGCGGGCGGAACGACAACGAGCGGCGGCACACCCTCGGCGGGCGGCGGCAGCAAGACAACGGCGACAAGCGCGGGTTCGATCGGGTTCGCGCGGGGTTCAATCGGGGGGTTCGATCGCGGGAGGAACGGCGGGAGGGACGAGCGACGAGCGACAGCGGACGGCAACGGGTGCGAGGAAGGCGACGGGTGCGGGGAGGGCGACAGCGAGCGAGCGGGGACGTACGATGGGCGGGCGCTTAGACGAGCATCAGCGCGAGCGGGCGACTATGGCGGCAGCGCGGTGGTAGGGCTCGGGAGTGCCGTGGGAGGAGGGAGGATCGCGGTGGGATGGGTGTCGCATGGGGGGATTCATCGGGAGCGACGGCGGCGATGGCGGCGGCGGACTGCACCGATCCGGCAGTGGTGACTTCGGCCTCACCAGGCAGTGCCTCGAATTATCCTTCGGGCGGAGGAGACACGTTTGTGTGAAAGTTTCCGGGTGGACAGGCGGAAGCTCGCGTTTGGCCTGAGAAGCGGGGGGTGAAGATGACGGTTTCTCGTAGGCAAACGCGTACCGTACACTGTGTATCTACGAGCAATATATACCAATCATTATCGATGGTGGCGCGGGGATGAGTGGAACTGATCACGTAAGCCAAGATGCAACAA

>TU1567-GaLu96scf_14-164119-164805 ATAAAATATACAGATGCGTGTTGCTGACAATACAAGGCACGGCAAATACAAAATAAGAATACGAAGGAAAAGATGGAACAGAGAAGAAGCTCCATCCAATGGCGGTAACGACTGTTGATGAAGGGTCGGGTCAGAAAATCGAACGAGAGATCATCGAAGTACCAAACCTCGATTCCGGGGTCGTAGTGAACATAGCGGCAAGCTACACCTAGTGCTGATGGCGTAACACCGGTAAGCTGGTATCCGGAATGCAAAAGTGTGCAGTGCTCACCGTCTTCAGGCAACGATACACCAAAAGTGCGATGACGACGGTTGGTGCTCGCTGTCAACGGGCACCGGTAGGTCGATGACCAGGGACGGATCTTCGGTATCTAGTGGCGATGATGACACAGCGACCTCCGCCTCGGGTTCGAGGAACAGGTCGTGCGTGAGTTCAAAGAGGTCCATGGAGTAGAGCAGTGAGAACAGCGTGGTGATAAGGCGGCGTCTGAGCAGCAGACTGTTAGGGGAGGCGCACTTGAGTTGGTTCGACACGCGACAGACACTGAGACGGACAATTATATACCATCGAGAACGGTGGGGCTCGGGAGGCGCCCTGTGGTTCGATGGACTCCCCGGACTTCGGGGCTCCAGAGAGCACCTGAGCGATGCGAACAAAAGCACAGCAGAATGAACATAGAGCCTCGA

>TU1570-GaLu96scf_14-171993-173772 AGATATCTTCAGTACGTTCAATTGCTCGGATTATTGTCGTGATTGATGTATGTATCTCTATTGCCAGTGTGGAATCGTGCCGCCCAGTGTGGTTTCCCTACTGAAGAATCCTCTTGGAGCGGAAGTTGATGCGGGCGTACCCTCTCCAGTGAGTCTACCTCTATAATCCTCGTGGTCATCTCGGCATCAAGTCTCCTTTCTACCGTTGGATGTCCGTGGTGCGGCTTGTGTCGAGGCAATATTATGTGAGAATTTCCTGGTCGTATAGGTGCGTCTTTTGAGTCGATCCACGGTACTGGCTGTCTACTTAACGGTACATTTTGACAGCCTGTTACCGGTGCCCCAGTGGATTACTATTTTCGCATCGCCTTCTGCTCGGTGAGTCGTTCCGTCTGTTGATTCTTTTGTGGCCTTGCATAGGGTCGACGGCACCAGCTGTCACTCGTACGGCACGCGTCTCATGCTACGCCTCGCGTTGCTAGGTTGCGGACTCGCGGCTGTACTACGGTTGTGGTGCCTCCATTTCTCTCTTTTTCAGCATATGTTTTTCGCCGCGAATACTTCCAAATAAGGCATCGCTCTGGAAAGGGAAACTCTACCGCTGCACACTTCTCCTTGCTATGCTTGTGGTCGCACACACCGATTTAAGGCCTCCGACATGCAAACTCGCAAACCATCGAACCTTGCGCCCTCTCTTCTCGCTGGATCAAGCTGTGGACTCTCGCCCTCTGGCCTGTTCATCCCGGATACCAATTCCTCGTTTACCGACCGACCAGTCCTCCCCGCGTACCGATCCATCTTCGCGACCAAGCCATCTTCCACCCGTCTAAGCTATCTTCCCCGTGTCCCGAGTCATTCTCCTCGCGTACTCTTTGTTCCCCACCGCTTTCCCCACCTCTGCCTCCCTCGCCCTCGCCCCTTACGTGTGTAGAAGCTGTGGTCAAGTACATGTACCTAATATATTCCCTCCGGTGCAACCCCTCCCAACCACATTCACATCGAGCTCCTCAACGACCAAGAGCTCGACCGCTGGTGCAATTCTAATGACGAAGATCACGGCAGCAACCACAGCCGTGTTTCAAGCATTGTGGTCGACGGGGCCTTTCTGGACTCCCTTCTGGACAGCGACTCAAGATTTCCGGCGGTGGTTGCGTTCGAGGTTCGTGGTGAGGTACCCGGGCCACCCTCAAGCAACTGCACGAACTGGCCTCCCTCGCCGTGGGCTGGGTTGCCAACACTCGCTGGGTCAAACGACGCGCTCTGCCACATCCGTACCCGCAAGCTACTTCAGGTCCGCAAAATGGTCGTGAGGCTTGCGCTCAACGCATCTGTGTGCGAAGCACGGACCGCATTTCGTCGCAATGTGGCGGCTTTTCAACCAGACACGCTCATGGCTCACCTCGCGTGCACGGAATGGGCTGAGTGGCACGCCCTCGAAAGTCTTCTCGTACGTCTGTCGCACTTCCTGGGCCTCCGTCAGAACTGACCTCATGGGGCGACACGTTCCTTTAACAGGTTGCGCTCTTTGCTGCCGATTCTCTTATAGGTGTCCGCCGGCTCTACCTCACGCTTTCCCTGCCGGCGAGTACCAGGGACGTCTCAGGTGAGTGCGGAACCACTGGCTTGGGAACTGACCAATTTGCTCTCCAGGACTTGATACACCGGGCGATCCTTCCGTTGACATGCGCATCGACGATTCGGAGCTGCGATTCGCTCGAGCTTGTCCATGTGAAGGCTGTTACGGAAGCCGATACTCTATGTGCATATTACTGGTTGCGTA

>TU1602-GaLu96scf_14-429346-430653 TCTGAAACTCGGAGTTCTATATTCGGAAAAATGTTGTACAAAACGTGACATTCTACATTCCCATTATTAATCCTGTTTTGCCCCGCTCAGAACACTGTAATTCCCCGCGTGTCAACGCCACTGGCTCTGGACGACGACCGCGGGAAGCGGCGCGGAAGGGATCTCCGTGGCGCCGTTCAAGGTAAGTTACATGCTTTCTCACGGAAGCCCAGGGGCCAGCGGCACCCTGCATCTGGCAGTGGTCTGCCCGTCATCGTGCCCCTGCCAGTACGACGGTCTTCGTACAACTGCGGATTGCGGTCGTTGCAACAGTCCACCCGAGCAACTGTCTTCATCCTCAGGCTGCGCCGCCTCCGCTCGATCGTGGACGCCGGCACGTACGGCAACGGCAACGATGCTTTATCTGTCTGTCAACGGTGTGGCGTGTGCATGAGCACCGTTGCTTCCTAGTGAGAAGCAGCGGCGATCATGCAATGGATGGCATGTTCCTATATCCCGTCGATGAGCTCGAGGTTATCACGTTCTTCCTCCCAGCCTGCCTTCAGCTCGTCGCTCGACACCGTGTATGCCTGACGGCGATGTTGCGGTCGTTGGACGAATGAAACCTGCGTCCCGGAGGAGTTTGCGGCCTGGCCGCCGGGGATTGGTCCGGATCCACCATGGTGGTTCCGCTTCCATCAACAACATACCGCTGCTTACCGCTTTATCCTGGCTTTCGCATCTGCAACCACGAGCAGTCTTCGACTCAACGATAATCGCCTTCCAGCCTTTCAGATGATGCTCGAATGGATCAGTAAACTGCTATCGAGCCTCGGGGATGCATCGACAGAACAGAATCTAACCGGAAGATATAGTCTGACTACGTCGACCCGACCCCCAAGTTCCTGCGGTACAGATGACCTTCGACAACTGCAACACTCCTTTGGCCGAGCCATACAAGTATGTGTCAGTCATTTTTTCGATATCCAAACGACCACTGAAAATGTCTGATGGACTTCGCAGGACGTCATTGAGTCGAAGGGACACTAGTGCTGCCAGTGCATCAACGACTCGAAGGGTGGTATATCAGCTTCGGTGAGTAGCATTTCAACCTATAATTCGGATATGAAGCGCGAGGGAACCCCATTCTCTTCTTGAAGAATGATAGGTTATCTTCCGCTATAACGCAATTATTGGACTGCGGACGCGTACCGCTTGCGGAAGTGGCTCAAGGCGGGGACGACATGAATATTGTGATTGCCTTAGTCTATTTTATTATTCACACTACTACAGTATTTACACGTTACTGATACAATGCATCCTCCTCCT

>TU1616-GaLu96scf_14-565230-566392 AGTTAAAATTAATACTCAAACGGTCGCCTTTCAGATCGAATACAGATATGAGTGTGTGGGAGCTGGCGGCCAACCGGGCAACGAGGTCACACAGAAGAGGGTTTCTTCATAGAGCGCTAGGATGGGGCAGTAGGCTAGTCTACTGGTCGTGCGCGGATTGCCGGCCCATGACGTTCTGGGGCTGGAAGACATTCATCGCTAAATCGCAACCAGATACAGATAGAACTCGAACTCACCGTGATCTGAAGCTGGAGATCGGAGGCACCGGTTCCAGAGGCCGACAATAGGGGTCATCCGTGTCGATTAGTCTCCTGTATGGAAGGGACTCGTCTGCTGAGATCTTCGCCTCAACAAGAATACGTACGCGCTCACCTTGTGTCTGCAACAGAATAAGGGGAAAACACTCACCAAAGCCGTTGACAAGGACAGCGACCCACCACTTGGGAACAACCAGTCGTTGGAGGCAAGGAGGACGGGCACGATGTTATGAATCGGCTAGTTTTGTGTGGAGGTCATAGACGAGGAGAGAAATTGTCCGCCCGCATCGTCCCCCGATCGCAAAAAACCTGGAGGATGGAAGGCACGTTGGCTGTGTATGTTCCTACCGACACCGTGGATACCTAATTTTTCAGTTGCGGAGAGGTCCCGATACCGGAAAAGAGCTGGGACGAATGAATTAGCCCCTGCTGACAAGACGAAACAATGGTTAAAGTGTCCTAACGAGACGCGTTGACGAGTTGACGTCGTATGAGGAGTTCCTCTGCCTGCTCCTATCTCTCAGTATCTAACCTGCGGTCGGTGACGACAGTACTAAACAACATTGAGGAAAGTGTACACCTCTCCATAGACTCAGCGACACGTATACGGTCATCTGGCGAGTTGCGGGAGTTGTTAACTTTCTTGAAGATCTGCGGCAGCCGTCCACTGGCCTGGGCACTGCTGAAAACCTACTAGCGGCTCAGTAAGCGGATTTTGTGTTGGCCGAACCACTGGCACGGCAGTCATTAATGAACTAGAAGATGGGCATATATGATAGGAATATGAGCTGAAAAAGCTTTTAACACGAACGCTGGGTAAATACCTGAGACTCCTTGAGCGCCGACAGCCTGGGCCTCCTGGCCCGTCACGTCCAAAACGTAGTCTTCAAGCAGATCTGCGGCGAC

>TU1646-GaLu96scf_14-724083-725000 AGCGCGACGTTGACCGTATCGTTCGCATGAGTCTCTCGACCTTTTCGAACTTGAGGTCGAACTTGAAGTCTTCGAGGCCACTCTCCGAGACCATCCTGACACGTTTCAAACTGCATTCCACCATCGTCAGCCCTACGACATCTGCACTGTGAGGTCTCCCCTCAACCTGCACTGGCCCAGCGTCCGACGACTTCAGCAACGCGGACGTCGACTCTGAGTCTTTGTTCGAGTCCTTCATTCCCTACACACCCAATTCTGTCGTCGCTCAACCGTTCCCATGCCGTCCCTGCAGCGGGCGACTTCTAGGTCCATCCCCATGCACCTGTGACCTAGTGGATTGTACCTAGGCGTGAAAACATGCCGCACCATCATCGTAACCTCCACCACGTAAGTCAGCCTTGTCGGCTTTCTTCGCTCCTCCCTCCTTCATTTCCACTCGCAACGGCACATACCGACTCGCTCGACGACGGCATATCTCCGTCGTCGACTCCCACCCATGATATCGTTCCCCACCACATACTTACATGTGTTCGTGCCTCTCTCTTCGCCTCCCCTGTGGAAAACTTTGGTCCTTTAACCCCGCCTTCACCCACTCACACCCATACGGTCATATAACATGACTACCGTAATGTATGCTTCAATCTTCCGTTTGTACTACGACGACGACATGTTTGCCTACACTCTCCAGCAGTAATCCCGCCTGAATCGTTTTTGCTCTACATCATGCACCCTCTCTACATCTTCCTCCTCTACCCCTCGTGGATCGCTTTCAACCTCATCGTTTCAATGTACGGCATATAATTTGTCACATTGTGTACGATTAATGCGCTGTCTTGCGTAGCGGGCATCGGCATACCGTCGCCGGCAACCTTGTTACTGCTGCGAAGCCGTATAACCGACATGAGCATGTTGTTAACA

>TU1651-GaLu96scf_14-758039-758294 GTTGACGCAGGCCATTGGCCCGGTAGCATTCGGTTTGGTTCTGTTTGGTTCTTTCCCGTTTAGTTCCCAACGCCCGAGAAATCCCTTGTATTTCCATTCAACCCCAGCTCTCCTAATGCCTCCCACCTCATCCGTAGTCTCCGTAGTCGTACTGTATGATATACTGATTCGTGTATGAGTTCACCGCAGCCTAGAGCCCCGTTGGAGCTCTGACGAGATGACTCAAGCCTACTTGTATATTAGAAGTGCCTCCCCA

>TU1670-GaLu96scf_14-898033-899439 CAAGTCTCCCGTCATGCTCAATTCTATTCCTCGGTTCGGAGCTTGCGTCGAATCGATGCGAATTGCTCAAGCTTAATGCTTACACTCTGAAAATTAGAGAGCTAAAGCCGAGGGAGTAACGGTGCGTCCTCGTCATTGTCGTCGACGCTCACCGGGTCAAACCGTGGCTAATCAACTCATTATATTGATTCTCGCACCCCAGAGAAGCCGATACCTCACGTATTGACCCCTTCGACTCCCTACATGCATTTGTACCATTCACCCGGGCGCGCGGTGTTGTCGATGTTGCAAAAACTACGTCGCAGGGTGCGTACTTGGGTCATTTTGTCAAATGCAACGGAGCCTTGGAGTCGAAGCTCAGCTCCCCCATGGGATCTTCAACCCCTGTTGAAGGCCCTGGGCGCGTGCAAAAACATGGTGTCCGTTGAACCTTTGAAGTGCTAGGAAAGCAAAGCTGTCCATATTCGGATGGATCTCGAGATGGGAGACGATTCCGGCACTACAATAGCCGTTTTGGGCTGTGCAATAAGGCGAGGTGCCCGAATATTTCAAGGGTGCGTAACCTTACTTTTTTTGAATGATCCGCTGATGAGATTCTAGGCCCGGTTCATACAGTGCGATGGGGTATCTCGAAGACCGATGCAGCCAGTACACTTCGTGAGGCGAGATGTTCCCATATTGGTAGAGGCCTTGGGGTTCGATGTATGACATCCGGAATGGTGAACGCAGTCCACCTAGCGCACCCACTCGTCCGAACTTCGCTCGTCGAGTGGCGATTTCCATCATTGTAGGTGTTGTTACTGGTACCATCGCGCAGCCTAAGCGGTTGGAATCCATTTTCCTGTCATGATCTCTCTCTATCTGGGATCTTGCCGTCGCAATCCTTTCCTGGGCCGAATATGACCGCTCTCATTGGGCACGCAGGTCAGCTACTCGAACACATCTTCCGCGTCTGCATACAATGACTAGGTCTTAATGTAGGGGGAAGCATGACAAATAGTAGATGCGTCCATACTTGCGCTACCTGAGCGGACCATTGACCTGCGCAGATGCACCGGGCACCACCAATCACTCCACAAGCGGTGAATGTAAGGTTTCAACGCTCCTCACTTCGCGCCCAACTGTGTTCTGTCATCTCTCGCGCACAGGCACAAGCGCAGCGATACGTAGGCTGCACGATGCAGAAGTTGCGATTTACTGCCCTTCTGGCATCTCTCTAAGTACAGGTGAAAAGGTAAGAGTCGAAATCCTTTTTATCGCGAATGTCGAGACTCAAACTTAACGATGATGCAGGTTTGCGACCAACGGCACCGTGCGCCTGTGGGGATGGCTCTGTAGCTTGCGGTCAGATGGAGACAATCACGCATGTCAGATGCTATGCGTACTATCCTGGGAGGTATATTAATA

>TU1680-GaLu96scf_14-975848-976418 GACGCGTAGCGTTCGAAGTTTTCCTTCTGTCCTTTGCGTACAAGATGCTACGGTTTCTGTAGGTGAGTGAACCCTCCAAGCGAGCCCTCCAGTGATGATACACACTTCATACCATATAACTACACCGAAACTACATGTAGACAACAACTTTATCTAGTTTCAGCCTTGGCTGACCTTTACTTAGCAATGTTGTTTGAAACATGTCGCTGATCAGTCCTTTCATCTAGCATATCCCTGTCTCCTTGCTGGATGTTTCCCGGACGTGGCTCGGTCTACAGTCGGTGAGTATTTTGTTTTGCATGAATCTACCCGGATGAAATATTTTCGATGGGCGGACACGAATGCTCTCGGGATCATACCAAACTCTCCCAGTGTACAAAGTGGAGGGGGTCCCGAGAACTTGTTGATATGCATCGAGTACCTGACCTGCGTCCCACTTTGAGTCTCAGATCCCAACTGACCATCCTCTTTCTCAGATCTGTGTGATTCTTTTGGTGTTTTCCACTGTCATGTTTCTCCAACGCTTGATTCCTGTATCCATGGTCCGAAGTGTAATGAATACCTATTCCCA

>TU1690-GaLu96scf_14-1079787-1080094 CAACAAATATATTGATGGAACTGAGCTAAACAATAACAGTGAGGGTTGTAAGGCACCTCGTAAGGTCGTCAGTTCGTGTGAGTGCGAACAACGGCCGAAATAAATGCCTAGTCAACAGAAGTCGATCTTCCCTTCATTAGCAACTCCCCACCTGATGGAATGCACAGAGGACTGAGCAGGTCAAGCTGGGAGTGAATAGCAAGGGCGTGTCGGAGTAGAAGACTGTGGCCATCGTAGAGACAAACCAGACGCGTTTGAAAAAAGCATTCCTCAAGACTCATGAATTGGAGGACGAGCACTGGACCAGG

>TU1740-GaLu96scf_15-224375-225121 GCTCACTGCGCTACTCAGTATTCAATATTCAATATTCAGCGCTCAGCACTCTGCCACTACTGCTGGCAGAGTAAGGAATTCCAGCGGCCACCCCCCCATTCAAAGATGTACTACTGCGTTTCAAGTTGCGAATTGCGCTATGGCCGTGGGTCACTTTTTCTCTCGTGGGTTGTCAGAGTTTAACGTTTAACAATGGTACTTAGAGTCGGCCGACCATGCCTTGAACGCCAGTAGTGCGATTCTTGAACTAGTACCTGAAACCCATATCAAAGCTAAGTTCGTGATAGCATCTGACAGGCTTAGCCCCCGGAGCACCTTATCAGTTCCCCGCCGAGTTTCAGCATCTGCTTGTCAATGGCAGGTGCACAGTCAAGGCCAGAAGAGATGGGCTCCAAGAGAGGCGCGAATGGCGGGGAACATGGCGACCACGGCTCCACTGGGTATTCACGTTACCAGACATAGCGTATGTGGCGAGTCGTCTTCACATGGGCAGAGGTGAGTTGACAGACCGCAGCTGTTTTCCAGGCCGTGCGCCAGAGTTGGGACGCAGTTATGTAGAAGCCAGCAAGTAGTAAGTTGCAGCCCGTTTGCTATCCTCCCTTACTGATTGCGATGTCTATCGATAATTCCACACTCCCCTCCCTCCTGGGACAGATCGAGGTCAACGCGCTTGCACTGAGCATCATAAACGATTTCGCCGTCGGTCATACAGCAATGCTGGTCGAGCTATTCCTTTCAAGCCTGTTG

>TU1767-GaLu96scf_15-314835-315740 TTACGTTCACCCGAGGTCGGCAACTTGCGCTTCAAGATCAGCCCCGATTGCGTTCTTCAAAGCTTCGAGTCCGGGTCCAGGGAGGGAGCGCGAGAAGATGCAGGGAAGCCCCCCACAGGCTCAGGCGTCTGACCACACGAAGGTTCCGCAGGCCGCGCTCCGCCCTAAAGGCTGCGCACATCGGTAGACATCGGTGGCGGCGGAGCGTCGCGTTTAACGCGACCGCGCGCGTCGGGTGGACGGGCCAACCAGCGACGCGTGCGCCGAACAGTCGAATGGACCCATTGCGGTCGTCCAGTGCACGGCGCACGCATGGCGGACGCCGCGCTGTTCGGAGTCGGGAGTGCGAATAGAGCGGAAAATCCCCGACGCCTCCGAGCACAGAGAAAGGAATCGGCGTTCCAGCTTCCACCAGCGCAGAGCGCTCGCTGTCGGATGAGGCCAGAGGAGCCTAAATCTAAGCTCGCCGCCCCCCCATTGGATGGCCGTGCGTGCGCCGGGTCATGAGAGGTGCGCTTCTGCTTTGTGGCGAGCGTGCCGTAAGTGCACCGAATCCTGCGCCCATGTAACGTTATGACAAACGTCAAAGGAGCGGCTGTGAAAACACTTCAGCTCCTGCGCCAGAAGCGGGGCGACGAGGTGGGGCGTTCTGATAGATCGTGGCCGCCATCGATATTACTGACGTTCACGTTATCGACAGGGCCTGGGTGGGGCAACGAACTATTCCATGTACCAGACTACGTGTTCGAACAATAGTTCAGAAGTAAAGAAGCTGATTGTCCATCGGTGCGACGCCGCGGACCTAGCTCCGGGTACCGTCTGGGCTCCGACCGTTGGCACGAAAGATGGCCACCGCATGGACGAGTTCATGTCAGTACTCAACGCAACCTTGAGGCCATTCATCGC

>TU177-GaLu96scf_1-1272656-1273835 CCTGTGTTCCGGGAATAAACGGAACCGGCCAGTCCTGCGGATGCAGAGAAGCCCAGCTCAGAAGGTGGTGGTCATCACAAGTGATTTGAATGAAGACCACATACGAATGACGATCCGCTTCCTGCCGGGATCACGAAAGGCACAATAAACGCACTTTGTGTTGCCCCGATGTCAGATCAGAAATATATGTTGTATGCGTGGCAAACAGTCGAATTCACTCACGATGTGTACACCTGGAGATAGTATGGGGCTTCCGGATTTGGTCTGAGGCGTCCGAAAAATATTCGTCAAGCAGAGAAAGCAACGAAAAAGAAGGGCGGAATGATGTACCCAGTGGCAGATCGGCGACCCCATGTTATGTGGAGCGTATCACATTGGGCTGCGGGTGGACGGAGATGGCGGGTAAGGTTTGAGCGACAGAAGGTGGAGAAGGTAATGGACGTACCAGCAGTGGGGATTGGTGTGCTCTCATCTCCGGCCCAGTCGAAGTAGAAACCCTATATTAGAAGACAGACGAGCGCGAAGGGTCAGTCCTGGCAATGGCTGAGGAGAAGTCGAGGCTTGTGGGGACACAGCGGAATGCTCTTACCTGCTGCATCTCACTAAAAATCTCCGCCGACGCACTCCGGAACAGACTATGGGTCCATAGCAAGCCGAGTGTTAGGGCAGAGACGAAAGCATGTGGTCGACAAGTCCGGGCGGGCATGGACGATTGGCTGGACTGGGATGCGGGCTGGCGAGGAAGTCCGAGATTCCAGCACGGCTGTGGGTGGCCGAGAAGACGAGAACCAAAGGCGAGGCGAAATCAGAGAAGCAAGGAGTGACTAGCAGGGGGGCGAGACTAGGAGGGAGTCAAGGGCCTTGCGGGCGGGAGAGCAGGAGGAGACGCGACCCAAGCCAAAGGCGGTAAAGATTGAGGAAGCTGAGCGTCTGGACGCCCTCCGTGGTTGGACACGGAAAGCGTGTTCGCGTCAATACCTGACTGGCTGTGGTTCCAGGCCAGGCCCCCCCAGCCGATGACATCTTCAAGTCGCATTCTCCCCACCGAGATCCTTTCGCGCAAGATATTCTTCTAAGTATGTGTACACGAAACCCGATTTTGAGCCCGACCCCGAGACATCCACGCCTCAATTGACAATTGAGAGACGTCGACGGGAGCGGCGGTTGCGGCGGAGGGCAT

>TU1789-GaLu96scf_15-432408-432811 ACTACAATGAATACGATCTCATATTTCCTTCTGAAAGCGTCCGATCATCGTGTATGTTCCATTTGGAGGGGCGATGCGCGGAAAAATATGGCTGGGCTTGGCTGGGCACGAATTTAGTAATAATAGTACGGGGTGTGCGGCTGAGACACCGCTGGATGGTTTTGAACATGTCCCTCTCCGCCGAAAATCACGATAGAGGCGTTAGCTTCTGTAATCCGAGCTGCTGCTCGTGGCAGGCTTGATGCAGAGCGGAGGTTAGAGGATCAACTTGAAAGGGACCTTGTGAGCGATATCAAGGACTAGCAGTTTGAATCCACGACAGACCGTGCCCAATAACTCACATTTGTCAGTAAAAACATGCTCGCGACTCATAGAAAAGTCAAAAGGCTGATACACCGAGCCTG

>TU1802-GaLu96scf_15-473609-473972 CGGACTCCCATCCGTGCAGACGGACCCCCACAACCCTATCGCGCACATCGCTGCTCAGTTGAGAAGATAGAGGCACTCACGCCGTTATGGAGTGCTTCGATCGGAACGAAGCATCCTCAGGCATTCCGTGGACAGCCAGCCTGAGTCTTCTGGGCGATATCGATCGGCGGAAAGCCCGAAGCGATGTCGACTGTGAGGGCGGGAATGCTGTGTCTCTTTTGAGGTCAAAGGCCGAAGAGTGTACTGTACATGGATAAGTCACAGCGAATAGAGAAAAAAGTATTAGCTGGATTACAGAGATAACTCAACTGTGTCCCTGTGTGCTCAGTTCCCGGATGGAACAATGTCGCGTCCAATGAGTGCC

>TU1846-GaLu96scf_15-716187-717237 ACCTCCGCAACACAATCCTCATTGCACGCACAACGCCGAGCGCAGAATCTAAGACCTCTCACGCAGTTGTCATCGCGAGAAAAGTGTGCTTACAAGCGCTCGTTATCATTTCAGACTAATTACGTAGACTTTTGAGTCAACCGCGAGTGGCTTAGCGCAGTCATCGACCTCCCCACTTTGTTCCCACTGGTGCCGTTCTTCGTCACCGCTATGGACGTCTCTTTGCAAACAGGAGGCAGACTGCCTTTGAAAAGGCCGGCGCCAGCAAAGAGACTCGCAGAACCACTGCAGGACGATCGTCATCCCAGCCAGGTATGTCCTTCTTCTCCGGCCTGCCACCGTTCATCCCGACGGACATGAGCTTCCTGAGATCGATAGTAGTGCGGCCTGATAGTGCCTTCCACACATGGGCTTGCGATTGACCCCGGATTGGCTCGGTCGCGGCTTCATCATGATAGATTCGAAGACGATAGAAGCGGGCCTCCGGCCTTAATTTCGTCTACCGGGCCACGAGTCCCGGAACTCTCTCTTTGCTTTTCTCTTCTACATTGCCCTCGTGCTTAAAGCCCCCTCGGACGAGGGATGGATGCAAGTCGGAGGCACAAGGGATTGAAAGAAATGCTCATGACCTCTTGGAAGCTGTGGAAAACCGTCAGCTTTGCTCCACCGTGACGACAAGCAGGAGGAGCTGGGGTCCGACGGGGAAGCCGCGAACTTCGTGTCTTCCTTTTTCTCGAAGATCCTCCGGAGGCCCACGTATAGAGTCATCCCATGTGCTCACAGGTCATCCTTTCAGGGAGCGAACATGCAACGCCCATATCCCGTTTGCATGCGCAGCCCAGCCATGGGTATACCGCTATTCACTGAAGCCAACGTAATACGCATACGGTTGCCCTGCACAACGGCTTGTCCGCGTCTTCGTCTCCCCTCCGTCCTGCCCTGCCCATTCCACTTCATACAACCGCTATGCTCCGGCCGAAGATGCCACCCATGGCCATCCCCGTCCATGGCTGTTTTGGTGTGTGCTCAAACGCCCCGTTCAGAACCTCCA

>TU1863-GaLu96scf_15-861991-862235 AGGATCTATTTTATAGGTACTAACTCGAGTCTCGCATGTTTACACATGCCACATCCTTCAAATCCAGTCGATGACACCAGCGCCCACTCGTATAACCGAGAACATGGCCGCCTCGTGCGGTCTCGAGGACGTTTGGTCCATCGTCGGTGTGTCTCTTCGGAGATGTGCCGCGGTGAACCAGCGAGTCGGGCGTATGTCTGATGGATTCTTTCGTAGATCATACTTGTTTTGTGCCGAAATATATT

>TU1888-GaLu96scf_15-971672-972088 CGACCCTCCTCCTGGCCTCCACACAGGTGAGTTCTTGAGCACTTCGACTGTAACATGGGCCAACAGGTGTGGATTCAGATAACCGACTCGTAGGCCCGGTCTTTTCTCATTCATATTACTTGCAAATTGCAACCAACCACCTCATCTATGAGGCCTTGTCCGACTCCGACCCCTCCTTGGTACGGTGAGTACTTCAGTTGTCGTCCTGCGAGCGTTTTTATAGACTTCCACGTTTTCTTCTTAGCGACCGCGAGGGGACACGGTAATTGCACGCTGAGTCTGAGTTCCGGTTGTGAATGCCTGCCAATAGTGTCGCTGACACGACTTTATTCAGGGTCATGTTATACTAGATACCTCGAGCTCGCTAGGTACCCTACTTTATGCAGGCTCATTCAGCACCTCCTTATTACCGGTCCT

>TU1890-GaLu96scf_15-975638-975867 CTCGCAGGCCACCCACGCACTCTCAGATCGAGGGAGAGCGGAGCATTCCCACTTCGTGTCCCCGGTCTACGGGTGGCTGTCTCGAATATACGAGTGCTCGCTCAGGTAATTCGACCCTACATCGTATCCATATTGCCTACCACCTGTGGCAGATCTGTTCTACGTGTTCTGCCTGCGCCGATCCTTGGCCTATTTGTATGGTTCGATATCCCCTCACGTCGACAGCCCTC

>TU1892-GaLu96scf_15-977659-978093 TTGGAGCCGCGTCGGAATGAACACGTATTAGCAAGATGCGGACCCATCGTCGTCGGTCTCCGACGGTGGCCTGACAAGCGTGGGGCGGTTGAGATCGATGGGGGCGTATCCGGACAGGCCGGATGTTGCCCGGAAGGTGTATATGTACCTAGCGGAGGACAAGTTAGTAAGATAAGATGATGAGGAGGGAGCTCGTAACGGACCACATCGACATTGAGTCGCAAAAGTCGTTCGGAGGGGATGGCTTCATCGTACTTCATATTGCCAATGAAGCGGCGAGGCCTGGTGAAGACGAGGAATGGTGGCGGGAGGATATTCGCTCCAGAACCGTATGATTCAGTATGCAGCGTCCACGGGTGACAACGAGTGTCACAGAAAGGGGATAGGATAATGGAGTGATTGAAATTCGAGAGTTTGGAATACAAGGGTGAGACT

>TU1922-GaLu96scf_16-158013-158512 TCTACTCTCCGACCCTCTTCTCGCTAATGGAATGTATCGGTGCGCGCACGTATGCAGGTTACGACATACCAATCGTCGGATTTGAAGGACTGATACGCGTCAAATGCAGTACGAGACCGCCAGCATATTCAGTCACGCAACGGGATTCTGCCATCCGCTGGTGCTCCTAAAAGTCGCGATGTGATCGCTCTGCCGGACGATTTCCCCGATCATCATGAAGCATGGGCATGAATGGAGGCTCTCGCTCTTTGACCTCGGCCACAGCCCACCTGATAGCCTTAATCGGGCTTTACGAGCACCGTCCACATCTTTCTAGGACACGAGACGGGTCTCTTGACGGCATTCGGGGTCGGCGGCGAGCACCGGTGAGCAACTGGAACGAAGTGGCACATGGGAGGATCCATTGAGTCGAGTCGAATGTGGGGAGTTGCGCTCACCCCACCGGCCCTCGTACGACTGAGAAGCTCGCGGGTGCCTTGACGCTGACCTCGAAACGGCGT

>TU1933-GaLu96scf_16-190366-191149 TGGGCCTCGGCTCATCCAATTCTAGCACGTGGCTTACCAGGTTTACGATATATCGGATAGTAAGTACTGTTCCAGTTCGTAACCTCGCGTTGACGATCCTCCGCCCTCCTAGTCCTGTATTTACTAGGCACTCTGAGTCCTGTATGAAACGTCCAGGGACAGCGTGAAACGCGCATGCTCATCTTCTACGCGCCGCTCACAATCACAGGTATTTTCGAAAGCCCCGGTCAAGCGCGGTACAAGTTAAGTAGGGTGCGGCGTGAAGAGAGCGACAATTAGCACAAGAGCCACTACAAGCCTGCAGCTGTGGCGTACCTTAGGCAGTGTATGGATACCGCGATCACCCCGACGCGCAAATGAGCGGAGAAGAAGGACCCATCCGAGGCAGCGAAGTAGACCATCTTCTCGCCGATGGCGACATGTAGCTCCTGGAGTGGCCAACATCTCGGACTATGTCCGTGAATACTCAGGCTGTGCGCTGCGGTGCTTAGACTCCGGCAAGCCGCGAGCTCCTGTATGTGTTGTGGGCTGACTCTTGTGCACGGTGATGGCGGTGCATGCAGCATTGTCTACGGAGAAGATGTATCGACAGATTGAGGATCGAGGGGCTGTCCTCCATTTTAGCCACCATTTGCTGGACACAGGATGCTGTGAGATCGGTACCTTGTGATACAAGAGCGTTGTGTAAGTAACGTGTACCTCACGAAAGAATGTGGAACTCCCGACTTCAGCGAAGAAATGGTTGGGGAAATGGCTGGAGGCTCCTTAACGTAGGTCAGGGAGA

>TU1951-GaLu96scf_16-342363-342694 TTACTACAGTATGCCTCCAGCAAAGAAGCAGAAGACCGCCCGTAATGTGCGTTCTTCCGAGGCGGACGGCGCTGCTCAGGAAGGCGGGCCCACTAGAAACCCAGACCCCTCGACCTCGTCGGCAATAGTACCGTCTTGGAAAGGGGCCAAACGCCGGCGCACATCCCTGGAGAAGTTTGTCGACGGTCCTGTGGACATTGTAGTCGAGGTGTGCTAACCTGAAGACTTGGAACCTGCTCGCTCTCCGTGGTTGTTGTTTGGCACACGCTAACCACATTCGTTCCTATCCAGGTGTTGGCATTACTTCATCCTCGAGACTTCTGCACGCTCTG

>TU1970-GaLu96scf_16-464251-464922 CCTGCCACCACACTGCCATGTCCGACGCCGCCTTGACTCCGCCTCTCCGTCTCCAAACCGTCTCTGTCCAGCAAATCTCGTCCGACGTTGCCCAAAAACGTCTTGACAGCTTTCTCCATCGCTTCCGCACTCGCAACCTTGCCAAGAACAGCGGTGAGACGACCACTTCCGTCCAATTGCAGAAGCTTGCAGATGCCCTAAATGACGAACATTCCCAAGCCAGCTGAGGTCTGCCTTCATATCGTTTCATGGAACCCTTCAACTGATCACCTCACCTCAGACAGAGACCATGCGTCTGCACGTCGGACTACCATCGGTGGCCTCACGTTTGTCGCTATCTGGGCGTCGTGACGTGAGATTCGGCATTCAAGTCGTTCAAGAGTCGTATGATCACCACTATTTGCGAGGAGCTATCTTGCGTCCCATCGAGGTAGTTCATACCTCAACTGGACTGGTCAAGCGAGTCGATACCAGTGATCTGGAGCTCGGGCCGCGGTTGCACCGTTTCCTATTTTCAGTACGCCCCCGACACGTTGTGTACTTCTCAATGTAGATCATAAAGCGTACCCCCCCTGGAAAGCTGTCTTCCGCTTGGATAGGACACGACTTAAACCATTCCTTACGGCAGATAGTCATGCTCTTATTGTCCCACGCTGAGGTGGGCAACTTCGG

>TU1982-GaLu96scf_16-522134-522485 AGACAATAGATTTCAGGTTATCGTTAGTCACTGAATGGTAATGATCTCGGGCGTAGACCACTAGAATTTGTGCAAGTATCTCGTTGGGAGTCGGTTGATATGGGTGAAAGCATTAAGATGGGTCTTGAGGTCAAGGATATTCCTCTGATAGACGTAGATATCTCCCAGAATTGTACTGCGTTCCCCTGCAAACCTGGTGCTCGACTCGAAGGTCTGATCACCGTGTACTGGCGACTGCATCGAAGCTGAGGGTGAAATCTGCGACAAGGAATGATCCCTGAATCTTACAGACTCCCATAAATGCGGCTTACCTATGAGCTCCGGCTTGTCGAGATTGCCCCTAATGGCGGAT

>TU2024-GaLu96scf_16-785204-785693 GCAATGTGGAAGTTTCCGTATTCTCGCCACAGCGTCATGGTATCATCAATCGGGGTGAGTGTAGGCCCTTCCCGCATGCGCTCACTGTCATAGTCATGACCACCTTGATTGAGCGACAGAAGTGAAGCCGTGATGCGCGGTGCTGCCACCAACGGCGAATTCTGGATCTTCTTCGCTTATAAGCGTGGCACAGAGCGAAGCTATTACGCCAGAACGGAGATCTTCTATGCGCGGAACACGTCCTTCCTGGATTGGATACTGGGTATTCTCATCGATTGGGTGCGTAGTCGTACCGTTTTGAATTTTCGGGGCTGATGGCTAGGGGTTAGATTGAGAACGCAGGGTCCTGCGATGTATTCGAGTACTTCAAAACCCCATCATATAATTGAGGCCAGTCCCCTCTGCCAGTCGTTGTACTATCTACAATGTCGCTGTTTCCTGTTCGTAGCGTTCCGCATAAAACGTCCTTTGTTCCTTTGACAGCGGCACG

>TU203-GaLu96scf_1-1472540-1473031 GCCCTTGCTCGGAATCAGAAAACACTGCATAGAGCTGCATTGCTGCAACGCGGTTGGGCATGACGCCAAGACCGTGCGAGATCGGTGCGCACTTTGTTCGCTGTTTTAACCAACGAGCGAGCGCCGGAAAGAATGAATAGACGTGGATGGATGATCGGAATATTGGAGGGATTAAGGAAGTGAATTGAGGAGGACGGCGCAGTTGGGATCGTCCGCACTTGACCACGGAGGGTGTAAGAATAGGATGAGCGGGGAAACGCGGCAGGGGAACACTAGGGAACACTATGGAGAAGAGCACTTGGCTTGAGCTTGGCAGTAACTGAACTAATTCTGGATCCGGATGGAAAGGTCATTGATGCGTCAGTGGGCGGAGTGACTGCGGAGTGGGCGGACGTCGGGTGTTGTTGTGGATAGCAGGCCACACAGGGCAGGCATGGGGGCCAACAGGGCAGCAGGAGGTGGAGAAAGGGGAGCAGGAGGGGGCGCGACGGA

>TU2115-GaLu96scf_17-207318-207667 CTTCCCAACTTTCAGCCCAGTGGAAGCAAGCACTGTTGGTCCCCCCGGAGGACCAACAGGGCCTGCTTTTGCCGCTATCCATACTCGAGGTGAGTCCCCCAGTTGCCTGCTCTTCTCTGACCCTCGCAACTGCACCGTGGTGAGTAGGCTAGTGTTTGCATTTACAATATTGGACTAATGGCATCTGCTCTGTAGCAATTGCCAGATGGTGGAAGGGGAGCCGCGGTGAACAGGGGAGCTATTGAGTCCATGGTGAGTGCCCTGCAGGTTCCGACAGTGCTCGTGCTGATTCATTTTTACCAGATAATTGTTTATCACTGCTTGTATATCACTGCTCGTATATCACCGCT

>TU21-GaLu96scf_1-92881-93151 GTCATCACCATCTTCATTCCACCATCTCCCGCGCCCCGACCTTGACCTTGGCCTTTTTCGATTTTCGGTAACGCCATTCCGCCTCGCCCAGTCCCGCCTCGACCCCCGGCTCGTCGCCCACCCCCTGGCTCGTGTGTCGTGCCGTTACGACCCCTGCGTCAATCAAGCCCCGTTCCCCGTTCCCCGCCGTTCCTCCCCTCAAGCTTATATCTTTCATTTGCGGCGTTCTCTGCGTTCTTTGGGGTAAGTGTGGGGCCCCGCGCGGGGGCGG

>TU2218-GaLu96scf_17-989009-989257 AGCATTCAATACGATGGTAGTATCCGAGAGGGAGGAACTACAAAAACGTGCGTGGAAAGCAGACTAAGGGAGTAAACCAGTGTAGTATCGCAGCTTGAACATAGTGTATAAGGAACGGTCTCAGTGGTACAGTACCAAGAAGGAACCCTGCTCCGCGTTCATGGGAAACGAGCGAGGAGACGAGGAAACTAATATCGTAGGGAACGACCAATACTGCCGCGGAATGCGAGCGAGCGCTAAGATTAGTAC

>TU2239-GaLu96scf_18-106370-107427 GGAGCATGGCTCCAGTCACGGCGTCGCATTAGTACACAATATCTGCGAGCTTTCGCTCTTCGCCACGTAAGCCCTTGAATCCTCCAAGTCGGCTCTGGGTCGGAAACCCTCATGGCTGATGACCAGCACACTTTCGGAGCTCCCTCGGTTGTGGCTGGTGTGATGGGCTGACATCGTGGTGTTGACCTCAGCGGTGAGCGGGGGACGTAGCGGAAGGCAATAGACACTGTTCAAGCTTGGGGTCTTGAATCCGTTCTCGATGTGTGACCGGTTCAATGCCACGATCACAATAACGATGGTCGGATATATGGCCTATAGATATCCCCACGCGCAGTCGTAATCACTGTCAGTGAATGAAAGGACATCATTGAAGTTGGACATACTATAAGGGGCACGAGGGCACAATTTACGATAACATCAAACATCTGCCAGAAAACATTGTCATCTAACAGGACTTCCGTGTAAGTTCCCGCCTGCCAAGCGAGAATTATGACCTTTACGTTGTTAATCAGTTACCCCAAAGCCGTAGTCAAGGTTCAACTTCGTCAATTGAATCCAGCACTCACCCAGAGAGCGCAATAGATCATTCCTGATTCGACAAGGATTGAGAACACTTTCTCCACTTGAGACGCCATAGGGCCGGTCACGACGTATTGCTTCAGCGCCCTTCTTGATTGCCTGCAGCTTAGAATAAGCCCCAATACCAGCTTACATTTGGATTGAAGTGTCAGCATACCATCCCTTCATTGCAACAACGACCGTAGCGAATATGTTTGTGCTCAAAGATAGGGCTGTTGCAGTGACACCGTACGAAAATCCTTGATACAGGCTGCCGTTTCCATCAGCATGTGGATCCGGCCGCCATGAACTTTGACCGCTGTCAATGGCCAAGCATGAATATCTGGTATCCACGATGCCCAGAGCTGTATATCAAAATAAAATCTCAGTATTCTAAGATAAAACGTGAGGTTCTCCGAGTTACGCACCAAATGTCGTCAGAAGTAGCATGATAGACAACCCCCTGACAATTCTACTGCCCTGCCAGACTACACATGCTC

>TU223-GaLu96scf_1-1632003-1633287 TCCTCAGTCCTCTAGATCTCCCCAACACGCAAGAATGCTCCTTCCCAGGCCGTCCCTCGGGCTATCCGACGGGCTTTCCTCGTCCTCGTTCTCTGATCTGGCGCTATTCGACATTCTATAATAGCCTCATGCTGAAGGATAGCGAAACCTCACCTCCCCATGTTCTCCACCTAGCTCTTCTCCCCCGGACAACACCCGCGTTCGCTCAGAGCCAGTCCGTTTCTGTTTTATGCGATCCGGCATTCAATAATCGTCTTTTACAGTACACGATACGAGTCGCGGGCTCGCGTCCGGGACATATTCACCTCTCCTAGTCCTGATATCCTCCACGGCTCCTCCTCGGTCGGCTCACTTCGATCTCGTTACCACAGATCTCTACATATGACCGCCTATCTTTGGCTGGTTTGGTATGAAGCGCCCACGAGGAGCACTCCTCGGCACTGGACTCTCGCAGTGAGCTATGAGGCTAACGATCGGGCCTACGCGACTGTGTACGAGGTTAGCATTATTCTTTCTGTTCAGCATCCGATAATCTTACGCCCCGCAATCTAGGTGACGGTCGATTTCTCCGGGCAGTATCAGTCGCGGGTGATACGACGGGTCCACTTGACGAGCAACCACCCATCCGGCGCGTACAGCGGCAAGATCTTGCTTGGCGAGATAAACGACGGCGTTCTGTGCGCTCTCGAGGCCTACAGCGAGACTGCCGCCGAACTGGTCAACAGTCGCAACCACAAGCGCGGTCCTGGTACCCAGAACTGCCACGATTGGGCTACAGCCATTGTCCGCAGTCTTGAGGACGCCATGCTTCTACCACAGGTGAGCCAGATGACTTCGGACAACTTGCTGGTACGTACGGATGACTAAACTTTGGCATGTAGGGAGCGGTCGCCCGCGTAGAGAGATGTCCGCGGCTTGGCTGAGCTAGTTATCGTGCTGGATCACTGTATCCATAATGTTTGACTGCAGCTTTCGGCCTCATCCGTTCATGCTTTCGGGTTCGTGGGACAATGTGGTTTCTTCTCTGGGTTTTATGGCGCCGTCGTATTTGGTATACTCTAGGACACGCTGTTGTACGATTAATATTGGGCTCCTACTGCTTTACATGTATTCGTATTATTCACCGTGGTCATAGTATACCTCCCATTTCGTTTATTTACATCTCTCGCTTCTCTGGTTTGGTCGGTGGACTACACTATATCTGTGGCTTCAAGCCTCAGGACAACAATACTACGGGGGTGAGCTGCAGCAGGCCTGCTGCAGTTAAGTACCTCCAGGTTGGCTC

>TU2240-GaLu96scf_18-109289-109978 TCGTCATCAAGGCTCTGGGTAAACTCTTCATTTCTCTCATATCCTGCTGGATTAAGTGAAACGACTCTATTGAGACTTAGTTCCAGCTAAGCACTTGAAGCTCTGACTTCACCGCCGCGGCGCTCATGCCGGGCCTCGTCTCGTGTTCGCCAAACACAACGAGAACCTCAAGAACCTGTCAAGCTACCCAAGGCAGTCTATTCTATCGTACCAGAATCACTGCAGGTCGCCATTACTTAGTGGACACAGTTCAAATGACGATTCAGACGGTCGAGAAGGCGGGACTTTCGAATCTGCGCCCGAAGGTGTGTCTGTTCCGTGCGTCCTCTCCGTACGTTCGACTGCTTAAGTACTGGGTTCGTGCACCGTGTGTGCGAGTTTAAAGGTAAAATATTACTGAGCAGGCATGCTCTTGTACGGGACCAGCTCCCCATTGACGACAGCGACCTGTGTGCTCGCTCCATAGGGTCGACATTTAGCCCAGGCTCAGCGCCCCCCCCTCCCGACAACGATTCGTGCTTGCGGAAAAGCTCTAAGCGTGGAAAAATGCCGGGCTGCTGCCGGGTGAGCACTTCTCCTACAACATTAGAAAGTGTGTACGACCCCCAACTACCACTGATCCGGATCCATGCTCAAACGACCGCAAGCAGTCTACAGGACGGCATGGGCCGGGATTCAGCCCGATGAACG

>TU2272-GaLu96scf_18-228548-229251 ACTTGCGCATTCGGCATTGGGCGGCGGGCCCCAGGGTGTGGGGTAGCTGCGGCTGCGGGGCCGATCCAGAACCTTGACTCTGAGCTTGAGAGGTTGTTGAGAGGATATGTCCATACGTTGCGTTCTTCCTCCGGCGCCCAGGTGCAAGTGCGGCTGCCAAGCCACGATGATGAGCTCAGCATCTATGCACGCTATACTCGGGCAGGGCGGAAGACAATGGTATTGGGCGCGCGCTACGGGAGCTGCGCGGGACCACCGAGACCGCGTCGCCACCGCTCTCTCGCGCGACCGGTGGGCCGAGCTCAAGCCGGCGGAAGGCGAATGTGCCTACTACTACATGAGACGTAGCTAAGAAGAGACTAAAAAGGGATAGCGGAAGGGTGGGTGAAAAAGGAAAGCTCGACGCTGAGTACGAGCCCCCGTGGGCGAGAGGACGAGAGCTTGGGGTACAGGACCCCCGGACTATATACTAACGTGAGACAAAGAGCCCTTTGTTCACCCGGAGAGGGGTGCGCGAGTGCCGCTGCATAGACCCTGGATAATAGTCATTATCCACAACACCTTATTTTTCTTCTCATGAAGCGACGAGTCTAAGGGGACTCATATTTGCGCCCACGTTCACGCTGGCACACCGCTAAAGCCGGGGTTTACGGCGCCATCCATGCCAGTACTACTTAGTAGTGGAACTTCGGATTCGAGGTGAG

>TU2292-GaLu96scf_18-386283-387106 CAGGCAATGGTGATGTATGTTGACGTTGAAGGCCCTGTGCGATCATTACGGCGTTAGCTCTCTGAAATCGAAGACACTGTCGAATTGACTCGGTGAGTTTGGGCGTACCTTGTTACATAGTTGACAGCACTAGTGAGCACCACTGCACTGCTGTACCAATCGCTGCCGCGTTAGCAAACGCAAGTATTCCAAGGTGACCGCACGTCTCCCTATCGTACGAGCACAAAAATCCCGATACTTATGCGCACATCGATGTCCCGCTGCTATTTGATATTGAGAATGGTGTAGAGGAACAGATGGACCATGGTCTTCTCCCGGCAAGGGGGACGGATGAACTTAAAGCCTGGGGAGACAACGCAACATCAGGTCAGATAGACATGCAATGGGGGAAGGTGGGATAATTGATTGCATTCGAGTGTTCGGCGATGATGACGTCGAAACCACGCAAACTACTAGCGTACCACTCACCGTCGTAAGCATTTCAATATCCAATGCTCATCGATATCAGTCAGCTTCATGGTGCTCGCACTTGCAAATGTCGATCGCATTTCCTCCCTTTTCCTACATCTTGAAGACACTGGTGAGATTAAGCACACGGTAGAGGATGTGTTACAGGGCCGTGAGTCTTGCGAACTTTGAACGAGTACTAGTTCCCGTCTGGCCTTGGCAGTGCACAGTACAGTGGGCCGTATGTACTGTGATGGGGTGCCCACTAGCCAGTGCTTCTGGGTGAGCTCGAGGCCGAGATCATAGCCTGGTGCAGTAGGGTGCAGTTCGGTGTTCAAGAACAAGCCCGGAACATGTGGACGTGATGGCAAATGGCC

>TU2303-GaLu96scf_18-429706-430243 TGGTACGCTACCACGTAGTACACAGGGGACGCGGAGGAAAAGCGAGACACCAGCGAGGAAAGCGTCTTACCTGCTATCGCCCCCTCGAACCAATCCTTCAGCCACATGCTATGAGAGCGCGCGCGTAGTGTTCCTATGTCATGGTAAGGCACCGCGAGGAGGTCGAATACTTGAAACACGTACCTATATGCGCATATACTCATAGCAACCTAATCATTGTATTCGTGAGTGACATAGACAGGCTCAATGGCGTTCGGGGCTTACCTAGGACCGCATACATGCGTGGAGCTTGCTGAAGGGAAAGCTGAAAAGCTAAGTAAGATTCCGAATGAGGCACGGCGGGGACTGATACTAAACGTACCAAGTGGGGTTTGTGACGTCGATCTGGCCGCCCGGCGAGCTCAGCCGAGCTGCGAGTGGCAGAAAAGCTGCCCAACAGAGGGTTAGAAAGGGCCTATCTGAGCCGGAGCCAATGCATACCTGCATGGAGGACGGGAGTGAGGTGATCCGCATGCGCCGGCATGGGGACAGGAGAGGG

>TU2308-GaLu96scf_18-446182-446393 CTTCGCCCACACGTCAACCTCGGAGCCGTGTCATCGCAATACCCTGTCCCTGAGGCCCATGAGAGCACGAGAGTCATATTGGTATTATGATGGGATGTGTTGACTCGGTTGATGCGCTAGGAGAAGGAGTCCCTCCCTCACTTGGGCGGCCGCGCTTGTGAGAAGGCGGGTCACAAGGACCTTTTGTAGGCTTCAGCCTGGATTAAACCTGT

>TU2311-GaLu96scf_18-460663-461280 CACGTGCACGGTGTAGATTCAAGGTACGACGCTAGTTGAGATTCGCGAAGATGGGTGACAGCGGACAAACCTTGAGTATAACAGTAACGCACGGGGCTCGCGGGCTTGACGGTCTTGGGTCGTGAATGATTTGTCGACTTCTATAGGGGTGTCTGCTGGTGCGTGGTACTCATTTCCGTTAGACTTTCTACATGATGTTCTGGGCTTCAGCAAATTTCGGTCAACGACATCGAGAAAGCGCGATGGCTTCGAATCGTCTCACCTCCCGCGCGTGTACACGCTTCACCGTCTGCACTACTGTACCTCGCGACCTTCGTCTGGCGATGTGACGCTCGTGCATCATGGATCATAGATCATGCATATCATGGGTCGGCGCAGCGCCTTCTGCGGCTGGAGCAACGTTGACGTTTCTAATCGATAGCGTGCGGCTTCGACGCGACGCAGGCTCGCAGCTGACCCAAAGTTTTCCGGAGCGTCCACATTGGGGAATTTTGTGGCTAGCGCTGCCTTCGGCCACCCTGCTCTGAGCTGCCGTTTTTGAATGAGAGCCGATCATTGTACGCGGACGTGCGGGCACGCGCACCTTCATATCAGCGGATACGGGCGATTATCACCCGT

>TU2325-GaLu96scf_18-539188-539496 CAGGGTGAGGGGCGCGAGGACAGAGAGGACCCGTCTGAGGTCGTGGACCTCGAACCAGAGGGTGTCAAGGGCGATCTCTGCCCAGTGTTTGGATACGCATGCAGCCCTTGCGAGATCACGGTCGTCTAGGTAGGAAAATATCATGGCGAGGAGTTCGTGGATGTGGCCGAGGGAGCGGGTGTAGGTGGGATGCATGCGTGGCGAATGGAGCGTTTGGCGAACGCAAGGATTCTAGGACGACGGCGAGTCCAAGGGCGTGGGTCCGGGCGTCGACCGTGCGGAGGACGACGACAAGTGAGCCTCTTCGCC

>TU2334-GaLu96scf_18-565270-566406 CGTTCTTCTACCGCATCGTGGATCTGCGTCAGATACTGGAGTCTGACACAACGCACAGTCGTCCACTACACGATGGCATCGCGAGCCATCGCAATGTATCCAGAACTGTTTCTCGGTGCAAAGTATTAGGCAAATGTACAATAGCGCATTTGGGGCGATGTATAGCGATTGTCGGCGATGATTACAAGAGTTCGAGCAGCCCTACGTCCGAAAGAAAGACTTGGTAGACCCGCCGGGACCAGCTCCGAGTCCACTGTCTGCGAAGTTAATACCGAGCGTGCTTCATAAGGTAAAGAAAACCACCGAATTGATGCCTCCTACCGTCCTCTGGCTACGCTTCGGCGGTGACCGCCTAGTACAGGCGTCGATGTCAGGAGACAATCCCAAGTGATACCACATGGGGGGTGGTCGCCGGCGGTGCCTGGGAGAGACTTGAAAGACAGCCCGAGCGGCGATCACTTATACCTGGGGGGAATGACTCCAAAGGACGCAGAAGGCGGAAACCAAGTACACTTTTTGGGAGCCCGCGGAATGGAGCCTTAGGGTAAGGTGAGGGTCGCCGCTCCCGATGAGGCGTTGCATTCGCGCAAGGATAATCACAGCATATATGCCAAGGCTGCTGCCGTCAAGCTTTTATAACCACTGGCAGGGAGAAGCACGGCATCAGTACAGATACACTGAATGAAGCGGACACGCTCACGAGTAAGGTACGTAGTATGCCGCGGAAATCCGTGGACTCGATCAGTAAGCCGAGGGCCCTCTCTTTCCGGGGGTTTCGAAATCGAGCTTCCTATTTGCGTGTGCCGTCAGCATGTAGTTTTTTGTTGCAGTGTAAGACAGCCGCCAAACCAGCAAGTTGCAACATCGAAATGATGCCTCCGTCTCCCCATAGAAGGGCATACGTAGGTGCTCCAATGGACTGTGGTACAGTCATAGTATCCTCGCCCATTGGTCGAGGATTGGTCGACGTGGCAGATTGAAATCGGAGAAGTTAGGGTACCGCTAGTGAAGCGTCGACGCTGGATAGCTAGTACGGAGGTGGGTAGCGAGAAATACTTCCAAGTTCAAAGGTAGCTCAGACAGATTTTCAAGACTTGAACTTGAAGCTGAGAGTCGGACTCACATAGCGGGTGGGCG

>TU2387-GaLu96scf_19-156698-156995 ATATATTGCATGGGTAATGCGAACGTACCCTATATACAGGGGTGGAAGGAGTGACAGCCCGTAGGTTTCCAAGACCAAGTGGAAGGTCAAGTAGCGTGAGGAGCGAAACCTAAACAGCAGAAGTAGATGCGGCGAGTTGGCGGCGAGATGGGTTTTATACCTGTCGTGTGTCGCCGAAGGGAAGCGAAGTGCGAACGTTGGCGGAGTCTGACCGGTGCGCCATCGTGCACGATCGTCCCTGACGCGATTTGTCGTCAGAGGTCCAACGTGGGCGGTGCCGTTGAAGTCTTGAGGTTCC

>TU2389-GaLu96scf_19-161923-162420 TCGCGCCTGCATACAATTCGTGCGAAGCTTTCCAACCACAAAGCGGTGGGATTGTCCGAACAGTGGCATGGTAAGTCTCGTAAGGACCCGACCCGACTTGGAAAATGGGCCACAGGACGCCTCTGGACAGGTGTCCTCTGCTGCCCTAGTGCTACCCTTCGGCACTAACATTGAGTCTATCCACCAGGGAGCTTAAATCGACAGGCACTCCCTGTCGGACGGTTAGACGTTCCTCGCCTTGGCGGAATCGAATACCTACCGCGTGGCATCGGATTGAGCGTTCAACGTTCAACTGGGAGAGGGCAGAAATAACGTGAGCCGGTCTCGATCAAAGGTCAGTGACCTTATCAGTAACCCTGTCCCTAAAGCTGAGAGCCACCGTCGGTCCCTTTTGAGCCGTTAACCGCTCTAAACCTCGATTATGAGATCTTCGGCTGCATTCGTTGTAACCCGAGAGCGGGATGTGCTCGGCGTGGGTTCAAGAGATAAGCGTCGTGG

>TU2407-GaLu96scf_19-253411-254965 ACGAATGTGGCATAGCGGCTCTCAGCACCTCACCTCAAATGTTGGCGAATATCAGTGTCACTGTGCTGTAAGCGTATATTCCATCGCGATTCAGATACTACCACTAGGTGCATATGCATCTCCCGCTTCCCGCAGTCGCGCATGTGTGCCCTTCTCGTGTTTCCTTGATCCATATTCTTCTATTCTGGCACATTGCGAATGTCGCGCGAGCTTTCTTGAGCCGCAGCGTAGGGCGATTCCGTTTCATATGGCTATACGTTCGGACTACCATGCTCCATTGTCCACAGCTACTCCGGGGCCCTTCTGACTCGGAGGTTTCGCATATCTATGTTGATGCTGACTTTGTCGTCACCGATTTTCAGGTATAATACTAGCGGCTCGGACGGAAGTTTCCTCTCGTTGAATCCATCCGCTTTAACGTTATTCAGGTTGCATCGACGCGATGATGACCGCGAACCGAATCCGTCCGGTTCTCTACGAGTCGCCCGTTCAAACCGGGTGTCCCAATCACGAATGGCTTCGATATGACATGGTGAACCGAGCTTAAGGGTCAACGTACTCACGATATTAGACTAGCTGCGGACAAAGATGGTCACAGGCCGGAGATCGGTGATGTTCGCACTCGAAGGTCAGCTTGCGCAGCACCCACTTCGCTCCGGATTTCAAGCATTGATTGAAGGTGCCCGACTTTCGGCTTCCTGCACAAGATATTTTTCAACCAGCGTTCAAGGATCTGCTTGCCTCGGTCCCCATTGAGGTGAAGGTCATCCTTGCTGCACAAAAACTACTACTCAAAAGCCGTAACTACTACTAGAGCACCTGCGAAGTGGGACAAGCAAACGAGAAGGCTTGTGCTTCGCCCGCATGACCTCCAACATTGTTAGGCTTGTTCTTCCGCCACTGCCATCATATTCACGCCATCTTCATCTTGCTGACGGAGAATAAAGCTCTCGCTGACCATGAAGAGCGAAGCCGAGGCTCTGAACCTATTTATGTGCTGCCTAGGCCCACCGCGCTCAAGAGGCAGCACATTCGATCTGCCACGTTCGACCCGCAGCTCGCGGGAAGTTCATATGGGTGTGGGCGGACGGTCATAGAACTTTCGTCCGTGCACGCAAGTCATCAAGGTGAGAAGGCTTCCTCGTTGTGTCGTTCACATTGGTCGGCCTGTCCCTGACGTACCTTGAAGTTTATTTACGCTTCTGACAATACATTTCGTCGCAGGGGCCGTGCCCAGGCTGTGGTTCTCCTGCGTGTCTTACGCGCTGTCCGTACGCGGGGCACGCGACCTACATGCGACGCCAAAGCTTAAATTCCCTCTCCTGGGTTCTGCAGGCTGCGGTGCGACGAAAAAACACGATTTGAAACAGTCGATCCCTGCGGACTCGGAGTCTACCACACACATTATTGTACATGCTACGGATGGAAGATATCCTTTTCAACTCTTGCCTGGTTCAACTAAATCTGCCCATCACATAGCGAGGTCGTGATTTGGCCGCAATCCTCGGCGCTCAATGTCCTCCTCGCACTTACAGCAGTCGATCAATCTGTAGGG

>TU2442-GaLu96scf_19-538695-539773 ATCTCATTTGTCGCCACCAGGACACTGTCACCTGATATGATGTGAGTATTCATTAATTTGTTTTCCATTACATGCTGTATCGATCGCCGGTCGTCTCATACTTACCCGATACGTAGAGTTCATTGTCCACCGCCGAACGACCCACCGATCTTCTTGCACTCCCCGATATATCCACCATGACCTAAGTTGTGAAGCTCAACGTTCACGTTCAACCCGGTTACCCTCTACTGTCGATCTCTCATGTGAGTCCACACGAGCGGCGAGCTACGGTGACCGGCTTGTTGATCCTACTGTACTTACGAACAGCGGCCACCCTCGAAGCGATTCCAATCCTGTGTCCGAGTCTACCATGCCGCGCTAGTGGTGAGCAATCTCAACCCTTCACGGAATCATCCAAACAAACCTAATAAGGCCCTCACGAGCAGCGTCGAACTGCCTTTGTCAGAGAATGGCCGTCATCGATGAGACCTTTCCCGACATGTACAAAGCCTGCGGGACTGCGGTCCGACCGGTCGCCCCGTACCAGTGCCGTCCCGCACGCGTGCGGCGCAGCTACCGCGGCTTCGTGGCGTCGAGGGCGATCCCGACCGCACTTGGCCCGCACAAATCCGACACTGGGGGGTGCTGACACGCCCGCTGTTGCAGGGTGCAGCTTCGCGACGAGCATATCCATGCACGAAAGCCTTGCTGCGGGACAAGCTCAGCCAATAGCCTCAGTAAGCATGCTAAACGGGACCTGTTTGGCTTCTTGATCGCTTAGCTAGCGTGAGCCATGTTTCCGAGGATCTTAGGGCCGAGATTCCCGTCAAGGGGACCCTGAATCCGACAGAACACTGCAGGACCACTCGATCGCGTACAGTACCGGGGCGCGGCACACCAACGGTCATGGGAGTATTACGCCTTACCCTCTAACGGCAATGAGCTGGGACTGACGCCGCGGTCTCGGCTGTGCTCATGACCCGAGGTAGGACCCCTGACAATGGGTGGGATGGCTGGTTCCCTGATCCGGACAAAATTTGGCCGGTCGACCCCCCTCAGGCTGGTTCAAATCCGGACAAAACGGGGTCAAAATCGGCACC

>TU2453-GaLu96scf_19-595332-595774 GCGTCTACCTCAGAAGATAGGTCGGAAGATCGGTCGGAAGATGTGCTGACGTCGTTTTGGCAGCCAAAAAAAATTCGGTCATCTCATCATTTTCGAAATCAGCTGTAATTTATCACAGAAAAACAGCCTATTATCAGAGGTAGAGGTCTTTCGCGAAGGTGGAGGTTGGATAGTAATATGAACGTGGACGATAAAAAGATTCCGCACGACGTATATCGAAAATACAGTGTTGGTGCTGACATAATTATGGTAATCGTGGTACTTGTACTTGGTCAAGGAGTTTGTTGTACATGTCTGGTATACGGTGGAAGTGGTCTGGTCCTCTTTGATATATACGTACGGTAGTGAGGTACGTATAAGTAACCGTATGCTTCTGAGATTAGGGACGCTCAGATTTCGAGCGTAATGTAGCGGTGCGTGCGGCCATGGTGTTGAAGCCTACA

>TU2458-GaLu96scf_19-622600-623271 CTCCCGGTGTCCAGCAGTCAAACCTGTCAGGTGTCCATTATTTTCTGTTTAGTTTACTTATTCGCTTTTGTCAGTTACTTCACCGGCATGGAGGTTGGCCCGCGTCTAGCGGTGCATCGCACGAACGAACGTGGATCGGAGTGCCCCAGCTAAGAAGCAGCCGACGATCGTATCCCGATGGTGGCTAACAATCTCTTCCAGTTGGGGATATGGTTTGAAGCGGACGAATCTGATTGTCTTGTTTAACAAGTGCCTTCCCTCATGTTGGGGTTGTTCTTGTTCTTTCGTTGCCAAGTGGAAGAAAAGCGACGGCTAGAACCAACTGCGCTGTTACTTACGTACGTTCCATCCAATTTGTCCTCTGAACGGTGCTGACGCCCTTTCTTCATACAGAGCAGAGGCCTGTGCATAAGTATGAGGGACGTGGTCGTTGTCGAACCCTCCTGGCCCAATCCTGCCTGGCTTCAGTACGTCCTTAAGCTTCCAGGCTTGCCTCTACTCATGCAACTTACGAGAATCGGGAGCGGAAGAACTTAGCCGCGGATTAATCGCGGGGTTCTTCGGCATAAGCGTTTTTAACGCCGCGTGCTCTAATCGATTCCAGCGAGACGCGAGATCTCAGCGGGGCGGTCGGTTAGACTTAGCATCGCATGTGAGCTGAGTGGTGTCCAA

>TU2461-GaLu96scf_19-626804-627629 GAATGTGAATGAACGGATTGGACTTCTGGCGACATGTTCGGCCATTACATTGCTCTCAATACACCGACCATAGTCCTAAGGTCAAACTGGCGAGCAGAGAGTGCGGCAAGAGGGAGAACAGTGTGTGGTGGCAGCGGAAAGCCCCGCGCGACCCTTGTTCTGAGACGCGTTCGCCGCGGCGTTGAGAGATTGAGTTAATTACCGACGTACCTCCCTCAGCCAATGGAGGCCACTCACCGAGGCCATCTGCCCGCAGCACCATAGAACTAGATGTGCGTGGCACTCAATTCATCGACCACCTAAAAGGAAATGTATTCCATCAGCTCATCTGCCTAACGTGAGGGGCGATACAAGTCGTACTGTCTCCGCTCGGTATTGGGTCCGCCATAGAATCATCTGTCAGAGGGTCCGCAGTCCCGATTGCCGTGTTCAGAATACGACAAGTGTGACTACTTACAGGTGAAAAGCGGGTGCAAACTGGAATACGAAGGGGGTCACTGGCAGCCGCTGGACGTCATGGACAACATTCGCGGCCGGGCTGCCACACCGTATTCAAAGCCAAACATTCCTCAGGGACATTGAGCGTCGGGCGTGTTCACTTCTTTCATAGGAAATTTTCGCAGGACAGAAGGAAGACGGCAACATGTGTTCGCGCTTCGCTCTCGCGCTCGGGCGCAGAGTCCGAAAGCACACATCTGGACAAGGAAGTCTGGCCCGTCCACGGAGTACTCCGTGCGATCGTACAGTGGCAGTCCACTTCCTGCGGAAGTAGATCGTTGAACTCGGCGGCCTCGAAGCGACTCCGGAATACAACGGCCGTAGAAGA

>TU2474-GaLu96scf_19-705342-705839 TATTTTTAAAGGTTGACGGTCATCTTAATTGGTCGTGGTGCTCGCTGACAAACACCCTTAGCAATAAGTATGTCGATTCTCCGACGAGTCGGACTCGGAGTCTGGCCATAATACTGACCATACAGTCATAGCTCACGAGGAACCAGTCCTGATCAAGTGGTATTTCCGTCTTAGCAAGAGGGGAAGGTTGGAGTACTGGGCCAAGCCATTCCTTATCTCCGGAGTCCGCAGCTGTGATTTGCGGGACAATGGCACGACCGCGATTTTTCACGCCTCATACGGCGTTCGAGGATCCTTCGGATGCGCATTGAAGCTTTGCGCGACTCGATTGGACCCCGCCTTCTTGTTTTCTACGACTAGCCTCGGTCTTTAACACCGGAAAAATAAATTTTAGATAGACTGCGGTGTACCGTTGTATTATCCCCTATTCTCGAGTCTTTCGAGAGTAGATAAGTAGCCAACGTCGTACATGGGCCCACAGTAAGTAGTACTAGTAGA

>TU2482-GaLu96scf_19-783711-784686 TGGGACTAGTACTGTCTCCACTGTCGTCAGAGACAGAGACTCGTCAACGTCGTCCGCGTCAACCATCGCACCCTCAGCGGAGCCTCAACAGTCGATATCGTCCTCATAGCATCTGCTCCCATCCCTCCGCGATGCGCTCAAGACCTTCTTGATATTCCCGGGGCCGGCGCAACTGATATTCTCCGTATGCTCCAAACATCGTCCCCCAACTGCTCCGCGATTCTCGGTCATCTCCTGATGTGTCTGTGTCCTATACTCCCCAGATTTAGATTGACTGCCGATTCGCTGGGTAGTCCACGTCCATTCAAAGCGGACCTCCACTTGCAAACCAAGCTGCCTAATTCGGAGACATGCCATGGGCGTCCTGTCCCGGGCCCTTGTCCTCCAATCGGTCGTACCCGCAGCATACAGTGCGACGCCGCGGTGGCACCAAACCTTTAACCAAGTAAGTTAACTTGATCTTCCAACTCCAGCAACAGAATCCCTCGTCGCCGTCCGGACTGACATCGCACGTTGCTTTGCGACGTTTAGACTGAATCACGCTGTCACCCCAGCGTATCGAAGGACCTGGGTGCGGAACAAGCCTCAGGGTCTCTTGCGCAATTGCTCCAGGAATATTCGGACTCGCCATTCATCTCGAGGCTCGACGTGCCACCCGGACTTCCTTTCCCCGCCCATCGGCTGCGCAGCCATCGGCTTGGGCGCCCGGATTCCCACTCTCGGTGGCGAGGTTTTGACTACTTTGGAGGTGCGACTCTCACGTACGACGGCGCCAGCCTGTGCGCTTGGTACCTGGATATAAATTTTAAACGCTGTCGAAGGTTGGCGCGGGAGAGTAGGTGGCACCCAGATCGATTTGGAGCCAGTGCACCCGTCTTGTTACGCTATCGAAGTTGGAGTCGGGCTTCGAGCGACGGCGACCGTACTTTCACACCTAAGAGCCGAATTCACAATTCATAGAAGCCGTTTCATGGTC

>TU2500-GaLu96scf_19-874329-875763 ATCCAGTCAACTCCTGTGTCCAGTCGGCCGTGAGCAGTGAAGAAAGCGAACCTTTCTGACTGCAGGAATGCCAGGGTCTAGACATCCAAATTCGATTGCATGTTAAGGTACGCGCATCCCAGGCCTGGGCCCAGACTCTTCGCAGTCACAAGGAAGCTTCGGAACTGCTTGAAGTGAGCACAGGGTTAGAAACGAGCAGCAAAGCGGGACGTGCCAAAAGTATGGTTAAGGCAATCGAAGGGACAGTGGTAAGGCCGAACAAGACGACAGCCAGAGGCCATGATCTCTTGTTGAGTGCAAACACGCGGAGGCAGGTAAAAGCTAGGAGACGCCGGGATGAGCGAGCGTTAAATGTGTAACCGTCGAAACACGCTACCTGCGGATATCCAGGTTAGAAGCCTTCCTAAGATGTCGATGGTTGGAAGCAACACAGCACAGCTGCGGGGCAAACTGTTCGATTATCAGCACCGCGGCAGCATCAGAATACCGCATACCTCTGCAGAACGGATTAAGTAGGGCTCAGTTTCCCACTGCACGACTCCACTGGCAAACAATGGCTCATGAGATTACTTACCTCATCCGATAGACTCATGACAGCCCGAACAGCATTTAGGGTGCTGTACAGGAAGCCGATGTACCTGTTCAAGGTGAACATCACAGTTGCGCCCGTCACACGGCCGCTCGTCCAAAACGTAGCCACCTCATCACCGAAAGTCGATATCCAATAGTGCACAGTGAGAACTACGGTGGTGGCGGCAAAATCAGGGATGTGAATGGTTAAAAATGATTCGCAAACATGTCGTAGTGATTGAGCTCACCAGCCATCGAAAGGGTGCAATTCTGAGTCCTATGCAAACTAAGATACTCATAAATTAGATAAATCCGTTCAGCAGGCAGGCAATACGCAATCTCACTAGACTCTACTAGTGTTGAGTAGGACTTGGAAACTTTGAGAGTTGGTTAGAGGACATCGCAGGATGGTCCCGAATAAAGGGTATGCAACCCGGGGAGTCGTCCTGGAGGCAGTGTGTATCTGTTCTTGAGCGGATCGTGGTAGCCGTCCAAGTGTAGTGAAGTGACCAATGGACCATTAGGAGAAGTTGTAGTCTGGCACCAGGCTGCGCAGCGCACTTCTTTGTTCGCATCGGCGACGCCGAGTTCTGCCGCCCCGCTGACAGGTTCCTCGACAGTTCGACTTGGAAACCTTCGAGCAAGTGGTGGTCAATATCTTATCCGCCACAGCCTCCTACATCCCGCAGCCTTTTGTCGACGCAGTGCGGACAATTCGGTTCATTCGAATTCCTTCCATCCGAATCAGGCTGAAGACCCCCACGTCAGCGGATCGAGGAACTTGAAAGACAACAGTGACCCACAGTGAGCACTGAGTGCTGTCTTCCGTGAGGCTGAATTTGAGTCCCGGAGGTATTTCTGATTC

>TU2527-GaLu96scf_2-132034-132295 TGCAAACGCCGACGTCGACGGCGACCTGACCAGCACTTATCGCTATCCACCCGAGTCGGACTGACTGTCGACGGTTTTCTCACTACCGCCGAGCTCAAATCATTATTTATAACATCGAACAAATCATGCTACTCCGACAGAATTCACCGGCACCCTTCTTCACGGCGCCATACCGGGACATGGGCATCGATGCGTTGGGGCCAGTGACCCACCAGATCGTCACTGATGTTCAGACTTTGGTCGGCGCTGCCGCACCGTCCGG

>TU2546-GaLu96scf_2-293814-294703 GTACAAAGCCACCTTTCCGATGGCTCTTCTGCTCGCCGAGCGGAGTCGCACAGTAAGTTACGTTCCGCCTCGAGACACGGTTTGTCGGTTCCTGATAGACTCCATTTCTCGGCGTTCTGTCCCCCAAGGTTCAGAGCGTCGACTGATCTGCTTCCCCGTGTGGTTCTCAAACAGCGTCCAGACTTTAAGCCCTCGACTATCTCGATATAGGAAGCAGCAACCGATCGCTCCTCCCGCGAGCTGTCTCAAAGCAGGCTCGTCATGGCGAACGGTCGTCCTCGAATGGAAATCGTTGTCCTTTTGTCCACTGCCAGGTTCTTAGTCTATCCTTTGCGGCACCTTGCGAGGCTTTGCTCTTTGGCGTCCGATCCAGGGCCCGCCCCTCCTCACTAGGCCTCACTCCGTAGCTCACCCCTGTGCTGTTGGCGACCGCACGAGCAGCCGACGATATCAACTAGCAACCTTCCTTGTCCGCTTCCGGCGAGCCTGGACCCGACCTCTCGCCGACCCTCAGGCTCTCTCATACCCTGCCAAAAGTCCGAATATCTCGCAATCCAAGACACTGTCGATCGCGCAGGTGAAACTCGTTTCAACGCACAGCATAGTTCACCAACCTTCTCCTTTGTCTGCAATTCTGCCGGATCTTCCATGCAAAAAGTGCTCGTCCCTGGCCTTTGCATCGTCGAAGATGGTTGGCGCTTTGGCTACCTATTTTCATTCATGCAAGAACTTTCCTCCGGCTCGGGCCGCCCCTCGGCCACACCACGCCACCACGGGCCGAAGGCAGCACCGGTACTCTTGGCTTACGTACCTCGCGACGTCGACCTCGGTCCGTACATTAAGCATGTACTTACTCCGGATCCCCACCGGCAGTCCGTGCTGTATAACGA

>TU2547-GaLu96scf_2-294857-295406 TAGTGCAGCCGAAGATTCGCTCTGCAGACATGTAAGTACGGCAGTACAGTCGCCTCGTGGCCAGCCGGTGCACCTTGCCAACGACGTCCCCCTTGGCCGCCGATTCCTACACCGATCGTCAACGCTGACCGCGACCGTGGCCGTCTATCTGATCTGGGCGGTGGTGGGTGGTGTTCGATGGCGGCGTTCTCGGGCCTCTGCGCCAGGCCCCTCGCCCAGCCCTCGTCTCACAAATCGGCGTGTTCCGTCAACGCATGGATGGTTAACCGTCGATGGTGGTCGACGTCGAACCGGTTTCTCCGCCCCGCCCCGTGTCTCCAATCCGACGGTCGCTCTCAGCTACGGTCATGTCCACGCTTCCGTTTGCGCGTCATAAACCGTCGACCGCATCGGAGCGCCGCCAGTGCCTGCAGCTTGAAAGTCAGGCCAAGAAGATAGCGATCGTGTGCGCCGAGGGTCGATCACGGTCTCATACGGGCACGCTCGCAGGCAACTTTGGGAACTATAGGACGCTGTTGCATGGGTCTCGTCTGCTTGTCGCGCTATTCTA

>TU2554-GaLu96scf_2-327288-328078 GCACGCCAACTTGTGGTCAACATCAGACACAACCTCCCTATATCATACAGAGAGGAACCATCCGAAATCCGATGCGGCGACCATGCTCCGGTGGTACGTTCTGCAGGTGCCCAGTAACCTTGTTACCTGCTTTTGGGGGCGCTGCTCCGGAGGAAGACCGCACAGTCATCATGACCCCGAAAATTGACTGAACGCAAAGACACACCGTGACATTCATGAATAGATGTTCTTGAATAGAGAGCTTCTACACACCTGGGCCCGGGGGAACTGGAAGAACGTATTCGGCGGGACCACATAGAACGCCGCTTCAATCTTTCTGATCTTCCGTCCGGCCTGGCTTTCGCCCATCCCCGGACATATCCTGGACGAAATAGAGGGATAAAACGGTTCTGCCGAACCAAAAACCATTCAACGTAAGTGAACCATCAGGAAGCTTTGTCACTACGCACGGACCTCATTACCCGCTGCACCGGTTGAACAGGGCGGAACGATCTCCAAACACCCAGCGCGGCAGCATTTCAGAAGATGTCGCGCACTTGGAAACCGGACGAGCGTTTGCACTTCGTCAAGGAATGGATCGCGACGAAACCGGTCTGCCGAAAAGTTTGATCGATAGCGTAACCCAGACGCAGACGGGCGACCGATATGGCAACGGCCTTGTTGCGGCGTCTGGGAGGGCAGACACAGAAGATGAATCGCGGGCGAAGCACAGTTTCTCGAACGTTCATCCAATAGGCAAGGGAGCGGGTTCGCAAGTATGCTCAACGGTCGGATGCACAGCAGTGCCGGGA

>TU2558-GaLu96scf_2-361783-362461 AGCGAAATGCTCGTCTTCACTGCCGCACTAGACCATGTCCGTTGCCGGGATCCCGTACGCTGCTTGTCTACACACCCTGCTCTCCCCCACGTTACCGAGACATAGATCCAGACTCGACGTCCGGATCGTCGCCGAGCGAGCCAGTGCGGCGTAAGGACGTCGCCCCCCCCTCCCGAAGGCGATATGTTTCTTCCCATGAAGTGCTGAGGCTGCCGCTGACTTTGTCGGGCGCATGCTGCGGTGGCTGCGGTAGCGGCTCCCCGGTTCAAGCTCGCGACTCAATTGCTCAAGCTGTGTGCTTATCATCGACCGGTACCGCTCAAACTTTGGGCATCGTGAACTCTTGACTTCATAATTGGGTAAACTGTCTAAGCTGCTGCCGGTAGAAGGCTTAGCGGTGCTGACGAGTGCGACCTATCGGAATAGGGAAGAATGGAAATGCCGTGGGCAACTGGAATGAACTCCTCCTTCGGCAGCGTGGGAGCAGAAGCCTGCAGTGTTCTTGCGGATACTTACTATACATACCCAGTACTAGCAATACAGATCCACGAGCTGGATCAGCACTCGATCAGTAACCCCCTCAGAAAGTACACCGACGCCGACTTCTGCATTTCACGCGCGGGTTCAAGCGGAAGGCCGAATGATTGATCGATCGAGCATTGTTCACTATGTAGGCACA

>TU2563-GaLu96scf_2-374707-375131 GGACCTTGGCTTCATGGACAAAATCGTCCCTGTCCTGATGACAGGGCGACTGCAGCCATCAAGCCATCTTGGACTTGTTGTTCTCTGCGTCTGACGCGCTGTTGAAAAACGCGCACGAACATGAACGTGAACACGAACCTGGACTGTAATTTTATCTGTACCAGTATTACCGCCTCAGCATTCTTCAAGGTAGAAGTACTCAAGAATTCGTAGTGTAAATTATCCTATCTGCTCTGCTCGATACTGTACCTGCTCTCCTCAGATCTTGTTGTCTTGTCCACTGCTCCCCACTCCCCATTCGCATTCCCCTGCTCTCTCCCTCCTCTTTCGTGTTGTTGTCCCCGCTCTTCGCCGTCTGTTCTTGGCCTCTTGGTCTCCAAAGTCGCCGTCCCATCCTAGTCCTTATCGAAACCTCGCAATCACAT

>TU2564-GaLu96scf_2-375328-375991 CAACTCTTCACAGTTTCACGTACTACTATTAAAAGGAGAGTCGGAAGTCACTAGTCAACCTCTTCCCCCCACTGCACCTTCGTGCAGCCCACCTCCAGGTGCCGAAACCCCACCTGAACCTCAAACTCACCCTTGGGTCCCAATTCCATGACGATTCTAACCAAAACTGACGTACGTAGACCCCCATTCCGTTTAATCGTAGCTACTGACCGTCTCAGTTAGGACCATATCAGGATGGCGGACCGGGGGACGGATACCTGATTAATCTGTACGATCTCAAGTTGATCTCAAGCTAAGTAAACATCCTCAAAAGCCACGATCTAACGCCCCGCGCATTCAGCTGTATGGATGCTGAAACCCTCGACAATACTGGACCCCGGAAATATTGTGCGGCTCTCAAATCATCCGTTGAAAAGACGCGAAAAGCGCGACCTTTTCTTTCGGTCGACGCAACGCCGAGGCGCCTATGACCTATCGACCCCCGCTCTCGCGCGCATTCAGTGCGCGTTTCCACAGAACGACTTACGGCTTCTTTCAAGCCTGCTGTGAGGTGTGCCCCATACTCTCCCGTGCATCCCGTATTGCACGTTGTATGCGCGCACCGTTCACTTACATTGACAGGCCGACAGGCGTTCCCGTATGGGAACTCATGTACTACATGCAG

>TU2584-GaLu96scf_2-595069-595921 TCAAGCCAGCCAGTCCACCCGACGACTTGCGGCGGACCAGAGGAAGGACAAGTCCTCCATTAGGACCAGATGAACGATGGTCCAGTTGAGCGGTTGAACCGAGAGAGGGAAAGGATGCTATAAGCCCAAGGGCAAGGATGCGGCAGAAAAGGGACATCATGTTGAGACTCGTCACGGAGAGGACGGGCCCAGTGATGCTGGCATTTTATGTCTACCAGGGAGCTTGAAGTCGTGTTCAACGTGCCCGAGGGCGTGTCGCCGAGACAAGTCACGGGCGACGCTCATAGACCGCAAGCGCTGCTGGTGTGTGCGTGCTTTCTCTCGAGCACCGTCTTTACGCCCACAGGACATCGTTTTGTGGTCTATAGTGCAATTGAGATCCCGTGCAAGGTAGAAATCAGAACAGGAGAGACCAGGGTGCGCCCGTCTTCGCCGATGAATTTGAATGGGGCCAGATGGCCGGCATGTACAGCATTAACCAGCACATGATGCAGTTTTCCCAGTGTCCCGTTTGGAAGAAGGGTACACGGGCTAGATGGTGCGCTCATAGACATCGCCTCGACTTCCCAAAGCTTCTTGATAAGCACACTGCCGGGCGGCGAGCATAAACTCCTAGGGATAATTCACTGCAAGGCGTCGGTTGCTACGAGCAACGATACCCGGCATCAGCATACGCCGCAAGAACACAACCTTTTCAAAGATGAACACACGCACCGATTCCAGGGATTCCCAGCACGAGCCGCCAGAACGACTAGGACGATGTTACGCGGCGGATGTGTCTGTGTCTTGCGTACACTGCTGAGATTTGAGGATGGTCTACGAATATGCACAAGCTTAGGGCGATGGAATGGTC

>TU2590-GaLu96scf_2-619640-619878 CCAACGATGTATGTCTCAACGACAGCTCAACGACGGTGAACACTCTTTCCACATATAGTGATGCAATTGGCCTGTGATGTCCCCATCCGACTGCCCCCATCCGACTGCCATACTCATCTCAGGCTCAAACTTCCTCACACTGGTATACTACTACTAGGCTTTCAGCCAACTGCGTACGGGGTGGCTAAAGCTGGTCCTCAAGCCAACAGTGGTTTGAGTAGGTATGTGAGACAGAGCAG

>TU259-GaLu96scf_1-1961621-1962223 ACTGTCAGCCGTTTATTAGTATTACTATTGCCCTTCCTCGAATACGGTGATCGTCTCTCCTCGTCAGCACCTCGTCGTCCTTGAAATTGCACCCACAGGGACAATAGCGGGGTCATTTCGCAAATCTCCAAGCGGGTGGCAAAAGATTGGCAAGAGGGCCTGAAGTTGCTTGATCGTCCTCCAGTTCAGGTGTCAGTCTGTTCGATGTTCAGTTCTCAATCATATCTACAGCGCTCGGCTATCCTCTCCCGAATCCTCACGCCCTCTTCGTGCGCCTGCGATCATGTTCCTTCTTTCTCGCACGAGGGTCGCGAGTTGCACCAGGTTGCACCCACTGACAAGTTCTATTACTCCCAGCGCCTTCCCAGACAACTCTAAGTCATTCATTGGTTGGCTTCTTATTACTATTTGGTGTGTGATCCCGTTATAACCCCTCTTATGCCAGTCATCATATCACATACCATTGTGCGCAGCAATTAATAAACCCACCTTACCTACGACCACGAGCTAGAAGCTCCTGATGGACGTCCTCCCCACGTCCGCTGAATTCTTTGGTGGCGCACCCTCCGTACGAAGCCCAGGAGAGGCGTTCATACGTCCGGC

>TU260-GaLu96scf_1-1962322-1963127 GGCTCGCGCGGCGGACGATCAATTGGCAACGCCAAGTCGGCGTCCGGCGGGCCAGCATCGCGCCTTCGGAAGATGCGCCTTCTGCGATTCCTGTGTTGGCGGACGAAGCGCCGCGGCCGTTGTGATTCCTCCCGAGGAAATGTCCGACTTGCACGCAAGGGCGACGACAACGAACAGCTAGGGTCGGGCACGTTGCCTCCCCTCGGACCCTCAGGCCTCAGGCTTTGGGTAACCTGAGACCGCCTTTGCCCGCACGGCGTGCACGGCAGATGTTCCCCCGGCCCCGCCGCACAGCGGTGGCAGGAAATCAAAATGTACGCTGTCACCACCTCAGGTGTTCAGTTCCTCCTCCTCAACGTCAGGAATACGTCAGGCCTCGCACCGTCCAGCGCTCGTCCAGCGGCTCGATTGATTCGATAAGGTGACTGATGGGAAAGGCGTCCTGAGAGCGGTGGGTGGGCGGCGTAAAGAAAAGGCGTTCTGGCCTGGGATGATCCTTGTATTGGTAAATGTAATATCAGCGGACTAGACATCCTTGCGAATATTCGCAATACGTACACAGCACCGACATGTCATCTCTTACAATTCACACCTGGTAATTCGCAAGCTCCTTACGTCCGCTGTCGGAATCACTTGCAAAATTTGGGCGTGGGACCTAGCTGATTTTCAGATCTTCTTGGGTCGGCCAGCCCTGCAGACGTGATTGCCCGATGCGGTGATTCTACTGACTGGCAGGGGACACCGAGTTAGAGGTTCTGCCCTGAGACGGTCCATACCTGCGAGAGCGAGAGACATGTAAGAAAGCA

>TU2610-GaLu96scf_2-759235-760033 TCCTATTATTATTGACTATTCAATGCACAGAGACGCTGCCGATCACGAGCTGGGTGAGTGTTACTTCTTTTAATTCTAGAGGATGCCGGAACTAATATTATTGAGTGGCACGCAGACGATAGCTCCATTGTGATAAGACAAGAGATCACAGGTTTCTCCTTATCGTCGCCTCATACGAGCTGTGCTATCTCTCTGTGCGCCACCGGTGTCGATAACGTCGTAGGGAGTTCGAACTTAGCTCTGTGCCCCAGGAAAAAAAGATTTCTGTACTTATCAGAGGATGGACACGGGCGACCCCTACCACTCACTCTCCAGTCCCGACTCCGTACGCTTTTCCATCGTCCTCCCTTCGTCCAGTTGTTGGGATATACTCTGACATGATTGCCGCTGAACCTCATGCTGCTGCCAGCCTTCCTACCCTCTCGAGCTCTACCGAGCCCACTCCTTCCAAAAGGAACGCATCTTTGGAAAAAGCCGTCAATTCAGCAGTCGTGGTCGTCGAAGGGGTAAAAGACGAGGTGGCGGTGGTGGAAGGCGATGGAGCAGACTATCCGGAGGGTGGGCTAAAGGCATGGCTCGTGGTCGTCGGGGTATGCCCAGGCTCCATCGAGGGATAGCACCCATTCACTTTGCTCGTATGTAGGCTGTCTGCGGTATGTGCGCGACTATCGGGCTCATCAATGCATGGGGAGTAAGTATAATTCCTCGCTGCCTGCTTGAATAGGGTAACTGAAGCCCGCTCGCAGACGTTCCAGGCGTACTATAAACAAGTCGAACTAAAACAACAATCGTCTTCTGA

>TU2620-GaLu96scf_2-819152-819633 CATATTGCGGGCTTCCGTCTCTCATTCCTCTTCTCAGGGCGCCCTTCCGTGCAACATGAAACGGGCTCAGTTGTCTAATGTCATGCGATGGGTCGGAGCGAGCGTGAGTCATCTGTTTGCCTATAAAATTGCGCGAGCCCGGACACGCCCCAGTGCCATTCCGCGATCGTCCGACACTCTATTCGCGTTCTCTCTGCACGGCGAGCCCACCCCCGTATCGAGCGCATACCGTTTCTAGAACCTAGACTATCCCAGTAATCCCGCTGGCCCTCTAGATCGCCCTGCTACTGCTAGACACCCCCTAGACCGCGTAGAACGCTTCGCCACGTGAAATTCGCGATGAACGCAGTGAGAACGCGGGCAGCCAAAGGTGCGTGGTTCCGTCAGTGGCTGCCGTTGCGGCCGTTGCACTTGGCACAAGCACGTCTGGGCTGCGTACGAATATGATAAGTGCGGCGATACGAGCACGAACGTGAGCATGT

>TU262-GaLu96scf_1-1968035-1968408 TGCGACCGAGCTCGAGTCCCGTGCGCACCAGCGTGGTCAGCTGGAGCGGGGCCCCTGGCAGTAGGAGGAGCGAAGCTTGTGGTCTTGTGCTCCGGACCCATGGCACGATCGGACAGTCTGCGGAGGTGTTTGTCTTTACTGGATCAGGGTGTGCGTACTTACCCACAGATATCTACCAGGGCATCTCAGCAGGGCATCGACCACTAGTACTGGCACCCCCCGCCGGCCAACCGGTTCACCGGTGCAAAACGATGGATAACCAATGGATGTTGTTGACGCCGCTGATGGCTACGTCAGACCTCAGAAATACTAAGGACGACCATGCACAGGGAGGCCCAGGCGGTTCATCGATGGATCGCGCTGAAGATTCAGTG

>TU2637-GaLu96scf_2-895575-896928 CGACTGCTGTGTACCCGCTAATCGGGCGTGGGGGGCGCCACTTGCCCCCCATCCTAACCCATATATCATGGAAGTCCCAGTCTCGGTGAGCGCCCAATCCCCCTTCGTCCCCCCAGCCAAGCCTCCGTCAGAGCAGGCCTTCGTCCTGTTGATACCTTCATGTCGCTGTTGCCCACGCATTTAGCCTGTCCTCCGTACGCCTGCGAAGCGGCTCCGGGTCCCGTAACCGCGGCTATACCCTCCTTACCTTCAGGACCCGCCGACCGGATTTATGTGCTAGATGGCCAACTGGTCGTCGCTGTTCCCCTAGCCCGGTATGAAGGTACGTACCCCATTAGGCCATCCTAGTCACTTGACCCTCTGAGGACCAAGTCCCCTGTAGCCCTCATCGGGAGTCAACACGCCCACGCCAACCACTTCCCACTTCCCTCCCATGGTATCCATGCACCTATGCTTGAAAGCTCTGCGAGGCGCACATGGTCGCCCTATGGCTCTCCTGCGACCACGGAGGTGCCGTACGCCAACGCTTCGATCTCGGCGACTGCCGAGCCCGGGAACTCGAGGCTCGACAATCCGCGCGCTCGTACCCGCGTCCCTGTAGCGGTGAGTTTACTTGCTCTTCCCTCTCTCGAATAGCGGGTGATCTCTGTCGTGCCCACAGTGCGTCAACTGCCACGAATTGCGCAAATCATGCGACGCAGCGCGGCCCTGCCAGCGGTGCATGAGACGAGGTTTGAGTGCATCCTGCGTCGATCACACTCGGGCAGCCAGACGGAGCTCTGCTAGCCAGTCCCCGAGCGATAGTTCCCCGGCAATGACCTTGCCACCCGCTATTGGAGACAATGTGCCGTTCGTCGGTTAGTACATCACAGCGTCCCCACGATTATGCCAACCAGTTCATGTCTCATGTTCTGGTTTATCAGGTAGCGAGGGCCAAGGAGGAAGTAGCGGCCAGAATGCTCCGCTACTTCCTCCTCAATACCCTAACTTTCTCCTCGAGGAAACGGCCAATGTAGCCGCGGCCAACAACGTGGATATGAGCGCGCTGTGGAGTTCCTATTAGTGTGTCTCTGGTTATCAACTCTATCTCCAAAAGTGCCGCTCGTCGTTCTTGCATCAGAGTTGTCTGTTCCCCTATCCATCTTGCTTTTGACGTACTGCTCGCTCATATTTTACGCTGCTCAGTGACCCATTGATATGAATCTCGAGCTGTCGTTGTTTCGGTTTCAATACATGTTAAGTACTATGATATCATCGCTTGTAGCTATCCGTGTACTTTATGATACCACAGTGGTTCTCGCACACAGAATCTCATCGGGTGTACGCATACAGAGGCTGATGCGGGACGCACTCT

>TU2640-GaLu96scf_2-903370-903874 GGCATACAGGAAGGAGTGTAAGATGGCTGATGGGGGAAAGCGGACGAGGAGTAGGACTTACTAGTACTGTTGTAAGTTATCGAGCGTGGTCAGGATGTTGTATGATGCCGTGTTATAGCAACAGAAGAAAGGCCGGCCGGCAGCTGCTATACGTATATGTACAACTTGAATCCCTCAACCGCAGCCAGGTGGACGAACCGTGTGCGAGTTGATGAAAGGCAGCTCGCCCGTCTGCGGACTACCCTTCACTGGCACTCATCTGGTAACTGAACTGTGAGTAAATCTCTTGGAGCATGGCTCGGACACGACATGCCATTGGTACTTGTAAAAAAGGAGTGTTGAGATGACATAGGCCTCCTTGATTCCAGATGGGCTGTGCGGAGGATTCGATAGTCAGTACTGGTTTATATTGATGATTCGTGTTGACAACCTACTATTAATCTTCTAGTAAGTACTTACCCATCTCTTACCGATTGAAACTCTGCCCTGTCGAAACTTGAAATCA

>TU2653-GaLu96scf_2-950088-950319 TGGGGGGAACAGCCGAACAGGTTTCCTTCGCTCCTGGCCCACATTGGTGGAGGTGTCCCCACCACAGCGCTCTCGTACCATGGAAGCGCGTCCGGCGAGCACGTCGTATGGGTCTGGGACATCGCGGCTGGCGCCGCGCTCGCTGAGCTAGCGGGACATAATAACCGCGTAGACGACGTCTCCTTCTCCCCAGATGGGAGGTCCATCTCTGTTCGTGTCGCGCAATGGCTTT

>TU2676-GaLu96scf_2-1129791-1130936 GCGCCCCTTGGGTGCAAATGGAACATACCACCATGAGCATACATTGCGTTTTTGCCGTCCTCAGGGGTCTTTCCTCATCGTCAAGTGACTATTCGGTCTCCCTCGGTTCCGTACCTGCTGCGGAGGGGGTGTAGGGCTGAATCCCGCCTGACTTTGCTACCGTATTCAAGTCAGAAAATTTCTCTGAATGACGGAGCTGGTGCGGAGTCGAGCGGCAGACAGCAGTACCGTGCAAGGAATTATGTGGGGTCCGCTCTGACAACCTTCGATTTATGTTGAGGACTTCTGCTGACAAATTCGCCGAAAAGATAGGTACCAGGTGCCAGGAACACCCCGCGTATGTTGAGTGGAGTGTGATGCATAAACGCCTGCCAGGGTTCTCTCCCTCTCCTCACCTCGCGCGCGTAAACATTGTCGTCTATCACTGCCGGCGGCCTTATATAAGGTACGTATGTGAATGTATTATCAGTGTATAGTACGTTGATGATTCCTCTTGTTGCCGAACGATGTCATACTGTGACGTTTGATTGCACGCAAACGCTCTATGCTCGCTGTCCAGGAAGTTCGTGCAAGCCGTTGTAATCTTGGCCGGCCGAAGTACTTATTGACCGTAACTCCATGAAATCAAGATTTTAGTCATATCGTCATCCGTATACACGCCATCATCGGTCAGCCTTTCTGGGTTTCTACCAACGATAGTTACCGCATTTTGACAACGCTTCTTACACTTCTAGCTTTGGGAAACTCACGGCATACAACCACCGGAACCTGAGGCCTGAGGTATCGGGGCACGAGTCGTCATCGGTCGTCCGAGAAACATAGGGCTCTTGGACTCATGGTGATGATTTCCTGCCTCCAGGCCGTCTATCCGCTCGTCTCTCTCGGACTCGCAAGGTTTGACATAGTTATTGGGCTAGAGAGAAGACCGCCATCTGTAAGCAACGACGGACGGGAGGCCGGCAATCCGCCCCTTCAACACTGCGTACTTACGTTAACCGAGTCCCGCATCCGACGCATATTTATTCCACAGTTTCCACTCATGATATTTAAATTTTATGCTCGCTGCGGGCAACGAGTACTGTAGCGTTCGGCATTTATGTCAAACCAGGATTGGTCCCACTGCCCGCGCAGTGCCCGCACTCTTTG

>TU2684-GaLu96scf_2-1179829-1181186 AATGGGCTTGCATTAACGCCAGAAACACTATAAATCCCTCCAACCTGGACCGCAGACATTAGTCTCTACATTCCCTCTCGACCCACAAAGTGCCAACCCACCGTTAAACTACAATATGCGCGGCCTTCTTACTATCCTCGTCACAATCCCCTTGTTCATATTCTCTGCCTATGGGCATCCCGCGGCTGAGCCTAGCGGTGAATGTGAGTTCTACCATTTGCATTGCGGCCGAATGAATGCTTTCTCACCAAGCCATGCTGTAGCTGTTCCTCCAGTTGATGATGTGGACTGGTTCCAGCGCAATATCTGACAAACCGCGGAAACGGAAAGTGAGTACATGGGTAGGCCCCTGATATCTATACACAAGGCTTACATTGACCATAGTTGAAAAGAGGAAGCACCTCTGCGTCGATATTAAGTACTATATATGTGAAGAACGGATGTGATCAGTTGAATATGCACTTGTTGTTGTTAGAAAGAGGGGGGTCGCAAACTGAAACCGCCGGGCATGTGCTTCAATGCAATGAAGGGTAAAGTTTGATAAAAGTAAAAATGTTACTGTGCCAGGCCTAGGGAGTTCATGAACACGGGATCGAAGGGGGGCTGGCGATAGGTATCATACCTTGAATATTGAGCCTTGGCCCTTGCATAACGCTCGTGCTCTTGCATTACGATCAAGATCTCCGAAAAGCTCGACGTCGATGGAAGCAGCTTAAAACTTACTTTGACGATATTGATACAGAACCCACAACGCTAACTGCTGGGGCCAGATGTTCGTCACCAACCCCGCATACGGCGTCATGATGTTAATCAGCGTGTATGGAGACGGAGGAAGGTTGCACAGCCCTGCTATCTAAGGTGTGTCGGCGAGTTCGGGGACCGAGAAGCATTTGCCCGACTTCGAAAGATCCGGGGACATGTTCCTTGAGGTATCAAGTGACGTAGCGTGCGTTCTGATGTTGAAGTAAACGCAGTGCTAAAGAACGGTGGGGGAAGCGAGATGGAGAGTGGTCAGCAGTGAAGTCTAGGCCATACCGGGCGAGCGTAAATGGCTGACCATCGTGCTTGTGCAGCCCTCCCACCGATCAGGTCGGAGAATCCAGCAGTGCGAGATGGAAATCCCTGCATCCACCGAGTGTCGTGTCCACATCTTTCCGTGACTAGTACTGGGTCGACGGGCGCAGGCGGCAGCGCTCCGGCAGTTGGCGAGAACTTGACTGCGAGAAGATTGTGGTCACGACAACTTGAAGCGTCAGTTAGGTCTGTGCAACTTATGTACCATAGTTCTTGCACCGCAACTTCTCGCCATAAAAAGCACCTGAGGATGCATGAGCATGGGCGATAGCAGGAGAAGGAAC

>TU2689-GaLu96scf_2-1200490-1200733 CTATTTTATAGGTACTAACTCGAGTCTCGCATGTTTACACATGCCACATCTTTCAAATCCAGTCGATGACACCACCGTCCGCTCGTATAACCGAGAACATGGCCGCCTCGCGCGGTCTCGAGGACGTTTGGATCGTGCCTCCTCTCCCCCTTCGGGGTGGATTGGAGTGCGAACCAGCGAGCCGGACGCTTGTCTGATGGATTTCTTTTGTTCTGTAGTTTTGTATGTTGGCTCTGGAATACAT

>TU26-GaLu96scf_1-102782-103250 TCCTCCCCCCCTCTCACTGTTCAGGTCAAGCTTGAGCACGACGCGGTGACTGTAAGCCCTAGCTCAAAATATGCTCAAACAGTAATGATACTGACATGATTCAACCTGGCCTGACCATCGCCCTGATGGGCGTGACGATTTACGCCTTCGACCACCGTGGTGGTTTCGCAAGGCTGAACTCTTTCAATAGTAATCGTATTATCACCTATGTCGCATAATAGCACCCACCCGGATCAGGTGTGAATCCAATTCTGATTATACATGGTTAAAGATGATGCTTGGCTTACTACATCTGCGCCGCCGTGCACGTTTCCACAGACAGCAGATGTCTCTGTTATTGTCTCTTCTGCCATTCGAGATGCGAAACTCGCATTATCCAAGGATGGTCTGGGAATGAGGTACTCGTACTCCATTATCCATATGATAGAGCGGGGTAGTACTGATTGAATTCGTTCACCATATATATATA

>TU2701-GaLu96scf_2-1317789-1318562 TATTCAGTGGACCGCGGCGCATAAGCTCACGCCATCGTCATCGAGATTTGGGAGGCCAGAGCTGGGGCATGAGGTCCTCCAATATCTGCCAGCCTTCTACATACGATCCTCGCCACTGGACCAGCTGGGCACAACCATCGACTGACGACGGGCGGAACACGGACGCAGTCGTTGCCAGTCGAGCCGTGGACGGTCCTCGGTCTGGCCAGGATTCGCGGGTCTGGACGTCGTGGAGGTCGCACCGGCGTTGAGCACCGAAAAGAATCTAGACCGGGACGGTTCGCCGATTCATTCATGGACGGCGTGCACGACGGCCACGACGGCCCCGCCGTGCGAGGCCACTGAAGCTCTGCACGTCAGGCTTCCACATGGCTGTGAGGTCACAGTGTGGGCTCGTCGTGAAGCTTGAGTTCGTGTGATGGTTTGCCGTATGTACGATGATGGGATCCGTCCTGTGATCGGTCAGTAAGTATCGCGCGAAAGAACGGGTCGACGTCAAGGAGACCATGCTGTACGGACTCAACTTAAGTATATGATCGTGACGAAGTGACGTTGGCCGTATAATCATGGCGCGACTCAATGGCCGTTTCGCCACATGCCAGTGGGTGCACTGAAAGTAATGCCTGTCGGCGGGAGATTTAACTTCGATTGGAACTTGGGCCACGATGAAGTTCGTTGGAGCTCTAATACGTACTTCCTCCAAAGTGCTTTGGTAATCGCATCTCCCTCTTCCCCTTGCGACACCCAGAGGATGCTGAAAAACGAGTCAAGTTG

>TU2706-GaLu96scf_2-1403468-1403738 AAAGCATACCAAATAGCGCAACCTAAACAGATATGGCTGGCAGAAAACTAACGACGACGCCAATACTCATATAGCAGCAACCAAAAGCGCGCACGCACACGAACGAAACCGACACCGGACACAACCTCTGTCAATGCGTGTACTTCTACGAAGGGGAAGAGGCCCCTCGGCCCACGGCTTGTGGCACGCAGGAACCGGAAGACGACCTTCCCCGACCGCAGACGCACGCGCCCTCCTCCGTGTCTACCTCCCCTCCCCTCCTCTCCATCCA

>TU2724-GaLu96scf_2-1451287-1451843 TAGGCCGTCCGACTCCCTGGGCCTTAAACTTGCAATGAACACACTAGCACTCTCCATTTCTCCCAACGCAGCCTGAAGCTACATAACGCTACTCTTTACATAAGGGCGACATCGCTATCCGTTTCGCCCACACCCTGAAAGCTCATGTTTGATTGAATTTAGCGCCGTGACCAGAAACTACGTCTGTCCTCCGTCTTGGGAGTCTTGTGGCCATGCGGGGACAAGGAGTCCACTAGCTAATAGCCAAGAAACTTTGCACACAAAGGTATATTGCGGCTAAGCTACTCTGTTGAGGATCTCCTCTGAGCGGCGGTACCTTGTCGCCCGGACTTCATCAAGACCACCACATTCTCTCCGACGAGTCCGCTCGCGCCCAGGATTTCCCTCGGAGAAGTAGCAACGACATGTTCCAGCATGGTGAGCCTGTCTAGTTTCTTTTTCAATAGAAACTGCTCACTGGCCCTCGGTCAGATTACTTAGAGTTGGTTTCGCACCCCAGTGGAGATTACGCACGCGTTCGCGCTACCTGTACCTTCGATACCCCGATGGGAACCTTC

>TU2749-GaLu96scf_2-1618707-1619070 CCCTCATCATCTATGCAATGAGTACGGCCCTGAATAGCAGCGTTGAATCACAGTGAGGCTCTCCTTGCCTATTCCGCCGCTGAGCTCTCGCTGACATCAAGTGGGTTAGGGACATTATTCAACGCTGTCAGGTCTCAATAGCTGGTACGTCGCGGTATGAGCTCTGTCACGACGTCGAGCTGATTACATATTCCCCTCAGCTTTGCTCATTTACGAACTACTCATCACTTCGGGCCGGGAAGTGAACACCATATGGCGTTCTCGGCCAAAATGGCTTGCACTGCTCTACATCCTGAATCGCTACCCTGAAATCCTAAATGCAATCTCAAACATAGTTTCCTTCTTCCCACTGTATGATCAGGTG

>TU2751-GaLu96scf_2-1655746-1656795 TATGGAATTCTAAAGTATCACATATTGTTACTGGAGGCAACTGTTAACAAGGAGCGACCGTCTAGTGTTCAGCTGTGTGGCGTAGTTCGTCATAAGAACGAAACCCTTGTTTTAGTGGTGATATCAATACGTACCGACGTTTTACACTTCATGTCTCTCTTTGATGGACCTGTCGAACCCTCGTCCATTATACTCGTCATCCAGGGTCCACTGCGATGGCACATCGGAGACCAAGTCCCTTTCCGTTGTGAGAAGTCCTCTGGTCTCCGGTACTCCATTGTTCTCACCGTTCAATCGTGCACCGATCTTGGCAAGTTCCAGGAGAAAGCGGGACAGGAATATGGTGGTGATACTAACGTTGTGACGATGTCAGAATGTAGACAAATGTAGCATGGAAATTGTCACCAACACGTCTCTGAAAAGGGTTATTGACCCATAAAACTGATTGGCCGTGGATAACGATTTCTTGGATAGATTTGTCAGCCAACAAAGGTTAGGATGCAGTAAGTGGTCTTTACCACAGTGAATACGAAGGAAACAACTTGGAGGATGGTCACAGCTCTGCTCGTCGATGTCAGTCGAGATTTTCTGATAAGAGCTAAATGGTTATTTACGAAAAACATACGAGTCCTTGATGACGGGAAGGACCAGCATGAGTTTGATTCCCCTCCGTTTATAATGAAACTCACCATTTTCATACAAGACATCGGCAAGTGAAGGTTGGCGGCTCACTTTTGTCGATGAGCGCCATGCATTGTACGTCGCCCGGTAGGTCAAGGCTAGGACCGCAAGCTCCGCCAACAGATTGAGCGTTCGAGAAATATCCACCACTGTAAGTCACGATGTTAAATACCCCACACGAAGGAAAAATAGTGATAAAATGAATAATCACTACATGTCGGCAGCGTTAGATGCCTGAAATGCAGAGACACAGGCAAGCACTCACCGGTATGCTGAAACGAAACGACCCTGAGACCACTTCCAGGCAGTTGTGCAGGTTGGCGGTGTCTCAATATACACCGATTCAACCCCTATTACGTTGAGCTGTTG

>TU2763-GaLu96scf_2-1775021-1775359 GTCGTCACCTTCCTCGGCTGTTCAGTGTGCATAGGAAGAGATGAAGAATAACCGGTCTCAGAGGAAGAGACCTATTGCTCCTCGTAGTGACTCGGTAAGTAATCTCTCACACGTTGAAGATTGATTTGTCTCACGAGGAGATGCGTTCTGCTACGCCCCAGGTATCAGTTTTGCAAGGATCTATTTCCTGCGTCTGGGTCATTACTTGGCCGTGTGTGAAGTTGCGGGGACCCAAGTGGTGCTGTCAAGGGGGTTATCCCGTATCGTCAAAACGCTCCCTGATCACCTGCACAATCTGCACCCGAAAACAATGGGTCGGCGGCGATTCCGTACATCATC

>TU277-GaLu96scf_1-2071684-2072110 GCACTCTCACCCCATCGCCCTCGACACTGACGCTTTCTCATCTAGCCCTCAAAAGCATCTCGACGATGACTCGCTCGCCGGTACGCCGGACTTGAGTGGGTCGAGGACCTTGAGACCGTCACAATGGGCTGGAGAACAAGAAGACCTTGGTTGCGAATGATGGGACTTGGGTGCGGCGCATTCGTGCTTTGTTGATGGGGCCTATGTCTTAGCAGTTGGCTTTTCGTGGTCACTGTATGAGCTGCGTCGCAATGTCTCGTTGTACAGACTTCTTTGCAATTGACTAAATTTGTTGTTCTTCCTGTGCGTCTTGACGCTGTAATGCGTGTTTGTGATCCATTGTTGGTGTCCGTGAGCCAGTTTGCATCGGAGTGGGAACTGAGGCCTGTTGGGGACTGGAACTTGAAGTTTGCATGAATACTGCGGC

>TU2799-GaLu96scf_2-1913202-1913542 ATAATCCCTTGTGGAGAGAACGGTGCAAACGAACAGGGATCGCACTAGAATCCGTTGCTGATGACATCTTACGGTGGTCCCGCGATGAAGGCCTGTGTACGCGGTCACCAAAACGTCCCTAAAATAGCTAACATATGACGCCTACCGCATTGCGCGAAAATTCGACGAACTCCTGCGGTGGACACTTGAAAGGTCCTGACAGATTATCGTGCTTGAACGAAGACGCGTTAGGAATTCGGGAGCGGGTGTCGTTGGTCCAGCGAAGAAGCAGATGAGCGCACAGGGAAGTCAAGCAGGTTCTTCAGATTATCCAGAACTTGTCATGATCAAGCTGAAGCCAA

>TU2816-GaLu96scf_2-1968689-1969302 ATGGGCTGACATATTGTCAATTCTGTGAGTTACGAGTCTCTTTCTTCGAGTACTTTGGGTGCTGTTCGCCGATATTTTTTCCGGAGTCAGTCAGTTTCTCATGCCAAAGTAAGTGTCTACTTGCCAGTGAGTCTCATCGTCGGGCCCCCGGGATCCGGAGAGGCTGTCCGCCTTCCGATCTACATAACCTATCTGATCCGCATACAGTCCATGCAGACCACACGGCACAAAACCCCCAAATTACGTCCGGTCTACTTTCGCCCCGAGCGATAACGATGGCATTAGAAGCTGAACCGCCTGCCACAGCCTGAATAACCAATGACGAGCATCGCCTTCCCAGCGACTCCACCCAAGGATGGTCGCGTCCACTCTCCTTGTGAAGCGGTGCGTCCTTGGTAGTAGACTACAATGATTCATTCTCTGTGCCACTCAGTGCGACGTATCGGCCAGGAGGTGACTTCGGACTACTAGTAGCCGCTCGATGTGGCCTAGATACTTGGGCCACGACCACACCCGAGCCGCGTGTAGGCGTAGAAAAGAGTCCTGTGTCGGTGACACATAACTCCAACTCCCTTACCAACAGTCGTTCATACCTCGCCAGCCCTTCACACTAT

>TU2817-GaLu96scf_2-1969556-1970218 GGACTATTCGGCTCTCCTCTTTCTGCGCCGCTCATCTAACTCACTAGTAGCATCACTTATCTGTACAAGATTTTCCAAAATTTTCGCCTTCCATACAACCCGGCAACCGCTTCAGTGCCTCCGGAGTTTCGACAGCGGCGGGCTCGAGCGAGGCGTTATCCAGGCGGTATCTATTAGCCCCAATACCGGTACGTGCTGCGTTATGCTTAGTTTGGGGTGATTTAGCAGGTGAGTCTCCTTCCATGTTGCTCGGGGGTCTACGTGGGCAGTCTGTTGCGCGGACACCGTTCACCTCCGAGCACCGAAAGGTAAGTGGCGATCTCTCCACTATGGATACGTGTCCTGGTTGGTCCCAAGATGAGTTTCTTCTCCCCCGTTAATCGAAGGGCCGCAGTGGGTCGCTGCAAGTGTCAAGTACGCACGTTCGGTCAGTGGATGGGTAATGCGAGCCGGTACAAGCTTAAGTGAGTTCCTGTCCTGCTATGGTACGAGCCATAACTGACATTGCCTCGTACTAGATACATCATCATGCCTGTAGCTTCAGGTGTTCGCTGGCGATATGCGGGGCGGATATGGTGTGAGTAGAGGCATGTGAAGGTAGGCAAGTATGGTGTAGATAACATGTAGGCTGTTACGGTGTACTTAGTGCCGCATGTAGTAGAT

>TU2824-GaLu96scf_2-2003077-2003519 CATGTACGAACATGTATATTCAACTGCCTACTCGGATCGCTCCGCTGCAGCATCTTACCTTGTTCCCACCGGCCGGACGCGGGGACTGCGTGTATCCCGTGCTGAACCACCGTGTCTTCCTTGTTGCCCGTAAGACTGAGTGCGGGACCAAGTGCGCGATTGCAGTCTCTCCGGTGATTGTACTTTGATACGACAACACGTGGTGGCCAGAAACCCATGGTCGACACGCACCGCTCTCTTCAAAACCTGGCCCTGGCGCTCCCAGTCCCCCCGCACTCATTCAGCACCAACTCGCTTCTGTTTTCGCTCGACCTCACTCGCCTGCCGTCACCACCGCCTGTCATCCCTAGTCCCCTCTCACGCCGTTCCTAGTCTCCTCCCATCTCACTGTCGCGAACTTCTCCCCCTTTCTCCCCGTCTGGCCTGCTCATCAACAGGCTCCG

>TU2829-GaLu96scf_2-2024517-2025033 CTCGACAAGAACCGTCCTTCGACCACATTTCTGCCGGCGTGTGTCTCGGACACAAGTACTAGATGGACCGACTTCTTGACATGTCCCTCGCGCATCTCAAGAGCCACCACACAACGGACCTCAAAGTTTCACACTGGCAGTAGCACTGCGCGTACTCCGATGAGCTCGAGTACGAACAAGGTCTCCCAACCAGCTTGCGCAGTGTTCCCATGACCGGCGTGCTCAACTTCGTGCGGCTGACGGGCCCTGGTCCTTAGAAAGCCCTATGAAGAATTCCCACTTCGGCCGAAACTCTCTTGGGATCCGCCCTGCACGCGACCCGCCTCCAGTCCGCTTGTCGACACCGGACGAGGGATGCGAGACGGCCTGCGCAACGCCGTCATTGTCATAGAGCTCGCCGAGGAGCTCCGAGACGCAATCGCCTCAGGCTCGACCCACAACGAGGGGCGAGGACGCGAGGACGCGACCGTCTGTAGACTTGCAGCAAGCCGCGGTAGTCTTGTTAGATCCTCGACCG

>TU2831-GaLu96scf_2-2026526-2027024 AATATTTTTCCTTCGAAATGAGCCTGTTCCCCTCTGTGCGCTGGCCCCAAAAAGATAGGAAAGGCTCGATATCTTCAAAAGAAGCCTTGATTGCGCCGCTCTACCGCGTTTATTCAGTACACTGAATGGCAGCTCCATTGGCATCCAAAAATAGCTACATACACAGACGGGTGTACGCTACTCGCGGGCTCGTCAACAGCTCGCGCGTGGGACCGTACGGGCTCGCGCGCACCACACCCGCCGCGCGCACCCCCATATGTCGTGCACACCCTCCCGCTCGTCCTGCCCATTCAATCCGCGCACTCCCTCCCACTGCGCGCTCCTCTGATCGAGCTCCGCGCGCCCGTCGCATGCGGTCCTAACGATGATGACCATTAGGTGACGTCATAGGGCGTGCATCTTCACATACCTGTATTGCACGTGAGGCGCGCACCCGGGAGAACAAGAGGGAGGGAGGAGGCAGAGGGTGGTGTAGGTTGCACAGCGGGGAGATGGTGGG

>TU2848-GaLu96scf_2-2112289-2112673 CGCTACCGCTCCTGCTTTTCAAACCGAAACGTCTTTTGCACTATGTCTGCCCGTGTCACTCGCAAAAGGGCCCGCGCTACAGAGGAGCAAGAGGACCTCCCCCACCAAAGCGAGAACAGGATCCGGAGGACGCAAGCGTCGTCCTCACTGGAAGCAGCCTGGGCGCACAGGCAGAGAGGCCACCTGAGGCGAGAGAAGGACAGGACTTCAACAAGGACGACGAATTCTGGTTCGACGACGGTACCGTCATCCTTGTTGCGGGCGATGTCGAGTTCCGTGTCTACAAGGGGCTTCTAGCGGGCCTTTCCTCCGTGTTCAAGGATCTGTTTGCCGAAATCCATCCAGTACGCAACGTCCGCATCGACGGAGAGCAGTCGTTTTCGTG

>TU2854-GaLu96scf_2-2128028-2129177 TCAGGTCCCTCGCTGAGGGCAGCCCATGTCTCGGCATTACTTCCGTCTTACTCAAGGCGTTTCCTTTTAACTTGCCGTCCATCGATATTACGGAAATAACGCGTTACCAAGACAATCTAGCACCGGACCAAGTCAGCCTAATGACGTTGTGCTGGGCGGGGAGACGAAGGCGAGTTCTTCCTCCGACCTTTGGCGGGTTTCGCACATGTCCCAGATAACACCGATGCATCCGCGCCTCCAATGCAGCCTAGGAGGACCACCTATGACATGAAGATCTCGTCTTTGCGAAGTTCTTGTCGCGTAACCTAGCCCTCTTCAAGGATAGCACGCGCACGCCGGCATCGTGTTCTGTTACCCGAATGTGCCCTAGCGTGTGGGTCAAGCCAAGTCCAGTCATCGGGGTTCGCATCGCCGGAAACGAAGGGACGGGTGGCCGTTTAATATTTCACTGGCGTCGAACGCGTGGCGTTCGGTGGCGACACAGGTGAGCATGGTCGTGTCCTCGGTATTGGCCAGGAGACTAGATTATCGCAGCTGACGGGACCGCCATTAGGCGGCCAGAACCCTCTATACGGAAAATGGCAGGCGAGCATTCGTTCTATCATCTTTTCGGTCGCCACGTTTTGCCCAAAGCCACACTCGTGTTATGGGCGCCTGACAATCAGCGATCAGCGCACAACGCTGCAGAGTTTGCACAGCAAGCTCGCACATTGAAATGGGGGAATTTTCCCCTGCAATGGTAAGTTCAGATGAATTCCATATCAATTGAGTTTCGTTTCCCGCTTTGTGGCGCTGCGGATGCATATGGATGGCCAAGTCGAGGAAGAGACTCGATTAGCGTGGATGCAAGATATCTGGAATGCATTTGAACCCATGATTCGAGAGAACGGGACACTTGACTATGTTCGGATGCTCCGATTGTTCAATGTTATTGACAAATGGCGATACAGGAATACTAGGAGCTGTCCATGGTCTTAGAACATCGCTAGCAACGGTTCGCCAGAGGTGAGCTGGCTTGGTTAGCTGGTTCCACATGTACAGTCTGGATGTACCCTGGGAGTGCATATTCACACAACACGAGACAGAGTGTTCTGTACGCGTGTCGACCTTTGGACCCACAATGTCGTCGTTGGCGATTGAATGCGAAAGT

>TU2858-GaLu96scf_2-2149908-2150311 CAACACTAGGAAAGGGTGACCTTCCGGATGATTGGAAGAATCCACGGGCGGGAGTCAAGGAACTCGCGGAAGGACTCCATCTGTTGGGCGGTTTGGATGAGTATGTTTCTGAAGAGATGGTAACGACATCGAGGTAGAAATGCACGGCATGTGAGTGAGATACGGTGAAGAGACGGCGGATCTGCTTGGTCGATGGTCATCTCCCATAGTTCTATGGGGATTTGATGCATGGGGTGAGAGGTTGAAGGATTGGTAGACAGAATGTGGGTTTTCCCTAGGCTATTCCATGCATTCTTATACCACGACTGTCCACCTGGCTGCGCCACGATTCCGCCTGATTGCGCGTGAGGTCGAACCGGTTACCTAGGGACTTGCCCTGATGGAAGAGGGAGGATGTCGTCTTG

>TU2900-GaLu96scf_20-33822-34152 ACAGGACAACTGGAATACATCTCTTTGCTTCACTCGGACGACATTTTTCGCTTGCTCCAACACAAACGGATCCTAGCCAGTCTGGCCGGCTAGTAAAATATAGGGCTAAAGGAGGTCGAGCTCGCCGCTGTTCCGAGCTGCCTCAATGTCGTCATGAGTCAAGCCGCCACTCTCGCTCATCGCTACCCAAAGCTGCCTGACGAGGTGCACGCTGATGACGCTGCTTCACTGCAGCTGTTGAAAAATCCGAGGAGATGCCACTGCTGATGAGCCCGAAGAGGCTGCTGGAGAGCGTGCTGAGGCATGGCTTTTGAAGATAAGTCCCCTGCCA

>TU2904-GaLu96scf_20-47815-48418 CGTGACAGCCTACATGTTATCCACACCGTACTTGCCTACCTTCGCATGCCTCTACTCACACCATACCCGCCTCGCATATCGCCAGCAAACGCCTGAAGCTACAGGCATGATGATGTATCTAGTACGAGGCAATGTCAGTTATGGCTCGTACCGTAGCAGGACAGGAACTCACTTAAGCTTGTACCGGCTCGCATTACCCATCCACTGACTGAACGTGCGTACATGACACTTGCAGCGACCCACTGCGGCCCTTCGATCAACGGGGGAGAACAAACTCATCTTGGGACCAACCAGGACACGTATCCATAGTGGAGAGATTGCCACTTACCTTTCGGTGCTCAGAGGTGAACGGTGTCCGCGCAACAGACTGCCCACGTAGACCCCCGAGCAACATGGCAGGAGACTCACCTGCTAAATCACCCCAAACTAAGCATAACGCAGCACGTACCGGTATTGGGGCTAATAGATACCGCCTGGATAAAACTCCGGAGGTACTACAGCGGTTGCCGGGTTGTATGGAAGGCGAAAATTTTGGATAATCTTGTACAAATAAGTGATGCTAGTGAGTTAGATGAGCAGCGCAGAAAGAGGAGAGCCGAATAGT

>TU2919-GaLu96scf_20-98683-98960 ATGCAATACCTGGGGACGGGCAACGCCTGCAAAGCCCCCGGCCGCTTAGTCATCAGGCGCCCGACCCGAAAGATGGTCGAGCCTGTCGAAGACGCCGAGTCGCACACGCTATGCGCGCGCGGTGCGCGAGCGTGGCAGCCAACCATCGAAACTCTTCACCACCCTCCTCCACCGCATTGAAAACTCGTTTCGGCTTCAATTATGTGTACTCGCTCTATGGGCAGTATTGCTATGTCGTAATAGATTTATATCTGACCTGAATGCCACCGGTTAAGGTT

>TU291-GaLu96scf_1-2187072-2187410 AAATAGCTCTGTTGGGGTCCAGAAATAGCTACATAGCCGGGCGTACGCTACTCGCGGGCTCGTCAACAGCTCGCGCGCGGGACTGTACAGGCTCGCGCCCGACCCATCCGTCGCGCGCACACCCATATGTTGCGCGCGCCTTCCCGGTCGTCCTCCGCGCGCTCCCTCCCACCGCGCGCTCCTGCGATCGAGCTCTGCGCGCCCATCACATGCGGTTCTAACGATGACGACGACGATTAGTTGACGTCATAGGGCGGGTGTCCGCAACTTCACATACCTGTATTACGCGCGAGGCGCGGGCCTGAGAAAAAACAATAGGGACGGAGGAGGTAGAGGGCA

>TU2936-GaLu96scf_20-150535-150882 TTGAGCCATGTATACTATAACTGTAACTCAACGATAGACTACATACAGTGTAATGAGAGCAACGCAGGGGGATGAACGAACTGTGAACACATACGGAGAGAGGGGCGCAGCATGTGAGAACGCGATAACGTAACGGAAAGCCGACGAACAAAATATATGGAGGACGAGGACACAAGGACGCACCTATGGTCTGGCAGAGAAACACGAGTCAAGAGAGCGAGCTGAGATATTATCACGTACCTTGACATGAGGAGCCTCATAGATAACCGACTCAGACGGCTGGGGATGTACCTGATTGCCAACCAGCGGCCAGAGACGGACAGGATGGATATACCTAAGTGAACTGTT

>TU2939-GaLu96scf_20-157542-157877 ACAGTCCGAAAAAGGAAACTCAGGCGCTGGGTAAACGGCGCCGAATGGGAGCGTTCGTAACATGAACAGATCCAATATCCACCACGTACCTCTGAATTAAACTGTCCTACAAGCCCATTTGAACTTACCTATGACCGCATCGTGCATGCATCCGGGCCGGACACTAGAGAGACGTAGTCATCGTTGGGCGCGGTGTACACACCTTCAGCCGAGACGGACGGCGCCGGGCGGTGACAGCAGATGCTGCCCTGAGCCCGAGAGAAATCTGAGACCACAGGGGCAGAGAGGTACGTGAGGAGATGATGAATCGAGCCAGAGGATGCGCGGCAAGATGGT

>TU2952-GaLu96scf_20-207387-207740 TGCTAACTCTTTATCCTCTTGAGTCGATAAAACAGGCCATCATTTCTCCACGGTCCACGACTCGATGCTCAAGCTTCAAGCCGAACGCAACACGGTGAATCTACAATTTGATCGTGTATGCGCATTGATACAACTTACGCAGACCCAAGCGTGCCACAAGTCCCCTGCACGACATCCCAACTCACACCGTGGAATGCAGTCGTTGCTCAACCAATGGGTTCTAAAACCAAGCTTGCGACACGCATTCACATCTCTGGGAATCCGAGGAGATCGCGTTGATCTCCACGTAGATCGTACGGGGCGCACCCCATCGTCCTGCCTGGCCCCAGATATCGAGCCTCTGAAACACCAGTG

>TU2959-GaLu96scf_20-241098-241430 AGGCCTATGCCGAGTGCTTCGAGCTGGTCATAACCATCCATAGATAGGGAGACGGATGAGAAAAGACATGCTTAACAGCGGGAATTTAAAGTCCTGCTTCCTTCGTGAACCTTGTCCGGCAGATTGTCAATATGTTGATGCAACAGCGATAGTGAATGGTCGCGATGATCAGGAAGGCATGTCGCGTCCCCGCGTGCGCCCTGCCAGTGCTGTGCAGCATTGTAAGGATGGTTCCTTGCTAGGCGATGCTGCCTTGCAGACTGTAGAGCATAAAATGAGGGCAATATTGGTATGATGTCGCACCGAAACTTACAAAACCGTGTATGCGAAAGC

>TU2967-GaLu96scf_20-274891-275162 CGAGGCTAAATAGTCCAGGCTGGTAAAGGGCAGATGCCGACTTGAGTTCAAAGCGCCAGCAAGCAGAACGTCAGCATCCACGAGGTTGTCCAAAGCAGAGTCCGAGTCCGCAATCTAGAGGCGAGATAAATAATAGTATCAGTAGGCTCAGGCTCCTAATACTAGATAAATGCCTATCGTCAACGCGAACCACACATTGTGCAAACGCGCCGTTCTTTCGGGAGCCCATTACTGGCTTGCTCATAGGCACTTTTGAATATGCACGACGAAAA

>TU2968-GaLu96scf_20-275281-275861 GCACGAAGCAGGGGGCGTGGCCCATAGAATACAGCGTGTGGTAGGTGAGGCTTGCAACCAGTCCCGGTGGGGGAGGTGGCGTTGTGGATCCAACTGACATACATATGCATGGACATACGTGGGGATCATCGGCGAAGCTTTCGGCCTCTCTACGGCATCCAAGCCTCTGCGGGTCATCAGTGCATCAGCGATCACCTCCTGGTGGGAGAGAGGCCCGTCTGTGAGGCAATATTAAAATGTACAGCCTGAATCATAAATCACCAACACTAGCTGGGTGCGAATGACGACGTGTGCACCCGAAGGTCTACAGAACTAGGGACCCACCGTATGAGCGCAGATCAGACCACATGCCATTCTCAAGCCCGGCACCAGAAGCATTACGCCTAGTATGTAGGACCAGGCCCGCCTTGGGACGTCGATGATGTCTCGGGGTAGGATGCGCGGTTAAGGTCTAAAGCACCCTCCGCTGTTCTTGCAATCTACAAACCCTATGCGTGTCTGCAATCAGGCGATGTCGATGTGCCTGAATGTCGACCTCTAAAGCATGGTCTGGGTGCGAGACTCCTTCACGTCGGAGGGGG

>TU2974-GaLu96scf_20-294046-295079 ACTTCTCACTGCCTGCATGCCTCACAACACAACTTAGAAAGCGGCTACCTTCCTTCTTGCTCCATCTTGGGGATGTCATCTCCGCATCACTGTGACCCATTCAAAACTCTAGTCGGAACCTACTGTGTTCAATGGCCTGCTCGCTTTGAAGTTACGACGCCATTCTTGCGCTCACGATCACCAAGTTTTCTTAACCTCTCTCTATTCTGATCATGGCTCAGACAACCAAACCTCCTGAAGACTTTACTCGTGACCCGTCGAATTTGCACGAAGGGAAAGGTTTCAGGGGACCAGATGGATGGAGTAAGGTAGTTCATTTCAGTGAGTTCGCCCGACCTTCTCTTGCTCTTGCCCCCGACGTCTTGATCGTCTGTGAAGATTCCTTGAGCACATGCATGTCAGGGTTTGCGATGGCGGTAGATTGGGGTCTTCACATTCGCTTTCACGGCACGGTGTGCCATCCGCTTCCTGCCAACGCCTGAGTTACGGGCGAGCCCGTCCAGAGTCTAATGGACCTCTGAGGGGTTTCGACGAGCAAGACCCATCGTAACGTGTAACATATGCTCAAAAATCCTCTCGATATCTGGTCCCCTTCGATTTTCGTTCGAGTTCTTGGACTTTCACCGGAGAAACTGAGGGTGGCTTCCATCTTATCCCTCCTCCTCTACTGACTACTCTTCCTATATCAGAATCCTATTACCCCGCCTGCGTCCTCATTGTTCCCTCCGCTACCCCTGTCGCGGGCCATGGATACCGTCGTTTGACACCAACATCTTCCTTAACTACACTTCCAAGGCCGTCCCAGTTGTGACCTTTCGCGACCGGCACTGCAAAAGACAGACGTGGGGTGGGCTGCGCTCGTGGACATCTGCCGTTACCGGGCTCGAAAAGAAGATCGTAGCGGCAAAGTACCATCGAAGCTTGCATGTTCCGAGTCGATTGCGATTGTTCTGTGTAACTTCTGCTTCTGGTGCTCAGTCTCGTGGGAAGTATCGATAGAACCCTATTGCACACACATATATATATACCCGGCA

>TU2980-GaLu96scf_20-326268-326533 TCGATCGGATGAGAATGCAAGGTTGGACTCAGCCAACGCATACACGTCGCGGGGGAGGGATGGGGTTCAGACCTCGAGGCTTCTTGGAATCTTGCTCCGGTGGGAGGACACGACGGCCTGGTCCCAGGCGATAGTCTAAGCGGCTGGCCCGCAGGGTAGATGCTGCAACGTAGGTCTTCGACGTTACGCAGCGGTCTGTACGCGTAAGCACACGCGCTCCAACCCGCCGAGCACGGTGTCCAGGTTTCGTATGAGCGAAAGGTTCC

>TU3004-GaLu96scf_20-476285-476514 CAAGCGCCTAAGCCGTAAGTTGTTCGAGAGGCGCTAGCAAGCGGCCTCTCGCAACTTCGCCCATCCTCCTGGCACCATTCCCCCGCTGCCTGGCCAGCACTTGGCAGGGTGCAAGGGGACCCTATCCGAACCTATCAAAGCCTGTACCTCACCCGGAATGCCTCCCAAAATCTGGCCGCGGGGGAGGGCTGCCGGCCTTAAGCCTGTTAAGCTGTAAGCAATTTCCCGAA

>TU3014-GaLu96scf_20-496992-497423 TCCCCCGCTGTTGCGCCGCCTTCGCCTAGCTGTCCCGCTGACCGCAACCCTCTCCTGCAGACCGCCTGCGCACTGCCTAGCCGACGCCTAGCCGAAGCCCCCGCTGAGCCGAAGCCCCCGCTGAGCCGAAGCCCTCTGTCGCTCAGCGCACCCGCCGGAGCCCCAGCTGAGCCCAAAGCCTCTCCCGCTGAGCCCAAAGCCTAGCTCATCCCCCGCCCAGCCCGACGGCTTACGGACGCGACTCCCCCTCAACTGCCGCGCTTCCGTGTCCTCGTGAGGGCTTAGCCCGGCGACTTCGCGCTTTCGCCTCGCTGATGCGAAGATTAAGACCTCTGTAAAAGCTCACAGGTAAGTACTTACTGGCTGAGATGAGCCGTTTTCGCAAGTAACTAACGCAAAGGCTCACCCATGCTGATTGAAGTCCCTGTGTAC

>TU3037-GaLu96scf_20-829125-830052 AGTGACTGGATGTAAGTTAGGCAACTGATCAGAGATGATCCGCGATCGAGCAAAGAGCGGATGACTCTCCCCGCGCGCGGTGGCCTACCGGACGAAAACTGCCTACATGGCTCTTGAGCCGGATAGGCCGCTCGGGAGCTAGGGACTGTCTCAGAAACAGATATGTATTCTTCTGGGCAAAGTACGCTAGTAAGACCTTGCTCTTTTGGGGCTGATGTCAGCTTCTCGGGAGGCCTGACCATCGGTGGATGATACTTACCAGAAGCTGCGAGCCTGCGATGACGGTGTTCCGGTCCCCCAAGAAAGCCCTTCAAAGTCCTAGCCTTCTATATCTTTCAAGCAGGGCGATGGCGATCAAAGATAGAGTCCATAAGTCACAACCAGGAGACCCCATATTCGCAAAACGTACGCACCACGGCTCCCGGGAATGGGAAGAGTAAGGAGGCGGCAAGACGTCCCCCGCATCCGCGTCCAATATTCCATCTCCGACTCCGCGCCCTCCCGCCAACTTCACTCCTCCATCTCTTTCCTGCAGTGGATCCCTGCGATGTCCCGCCCGGTGGCTGTTATTGTCATTGGAGAAGACGGAGGCCCCAACGTCAACCGCGGCGATGTCCGCCCCGTTCGGACGGGGCCTCGGTCCCGGGTCGCCCGGCGGTCGAGCAACCCCGTGCCCGTCCCTGTCCCCGAGTAGTTCCCGGAACGCTTTCTCATCAATGGCGGATCCGTCTGTGCGCTCTCGCTGCGTATGAGTATGCCAGTGAGATGGGGGAGGAGTGCTGGGCTCGTAATCGGGTACACTCGAGGCCACGTAGGAGACGCGAGCGACCAACGAGGTTCCTGTGCGAAAGTTTAAGTTCAAGCAGCCGAGACTCGAGTTCGAGAACCGTGAACTTGAACGTCCTCGGCTGCGAATCGAACCGAACCG

>TU3039-GaLu96scf_20-831350-831948 AGACGTACCTTAAAGCCTAAAAAAGATGCAGCATAGACGCGATGTGCCGTGGATGCAGAGAACGCAGAGGGAGTGGAGGCTGGTAACGTTGTTGTAGCATGCTTCAGAAAGGCGGCGCAGGATCAGAGGGTAGGAAGGAGATAGCGATGTCGAGGCCTGTTTTAATGGGACACGACGGGTCTGGCGGAGGGCATAGTAGTATGAGTGAAGCCAGGGGAGAGGGAGGGTAGGGGGAAGATGGTGGATGGAGGAAAAGTCGCGTTGGGGGCCAAGACCGCGTCGCCCATGCCTATATTGGTAGTCGGATGCGGCGATGACATCATACCGTCGATGACAGGACTTACTAGCTTAAGTCCAGCATGATGTTGAGGTTCGTTGGGGTGCTCCGATTCGTTGGGGAGGGATGTGCCTCCCCACGGTTAACAAGTGCTCAGATGGGGACTTCGGCGGGAGGAGGGCGCGGAGTGGCATCCGAGTCAGTGAGTTCACGCGCACGTGAGTAAAACGACGGCGAATTTGATGGTGTTGACGGTGCACAAGGTAGGAGGTGGGTATCGAAGGCTGCCATGAAGGAAGAGGTGCTGAGGGAAAGGAGAGAG

>TU3044-GaLu96scf_20-873340-873846 AATTACTAATCATCCTGCTTAGCATGGAACACCACAATCAAAACATAACTTCGCTGACAAACCGGGTGCCGGTCAAGTAGGTTACGACGCGGTTCGTACATACATCCGACGTGGAAAGTGTGCGTTCACGGTATACTATAACGGAAGACAGCGAGATGGTATGACGACTTCTCTTTGCACTCTCATCCCTGCAAACCGATGTTCATGACTGACTTCAAGTGTCGATACAGTGCACGACCGCTGCGAGGACGTGACAGAACGTCCATCTCAACGGACTGACCTGGCAATGTAGGGAGCGGCCTGTACGCTGCCGGTAAGCTACTCCTGAGGAGACCATAACACTGCGAGACGTACCTGATACGGACGCGGTTCTCTGGCGATGGGTCAAACCTTTCTCGTGAATGGTTGAATACGAAGATGTCGAGCGTGAACTAGTCGAGTGGGCACAGCCCACCCACATGCAAAATTCAGTATCCCTTCTCCGTGAGATAAGGCGACGGAAGAAGG

>TU3045-GaLu96scf_20-873998-874359 GGACAGTCAGTCAACGGATAGATTGGAGGAGGCATAGGTGGGCCCGTTTCATAGGGCGTAGGTCGGGGTTAAGGAGAAGGCGAAGGCTCGTTTGGAAGGGCACGACCAGTGAGATTCATAGGGTGTAAAGTAGGGGGGGTTTAGGGTTAGAGTACGGTTTGGGTCAGACTGTTGCAGAATTATGCGAGGTGGCAGGGCACACTGCGACACCGGGTGGTTGTCGGCTGGGTGGGTAGCGATAAAAGTGCGGGGACACAGAAGGCCTGTCTCCTCGCAACCTATGAGTATGGACATGTGAGTAGCAGATTTTGCGATGTGGCAGCCAGACTGTGTCATCGCATGATGGTCCAGCAGCCTGTGCG

>TU3087-GaLu96scf_21-148982-149478 TCGAGCACTGACAAGACGTTTTTCGCCCAACAGTTTCACAACACAATCTCACTCAAAACGGGGGGTCGCCCGATTGCTTCAGAGTGGGTGCAGAGCGAAGTATGTAAAAGCATTCTGTCAGAACTGGTGACTAACTTAGTTCATCGATAAAGACATTTTAGCAGCATCCCACGTCTCCTTCCCCGTACACCATTGCTTTCTGAAGACTTAACTGATGAACATTTACTTGAAGTTTGGAACAGCTAGCAACCCGTGCATGAAACTTGACGGTCAGTTTTGTCAATGCCCCTTGCGCGCTCCGCCATTGACTCAACGCTACGGCTCCCTACGACTCAGGCCGCTCACGTAGACTAGCAGATTTGGTGCGCAGTCAATTGTATCTTTACGCGCCACCAGCCGCCCGCCAAGTACGCTGGGCGAAATCACGACACTCCCAGTCATTGGTTGCTAGGTCCACAACCTTCCCGCGGTTTTACGTCCTGTGGCAAGGAGCTGCG

>TU3095-GaLu96scf_21-188035-188249 ACGGCACCGTTTGAGTGTTTCCCCCAGGGGGAGGCCAGTTACACCACGAGCAGTTACTGTTGTCGAAAAGGATTTCGGCGTCTACAGTTCGTTGTCCTCCTGTGTAAGTTATGAATGCAAAGGGCGGCACTATGTCTGCCTCCAAATCGTTAGCGGAGGATTTCGGAGGGCGGATGTAAAGACGAGTTGGTGGAGGCGGGAAATCAAATTAATCA

>TU3114-GaLu96scf_21-295298-295997 GTCAGGTACGTGGGGTTACTAATTCATATTGTATCTCGCCTCTTTCTCGCCATTGTGGTTTGTAGGCAATCTCTATACTGAGGCAGCACGGATCCTCTTACGCTTGCAAACGAACAACAAAATAATAATCTGCTTCAGCCGTACAACCCACCTATACGGGCTTGAGTTGCTAACGGCTCTGTGACATTTTGCGAACCTAACGAGGACTTGCAGTAACATGCCTCCACGCTGGAGAAAGTTCATCGTGCAAGGGTCTTGAGCCAATCCTTCAAACAGATGCCAGCGGTAGCGACAAATAGCTCACATATAAGGACGTTACAAGATTCGGTTCATCACCGATAGGTTCAAAATCAAGACAACATGATCGCGGTTTGGCAAAACATTTCCCTCATTTCGCCCCGGTGGTGCCTTGTTCCTAGTAGCAAGCACCGCACCATCTGCAGTTGCGCATGCGCGCAGCATGCCTCCAGAGATGATGGTAGCTGAGGGCTACGTAGTCGGCGCTGTAAGTATTTCCCTCGTCGGCAGTGTTCTCCGTATTTTTCGCATCACTAACCGTAGCATCGCAGGGGGCCTGCTCATACTTCCTGCGCATCGATCTACATCGTGGCAGTGTCCTGGTATATAACTCGCGGAGACTGTCGGGTACAGTAAGGAAGCGATGGACAAATTGTAGCGCTGCGGTGTGTCAGCTCTTTAA

>TU3144-GaLu96scf_21-427246-427718 CTCTCGGCCACCAATCGCACACATCCATGATGTTTCGAATCATACTTACAAGGTCTCGGGTCCTTCGGGAGGACTTTTCCTCGCGATGTGCCTCCAGTTCGGCACGAACTCCGGTTCCACCTCCTGGGGCTCTGCGTGGGTCAAGGGTTTTGCGGGATGTAGGACGAACCCATGATCATCAAAGGTTGCCACCGAGAGGCGCAACCTGGCGCAACGTTATGTGAACCGCACGACGGAATAGTGAGAACACCTTTAGCGTGGGCACCACAGTGTGTTTATGACTCTTTTTACGTTTGCTGCTCTTTGGATTGACGCATGAGTACGTACATAAGTTCCCGGAGACCCACAACACAACACAATCATGGCAGATCATGGCCGATGCCTGCGCCTCGTGGGAGTCTCTCCCTTCGATATGGGGCATGGTCCATCACGAGGGCCTGGAGCGGACCACACCGGTCATTTTGGTCGTAAGA

>TU3147-GaLu96scf_21-436719-437241 TACGTTGCAGTTAATAGAGCCATACAATGAGTATCTTATGTAATACTAACACCGGAACGCAGGCTTAAGCTTGGCTTAAGCCGGCTTAAGCCTGGCTAAGCCGGACGGCTTAAGACATGGCGGGAGGCTTAATATCAAGTAACTACTGGCTGAGATGAGCCGTTTTCGCAAGTAACTACTGGAAAAAGATTATCTATTCTGGTTGAAACTGGTGACTATTTTGAACTGCCTTGTCCCTACATCCGTACACGTTTTCGCTATACTTATCAAGTACGCCCATTCCCCCTTGTTTTCAATCTGTTTCTGCTCTTCTGGCCTCACTCGACGTTCTTATGGCTTTACCAGCTTTGCCACATGCCGTTACGGTGAGTCACTCTGAGCTGACTATTGTGCCCTTTGATCCTACTAAGCTTATCAACTTCTATTTAGAACTTCATGACATGCAATGACTACGCTTGTTACTCCGGCTGCTGTCCTGTACCCGTCCGACATTCATGTCGCGCGGGGAATGAATATTTACGTC

>TU3148-GaLu96scf_21-438119-438611 GCGTGGCCCTCCCAGCGCACATGGTGCCCACGAGTCGCGGTCGCAAGGCCATGATGGCATGTTCCGCCGGCACCGTGACCGTAAGCTACTAAGTGGCTACTCTCCTTCACTGGTAAGTACTGGTACAGTAGCATGCTTACATACTTATCGACAAGAATCAGAATGTTAGGAAAGCGGCCCCGCGCACATCGCACCAATCACTGTGCGGTCACGGTGGTGGTACCATGTCGCACCAACACCAGAAGGCCCGATCGTTACGTCGATCCCGGCTCGAATTCGGAAATACCGTATTGACGAATATTTGGCTCGAAAGCGAGGACCCCGCTGGATTTCCGGCGTTCTCTAGTCTGGGTCTGGGGCTCCGACAGTGCTCCGACATGACGGCGACATCGAAAGGTCTACAAAAATATTACAGTGATATTCGTGTACATGTTTCAACTTCAGCTAAGTAGTACAGCTAGTACCCAGATCGCTGGTCCATGCCATCGGCCAG

>TU3150-GaLu96scf_21-446980-447803 CGAGCGAATGCGAGCTAGAGCACTACCCATTGACGCTGCGCGCCCACAATGGGCATCTGGAACCTGAAATCGGGGTGGTCCTTACGCCTGAGCACGACGTATGCGGCCTCGGGGATGATCCACTTCTCCTCACCCCCAGGGAATCCCCAGGTTCGTTCGGGGGTCGTTTGGTTGGGGCTTGGCCTGGGACGCGTCCGCCGTCTGGATTAAAGATTTCGCCAGTGGGGAAAAACGAGGAAAGGAGAGGAGAGAGGAGATGGGGTGGGATATAAGTAATGACTACGTGATACGGCCGAGGAACGGGAACGTACGCCGATGACCCGAAAGCCCCCTTTGTCAATGATCTCCTGCGCCATCCGGTTGTGAGCCCGGAGGTGGACGTCGAGCGTCTCTTCGATGGCGCCCATCGTGAACAAGAATTCGCGGAGCTCCGCCTAGGTCGAGAGCTTATACGATGGTGCTATGCTCATCCGGTGCTCATCAAGCTAGCAGAGGGCTACTGGTGCCTGGTTGCTCAACTGAGACTCTTGGGGCTCTCGATAAACCTTGAGGGGGTGCGCCGCTGGCACTTGTTGGGGGAAGGAGCCTGAGGCAGAGGTGGTGGGCTGCACGGAAGGAGGATCGTTGTCAGCTCAGGCTCTCAAAACTGGGGAGGAGAAAGACCGCCGACTCGGACTAGTGGCTGGGATGACATGTGAAGAGAGTGTGGTAAAAGAAGGCCCAAGAGGATGGAGTAACATTAATAATATTAAGTGGTAGTGGCTGCCGTAACTCCAAGCTTTTGAAGTTCGAGTTGCCAGGGTTGTCACTTGTACGTTCAACGT

>TU3153-GaLu96scf_21-478424-478963 GGAGTTGAGCACGCGCCCAGGCGCCGTAATCCTGACGCAGTCGCCGCTGCCGCGGCTCACGTGTGGACGGCCCCTTCCTAGCAGACGGCGGCGCATCAGGGGCTCGCTGTTTCCCTCTCGCGCTCATCTCGTCGTTTGCTAGGTGAGCGTACACCCCCGTCTGTCCTGCGTCGTTTTCCTCTTGCTCACCTTGTTGTTTGCTAGCTCCTCGGACGCGCTCTTCGTTCTTCGCTTACCCACCTTCCCGCCCTTGTGCTAACTTGCATTTTTCTATGTAAACCAAACTTTCTGTATCATTACTTGTACAACACCGCTCGTTACGTTACTTCCCTCCGAGTGTCCCAGTCCCTGTGGCCTGTACGATATTCCGGTAGGCCCCAAGCAGTGTACCATAGTTTCTTCCCTTCGCTCGTGTCCTCCGAGTCCCTGGCCACTCTGTACGATAACTGGTCTCCTTGCTTCAACTTGCTACCACTCATGTATACTGTAGCGACTGTGCCCCGAAAAATGCAAAGGACGCCCCTCGCGGCCACGCCGCGG

>TU3195-GaLu96scf_22-54427-55118 CACGCAGAATTTGGAGCCCAGCGCTCAGATGTGGGTTTCGGGGATGGTCCATACGGGGCATGGTGTGCGCGTTAGCCACCCGATCTCCTCGTCTGAGGCCCGGCCAAGCTGATAAAGGTGATGTGAGAGCTTCGCCTTGGAGTGCGTGGAGGGAGTGAAACACTTCCTGAGCTTCTGGGGTCAATGATGGGAGCGTACGAGAAGTTCAAGTTGGAGCCGTAGTCTGTCTGGCGCTGTGTGTCGGACTGCCTGAGATCGGGGTGGATGTTCACCAAAGGACGGTGATGGGGGGCGCCGTCAACTCTGTGTTACCTTGCGAAAACAGGTAGGTGGGAGGCCGCGGCTCAGCTGGTGGCGAAAGAACCGGGAACGGTCAGCACTATGTTTGATAAAAGATTAATCATCTACGTACCTCTGGCAACATGTTTTGTACTGTGCGTGTCAGAGACTTTAAAGTAGGTAAGGCTCATCCAAGTAGATGGCTATACCTAGTGACCGCACCAACGCATCCGAGTCGAGGACGATCGATACCGGCTGGAAGCCAGTGCGAGTGAGTCACTTACCGATGGTGTGCTTGGGACACTAGAATTCGGAGGGGGGACGAACGCCCTGATGGTGACCGTCAAGTCCGATCGCGAGCCCACCAAGTTTCATGTCCGACTTCTGTTCTCCACCAAGACTGCGGCATCCAC

>TU3196-GaLu96scf_22-55195-55454 TGGGTTAACTAGGGTCAGCTCCTAGGGGTGACAGACGGTGGGAAGCATGTGCCGAGAGGGCCTGTTGCCCAAGGTCGGGGTCTTGTCCAGCCGGGAGCTTCAGCGGGAGAAGGTTCAAGTTGGATGGGAGCGATAAGTTCCGAGTCAGATCTGGAGCAGACTAGAAGAGAGGGTACGCCGCACCAAAGATGCGCGCTGAACAGGTTGCAGAAGATATCGGCAACTGCAGCCGTCCACTCCAACGCGCAAGCAGTCCGTCC

>TU3199-GaLu96scf_22-149203-149944 TCTGTTCTGACTTCACACTGTCCGGTGGTTTCCATGAGCCCCATCTATCCTGCGGGCGGTATAAGAGCTGGGCAAATGTATCCACTGGTCCGTACGTTGGACGCCTGCGGGTTACTAGTAGAAGCGCCTTCAAGCTCGGCTGCCGTGGCCGCCTCCTCGGACTCAGCCCAATTCATCTCCTCCGTCTGCTCTATTCCTCAATGAACTCAAGGTCTCAGGAACACGATCTTCGATTATTCGCGCCAATTGTGTATCCTTTAGGTGAATACCGCCCTCCAAAGCCTTGGATTAATGGTTCGGCTAGGTAAGTATAACAAGGCCCGTATCCTATCCGAACTTCTCCCCATGCACACTCATAGTGACTAATCACGGATGCCGTCCTTTTCGGAGGGACGACCATCGCCACCCGGCCCCGTCATCACAATTCCGTCTGGGTGGGCAGCCTCCACCGGCCCTGCGTGTCCATACGGTGAAGAAGCAAGCAAGCGTTCTGATTGCATTTACAGCGTGAATCTGTCGTCGAATCGATAGAGCAGGTACCCTGAAGGCCAGTGGAGGGATCGCATGATCCCGCTTTTAGATCCATGCTCATCGGCCGTTGTCTAGCGCAGACGTCATTCTTTGGTCAAATTGGACATTGCCGTATCACAAGAGCACTTCACGGAGCCTCACCTTGCGTTGCACTTCTCTTCCAGAATGGCTGCCCAGTTTGATGCCACCGCTGAACAAGTAATAGCCCTCT

>TU3203-GaLu96scf_22-157497-157974 CAGCCCACCGGAGTTTGTCCGACCACGTAACTTGTTACCGGACGAACATGAAAGAAGACCAGGAAAAATCACATACATGATACTGTATCTTACCGACAAAATTTAGTGAGCAGGAACAGACCGTGGGGTTAGATGCTTGCGACATTTAGACCTTCCCTCGCGTGAGGTGGCGTGCTCAATGGGAGGCGCTGGAGAATTGGTCACCTAGATATAATACAGTAAACGCTGATCGTGCGGTCATCAGTTACTTGACGCAAGACGAATGTGCAGTATATGTACTTACAGAGCAAGCCGCACTGACCACGAACGGAAGCAGCAAGCAGGTGCGATGCGCTTGTAGTGCCGAATCGGCAACCTCATTCGCTGATCCAACCCGCCGATAGCGTCGCTGCGATGCTTCTATCCTTCTGCAACTTGGAGGCTTGCGCGAGTATGAAGCCGGTAAGACTAGTCCTAAGTACAGCATAGGCCGGTGTAG

>TU3316-GaLu96scf_23-502816-503371 CAAGCTTCATCCATAGCCACTCGGCGGGAGAGGTTGCGCCCGCAGTTAGAACATACGCAGCGTGAAATCCTAGTCCAGAGGATAGTCACTATCTCACACCCCGCGGTCGGCCAGCCTCTGTTCTGTTTTCCCGCCTTCTCCCAGCCCGATCCCGCATTTCTCAACGACTCTTCCAAAACGCTCTTTGGCGTATGCCACCGTAGCTCTCGATGCCTGTCGTGTTGTCACAAATGATGCTACCAAAAATTCGGAAATTTTCCTTGCACTGGACCGTGGTGGCAAAAGTCCCATTCCCATCGACGACGCCAAACCCCTATCGCCCGGTGTCTACTACTACTTTCTTGGTCCTCCTGGGGAATCCGACATTTCCAACTACCCAATCGTCAAAAAGTTCTCCGCGTTCAAGTTTCCTAGTCAGTTACCTAACAACTGGTACAGCGCCCGACGCAGCTTCAAGGGGGACGTGCGGGACATCAGGAAGGTCTCCGGAACAGAGATGTCTCACTTTGTGACGGAGCGGGACAAATACCGCATCCTTAGTGGATGGACTCACTGT

>TU3317-GaLu96scf_23-503527-504186 CTCCCTCAAACTGATCCCCCGTTCAGTCGCTCCCAGAGGAATCGTATGTTGACGGCGACGAATGGTGGAAGGACGGCTGCTCAGACGCATTCCGGAAATGGGTATGCCCAGTTCCTTGCACCTCTTCCCTTTTCTATTTCCTGGGCCTTACTTACTGCATGAACTCATCCTCCCTCCATCCAGGTGCCCTCCGAGTCGAGGAAGTCGAGGAAGTCGAGGATTCGCCCGATATTGTCGCCTGCTGCACGGAGCCTATCCCGCACATGCTACGGCGTATGTCCAAGTATATGAAGGAAAATCCGTAGGTGTGGCAGGCCTCGACGACCCCTGAAGGCGCGACACCGGATGACAAGAAGAACTGGTACGCCAAGTGGATGACCCGGCCATTTTAGCGGTACGTGTGGCGGAGGTTAGCGAACCTGAAAGGAGCGTTGGGCGCATTTGCCAAGTCACTTCAACTCATCCTCTGCTTCGTCGACGCCGTCATCTTGCTGCGTTCCATGCTGTTCGGGTTTACGGTCCTTGCACCTGTTCTGGTCACGCTTTGTGGCTCGTTTGTGTTGTATCACGCCGCTCATTTGCAACGGCGACTCGAGCTTGAGGGAGGATATAACGCCGATATCACCATAGATAGTAGCCTTCTTCTCTCGCAAACGTCGT

>TU3321-GaLu96scf_23-534258-535072 CCGGGAAGTGGTGGGATGATCGGAGAATATGTCGTCATGAAGCGGCCGGTGCGGGTGAGCGTAATTTCGATGCGTTCTCCCGCTAGAAACACAAAGCTAAATCCTACCGCGATGCTCGATGACGGACAGTAGAAGCGTCGACGTCGCTGTCCTCGAGGTTAAACAATAGCGGCTTTCCGAACATTCTCCCGCTGCTGGGAAGCGTTTGAGCACGGCTGGAAACAACGTCCTCCTTCTGTGTAATATCCTCCGTTTATTTGACTAGGAGGTAGTCTAACATGGAAGCAAAATTCCAGGAATCAATGTTCACGGTAATTTGACGTGAGGCGGCGTCCGAGTGGAGTCCGCGTGCCAACGTCCACCCACAGGCTCGACACGTGAGGGGGACTCGGAGGCTTTTCATCCGCGTGCCTTGCAACTGCAAGTGTCATAACGAAAGCCAGCGCGCAGGCCGTCCCCTCAACTCCCCAATTTAGTTGCGCCCGAGCGACGCCGGCAGTCATCTGGAGTAGGGTCATAATTTCAAGGTATAGGTCAGGGTCATTGCAAGAGGGGACTGCGTATATGTACTGTTACATCACGTCCTGGCGGCGTTAGGCTGCCAATATCCCCAGGTCAAATGTACAATAACACCTCGCTTTCGCCGCGGATGATGGGGTCAATGATACCTACGACTCGGACGCTGCAAGCAAATGCATCAATGTTGCGTTTGAAAGGGTGTCGGAGGTGGTGGCGGAAATTAAACCCTTTCTTGCAATCCCCCAAAATCGGAAAGCGTTCCGCAAAGCACGGTGTCAACAAAGGCTTTCAATGTC

>TU3327-GaLu96scf_23-566816-567243 TCCCTGACCCCTCCAAGTTCTCGAGCTCTGTACTTCTTCCGACTATTCGCAACATGCAGAAGTGCTGCAGCTCGCAGCGCTTAGCCTTATCTCTGTCTGACGGATTGCGTCGTTTATATCGCGCGAACCTCCTCTAGACTCCGGTCGGCTGTTTTCAACCACTACGTTCCTACATACCGCTTCAGAGCCGCAGAACAAGACGCTTGCAATCAATCTGCTGTAATATGACGCCTTTTTACCAGAGTTGGGCGGAGTCATCTGCCAACCACCGGTGATTGTCTTGTCCAAGGTTTGCTTCTAGCCTCTGTCCATCGTCTTCGGGACAGTACTCACATCCTCGTTATCACCGAATCGGTGTGACCTTGCTCTGGACTTGTTCTGGCTGCTGGATATGTGCACTGATTTCACGCGCACAATGTCCTTGAATC

>TU3338-GaLu96scf_23-682597-683011 TTCAAGCATCGCGGGTCCTATCTTCTCAACGGGTCCTAAACCATACTGTTCGCGCAACTAGTGAGTTATGTTAATTTATGCTTACTCTTCACCCCCTGGCTCGAGTTAAGTTTCACAAGAATTTCCCTGATTAATTGGCCCAGTGAGTGGCGAGGCGTCTCCGCCGGTACTTGAGGAGCTCGGCAACTACGTTGCGAGGAGAATCATAGAGGCCGAGGATTTGGGCTTGCCTGAAGATTTGGAGGTACGTTTCCCGGGTATGATCCCATGAAAAAGTGTACTGATCTGCCACGGCCTTCACTGGCACTGGGGAAAATGCAAGGCGTCTCAATTGCAGGATGTATTGATACCCATACGGCTCCGACAGGCCAGAAAAATTTTTGTGCTCTGTCTCCTGCGTTCAATATGACCTGGA

>TU3343-GaLu96scf_23-696533-697099 CCTTACGCGTTTGCATCAGTGTTCAAACGCGACCCGCTTTCTCCCCCGTCTTCGCTGCTCCCGCGCGCCCGTTCCTTCCCACACAAACGCGTCACCTCTGCCCGAAGGTTAATTCGTGCTCGACCCGGTGGCGGCGAAGCCATCACGACGACGATGACGACCCGGTAGTGTTCGAGGCTGCCCCGCGACTACGACGACCAGGTGGCGTACGAGGCCATCACGCGACGAACCCGACCCCGGTGGCGCACGAGGCCACTACGACGACTCGGACGATCCGACCCCACGTAAACGATGAACCCGAACCGCCGGTGGCGTACCGGTACGAGGCCGCTGCGCGACGAACCCGAACACGGTAGCGTACGAGGCTACTACGATGACGAGCCTGACCAGTGGCGTACGAGGTCACTGCGACGACGACCGTCTGCCCGGTGGGGCTGGAGCCACCCGATGATCACGACGATCGTTAAACGTCGACGACCCCCAACCGGAACGCGACACGTACCGCATCCCGTACCCCGCATCCCTCGATCCTTGCCTACGTGTCCCCGCGATGTCCACTGTCCTTGC

>TU3409-GaLu96scf_24-557948-558644 CCCCCAGCCACGAAGTTCGCCCCAGCCTGTCGTCCGCTCAATGTGTTCATCGAAATCGCCGAGTAGCAGGCTACAGACGGTACCCCTGCGTACTCGTCTCTCTTGCTTTGTCCTGCGCTGTCTCGTCCCGCATACATCCAGCGCGCCCTACTCCTCCATCGCTCACCTCAGCGCAGGACTCCATTTCCGGTGCCCGGCGCACGCTCACCGACGCGTTCGCGCGCGCCTTTGCCGTTCGGTTACGTAGCTATATATCCTTGCATGCAATCTTCCACACCAAGGCCTCAAGGTCACGTCATCGCAGCTCACCACGTCCGGCGTCCGGCTTCCAGCCCCCGCTCACCGTCACGATCTCGCATGGGCTGTTAGCCGTTCATGGTCACTACCAAGGTGCCGGTCGAGTTAACGTTCTCATGCACGATCGCGCTCGGGGACTAGACTTCGAAGGCCGGGACCCACGTGGGAGGAAGTCGGTGGCCCGTCCCGCAGCCGCAGCCACGAATAAAGACGTCCGTTGTACGACGGGACTCGTGGCTCCATGATTGCTCCATGTCCTTCGAAGTTGCGTGCGTAGGCGTGCGTGCATGCGGCCGTCCGGCGCACTACCCCTGCGCCCCCTACCCCCCCGTTCCTGTTCGCGATGGCTCTGCCGGCCCGGAGGGCGATGATGCCTGTGCCTTCGTTTACAGTATTTTCA

>TU3410-GaLu96scf_24-559234-559998 ACGGTATTATCTACCATCGAGGGCGATGGATATCCTGAACGGCACCCCGTGGTAACTACTGATACGCGCGTGAGCATAGGCATCAATATTCGATCAATGCCAAATGGCCATGAGGCTCGGGTGACGGCGGCCCGGGGGCCCTTACACCCCTTATCGGACACTAAGGATCCGGATGTATCTTCAACTTGTGAACTGCGACCCTACTCTGTACACGGTGAAAAGGTAGTTCCAATATATAGCTGCAGGGAGCACGACCTCACCGCGATGATACACCATCTGCATGTATGCGTCGCCATCTGTCTTTCGACGAACTGTAGTATGCACCCTTGTTATAGGCTGGCGGCGGTCGTAGGAATCGGTGCCTATCGCAGCTCGCTGTAGGTCAAGGCGAGGCGTACCGAAAGGAAATCGCAGGGCTCTCCCCCCCTTTTTGATAGCCCGAACGCCATTCTCGCGCAGATGGACCAGCCGTGCGCACCGGGCGGAGGGGTTGACGAGGGAAGTGGCACGGGGAGCGTCGAGCGAGCGAGGCTGCGGGCGCGAGAGGAGGGGGAAAGAAGGATGGATGGGGGAGAAAAACTCGCTCTCCGATCTGGGATCTGGACGATTCTCCTTCAAGCCAGTCCAAGACGCGCTCGAGACGCCGGTTCCGTCCGCACCTAAGGTAATCGAATTAGGAAACTCAGACGCTCAGGCCGATCCGGACAAGTCGGGCCGGTCCCGTTGCTCATTCAACGGTTCAACCAAGTATGCCATCGGTTCGTA

>TU3414-GaLu96scf_24-584218-584508 CTCTGGAGTTGCTTGTCCGTGCTGTGCTAGGAGATCATACGTGCTAGTTCGTCCTGGTTCTAGTCATACACGACGTTGTATTGCTCTCTGCATTGAGACGACGTAAATTGCTTCCGAGGCCGCTGAGCTCTCTTTGTAAATATTGCCGCCTCTGTCTGTGTCTACTGGCAATCAAACATCAGTCTCTACGTGGCCTTGTCCGAGCGGGTCAGGTCTGCAAGGAACGTTCTTGAACTCTGGACCTGGTGTCTCAACTGCTTTTCGGTCTATTTCCTCCTCATGGATCGTCGG

>TU3417-GaLu96scf_24-590070-590515 TACCGGCATTTTCATCGATTTCGATGAGTGTTTCATCATGTCATGACCTCCAACCCAGCCATTATGTCATCTGGTACACCAGCCGGCTAGCCATGGTGTCAGTGCCGGAAGGACAAAAATGCATTACTAGAAGGGAGAAGCTCACCACAAGGCTAAACTATTGCCGTAGTTGTGAGGCTGAGGGTAACGGCAATCGTCAATTCCTTCCATGGCAGCCTAACACTCAGAACAACTTCAGTAGCCGTTCTAGACTCTGCCTGTCAATTGCAATCGTCTAATTTCGCGACGGACGTACCACTGGCATGTAAAGTTTCCACCATCAAGTGCAACAATGCTTGCGCCAGTCTAACCTGAGCAATAATCACCATTCTGAAAGCGAGCGAACTGGGACATTTCGGTGTGTCTGTGATGCTACTCGTAGAAGAACTCAAGAAGGCAGAGTGAAG

>TU3423-GaLu96scf_24-627984-628385 GTTTGGAGCGCATGATGCGCATCCCAGATTTTCTCATGCTACTTGGCGAACGCACAGAGTGGTCGTCTAGTCCTCCCAGATTGCGCTCAGATTGTCTCTCCATCGGGTTGTCGCAAACACCTCTCCCATTGTTGTTCATGCGGAACTAAGTTTCGCAAAACCTCCTGTGGAAAGATGAAGACCCTCGAAAATCTGGGTAGTGAAAGATTTACTAGGGGGCCCGGGCGTCCGACGAGTCGTAGGGCAGCATTGGAGGCATGAGAGCAGAGGGTTTTGTTGGAGCAAGCGAAAAAACGTCGTCCAAGTGAAGCACAGAGTATATTCCGGTTGTCCTGTACGGGGTCGTCGTGGTACAGAGGCAAACGGATGGGTCCGAAAATGTACAACGGCGGCTCGTATCAG

>TU3426-GaLu96scf_24-631824-632627 TCCTCCATCCACGTCGATCTCGTCTTGACTCTATCACTGGCTTTCCCTCTCCAGCGACTAGTCTGCTGTCTTGCTGGCTGGGTCTGAGAGGTATGTGTTTTTATTTTCTGCCTGACATACTAAGCTTGGTCCGGTTTGAGTGTTTCATAGGTCGTATTGGTGCGTTTGCCGCCAGCAGCTCTGGCAGATTCGGCCATATATTTCTGTCATATCGCTGTTGATGCTGACTCTAATGCCTGACAAGGACACGTACCTATCCCCATCGCCTTCGAGTCGACATTGTGTGTGGTGCGCGGGCGTCACCGTCCATTGCGCGTGTTACGGCCACTATCAACGGCACTCTCGGCGGTGTTTGCAACAGCGGTCTTCTTCGCCTGATACGTGGCCCCTTCCTCGGGCTCCATTTCACTTCCATACTACGGTATGTCTTGTAGCCTGTTTCTCTCCACTCTCATGCTCGCCCCAACCTTTGTCTTGATTCTGGGTCATTTGATGACCATGCATGTGTTTGTCCCCAGCTTCTTATTCACCGGCTACGCAATACCCTGCCTGAAGAGTCACTGCCCTGCGCCGGGCCCCGGCGTACTCCACGCAATGTGCTTCATCAGCCGGGTACGTCCATTCATTACTTAGATGGTACACTGCCCCTTGGATGTAAGCATTGTGAGTAGCCTCCCTCGCAATGTCTTCGTTACTGTGTGTGCTGACGGATATTTCACGCTAGTGTTTGCACAATCATTGGCTTGCTTGCCATTGGTGGATAGGAGGTGACGGAGCTGAGTGGATTAGAAGGGGCTTGGTATA

>TU3432-GaLu96scf_24-651732-652168 TTATCACATATGAGTGCTTCGCAAGTTGTACATCCTTAGGATCGCTGGCGTTTATACGTATCAGGCTTCCAATGCTGTCTCGGCAGGCACGGTCTCTCAAAATTGCACCGAAATGCAACCCAAGGAGTCGCTCGCAGGCCAGGCAAGGGTCAACGGATAGCAGGCCCATAGAAATAAGGGGAGGAGCGAGTCCACAACTTCAGAGCCCACTGACAACTCCGAACGGTTGTAGAATAATTTATATGTTGTAGGGAAGCCTGGGTAGATGACATCGAAAAACGTCTGAGGAAACCGACAATCGCAACTCGAACACTGGCGCTCAGCCACAAAGCTCCGAATCCTCCGCGCAGCGGGTGCTATGGCCGCATGCCCCCCAGCAGCTCGACAGCGAGCCCGCACAGTGCCCGCACCCCAGGCGGACTCCTTCGACGGGGTCT

>TU3434-GaLu96scf_24-654763-655348 TTAGAGACGAAGAGGTCACCTGTTTCCTGGGAAAGGATGTCGATACTTTGGAAAAGGAGTGGCCGACTCTCAAACAGCCATTCCTGAGAAACAAGCGCGCAGTTCTGCAGCGTAGACTGGTCGTCGGAAAGGGAGCTGAGGATCTCTCCGACTATTTCTGGGGGTATTGTCGGAGTCAAGCTCTCTGCAGCCATGGCCCCATCCATCATCCAAAGTGTCTGAATCGTGATGAGCAAGTTGCAGGTCATCGGCCATAAAGGGAATGCTTATTGCTCACCAAAATACTCGAATATTGATTGCGAAATCGATGCTCGGATACACAAGCTCGATAGACGTGAATACTGCGACTCTCCGGGTGGGTTTATTCCGTTCACGGGTCGACGCTGACTCAACACTGATAGTCGGTCGAGAACAGAAGAGCGAGATAGCGACTCGAGGTTCGAGGAGACAGTCACACAGAAAGTAAGTAGTGATGCACTACGAATCGATGCCAGCCTGAGCGCAGAGCAAACACAGACAAACAGACTGAGAGACACAGTCCTCGTGCTTTCGAACCGGCGCAGTGGCGCCGGTCAGCTGAACGGTG

>TU3438-GaLu96scf_25-66-477 AAATGAATAGCAGAGGGATGGAGGATACGGCGAGGACGTGTGGGTGGACGGATGACTGTCAAAGTGCGATACATTCGACAAATCCATTAGCCGCCATATGGAACTGGACACAATGACTTGGAAGAGAACGCACTAGACTGTACACGTTGCGGTCAGGGGTCCGCGGTCGTGGCGTTAAAAACCGTGACACTTGAAAGAAGACGAAAAGGTTCAGCGTCGAGCACTTCGGACGATGCCATATACAACTGACGTAGGTGCCGATTAATGGGTAGGTCGCTCCGCGAGGGCAATTTGACCACGGCTCATCATCTTGTGATGACCTAGTCATAGTAAAGCATTAACAATGGCAGAAAGGGATTAAAGGAGATCCCAAACCTCACCAGATCGTCCGCAGAGATGAGAAGCGTAATTC

>TU3460-GaLu96scf_25-81502-82360 CGAACTCGGCGTGGTCCACGACGTGATATGCGCCGAGTCGAGCCGGCCCGACGGGACACTCCAGGCCGAGCAGTCAGGCTTCTGAAATGGTGCTGGTGGCTAGGATGTCTTTTTCCGTCCGCCGCCCGATTGCTGGAACGTCAGCGCGTCATCGCGGGCGTGGACTTGCTCTGGGAGTATGTTCCAGGTACGGGGCCGAGAAGTCCAGGGGATACAGAGACAGATAACGAGCTCCCGAAGACAGGGAAATGTCCGGACGATATGCCGAAGGCTTATTGACCAATTCGTGGGCCGAAAGGGCTCTAGTGCGCGAACGGAGGGAAGGGGGACCCAAAGAGGGCTGGCGAACTCGGGAGAATAGATGCGGCGAAAGACACGGAGGTGCGTTTGATGTTCTCCGAGGTTGTGCGGAAAGTCAGAGTTGACGGAAAAGTGAAACGCCTCCAAGGGGTCAAGCTGCGAGCGAAGGTCGAGCACGGTCTCTCCGACGAGATTGTTTATGGCTTTCTGGTAGGTAGAAAGCCGAAGGTGGTGGCGGAGGTGTTGGAGCTCGGAGATGATCGTCTTCAAGCGTGTGTCGCAGTCGGAGGTACCGGTGAATAAATGGTCCGTCCAATGTATCCGGAGGTCGCGCAGGTATGTTGCATGAGAGAGCACTTCGAGCAGGCCATTACGATCGAAATTGCGATCTCTCGAAGAGCTTCCTCATGCAAGAGGTGGGAGACGACAGCGACTTTGGTCAAATCAGCTACGGGAAGGTGCTTCAAGACTGTCCGGAACACATCGAGATTCAGGGCTGGCTTGGAATGTCCTTGACGGTACCCCAGGGCACTGAAACGAAGGCGACTCAACACAACGA

>TU3479-GaLu96scf_25-318907-319279 GATGCCAGTGGAGCCCCGCCCTCTCACGTGCACGGTTGGTTCCATGTCGTTGCCGAGACGATAGTTGGATGCCTCTCAAAGTTACGGTGTATGGCGGCCTGATGAGTGGTGGCTCATTATCTAAGGCCGCGATGTCGACGATTGGGCCCTGGCGTCGTCCTCGAGCTTCCAGTGCCCTTGATGAGATCGTTTGCATTCGGGATCGTGCTGCTGGAGATCTCGTACATCGAACGTCAAAGAACCAGAGCTCCTACGTGCATGTTGTACAATGAGTTCAAGCAGCAGACAGATAAAAATTGGAATTGGGGCAGCAGACGAGAATGGGATGCCACTTACTACCTGGTTGCTACACGTCGAAACCTCTGATCAACTA

>TU3482-GaLu96scf_25-325312-325993 ACAGTGTTCAGTGCAAAGCCCAACCGCAGTATTGGCACAGGGCTCCGGTGCGGGAGACAGAAAGACATATCGGAGGTGTATCGACCACCACAATCATACTATATGGGCAGCTGGTTGGAGCACGTTTGTAGATGACGGAAACTGACATTTACAAGTGTGCCAGCAGTATCGGGCTGGGGAAGGAGGTGGACGAACTGGGACGAGAAAGCATGACACATATAAGAGGATTTGAACTGGTGACGGTGCACAGAGAGGGTCGCGACGTGGCTTGAGATGGTGCTTGCTTTTGAGTCTTCGCAAAAGATGATACGGGGACGCAATGGTAACAGATAGCAGGGGTTTGGTGTAGAGGACGGCTCACCGACAGTTGATATTCAAAGCAACGCCGGAAATACCTCCCTGAGAACAGGCTAGCTACTCTCAAGCCAACACCCACAGCCAACAACCTCCGGTTGACTCAAGGCGCGTTGCAGCTACTCAGCGTGAGGAAAGAGGAAAGCGAGGTGTCGGCAACATGAGCGAAGGCAAACTTCGACTCTCGAGAGATAGGGCCCGGGGTTTTTGGTCTTGTGGCGAATATCGAGTGGGGGATACTGAGGATTGAGCCGTATCCCATTTTTGCTCGCGAAGAAGGCGCATATAGTAAGTGTCTGCGAGACTCGGGTTTCGTCCGTGTTGAGGT

>TU3507-GaLu96scf_25-391074-391326 CCGCTCGAGCTGAGGAAGGCGCCCTGTCGGGTTGCTCTCGTTCCACCATCTCCGGCTTTGCCGCCCTCCGACTTGACCTGGTCTGCGACCCGTCAGCACGACCTTTCGTTTTGCAACGGTTCAGTGTACCGCCGCGTCCAGGTCTGCTGGCCCCAAAAGGTTTAGGTTTCCCTGGAGAGACGATATCGAGGATCTATCTCGCGTACGTGTTGCCCCGCTCTTGAGTCTTGACGGCATCGAACGAGTCGCGTGA

>TU351-GaLu96scf_1-2683183-2683672 AAGGAGAATTCAACGGGCAAACCGTTGCGTCACTTCACAGCAGCCCCACAGGTCCGCGATATTTCTGCTATCTGGTGTCAAATGGGGGCCAAACGAGCATGACCGCAGGCTGAGGTGCAAACATTGACAATGCGACTTCGTGGCAAAACCGAGAGGTGGCCATGGATAGTGAAGCTGCTACATCTAGCGAACGACTCGTGTAGTATACGGTGGGGTGAGAAACCTATAGGTTATTCGCAAATGTTATTAACACTAGTTATTAACCGAGGTTACTAACATGCGAGAAACTGGGCCGGGTTATTCACACTGCGATTATCGTGGGCGCTTATCCTGGCTAATCCCTCGTCCCGACTGGCACCAGCTGGGCCCACATTGTCCCGCATGATGAGTTCGTGTTGGTCGCGTGCTGACTACGGGGTAAACCTGGAATCCTGGATGCTCAAAGACTACCTCTCGGATGTAGCTGTGATACACCCGGAGCCTGCTGCCA

>TU3520-GaLu96scf_25-510687-510986 CACGCACCACGAGCACCACCATTGCAACATGGCGTATGACCAGCAATTCTCCCCTTGGCCCCTAAGCGCATTACGTTGACACGCTCCGACGTGTACTTTATGTCCGAATGGTCCCACGTACGCTAACTCCCATCGACCACGCTTTGGAAACACTGGACGGCTTTTGAGTCGAATGTCGGCCTAGGACAGTCTTGTTGTAGCAAACTCGAGGAGAACCTCGTGCCGTAGGAGGGGGAGTGTTTCCTAGACATTCACAGGGAGAGGTTGGAGATTGGGAAGATGAGGCTGGGTGGATTGGAG

>TU3521-GaLu96scf_25-511151-511904 TCACAAATCTGCGTTATGTCCTCCAGTCGAATGTTGGGGGTCTTTTGCAGGATTATTGGACTCATCAGAATGTCTGATTCTCCGCCAGTGTGCCACACGGCCCGGGTGTTGGGTTCGCAACGTAGTGCAACGGCTAGGACCACTGGCGGCCTAACTGGTGAGTTCTGAATTCCTAGCACGAATTTACGATACAATACACCGCCATAGGGCTCGCCCTACACCGATAAGATTTCGTTTGGACATCTATCCTCGTCAGTGTGGAAATGTTTTGAAATGTAACGTTCTAATGCCTCCGTTGTTTTAGGTTGACGAGGATTCATGTGAGCTAATTTTCTGTAAAGTCAGGCGACGCGGAGCATCTACGAATATGGCAGCTCAATATCTCGATTCTCTGCAAAGACTTGGAGACTCTCGTCGGCAAGCAGTACATCGTCGTAACGGGGGACAAGCTCGACACGTCGTCTCACGCCGTCATGTCTCGGATGCCTCGGATATCGACACTTTTGAGGCCGTTTTCCCATATCCCTGGGGTCTCTCAGCGACATCCATGGGGATATAAGCTTGCTGATGACGACGCGGCGGCTTGACCGAAGAAGACTGCACACGAGAACAGTCAGGCGCCTCTACGCGTGGCCCCGCTATTGCTGCCTTTGGCCCAATCCTCCCGGATACCCGGATAGCCACCCAGAATGAAGCAGATCCCGCCCGCCCTCTCTGTAGGAACGCCGCCGAGGATATGTCGTATGTCACAGCT

>TU3598-GaLu96scf_26-258849-259458 TCTCTACTTGTCTTATTGTCCTCACTCGCTTATCCCTTTCTGTCGCTCCCTTGTTGCCGCGTCGGAGCTGGAATCAAATCGCAGGGTATACATAAGCCGCATGATCGTCGCCGTCCCAGGCCCCCCGTCCTCAAGTTCTTCACCATCCACGATGTCCCTGGCCATTCGCTTCTGCAGGTACGTTCCCCGTTTGTGCTTCCAGAGACACATACCATCGTAGCCTACGGGAGGTGGCTATGTTAGGAGCACTGTCCCTTCTGGGGATGCCGCTTCCAAGTGGCACATCGTCCTCGTCGGACTCAGCATTGTGCGTACTTTCTCCTACCCGACTACTATCTTTCCGTCCTCCCTATACCTTGAGCCCCTTCCACATACTATCTAGTGCGTACCGAATGCTATACTTTGCTCGGTGAGTGTTATAATCTGTCGATCTACGAGGATCCTCGGGCAGCTACCGCGCGGTGCGGCGTAGGATACCAATTGCCAATAGGTGCAACGTAGGTGAGTCACCTGTTGCCTCAGTTCCGAGTCTGGGAAAATATTGTTAGTATATATCTGGGTACATCTGTAGCATGGCGTATAGTAGATAGGATTGACCCTTGAATATATC

>TU3615-GaLu96scf_26-350439-351616 CCGACATTTGCCGAACATCCCAGCCTCCAGCGTATAAAAGACCGTCACCTCGCCCGTCTTCTCCTTCCCCAGAACTCCCATGTTCACTTCTTCCCCTCCCTATCCTCCTAGGTGCGCTGGCAACTCGCATTGGTGTATGCTATGTGGGTCGCCGGTCGTACGCTCTGGCGAGGGGTCGGACGTCGGACTGGGATGTTTCTCCCCCACATGTCCTCGTCATCTGGGACGGGGACGGGGCCATTGGGCACCTCGCTATCGACCCGTATGTGACACTATCCTCATCTGCGCATTGACATTCAGCTGATCCGATCGTATAGGCTCATGCTGTCGGAGGTGCCTACCCACCACCCTTTCTCGGTGCGTTTCCTCCCTTCCTTTCAGAGGCGTTATCGCTGATTGTTGTGGCACATAGTCTTCTCTCCTCTTCTCTTCTCTTCTCTTCTCTTCTCTTCTCTCTTCTCTATCGCTCGTTCCCTATCCTGAGGGCGGTGCGCCACTGGTCATGGCTGACCCACCACCATGTACGTCCAACCAGTTCCGAACTCGAGGAGAAGACCCAGACTCACCTCCTCGATACCAGCACGCGCATCGCACAAGGTGTCAGCCGCCCGTCGTTTGTAAGTCCACATCGTGGCCGTCGACGTTCGCCGCGCGTCCCACTCAGGCTCCCACCCTCGTCGCGAATCACTTCATTGTGCCGGCGAAGAGGTGGCCGAGCCAACGTCTTTCCGATACAGTCGCCTGTCGGGGACGGGCGACTCGGATTGTGCGGAGTGGGCGCGTCGCATTGCAAGGCTGAGCCTCCGACTAGTAAGTGTCTCTGATGTTGGCTAGAATTCTGGAAACTTACCTCGATAATCGCAGCGTGCACCGTGACCTTCCAGACTGGACGCGGATTCAGTGAGTCTTGATTGTCGCCTCATTTACTGACGCTTGCATCCTTGCGCGGTAATGCAGCGGGCTCCGAGTTGGCACGCGCGAGGTTAGTCCATCGAGTAGGGTATCTTGTGGCATGGGGGGTGATAGCCTGAGATCGTATCAAAGACATGTAAATACGGTATTAGTCTAGAAAATAAAACATACTGAGGCGGTGGCAGTCTTAGCTGCACAATAAGTGTATTAATATTCTGATACTTGACGGGCACTTGCTCCTCGACATTGAGAATATTGGGGGCTCG

>TU3640-GaLu96scf_26-481360-482026 ACACTGTTGCTGATCGGCATCTCTCTTTCAGACATGCTAGTCTGCTGTTCTACTGCTTGGGCCTGGGGTGTACTCTTCCTTTGATCTGTTTCAACGTCTGAAAGCTCGATCTGCCTTGGCGTTCGGTATTAGGGTGTATGACGTGGCGATTTCACGGACAAGTATTCAGGTAAGTACAGTCATCTGTTTCTATCATATCACCATCAACGTTGAATATGATGCCCTAGAAGGATGTGTAACTACCTCATTCGTCTTTCCGTTTACATTCAGCACGGCGCGTCGATACCACCCTCCATGATGTGCCTTATACTAGTTATAGTGGGACAATAGCTTCTTCCTAGACTCCTTTCACTTCGGTACTATGGTATGTCCTATACCTCACCTCTCTTTGTTCTCGTGCTCACCCTTGAACTGACCGCTGTCCCGGATAAACCCCTTGACCCTGGCACTGCGCAACCATGCGCCCGTCCGCCTGCGCCTATTGGCGCACGCACACAGGCACCTACATAGACAACATGCCGAACCATCTCGGACCCCGGACGCATTGCAGATCGTCTACGGCCATGGCACGAGCACCCTTAAGGTGGCCTTCTACAACGTGTTCGTGTCCATCCAGTGCGGTCTCTTCAACATGTGCTTGCGCCATCCCTCCACTTCTCGTTTGGTG

>TU3653-GaLu96scf_26-538937-539374 ACGCCAAAGCAGGAACTCGGCTGTAAGCAGAGGCGGTTTCCAGTGTTCCCCTCTCTGTCTTTTCCTTTCTTCACGCTCACGACACGCGGCACGCGGCACTTGTCTCAGGGAACCAGCGCCGCAAATAACAGCAATAGTAACAAACCAACCGGGAAGGCATCCCATCGCCCCCCCGGGCCTCAGACCTCGGCTCCAACAGTTCGGTCGGCGGGACCGATGGCGCCGCACAAAACAACCTTAGTAACAGTAATGACCGCGACGGGGAGGAGGAGCCGGACAACATCTTGAACAGCCAGTACGCGGCGCGCGCACGGCGTGAGCTGCTGGAGCTCGCCGTGCGCGATCTTCGCGCCTTGGGGTAGGATTCCAAGTCACAGCTTCTGCCATGGATCTTTCACCCGAGCCGACGAGGTCCTGATCATGATGCACGTCACTATG

>TU3654-GaLu96scf_26-539617-539829 CGTTCGCAGATCAACGTCCCCCGCGACGCGGGGACCTGCACTCGGTACGACCCCGCGTTGTCGTCCGGCTTCGCCCTGAGTCACTCTCTGAAGCGGCCCTCCCCACGACCGCCCCATCGACATACACATTAGTAGCTATAGTACAGTATTTGACCGCCAGATGAAACCGTGATTTCGCGCGCCGCGTACCGTGTTGACCAGGAGGGGCGGTGG

>TU3688-GaLu96scf_27-102638-103002 TAGGCACTTTCCAAGTCAAATTACCCAAGGGCTCGTAACGACTGCTCACGGCCTCGCGCTTCGCGATGCGAATATCATGTCTGCACGACGGGCAGGGCGTTATTAGTCACCCGAGAAAATGTTTGAAAAATCGGACCCTGTTGTAAGACTCAGTACTCAGTGCCAGGATTTGTCGGGAATAATCCGAGTTCGGAAAGGTATTGTGACTGCCTGCATTCCCCCCTCCCCCCCCCTCCAGTCTTATCTAAATACATCAGATTAAATAACACCCTGTGCGAAATTCGATACTACTAAGATTTCGCAGCATGCAAAGGGGCGGGTACAGTATTTAGACGCCAGATGAAATTTAACGGCCAGGTGAAATC

>TU3708-GaLu96scf_27-213294-214770 GCTCCTGCTGTCGCGGCCGCAATCCTCTAGCTAGAGCTCGAGTTGCAAGTCCTAGTCCCAACAAGCAGAAAAGGTGCAGACCGCCGGACGACGACTCGTCACCTAATACATCCCGATTCCCCGGCGGGCCGCCACTGCCTCCATCTCCACTTGCTCCTCCGACTCGACGGTCTGGCGGATGCACTTCCTGTTGGCGACCCTCGTGATCTCGTTGAGCTCCGTCTTTTGTCATTCCGTAGAAGTCCGTTCATCACATCATCAATAATCTTCGGCCTTCTCTCGCTAGCAGTCGCTCCCACGAAAACCCAGGATCACTCGTCTCCATTCCCAACGTAATCATCTATGGACAATGTCTATGTACACCTGGAGCCCGAGCCGGACTGGCGATACGGCTGGCTGACAGCACACGGCCCCCTTTCCGCAGGACATGAAGCCCCACTGAAGGTATCCGTTCGAGGAGTACAAGCCCGCCCTTTCAAGTCGCCGATCATGGACGAGCAGCGTCCTACCCTACCCTGTGGTCCAAGCAGATGGTGCATCGTGAAGATTCCCGACTTCTTATAGAAGACGCCGGTGGAGTTCGATTCCCTACGCGTTGAATGTCAATTGAATGCCGCTTCTGACCACCGGCGTCCGCCGCCATGTCTAGAGCGACGCTTCCCCATCTCACTTCACGCTTCCAACACATGTTCGCGGGTCCCCACTGATATATCCCCTCCCCGTTTTCTTGTCATTTACGTATCCAGTTGTCCTATTTGTTGTACTACGCCCCTCCAACCCCAGACGACGTCAACGACCATCGACCCCCACGAGGCAGCAATCCAGCTGCTGCCGATCGGAGCAGAGGACACACACATTCACCCTTCGCACCGGTCCTGCTGCGTACGGAAATGAACGCAGATCTAGAAGGCCCGTAACGAGAACAAGACCGCAATCGGGCACATGAAACCGAGAAGCACCTCAAGGTTTGCACCAACTAAATGAACCACTGATCTGCCCAGCCTCGGGGCTCCGAACCCGCTCTCGCGACGACGTTCTGGCTCTGCTTCCGCCGCGCTTTAGACCCCCCAAGAACAACGCCGGGTGCAGGAACGACGTCCATGCCCAGAAGCCGTGCCCGTCGCGTGGCCTTGACCCTTCGCTGGGGCCACGCCCGTCGCGAATTCACATCGCTCATATACTCGCTCCAAATCCGGACAGATCATCTCCACGCCCACTGGGCCCGGACTCTCTCTCCCGGGCTGCCCCCCCACAGCGTCCAGCGCGATGACGCTACGCGCTGGCTTCGGGCCCCCAAACGGGCTTAGGCGATTCCCGATTCGCGATTGAGACGCTTCAGACGACGGGTCGACGACGGCCGTCGGGTCGCGCGCGTGTGCGAGCCTGAGATCGCCCCGGCGCACGGTTCTAGCGACGGGACAGCTGGCTGGTGCTTGCCACGCAGGAGAGATTCGTCGATGGTCGATGGCTCGCGTCG

>TU3725-GaLu96scf_27-267506-268432 TCGATAGAAAATTTGAATGACTACAAAGAAAGACCACAGAATCGGCAGCTGTGCTTTGGCCGTGGGGTCCGACCATATTTGCTCGGTCGCTGCGCGTCGGGCGGGATAAGTCGCATAACAACGTCCAACCTCCGATCAAAGAATGCCTACGGGACCTTTTATGTGCCAGTAAAGAACGTGACAAACCTCAAAACCTCAACTTTAGCGAGCGACGTGCGGCCGGTCGGTCTATGGCAGTAAAGACGTCCGGGTCAGTAAGCGCAGTAGGTGGACAATAGTCTGGATCTTGCACCGTACCTTTTCGCGCAAAAATAGAGGTGGTTGGGGAGCCGAGCGTGAACTGCATTGCGTCATTCCCTCTTCCATGGCATGCGAGGAGAGGGTGTTCCAGTGATGTTACTTAACACCTCAAACTCAGTGTGCATGAGTGTCGAGTTCGATACTGAATCAGATCTGCACAACGGGAAAGTTCTTGGCAAGAAACTCACCAAGAGGGGAGATGATTCATGCTTGATTAGCACAAACATGACAATATTTCAAGGGTTGGGATGGACGTAACCTGCAACCATAAATGCGGTCCTTAGCCCAGGTGCGGTGAGAGAGAGACAAGTAACCTACCTACTACACCGGTACCACTGTGTCGAACGAAATGGCAGTGACTAGGTTCGTCCGGATCCAGCAGCTGGCCATGGTCGATCGATCGTTCCACGTTCCATCCTGGCTGGCACCAGCACGGGGAACAAGCTGAAGCCCGGTGAGACGATAATCGTGCCTACATCCCCACGACCTACAGGCTCCGGACGTGCTCAACCTCGACGGATTCCTGACTACGTACACTTTGAGTGGCGGCGTTGAGTAGGCAAGGTACTGCGTTGCCAAGTCACTACTGCACAGCCTCGAAGGGTATTCTATCAAAAGCCAGTACAC

>TU3742-GaLu96scf_27-395919-396507 TCTCGGATTCCACCTCGACCCCCACTCCTGGATTGTCCTCTCAACCGAAGAGTGGTGGTGGGAAGGAGCCTGGATCCTCGAGCAATTTTCAGAGACTACCTTCAAGCTCGACATCGGCAGCACATCGACCATTACCCCGCGCAGTACCACGCCGACACGGGATCATAGCGCCATGTACAGGTCATGCATCATGGGTCATCCCTGTCCGCACGTGGCGAAGAGGAAGCAGAAGACGTTTGAGCACCGGATCGAGAGGACTCATGCAACAGTCCGATGGTATCATCGTAGTGCGTTTTCCTTGTGGCTGATATGACGGTCATCAGGGACTGACTACTAGTAGCGGTCATGGCGATAGATGGGCGAAGCTCAAGCCTTCTTCACCAGTCCTCGTATGATGTCCCCGTTCCCAGCCCGGAAGAATCTGATAGGAACAGCGAGGTGGCGCGAATAACACAATGATCTTAGCTATGGGACCTATCCAGCGCGACTTCCGCCTTCTTCAAGTTCTTGCGGCCACCATGTACTAAGCGTCAGAAGTGCGGTCATATGTGCTATGTAGGTGTACGTGTGCGTGCGAACGTTGACAACA

>TU3780-GaLu96scf_28-239155-239586 CTGGGAGATTTGTGCAGCGCTGTGATCCAGAGAGCAGTTCGATGCGGATGTGGGTAGTATAACGGGTGAGATGTACTGCCAAAATGTATTGCCTCCGTATCTATGGCTAAAGCTAAAGAATGATGCATGCCCTCGAGGTAGGGCCCGGACCGGGGACGAGGGTCAGCATTTTGGTCATCTACCGGACGACAATTGAATCCGATGTATGCAATGTTCCGGAGAATCGACGGTAACCGAGGGAGGGGACAAGTCCCCTACGGTGTGAAGTAAGGATAAAACCTGGATGAACACACGTAACTCTCAGTGCTGTGCTCGTATTAGAATGGAAGCCTAGTGATCGATCACGAACCTAAACTTACCTCAAGTCCCCAAGATACGCCATGTGGGGATAACGGTTGCTCCTCAACGAGGTGGAGAGGACCTGTGTGGGGA

>TU3786-GaLu96scf_28-290408-290763 TCCACACTGCTGCTACCCTCAAGTCTTAAGTAAACTTAGATTGAATACAAAATAATGCCTGTACAACTCGACGGCTGGAAGTCGATACGCACAACGCACATCGGCCGGGCTATCGCCCGCCCTCAATGCGCCGTGCCCCTTCCACGTGAAGCACGCCTGCGTCCGAGCCTGTGCTGTGGAGACTGTATCAAACCTTGGTATGTAGCTCCACGCCCGAACCACGTCTCTAACCCACCCATAACCTCGTGCCTAACTTTCACCCGATTCACCTCTCCTGGTTCGCGAGTGCAAACATTGAACAGCCGTTCGCCCGACCTGTCTCAATCCAATCCTTCCCGCGCGGCCGGCCCTTACAG

>TU3807-GaLu96scf_29-44504-44843 CCTTCCGATACGGTGGTAGGATATCCTTCAGGGAGGAAAACGTCACTCAGAAACTGTCGCCAACTTCGCGTCGATGTGTGGGGTCTCCCTGGTACCTAGAACCTCAACCTCGTATCAGCTCGAGGATCACCTCTCGGTCCTTCTTACTACTGTCCCCCACCTGGGATACGACCTTGTCGCCATCGGTATACAGCGCAGTCAGGCGTCCAGAAACATCCTTCTCGCGCACGACAAACACAAACGCACGAGTCGCCATCGGACGTCAGTAAGTTCTAGCCCGCTACCGTTGATCTACGTTATTTCGAGTAATGGTCTATCTAGGTCAATTTTGTAAGTATCT

>TU3829-GaLu96scf_29-240193-240589 GGCGCGGGTGTGTTGACGAACCGATCCGTGCCCTCGGTCGGCTGCGAGTGAGAACGACGATGACTTCGCGTTACGTGACGCGGGCACTCGAAGACACGGTTACGCACGCAGTCGAGGAAAAGCGTTCTGCCCATAGGTTGGTGTTCCTGAGATCCGCACCATCGCGGACAACCGGGCGAGCGGGGTACAGGTACATACAATGGTCGTCGGGGCGCGCTCGGGGGGGTCGGTCTCGGGGTATAACATGACCGGTATGGCAACCCGACCATCCCTCGAGTCTTGCCCTCCCTCCCCGGTTGTCCCTCGTCCTTCGTTCATATCCCCCCTGTTCATCCCTCGCGCCCGTCCTCTTGGCGAGAGTGCGACCCTGGCAGTCGCCGTTCTTTGTCGTCTGTTC

>TU3836-GaLu96scf_29-255398-255710 TTCGATGACGCGCTACCAGCGTTCTTCAAAAGTGCTTCAGCGCTCATAGTGGATCAATTGCGCTGGGCCCCCAATCTCCAAGGCATCGCGATTCAGTTGCAGCACGACCTTTTACCTTATGAATCCCGGCGGTGGTCGCCCAAATGGTACACCTCGTTGTTAAAACGGCCCGTTGCCCGTGAATCAACAAAGGCGCAATACCTTGGCCTGGAACTAGCGTTGGCGAACCTTCGCTTGAGACAGAACGTCGTCGTCACTTTGTATCTCTCCTAATGGAGCCAGACCGCCTCGTCGATGCTCAAGAGATGTTTGG

>TU3837-GaLu96scf_29-255845-256073 GTGCGTTGCTATTTGCAGTGAGAACGGCACCGAGGGTGAGTTTGTTACAGGTAGGGAGGGTGTCGGACAGAGCCATCGTCGGCAGCTTCAGGTACTTACAAGTGTCTCCCCGAGGGGCTCGGCTATCGCGAACGACCGAGTCGTCGCCTTCGAGTCCTGGAGACGGGAAAGCCGTATACTTGGGAACGCCAACCGGCGACGTTCACATGTGCCCCCTCCCCTTGCCAAG

>TU3839-GaLu96scf_29-258928-260045 TCTTGCCTCTTTGCCTCCTTCGCCTCCCTGCATATTCTCGGAATACGTTCTATCAGGCTGTTTAACTGCCCCATCGATCCTACGTGCGACCACACTTGCAAACCGGCTCCGGCGCCATGCGTCACATTTTGCATCCGAGAACGTAATACTTCCCCATCCACCATGAATCCTTGTGCACCTTCCTCGACGAATCCTCATCGTCCGCGTCACTCTCCGTGCTCAAGCGGCCTTGACTACCTGAAACTCGAAATCTGACGTCGCTTTCTCACTCGTTCACTTTCGTCTCGCCGCTCAAACTTTACCTCATTTTTCGGTCGTCAATCGAGCGTACAGATTGTCAAATGCTACCTCTCATGTGTCTATCTCGGGCTTTAGATTCCGCATCACCTGTCGCTGGGGCCCAAGATATGAACAGGAATGGGAACATTTTTTGCACTGGCCCTTCCAACTCTCGCCATGTTCTCCAGGCAGTACTTTGGCCTCGACCGGTCCCTTAAAACGTCTTCTCACGGAAATTTGCCAGGGCTTGAAGACATCTTCTGTCACGCTGCCTTCCGGGCGCTGATACGGTGCTTCTATTGACCAATCCCTTGGTCATGAGTACACCGCAGCCTTCTAGGAAATACTCGCCTGTTCCGATGCGGGCGGCCGGCTATCCGTGCCACATCATGACAAGATCATCTCAGTTCAAGGGGTAAGGGTACGTCTTTCTTCGTACATATCTTCGTTGAATGTTCATGTACATAGTACCGTCCATGCCGTAGGTGAGTTGCGTGGCGGCAATGCTTCTTTCCCCGCTCGCCCAACCAATCATCCTCGTCTGCCCAGAAAACACACCAGGAGGCTCCTCTGACGTGATACGCAACTTTGAAAGTTGCGCCGACGTAATGCCCAACCTTTGACAGGTCATTATGTAAAAATACTTTAAAAAAGCCTCCTCGGGGCGCGATCGAGATGCAAACATAGTATGTCAACCGGGGTTAACGGTGTTATAACGCGTACAAACAGTCTGGGATACGGCGGACATATGTACCCCTCCTATGATCTCTTCTGGGAGGCGTTACAGTCTTGTAGGAACAAATGGATACCCTCAGAGCTGAAACACATCGACACTGCAG

>TU3854-GaLu96scf_29-361252-361977 GACTGCTAGCTACCTTCGCGTAGATTTCGCTCCCGCACACCGTTTTTATCGATGCTGCAGCTTGACAATTGCAGTCTTATGCGGTGCAGCTCGGTACGGTTCGACTTCACTATACATTCACCAGTTCCCTGAAGCAAGGATAATTTAGGTCTGCAGGGATCTAGCAGGATTGATCTCGTCCGACGTGGCCTTGCAGTACAAACTCCAACTTGCGGGAGCAGGGATGATCGATTGATCGGCGGCCCTCTAGGCTCTTCCCAAACCTCTCTCCGAACGTCTGGAGAAAGTGAAGGCGTATAGAGCCGCCTGGCACAGCCCCACAATTGTCTTCGACAAACGTTTTCCACAACGACCCCCTAGACTGCAGATACTCCCAGTGACTGGCGGGGTCCCTCCGTATACAACAGGTCGTAGCGACCTCAATTTTGGGAAACCGGGATCGAGTTTGCGGGGTGTCCCAGAGGTGCGGCTGTCATGCAGTCAAGCGCTACAAGACATCGGCGAGTTCTCTTCTGTATCGGTGGACGTGGGACAGGATCTGGTAGTACTGACGAGCGAAACGTTGTACCCAGGGTGAATGCATTCATTCCATCATATTGATCGTGTATGAAGAATCTGAAGGCGATAATCGCCAGCTCGCTGAACGTGGACACGTATGTACGTTTGCTAACAAGGGCATTCATTCCGCACGCGTTGGCGGCCAAGCCCCAGCTTCGAAGCGCCTCT

>TU3869-GaLu96scf_29-462300-463280 TCCTCTGATTCCCTATACCAACTCGTCTGTCTTCTAAAGCTCCCGCCCCAAGTTAGTTTATTGACCAGTGCTTCTACCATTCAGTCGCCACGAAGTACACTTGCCATCTTCGACCGGTTACGTGCACTTGCTGGTACAGTCCTTGTGAACCACGGTCTATGACATGGGAAACCCAAAGATGAGCGGGGCTGATATCGACCGCTGCGGCGAGCCGGTCTTGTCGACACCGGGAAGGAGGAGAACGTCTCCCTGATCGGCACCAATGTCGAGCCCACCCCGTCTCCATGCCCCCGCCCAAGTTGCCGTAAACCCGTGTCCACGTCGATAACGTTAACTCAGCCTACAGGCCACCCAGCCAGGAAGCGCGGGCTCGGCTATTGCCACTACTAGTCCTTCGAGCAATATTAGCATCCTTTGCGACAGCCTACAGGCCGGCCTCGCCGTAAATATCCCGAGCCAATCTCGTCTCTGGTCCCGGTCGATGGACTCGCGGCTCCGCGGATTCGTGGAACGTTCCCCTGACCAGCCCGGCGTGCCTGCTGACTTTCCTGGCGCACGGCAATCCACGTCTTGCGGATGATGCTCGGGAACATCGTGGCCTCAGCGCGGAAGAACCGGACGTCCATCGTGCAGGGTTCCGCCCTGCACCGTGAGTGGGTCGATTCCCCCGCTTCCCGCTTTCACGTCAGTTTAACCATACCGCACTTTCTTTAGCTCAGGCTCCCAAGCTTTGTGCCTGCTCCAAGGCTCACTTGGGGCGCAATAGAATTGCTTGGGGTGAGGGATTGTGTAGGCAAACCTTACGCCCCCGCGGTTTCCCGACTGAATGGTAGCTTTTCCAGCGCCGACCCACGCCCGCCCGGGATACAATGCTCAAGAAACCGCGCTAGGCCAATCTCGCACTCGGAAGCTGTTCGACGTTCGGCGCGACCCCTGATCCTGATGCTTGGAAGTGGAAGTCGCCGACTCGGTGGTTACCAC

>TU3884-GaLu96scf_3-31956-32188 ATGCTCCATCCGAAATACGGTAAAGATCACGGTAAAAGTAGTCTGAATAACTTTACCATTCTCCGCTGCAATAACTGTATCACCTCATTGTAGTGGAATACGGTCCTATCACCACGTGCGCCACTTCTTACCGTCGCTATACAAAGGGCTTTACAATGTCCATGGCGAAGGATGGTAAAGGTATTCAGACTAATTTTACAATATTCTTTACCGTAATACAGGGCACATTACCC

>TU3907-GaLu96scf_3-193636-194449 ACGTCTCGTTCCCTGTCACGGGCATCTTCTCTTCCAAGTTCCAAGTACGCGAGATTCTGAGGAGGGGTGACCATAGCGTATTGCCCTGACAACGTGCAGCCGGAACAGAAAATAACATTCGCTATGCGAAGCGTACGCCGGATAGAAGTGAGCGAGCGGCTTGCTGTCCTGAAGAAGGGACCCGCGTCACGGCAATAAATCTGTGAAGTAGCCCGACGTAGTCTTACCGGGCGGTTTGGTATGGTTATGAATGTGTGAAGATCGATGGGACCCTCCAGGGCCCCTCCGGATCATTCTGATGCACGACGTGAACAAATGTTAGTGTCAAATGGACGTAAGTGATCTGGCGGGCGAGCACCAACCAATCTGATGCTGATGCGTGTGGGGCTCACGACCGGCCGCAGCAAGAGGAGAGCTCTACTGGTGACATTTTGGACACGGCCGTTGAAGCAGCTTATTCGACGGGTGCTTGCTACTGTCGCCGGAGTGGGCTTCGAGTTCGGCGCCTTGTACCGTATATTGCATTGTAGGACCCTGGATCGTGGGGACTGCCAATGCGAGAACATGTGGATTCTCTCCAAATTTCAGGCCCTGCAGCGGATCGGGGTGTCACAAGCGATGATTCCGTTGAAGGGAATGACGAATGGGGTAGTCTGAGAATTCCAGGTCAAATATAACACGATTTAGAGGATGAGATTTTCATTACGGCGTAATTGACACACCTGACCCATGAAACGCAATTCCGAGATCGCGCAAGATTATGCACCACTCATGTGTTCCAGAGCGGGTCGATTGAGCCCGAGGATGCGAGTCG

>TU3915-GaLu96scf_3-323869-324246 TGCAATATCACGCCTCGCTCTCCAAGCGCAGAAGCCCTCATTCATGCATCAGAGACAAACGTTTAAACAATCACGGAGTTTCCGAGTGGGTGTTCGTGGCAGTGGTAGGTCTAAAACGTATTCATTTATGTCTATGTCTGAACTTGAAAGTCTTCGTGCGTTATGGCCATCGTCCTTCGGATCGGTCACTACCTCATCTACCTTGGTCCAACGGCTCATTGAGCACCATGCGACCTGGGTTCAGAGCATCTGATCCCACGTCACTAAACGCCGACATTCACAAGGTTGAGGGTGACACACACACTGCCTGCTGTACTTGAGTTTACTGGTACTTACTATAATTGCTTGGGTTGTTGCGCATGGAAGTCGCGGAAATCG

>TU3916-GaLu96scf_3-324407-325460 GGTTCTTCCGAGCTCTATCCTAGTTCAAGTCACCAGTCCAGGGCGACACGGTGGCTCAGACTTGTGGTCCACTGGAGGTCAACGTTGGGCGCGATTTCACATGCTCTTGTATTCCGCCCGGGATTCCGTACATGGCACTAATTCGCAAGGCAGCAGCTCCCACACTACTTAGTACTGGCGGGTTCGACGGCCCACGGAGGGCTCCCATGATACAATTTATTCAACTGTCAACTACTCGCACATCCAAGCAGTTCTGACAGAACTTGTTGATAATGCATAATATCCGCACATCCCGATTCAACTCGGAACCCACTGGACTACAAATGCGCATGCACTCCCAGCCACTAGTCCACTTCGCCAGCAGCCACCACCCTTGCCTTCGCCCTCCCCCAGGCTGATCACTCCACCACAAACAATAACGTGTCCCGTTCTTGCGCCTTGTCGGCTGCTACTATGCCTTCCGTGACCACCCAAAAGCGGGGGAGGAGGATTGCCTCTTGGTCCGTTGCTCTGACTGCGCTGGCGCTCGTGGTTCGCTACATCTACGGCCGCGCTGGCAAACGAGGCGTATACTCGGCTTTGCTCAAGAATCCCGCCCCAGCAGCTCGACTGATTCGCAATGGAGAGGAGGAGTACGACTTCGATGAGTACGACGTTGTCGTGGTGGGCGGAGGTGAGTCGGCGGTCAGCTGTCGGTTCTACTTAGACTCTAGAGTGTTAAGCCGGGTCCCATGCTAGGGACCGCTGGGTGCGTGCTCGCGGCACGATTGTCGGAGGACCACTCGATACGCGTATTGTTGCTAGAGGCGGGTGGAAGGTACGTGTTTTCCTTCGGCGCGCGCTGTTGTTTGGGATGGTTGTTGAGGCAAATGCGCTAGCTCCGTGAAAAATCCCTTCGCCATTATTCCTTCCGCCTACTCCAAGCTTTTCCATACCCAATGGGACTGGGAGTTATTCACCGTCCCCCAGACCGACGCTGATTCACAAGTGAGGTATTGGCCGCGGGGTGGGTCCATCTGCTTTCCTTTTGAGCCCCTATTTATGAGCGTGTGAT

>TU3925-GaLu96scf_3-378627-379157 CTTCCCGGCTTGCCTATGCTTTCCAATGCTCAAAGACATCAGTGTGCACGCTCCTTGTCTGTCTCTTCTGTCGACACAGAATAACTGGAAGCGATCTAATATGCCTTCTTCGCATCGTGGCAAAAATGCTTCAGACTACGATTACCAACTTGAATTGTAATACTCCTCGTCACTGGCTACATAACCGACGTCGGCCGCTTCCCTCCACTACACATTCGGTTCAAAGGTCCTGATGCGAATTCCCGTTACACGGCACGCGGCAAAGTTGGAACTGATGTGGGACCGGTGGGTGGTGAATCGGTTACGCGTAAGGCCGTTCGCGGTGCCCTGGGCAATATGATCAGGACGACCTTGTGAAACGCAAAATCGACGGTGTATGGCATATACTGCCCAACGTCGACGGACTCGACGCTCAACCTCAATCTTCAACCCCGGTGAAAATCGACAAGTCCCCCAGCGTCCCGGAGCGTGCGCGATCATTCGTGGTCGTGGGCTTACGCGCGGGGCGATTCTCACGATGGGGGACGATGG

>TU3933-GaLu96scf_3-435600-436355 CTTCAACCACGACCCCGTGGCTACGGCGCCCGCCTGCGCCTTTTGAGCGGCGAACCCTTTGGAGCTAGTACCACCTCGTTAACATCAATCTCAGACCCTCTCATGCATGAATTCGAAAAGGCCATCCACGTGTACTGCTCTTCACAGCGCCGTTAGCCCTTGTGTTAGTCATCGTAGGTGGAAGAAGAAGATTTGTAAGCCTGCGCCACTTGGTCACTGGTCACTTACTATTATATCTTCCGAATCCGACTAGTATAAAAGATCTGTCCGAAGTCGACTTTCACGCGCCTTACCTTCTAGTTTCCCAGTGCACCAAAGAAAGCCAATACCCCAGTTTCAAACGCCTGGCTTTCACGTCGACGTCATTCACACCCCCACACAGGCATCAGCAAAAGTGCAAACGGGTCGTATACGATTACATACGCACACAATCCAGCCAATCCAGCCAATTTTGCAATTGACTTGCAGCGACCTCCTCGATTTGCGCAACCGCATGCAATCCCTCCAATCACTTGCAATCCAAGCAAGCGATTGCTACGATTCGGGCGATTGGCTGGATTGCAAATCAATTCGTACGAATGGGATCTAGCTGGCTAAATTGACTCCTGGGGATTGCGCAGATATAGCAACATAGGTAGAAAAACATATATATGTATATTTGTATCGAGTACATTGCCAAGTGCTACTACATAAAACTCAACAGACGAGACTTACATCCAGAACTACACCGAATGAATGGAGCTCCGAATCGTCATC

>TU3946-GaLu96scf_3-584736-585174 ATGTGGAAGGGTGATGGTGCAGAGCATTTGTTGCCACTGCTATGTGGCGACCTCGGACTTCGAGAAATACTTTGTTGTCGACTCCCTTCGCGCGATGCATTTGAATTCGGAATTGGGCGTGTGGTTCTGCGCGCTCCACCGATCAGCCGCTGTGCTAAGCCCGGAGCCCGTAAGTAGACGATGTAAAAGGCTGTGAGATGGTTCCTGAAGGTACAGCCCAAACACTACCCCTTAGCTCTCGCGCATGCAGTACTTACGACTTCACATACAGAGAAGAAGACCTAACGCATAAACGCCCGAAGACTACGGGCGATTCTGGCTATTTTGAGCAAATGCGACAGCGAGGGAGGAGTCAGTCAGACGTTCTGGGAGATCTGCGAGGGGCATGCGAAACGAGATACTGTATGGGAGGACGAGGATCTGATCGAAGTCGGTCGGT

>TU3965-GaLu96scf_3-734619-735211 CCATGTTTTAAACTTCCCTCAGTTTGCCTCCCTCCCCTTCCCCCGCCCGCGACCACTCCCAGCCCGTCTTCTGCGGCGGCCAACGCCCCACGCACCCCGCTCCGTCCCACGAGGCCTTGCCTGCACAGGGGAGCAATCTCTACGGCTGACGCTCCCCACGGGCCGAACCGAGATCCGCCTGGTCAAGAGTGGCTTCGCTCGAACTCGAATCCCATTGACTCTCTTCTCAGAACCTTCTTCTGGCTGAAAAATTTGCCCGGTCTTCTTTCGATCGATGGGGCATGTCCGGTTCGTCTCTGTCTTGTACCCCACATACCGTTTACCGTGACATGGCCAATGTCACCGTGCGACTCTGTCCGTTCCCCTCCAAGCGCAGCTGCCCAGACCCTGCTCAGAGTCCGACCTCGTCCCTCCCACCCTTCTCTTCATCCATCTCGCCCTCCCATCTGCGGCTCCTGCCGACCATCCCATCTCCCCACGCGCTCACCTGACATCCCACACGCACACATGCTCATATTTCCATTGCTTCCCTCTCAGTCCCCCGTACGCTCTTGACTCTTGCCTCCCTAAAACCCCCGCACACGGCGTCCTCG

>TU4038-GaLu96scf_3-1168527-1169256 ATCAGTCGGATACATGCGGAAGTATCGATAGGCCTGGTGCAACGTGACACCGTACATTCTGCGCGCGAGATAAATCAACTTCTCATCGTTTCCGTGGTCTGGTATACGGCTCAACGTACATAAGGCCCACATAACTCCCGATCAGATAAGGACCGAACGGCTGATCGACGGCTCCAGAAGTTGAAGCCACCGCTGAGGCGTCCATAAAGCTTGCATGGGGGTGCGGCGGGCAGAAGGACTTCGAAACACGGAGCGCGGGATATATATGGCGGAAAGGCAGAACGCTCCCCTTACATGCTGTCAACCATGATACGCGGCGACGAAGCGAGAAGACGAACTCTATACAGTGCTTGTGTGTAACTTGGTGCAGGGTCGAGAGCTCTGTGCCGGCCGCCTGACGAATTCGCGTTTTTTTGCACTTAATTACGGTCCAGTTTCAAAGAAACATCCCCCGTGAGCTGTCCATCAGTCTTCGCTGGTCAAGTTGTACGTTGACAATTTGACGTTGAACGCATTTATCCAGCCCGCCCAAAGCCCGAAAACCCACCAGTCGGTACCGCGCCAAGGGGCGTCCCCGCTGCAGAATGGATATTCCGGTCGCCAGGTCGCGATGGAGTAATGTTAGGCGTCGCTTGTAGCTTGGCCTTCAAGCGATCTCGTCATAATTTGTTCTGGGTCTTCGGCCCGCGGCGTTCAGCGCTCAGCGCTGAGTGCCAGCCTCGAAAGACTG

>TU4039-GaLu96scf_3-1169385-1169704 GTCGCGCAGCCAGGGCCCAGGGGGGGGTCTCTAAATTCCACCCGGACCCGAACGTGGTTCAAACTGGTTTAAACTTCAAAGATGGCCATACACTTAGCTACTCAAACTCGATGAGGTGAGGTGAAAGTTGCGTTGGGGGGGTAACAAAGACACCGATGATGCCTATCGGAGGCTCAGAGCTCTGTAATCACCATTGGTGGTGACCAGTTTTGGAGGATGTACCTGAAACTGCCATGACCCTCTATGTACTTAGCGTGGCGCATGTCTCACTCACCTCACTGACCAGGCAAGCTACTGAACTGATTTTGTGGTCGATGAAC

>TU4055-GaLu96scf_3-1261255-1261947 CGTTGTATGTCGGTGAGGGAAAAAAACTTTTGAGTTATGGATATGCAAGCCTTCGATGCTTGGCAGCTGGTCTTTGCTCAAACGCGCTGAGGCGTTGTGGCCTCTGCGTGTTTTTCGAGAACCCGAACCCGCGCCTCTCTACCACTAAAACTTAAATCCCAGACGCCACCGACCGATATTTCTTCACCTCAAGGGAACAGGGATGTACATACCATGTCTTAGTCCAATCGTCTGTTAGACAGGGTGTGCGTAGACGAAGCGTGAGGGTCATTGAATATCCTCGGATGGGAAGGAGGTGGAGACCGCACTAACCCAGGCTAACAGTGCAAGCGAGAGAATCGGACGGGTGCCCGGCGTCCTGCCGAAGAGAACCGCGGTGGTGATCCAGCAAACGACATTTGCAAGCAATTCTGCGGCCAGGAGGAGGATGGAGCGGCCGAAGAGCGTGAGTCGGGGGAGATAGACGAGATTCAAGTTGGCTAGGCGCCAGGGCATGGGGTCGTGATCACAAGCGCTAGCACTAGAAGGACTCCAGGACTCCAGGGGCGGCAGTCTGGACTCTGGAGACTACGGAGGCTATTCCCGAGGTTGCTCGTCTTGCGGTCGATGAGGTACCGGGGAGAGATATATTACACCGTTATCATGCTGGGTCTCCATGAAAATGCGAGGATGGGTCCATAAGCGCTAGAGATA

>TU4069-GaLu96scf_3-1376732-1377100 CGTCGCCAGTCGTCCGAGACCACAATCTTCCGCGGCCACCGTACCCTCTCCCACCATCCCTCTTCTGACTCATTTCACCTTCCAGCGACCTTTCGATCCTTCTATCTTCCGCGTCCTCTCTGCCGGGCTGGCTCATGTCCCTCGGCCGTTCAACATAATGATAATCCTCACTCACGACTTCCAGCGACTCATCCCACGTCTAGATCTGTCCATCACACTCTCATCCTGCGCACGTCAAAATACGAATACGCCTCGTCCTCCTTCCTCACTCTTCCTCACAGTGGTCTGAATACAGTTCACATGTACAATATCCGCTCTTGCGTGGTCGATTTTGGAAAATATGTGCACTCCGCCAGTATCGGAGAGTGG

>TU4109-GaLu96scf_3-1507762-1508356 TCTCTGCTCGAAGTTCTCTTATTGTCCTGGCTCGCTTACCCTTCCGGTCTTTCCCTTGTTCCAGCGTCGGAGCTGAAATCAAATTGCGGCTATACTTAAGCCATACTGTCATCGCCATCCCTTCGTCCTCAGGTTCTCCATCATCCGCGTTGTTTTCGCCGGCCACTGCATCTCAGACTGCAGGTATGTTCCCTGTTTGTGCTTCCTGGGGATACCCGCCATCATAACTACTGGAGGTGGCTATGACACGAAAGAAGTCTCTGTTGGTGACACCACTCTCGAGTGGCACACGGTCGCCGTCGGCGAACAAGGTATGGTAGGTACTTCATCCTCCTTAACTGCCGTCTTTCTATCCACCCTATGGCTTTAAGCTTCATCTATGTGAGCTTCATCTATGTCCTACCTGGTGCGTACCAAATCCCAGCGCTTTGCCCTGTGAGTATCATACCCTGTTGACCTTGAAGGATCCTTCCGCACTTACTCCGTATGCTGATAGGTGAGCTACCTGTTGATTGTTTCACAATTAGAGTTCCAACTAAGTAGTATGTCATATCCTCAAGAACTTTCATAGCACGGCATGTATGTAGTTGCCTAG

>TU4115-GaLu96scf_3-1528700-1528981 ACCATGTGTCCCTCGGAATCATACAGGAATCAGGAGATATGAGCTCCAGATTATTCCAAAGACATACTCAGAAAACCCATCCGGATTTCATGACATATGCTCCTCATATGTATCCCCCACTTACATGCCGCTTTCACCGAAATTATCATTTCACATATTCCCATTCGTATTCCAACATGATTCTTCGGGCCTAATTTCAAGCCTCATATTTTCACGTTCCGCTTCTGATTCCGCACATGATTCCGCAGGAATCATCGGAGGTTGCCCTCGGTGTCTGGTAAG

>TU412-GaLu96scf_1-3367105-3367636 ACGGTGTGCTTCGAGGCCATACCACATCCACCCTCCAAGTCATGAATACTCCACCTTGGAAAAGGCTTGAAGATGCTCGTATTCGATCTTAACTTGACGCTCTCCCACATTCGCGCCAGATCCCATCCGTCGACCCCATGGACCTTTCAACTTTCAGGCACGGGGGACGAGGGTCCTCACAGAAAACCCCAATACATGGCGGATGAACGCCACTGAAGTCGGCGACTGGTCTGCTGCCAATCCTTTCTCACCAGTTTCACGTTCCTTGTCTCAGCGTAGACGAATGTTGCGGTATGTCTGTGCTGACGAGGGCTTTCCCATAGGCGGCTCGATTGGGTTCTTCGGAGTTGCCCTAGAACAGTAGGTAAGGCCACGTTTTCTCGCCTGGTTTTTAGATATGCCTTAAAGAGGCCTTGTGCATGTTTTCAGACCAATCTGACCGTTAATTATTCAGGAAACGTCGGACTAACGAAGCGAATGGAAACCCGTCCAATAACGGCTTCCGGAGCTCTATCTCGAGGGTGCTTCTAGC

>TU4149-GaLu96scf_3-1678422-1679935 GCTGGACATAAAGCGGTGCATTTCGTGGACAGAGGAAGTACATGTTGCCCGGTGCGCTCACTGGAGGTAGTAACTATTAGCAACCGCGTCCTCACTTCCAGGTCCTCGTTCTCCAGATCTTCAGGCCCCGAAGCCCCCGGAGCCCCCACAGTGAGCGACACGCTCTACCCCACTTACCATGCGTGTCTCCCCTTGTCTCCGTAGCGTACTCCAATTCTAACCTTCCCGATTGTCCTCATGAGTTCCGATCGCAACGACGCCACCTTACGACCGTGGTTAACACGCTGGCGACCACCCTTGTGTTACCGCACCACAGGAAGCCCGATTGCGTAACGATTATGCGAACAAAGACACGAAGCGACGGAGGGGAGATTCGACCGTCAGGAACATGGGAGTCCTTTCGAGAAGAGGAGGACTACTGGAAATCTGATGTGGGGTGGTTCGTGGGGCGCCGCAGCCAAGACCGCAGACCGTTATTAGGATTCAGTTGTTCGAGGAAATGCAAAAACTCACTAGAAGCCTCATAGATCTGGTTTTATCGAAGGCAGCCGCCACGCCCAACCATTAGCGATTTGAGACGAGGAACCACGCCGACAGTTGGACCTTGAGACCTCGGCCCATCCTCAGGAATCGCGACGGAGGGGGAAGCACCGCCCTATCTGCATTGTTAGCGGGCGTCTCAAGTTAGGCTGGCTACCCGGGCACACGTGGCTAATAGTAAAACGTTAGGGTTCGATACACGGGCGATATGGGCTCTGCGTTTACTCAGGGGGTATGTGTGCCCTAGGCCTCAGGAGCCTCCCTCGAACTTGCGAGTATCTAGCACGAGAGATAAGCGGGCACAAAGCTTGCCGACAATGTGCGCGATCTGCAACAACGATCGAGGAATGGAGGAAGCTAGAATCAACTTGAACCCATGAGACTCCATCGCACCAAAAATCGGTACTCGGGCAAATCGGCCTCGCTCGCATCTCCTACCCACGCTCGCCGCGTACCGCAAGATGCCCCGGGGAAGACATCCGCCCCAACCGAAGGGTCGGGAACGAATCGCCTGCGCATGCACCTACATTAAAGTCAGTATGTCCAAGGTTTGGTGACGAGTACGAGAGAAGTTCTATACACACCTCGGCTAACAAGAACCGTGCGTGCCCTGGGTGGGATGACGGCACAGGGGCGTTCGGGTTCGGGCGCGATTTCCCAGGTCCGACTAGCGGGCCAAAGCGACACCACAAAACGCATACGCACGGCCAATCCATATCCGGTAATCGCTATTGAGAACCCTTGCACGCACCGCGGCTGGTCGGCATTCTTGCCACAGTCGTCCTCGGCAAGGTCGGTGAGGCACGTTGCGCCGCACAAGTAGCATGGGATCGGCGACAAATCCGCACCCTGGTGTCATTCGGGGTGGACGCCGGAATGCCGGGGGATACGCTTTTGACTCTTTCGCCCCAGCCACGGCAAGACCGGGGCAACGGTTGGGCACGAGGCAGGCGGTTAAGGTCTGGCAGACGAAA

>TU414-GaLu96scf_1-3377647-3378088 TAATTTCGACGCTGCAGCAGCTGGTGATCGAGTTGCAATCCGCCGGTCCAGCAGGCGCTTTCAGCGGAGAAGGAAAGCATTCAGCATATGTTTGGTTTCGATACTAGATGGTACACTCACCTCACTACCTCACCTCGCCACCTCACCTCACTACATAATCGGAGGTCATCGTAAACGAATGCCGGTTTGTCGTGCGGCAACGCGCGATATCTGATGTAGCGCCGTGTCGGGCCTCTGGCGGAGTGTCAGTAATGAATGGTGCGTTGAAGACATGCGGCAGCCACAACCCGTCACTGGTATTCAAAGCACAACGAAGGCCGAAGTCTGTTGACCTCCGATGTCATTCCTGGGCGATGAACGACTGGAAGGAAGTTGCGGCCCCTGAGGCCGCTGTCGCAGCCAGTCGGACCGCGATGGTGCAAGTGATGGTCAGGTACGTCCC

>TU4155-GaLu96scf_3-1695093-1696173 TGCGGTAGCGTCCCGCAAGCACCCGAACGGTGATAATCTGACAAGACATGCACAGCTTTCTTTCTGATATGTCGTCGTTGAAAGGTTTGCAGGTGCTAATACTTTGCTGGTGTTGACAGCGTCATGGGGCCTGTGTTGTATGAACGCCACAAATGGGGGTATGCGAAACTGTATGGTATTTTGTCAGACGCATGGTACTCGGTGGTTGCGCTTCTACTGCGGCGTTCTCCTGTAAAGGTTAGCGCAATGAATGCAACGTGTACAACCATGGGACAAACCACGGCGGTGCTTGCTGGCGTAAGTACATGGGCTAGATCCCAGGCGGCTCAGGGGTGAGGCCTGTGAGGACTCAGTACAGAACGCGTTTGGGACATGTCACCGCCCATGGAGCGTAAAAATGATGAGAGATCTGCAATTCAAGGTCGTTAGGTTCGATTCGGGCATGATATCATATCTAATTTTGCACACGTGGTATTCTCTGAGAGCCGACTGCACAAATAAGTAGCGCGATAACACTCGTTATCCGAACCGCGCATGCGTCGTCTTCGAGCAAACGGAATCCGCTCCTCGATCTACGCCAGCGTGTTTGCACCGTAAAGGTACAATCCATATAGGAGGATAAAGTTGTATGTCCGCAGTGTTGCCCGTGTATTCATTCCCGCCGGAGGAAGTCCGATGCAGATCGGAACGAACCTTGGCCGCATCTACAGGGGGAGCAGAGAGGTTCGGGAGGAACGTGGAGGTAAAACGGTGAGCCGGGGAGGTCACGATGTACACCATACAAAGGAGGCCGCAGCCCCAACAGATGTCGCCGGGCAGAAGTCTTTCATGCGCATCAGAGAAAACCCGATGAACCAGAATACGTGGAACTCACAAGATGAGCTGATTATCATTATCAGATAGAGAAGACCAAAGGACCGGCAATTGCATCCTCATCCTCGTCATCCCAACTCCTGCTGAGGTTCAGGTGAATCTTCACCGGAGACCGACTGAGACACAGACACGCGGAATGCTGGAGATGGAGAAGATAGATTTATTGAGTGAATACTTGATCGCAAAGGCAATGAGCAGTGTGGACGAC

>TU4156-GaLu96scf_3-1696577-1697341 CAAGTGTGCGATCGCTAAGCTATGTTTCCCGAGTGCACCCGACGACTACCCGACGGCGAAGACGAAATGATCTTGAAAATGGCGTCTGCGACTCCACGGTCTGGGTTCTCTTGCACTGCCTGCAATGAAGATTTTTAATATGTTCAGCTACCAGTCTATTCTCAGGAACGGGGGAGGACTCACTAGGTGTCGACCACACCGTGATATTATCGAGATGGTTGCCATCACGCCTTCGTTCAGGTGGCATATCAACTTGCGCGACTCGAGGATCTGAACATCTATCGTTATTTCTGACCGGGGAAACCGTGACGAGCCACTTACACCATCTCGGGGCGTTCTGACACGTCGACGGCCTTGGTCGAGACTAGACAGAATGCACGGGATAGCGGGCACACAACTTGGACGCGAGTTTCCCCGCGAAGTGCACATGGTCGTCGGTATAATAGAAGCCGACTTCTTGTGTCTTCAAGCACTTAAGCATTATCCTCTCCTGTTTCGGGACAAGGGCGTCCCCTTCTACGAATCTGTATTTCTGGTATAGTCAATGACTTTCCTCTCCAACACACTGAAGACACGGTTAGCTCACTAGATATGCTTCGAGCACGCGCTCATCCCAGATTTTCCAGGGGTACCGCATCCTATAGCTAACCATGCTGCGAAGTGAAGCATAGCTATCCCCGACCCTCGGAGCGGGCGAGCCCCCCCCGCGACATTAATATTTGTGTCTCCAGACTCCAGCCGTGATGTGCAGTCTCAGTCTCAGTC

>TU4164-GaLu96scf_3-1739038-1739500 CGGGCGGGCGGGTCCCCCAGACAAGGTCGAAAGTTGCAGAAAGTATTTGAATTGTTCTCGGACCGACGGTGATGACAAGTGGAGGCAGGTTGCAGCACAGGTAGCATACAGATGCTTTGAAACGAATCAAACGAGATGAACACCCGACGCTGCCACTCCTGGTGACAGGTATGCGTTAAAAGATGCATTTTCAGCAACGACAATGGGAACGACCCACCATCACTCACCTCTTGTAGTGCATCCTCAGCTCCGACGTTCTCCCTCCTCATCCGGATCCGCTCTTTGTCAACTTGTACCTGCTAAGACGGCGTTGTATATTACGGCAGCTCTACCTATACTGGCCGCCAGAGCGAGTCTTGATGCGGAGTGATTAATGCAGGAGACATGGCAAGCCACACAGGGTAGTTTCGGATGGGCGTGTATGAGTGAAGCCTGAGAAGAAGGTCGACAACTGAGTGTGTCC

>TU4178-GaLu96scf_3-1827008-1828091 TATGCAGGTACGTCGTTCGCCCCGTGCGTAGTGTAGATTCGTGAAGTCCGGTGCTGGGCCAACATGATGCTTGTAAGCACGGCGTGTATTGTGCCAATAGGGCTAGTATATGAGTCGGCCGTGTGAGGGGACTGCGGGCAACGTGCTTTCACGTCCTCTTCATCTCCGTATCCCAGTCGAATAGGTGCCTCCTTTCTCCATGTCTTCAGACTCGGATATTGCAGAAATCATCTCTGAGCAGTACTTTTTGTAAGTATTCTCCATCAATCCTCCCCTTGAATGCTGACCTAAAGCCTGTGGTTCACCGGTAGATGGGTCGGCCTTTTGGTCACACACGCTACACTCAGTGAGTGTTACCATTATACTAGACAAAGTCTGACTACTCAGTGCATGTGTTTGTCGTAGCCCTCATGTGCTATGATTGGTTTTTGAATCTAGATGAGGAAATCAAGCTCTTTTGGAGTCGGAAGGTGACTGCAGCGACAATACTCTACTTCCCCATTCGATACCTCGTGATTGTGTATTGGATACTGGGTTACCCAGTGGTTTCGATGCCAGGCGCGGTAAGATGAATACCGTCACCTCCGCGTATGAATTGACATTGACATTGAATATGTTTTGCCTTTCAGAGGTACGTCAAACATTGTATATTCATCAGAAATATACAGTACAATTCCAAACAAAATCCCCCTAGCTGCGAGGCATACTTGTACACCATATACGTCCTCCAATTACTCCTCTTTGTCTGTCCCGCTGGTAGGTGTCCCACTAGCTAGTCCATTCTACCTTGACAATGTTAACGCCGATCATATCTGCTCTAGCGTTCTCCGCTCTGCGCGTGTATGCACTGACGGGCCAGAACAAAATACTAGCTCTGATCACCCTATTCTTCTCATTGGGTCCCATTTACGCCAATTCAGTGCGCTTAATTCCCACTTGAGCCTCGTCATGGAGGGACCTACAACGGAGTTTACAAACATCAGGCGTATGAGGCCTTGCAGGTTTCAAACCCACCTTCCGGCGGCACGTGAGGGCAGCCCATGCGAGGCCGCGCATCAGTTGAGGGTGATGGGACCGCATGGGT

>TU4189-GaLu96scf_3-1882141-1884004 AATCAATATGCCAAGCCTTGAAGTTGAATACAGACATATGCTGCGCGTACCGTGGTATCCTCGTGGTGCTGTCCCTGTAGAATCGAGCAATTGTCAATGCTCTCGAAAGCAACGTCCATAGGACTCGGAATGAGTTCATACCACGAATTCGAAGAGGATTCGTCACAGGCAATGAAACATGTGCGGTCTCATTTCGCCCTTTGTCGGTGCTAAGTAATCATCTTGGTCATCCCTGTGCTCATACCTAAAGATCACGAGAAAAGGAAAGGGCAGAATGGTACGTCAATGTAAAGGTTACCTTGCTGGCTCCTCCGTCGGCTGGGGGAATTAGACATGCCGTCACAGCGGCCTCATGCCAAGCCATCCGAGGTCAAGTTTACTTCGCTACCGCATGAGCAGAAGCGGGCGAGGCCACTCTAGTCTCCACCAAAGCGATGACTTGCGCAGACTCATGAGACCAACGTTTCGGAGGCAAAGACCACTTGCGGCGAAAGAGAAAGCCGGACACCGGATCGTTTGCTGCGTGTTTGAGGACCTAGGAGTTGACTCAACAGCGCTCCGAGCGCTGTCGTGATATCGGAATTCGCTAGTGCAAAGAATGACTTGATAGATAAGGCGATGTGAGAAGTCACTGTGAAGTCCCGAATGGACCAAATCCGAACGATCCCGAATCATTGTGGGGGTACATGGGTGTCGAGCGTTTTTGACGAGAGTGGATGCTGGCTGGATGATGATCGGAAGCAACTTGATATCCCCGCGAACGAAATTACATGTGGCTCTCACAACGTAATGTCAAACGTCAGGAGATGACACCTTCATTATGCGTGTAGGGAAATGCCTGCCTTGGAACAACGAGTGACGTTGGACAGAGTGGCATGCCGCTATATGGGAAGTACTGTATGTAAATGAGAGGTATTGATGGAGGATTTCAGAGCCAGCTTCCCTATTTGCTAGGCCGTACTCGAGATCATGCTCGAAGCGGTTCGCCATACCGCGTTGAGCTCGAAGGCCAAAGGAGTGGCCCAAGTCTAAGAGACGTGTAAGCCACGATACGTAGTCACACGGCGAGAGCAAGACTGTAACTTGCCTCTCGTGCGTGGCACTGCGAAGGACGAGAACAGACAAGGTCACCCTGTCTTTCGCTTCCTTGCAGGTCGGTCCACGCTCATACACGGAGGGCGTTGACCATGTGATGCGTATGCACGCATCCTCTGACCTCAGTCATTGCAATGAGGTGCGATGGATCGCAATGTTTCAAAGTTAAGAATGATGCTGTCGACCCCGATACCGTACTTAGTCACCGGGTGGCGTGAAAGATCGGCGAAATGGGCAACACTCACCCACTCGGACAGCATATGCTAGACACGGTTGTCCGATGGAACCGCAAAATGCTACGACCGGCATGAACTGGACGCAGCAGAGCCAGGTCATTTGAGCATTTCCTCGTGGCACTCGCGGGCCACACAGTTACTTGGCGGGACACAGGTCAATCAGGCCTCTCCAGATCGCCATTCCGTCGTCTACTGGCTTCGAATCCACGATGTAAAACCACGGTAGAAGTCGTCCGGTCAATGACACACCTTCGAACACTCCCTCACAAGTACGATCATCGAACTCCTGTTCCTTCCCTCAGTCGACTATACAACCTGCAATATGCATATTCAGGTTGACTTTGAAGCGGTAAGAAACGGAGACCTCACCACGCCGCTCACCAAGCTGACCAAGTGAAGAGCACGAGGGTCGGACCGTTCACCTTCGTGAACTCATTTTGCGAATCCGCGGGCCACATGTTGTCGCACAGCCGTATCTGCGCATCAGGACGTCGAGGCAAGATTGTAATGCGGAGCACACAAGATCGTCGTCG

>TU4214-GaLu96scf_30-187481-188176 GTGCCTCCGCGATGCGTATTGTGTTGGTGATTTCGTCTTCGCGTGGATGACTTTGCTGAGGTGATCGAAACTGACAGGAGCATTAACCAATGTATTGGGAGGACAAAGTCGAATTCGACACAGAGATACCACCAACACTGCGTTGCGGCTCTGCGAGCTGCACGCTACCTCGGTTGATGCATTGAAATGGAGACGGGTTGTAGGAAGGCAAGACTAGTCCGTACGCCATCATCGAGTTCCGTGAAGCACTCACCGTCAGTGGTCCTAGACTCCTAGAGACGCTAGATTGGTAATGGCACCAGGTGGCAGGCTTGCAGCCGCAATCAATCCACCAGCCGTACAAGACAAGACGTCTGGCATCTGCTCACCTGTAACTATAAGTCTTTATGTAGATGCGTCGTAGCGCGTACAGTCCTACCGAGACTGCATGCAGCGTGCGCAGACATTTTTCCTAGACCCTATACCTGTTAAAAGTGTGTCCTTTTACAGTACATGGGAGGATCTGTTGGCGTGGAATGAGTACTGTATTCATGGCAACAAGCTACAGCGAGGTGGCAATTACCTAGCGTTAGCACCAAGCAAACGATGCCACTGATGGACACCGATAAGCAAGTACTGATGCAGATGAGCACACCAGAGGCTCAGCCACATCGAGGACAGCGAGAACACCGTACATGGGTCTTGTAATGAAAGGCG

>TU4234-GaLu96scf_30-298140-298515 ACAGACAATGAATTCGCCTTCAAAAGATTGTGCAAGTGGAAATCATTTACACTCTCGCAGCCTGGCAGGACACATTGCCATCGGAGCTCCAAGCCTTGCATCGAAGCTACGTTAGTGCGCCATCTGTACAGTAAGCCGGCGCGTGTGGCCTTTGCATGGCGAACTCGCGCCCAGCACTGTATACGTACTGCATCAGCAATGAAAGCGAGCAATGAGCGTAAGCACAGGGAAGCACGCACCGGTCCCCGAGTCGGCATCGGACCTGTGCGTGCGCGCGTCGGCGATCTACCGTACAGCGAAACGGACCCCGATGCACGTTATTTTGAACATCGAGCGGGCTCAGGGCGAGTGCTGGAGACCGCTGCTCGGTAGTGCC

>TU4240-GaLu96scf_30-311396-312235 GTCAGATCCAGCTGTGGCCCACCAAACACCGAGGCTCACCGAGCTTCGCCGTTGAATAAGCTCTATATGATGAGTATCTTTTCAACCTTGCCTCACGTACAGATACATATTCCATGGAGCCGTTCTATGTGATTGGGTTTCTTCAACGGTCCTCGTCCTCGACGCCCACTGCATGCCGCTGCCGCTGCCACCACCGTGCTTCTACGCCAGCCCGAGGTCCTGGCTCGCGCTCCGCTTTGGACAGGACAGGGTCGAATTCGAGCACTATTCATTGGTACCATCGTTCTGTATTATTTCGACTTAGCGAGATGGGCTCCGAGTCGATATCGGTGGGGCTTCTCCTGCGGGCTCACCTCTGAGATCTTACCGCTCAGCGCTCAGCAACGTGCGGCGATAGGCAGGGGTCCGCGCCCGTCGCAAGGCCCTGCTTCTGGCCCGCGCGCACGTACTCTCCGCGAGCTCGAGATCGCGAGCCGTCAGACTCATCGTGGTGGGAGGACACGACATATGACATACTGGTAACGGCATACTTGCGGCAGCGCTTGTCGCAGTCTGGCAGCCCATTGGCTGATCTTCCCCCTTGTCTTTGTCCTCGTGGGCGAATGGGAGGCAAAGCCGATGTTCGCAACCGACAGCCCCTCGCGCGCGAGGAATATGTTCCTGCAATAAAATTGTAAGTAGTAAAGATGCTCAGTCTGGATTTCTCCTCCGGGCCTGAATACTTCCAGCAAGGACGGTGGTGATACAGGGCGCAGGACGTTGATTTGTTCGGCGTTCGTAAGTAGAGTCGACCCTCCCTAATTGGCAGAAATTAATCAAATGCGCCCATCTCGTCCTCGT

>TU4247-GaLu96scf_30-346543-347399 GATTGTCAGCGGCCCTGCGTCTCTTCGTCGTCATCCTGGCCAATTATCCGCTTCACAGTACGGCAGTACTGAATAGTCCCGCTTGAAACCGTCTCATAACAATGCAGTGTACTCGAGGCGCAGGCCAGCTACAGCCTGAAGCTACGTCGATCATGTATCGAGCTCATCTCTCATTCTCGAGGAACTATCTGCGCTCTCCATGTCCCCTCCTTTGCCTTCGAATTCGCCGCTCACGTCCGACCCCGCGGATGCAATATGAAGGGGCTCGCCGAAAGACGCGACGAACCGCGGAAGCGTGCTCCTGGAGCCTTGTGCCGCGAACTGTACCTCTCCGCCAGAAGGCACCGCGGAAGGCGCTCCTTTTTGGTCGACTTGGCGGAGGTTGAGGATGAAGCGGGAGGTGAGGATGGATGAGATGCTGAGAGACTCGCGGCGATGGTTAAGCTCAACGGACTCGGATGCGGAGGCCTGTGAACTCACGGGTCAATGAAGATCGTGACATAGCTTGCTTTGCTGGTTATCGAGTCGAGCTGCGAGCCGCGGTTGAAGTCAACGAACACAAATATTAATATTTTCACGAGGTGCCACTTACGGAAACCGCGGTCAGGGTGACTTGCAGGACGTTCAGCAACGCCAATATGCTACAAGTTCGACATTAGCGGACGGTTCTTATTCTTAGCAGAGGCACAAAGGCACACATGAAATACAGGCTTCCTATGGGTAGGATGTTGAAGTAAGCTCTGAGGGGCGATGTGGACCGGAATTGCCACGACGCACCATCGCGCAACAAGACACTTCCCAGAGTCTCTCCCAGCTGGTGATCCCTCTGAAGCCTACGTACTCTGTATGTGTTCCGC

>TU4249-GaLu96scf_30-351777-352361 GCCCTCCCGAGGTTGTGGAATGCGCAAGCCGAGATATGATGCGATCAAGGGGCTCAAGGCGGGTTGAGGACACATATCGGTAGCAAAAGTAACGGTCTAGTGCCATATGCCGTAGTAGAGCTGGGAGATAAGCCGAAGCGGGTTGAATACTATGCCAGCTGGTAAACCGGCCGGGGGCCGCGGCGCTGAGAATCACGAAGTTCTGTGAAGTTGTTCGAGCCACCAGAGTGAAGTGAAGACATATTTAGAGTAATACTCGGTCTAGTACACTCACCGCATCTTCCAGTGAGTTAACCGGTTGACCTCGAATTCGGCATCAGGGATGTAGGGAACAGCATGATCGCGGGCTGCGGTGGGAGAACAAACTCATATCATTGATGACGTGGATCGGCACCCGTTTTGGCAGTTTAGACGCTCGTTATCTGGACGCCTTGGCAGAACACATTTCTTAGGAGCTTTCCCACACCTCAGCATCTGCGTTCTCCGCCTCGACGACTAAGAACGACCCAACTTGAGTCCCTTGCGGCGCATCAGATCCGAAGACCTCAAAGGGCTTGAGGTCGGATTCGAGTATCGCACTCGCCG

>TU424-GaLu96scf_1-3435530-3436280 CTTTGCTCCCCAACTTCTGTAGCATTCTATCTAGACCAAGCACAGGCCTAAACAACTCTCCGACAATATTTACAACTCAAGACACCGGTGGGCTCTATCACAGACGGTCTCAGATCCATCAGAGACGCCCGAGGTCGATCAGGTCGCTGTATCTCCCAGGCAAAGGTATGACGCATGATGCATGACCTCGGACGCGTTGTCTCACTGCAATACAGGCTGTAGGTCGCTGGCAACGTTCGCAAGATTGCCGGCCTCCGTACCTTGTTCGTCAGTCACGAAAGTTCAACAGAACCACAAGGTCATTCCCCTGCATCTTCAAAAGTTGGGTGAATCAGCACGAGCGGTGTGCGAGTGGTCTGTGGTCAGTAACTTTGACTGTGCTGGGCGCGGGCGGCATACTCGATAATAGATGGTCCAGCCACAGGACCTTGAGGGTCTGGGCAAAGGCACGGATGGTAACAGTCAATTGCAACCGACTTTGTCAGCCGAAGCTACTCGGGGAGTCTAGGAAGGTTAAGAGCTCCGTGTTCTCTTAGTAACGTTTCCCAGTGGTTGCGCCCTTCCGTACGGTTATTCGCTTCCCATTTCCAAGGTAGGCCGTTGCTCGTTACCATCCCAAAAACTTATTCCGTCATACTGGGATCAACGGCCGGAAGGTTGCGGAAGAAACGACCACTGTATGTAAATCATTCGCAACGGCGACCGGTGCCGCAAGTTTGAGATGCGACGTTCGCAAGGATGTCGGCTTACC

>TU4259-GaLu96scf_30-418575-419251 ATGCCTTCCGTTACACATAGCCTCTAGCATGATATATGCCTATGTATAGGAAACTGTGGAGTATAAGTGTTCCACGGCGGCTCTACCTCGCATAAAAGGCCCTCGCCTCGACGCACACAGCCGTTCTTGCTTACATTCGCACCCGACACCATGTCCTCTATGCAAGTCAGTGCCGAGGACGTCTCGATTCTGTGAGTCTAGCTGCAACTACGATTGCGCTTGCGAGAAACCTGACACGACTGCCGACTCCGGAATAATAGTTATACCTGTGATGATATCCTTCAAGGGCTCAATGAGAGACAATTGGGGCACGGCGACCCAATAGCTGAACTCACCACGAGTATTATACTATTTAAATGTGGACGCAAAACCCGGCAGGAAAAACGACCAGCTTGACACTATTATACTATGGTCTTGAAGTGATAGTGTGGTTTGTTGTCAACGACTCTGACAATCTGCAAGCTTTGTTAATGGACGGCGAGGCAGGAAGGAAGAAAAGAGAAGAACATGCTGGGGAGCGACGGTACATGGCCTAAACATTTTGCGAGTCACCGAGCCCTGACTGCATGAGGGAGTTAGTCCTCGGCAATTTCCTAGCGTCAACTTGGCACATAATTGCAAGTGCTCGTCCTACTTACAAGGGGGCGCAACAGTAACAGTTTTATGCAGCGTGTCCA

>TU4263-GaLu96scf_30-432199-432888 TGCCGTTGGAGAGAAGGAGCGAGCAGCTGATAGTCCTTCCCATTTTGACGCCTTTCCGAATCCAATATGACTGGTACGTATACCACCATGTAACACCAAGCACAAGTAGCTCTGCTAAAAGCTGCGATCCCCGGGAAATGACTGACACTGGGTTACAGAGGTGAGCACGACGGAAAAGGACAGTAAATTGACATCCTACTCACCACTAGAAAGAGTTATCAGTTCAGTACGGTCGGCACACAAACGCGAGCTATTCAACGCACCGAACAGATACCGTCCGAGATAGTGGAACTGAGGGGCTACAGTTGAATGGACTAGGTAGGTCTCGAGGAACCGTGTGGAAGGACGTTGGGTATCGCGGGGCAGAGAAACATTCGGTTCGTGAGACCGTGACACTCTACACGTCGGAGCAGCCAACCAGGGTCAACAAGTTGAAGACATTTGATAGCCCCGATCAGGGCATCTTGCCGCATGGAATGTCATGTCCTAAGAACGACAGTGGGCAGCGGGAGGGGGTCAGCGGAGCAGACCTCGCGATCGCGGTTGAGGGTGCGGGCGGGGTGTTGAATGATTCAGACGTACTACCTCTAGCTGCGGCTCACGACTCCAAGACACAGCTGGTAGATATAGCGAGTAGTAGTCGCACAAAGACTAGTCTTTATATAATCGGGAGTAAGATACTCGGGCCTC

>TU426-GaLu96scf_1-3438716-3439249 GGCGCTCATCACGGTAGATATTAATATTGATACCGAGAGAAGACCTCTCACCGACTATCGACTTTAAATTTGGGTTCCGACCTTGGTAGGACGCGGATAATTGGTGTTTTTGTGGATTGAGTCTAACGACTAAATCCCGGATCTGCTACGTTTAGCGCGTTTGTTGCCGCTGAGAGCAATAAATCGAGACAAGCCCGAGAAGGTCATTCTTCTACTAGTCGCACGATTTTGTTAGCCGCGGATTATGACGCGCGAGGCCACTACCACCGCCGACGAGCGCACATTCGGGGGCACTTGGCAACCGCTGTGGTGCTGTGGTGGGAACAAGGACGAGTTTCGGAGAGACGTCATTTCCTCCAAGGTCTCTGCGAGGCCGACCGACCCGAAATGGGCGGAAAGTCGCAGAAAGATGTTCTATGTGCTCTCGGCACCGCGCTACAGCGACCGGACGCCTCGCCTCCCTGCTCGAATGAAAGGCTGTCAAGGTTACGAAGCACGAATCCCGGAACATGCGTAGCGCGCTCCCGAGGGTTG

>TU4276-GaLu96scf_30-460947-461356 TGGTATTGTTGAGAGCGAAGGCGGACGTGCGCATACAATGACAGGGGGAAAGAGAGCGTACATAGAAGTGGATTACTCACCATACCCTCCTCAAAATGCATCGAATCGCCTGCAACCTCCGTAAACCTGCTTGTCATCCTCAAATCTGTTCGGGGCGTTTCGCGAACTATCCCTTGGAGCGGCGCGATAGGGTATTATGGGGCATAGAGCCTTTTTCGGTGGTAAGAGAGACGGTGCAACGAGCAGAGACCTCCCGAAGGACGATTGTCGAGGACCCGTCAATAGCCGAATGCTTAGAGAGAATTGCACAAGGTGCATATAAGGAAGCTAGAACGACATTGATGTTATGAAGCGGCGATGAGGATGCAAGATCTATGGTTAGAGCAGCGTGGAATGCCGTCTACCAGAGC

>TU4288-GaLu96scf_31-11133-11833 CCCCGTATCCCACACGTTAGCCACCAGAACGGTCGCCACCTGCATGCGCAGTGTTTCAGAATCTTTCCGTAGACAGTTTTCAGGAAGCTGGCATACTGTATCTCACGTTGTCCCCACCGACCTAAGTATGTCCAATCCTCCACCATCGAACGCAGGTAAGCTGACTGTCAGTCCCACATTGCATTGTCAAAGTATGAGCTCTGATTCTATGCAGGTGTGACATGCATGCACATCGTCGGTCCATGGACATCATGCATCATGCATTGTACGCGCGTGAGGACGCGCGGGCCAGCCTGAGAACGTCGGGCGCCTGTGGCGGAGAGCCCAGAACGGCGCTGGCTATAGTCTATAGAGCTACTAGCGCGACGCCGACAAATAGACCTTGATCAACCCCGCATTGAACCCGACCAAAACCAGCCAAAACCCGACCACACCCAAGGGACCCAAGGCAAGTTTCTCGATCAAACAGTAAGTTCGTCAGCCACACTAACCATAGTGGTAAATTCTAAATCATGGCACAAAGATTCGCGCCGGCAGTTGAACGCGTCAATAGTCCTAGTCTTGTCTGGACACTGGACAGATGTTCGTCCAGGCCAGGCAGCAGTGTCAATGTTACGTTGTACCTTTTACTCAATTATGTACAACCAGAACAACCCGGATCCACCCCGCGGCAGGGTATTCCATAACGACGCAAGTAGATC

>TU4292-GaLu96scf_31-41214-42005 TCACAGAAGCAGCATCTACGGATGCTATTGGTTTAGGAACCCGACCCGCCGAGTTGCCATGTGTTTCGGACCTGGGCACCCAGCGGTGTTGGACTCCTTCTCCATTCAACGACTCGTTCTTACTATGCCAATGAGGATCTCGCCCGGTATCCATACTATGTCGCAGAAGCATGACCTTGAAGCCAGGCCCGTATGGGATACGGCGGTCTCGGTTCCAGGTACGTTCCCCACATGGCTCATACCACGCCCCTTTTGCGGGCACTTCTATCCATTGCATTCCTTGCCTATGATATGCCTGCCCAGGGAGGTCGCCACAAGCGCGAAAGGATGATGAAGGCAGTGCCTGGCTCTGCTTCCCCCCCTTCAAGTTCGCGTCTGCTTGCTACTAGGCTTCGCATTGCCTTGCTACCACCCACAATAGTAGGCGTGTAGTCCTAAGCCTCGCGTTTCGCACTAGCCATCGCATCTCAGCGGCTGGGAACGTGCAGTCGATGCCACAAGCGCAAGCAGAAGGTTGGATTGCTTCCCCCTAGCGCTGTGGAGGGCTTGTGGCGCATCCTCTGTTTCGAACCCTGTTCTATGAACCCGTTGGAACGGTCATACTGCGTCCCACCGTCTTCTAATACCGGTTTTCATTGATGCTGGGAATGCCCGTTCAAGTTGGTATGAGTGTTACCTTATGCCTGACGAGTCCGATGGCTTGTTCGGAACGTCCGACACCGGTACAGCGCTCGGGACAGGTGTTGAAATTGTCCATATACCTTGATATACCTTATATCCCTCAGATAAATG

>TU4304-GaLu96scf_31-132619-133140 AACGTGCCCGTCGATTCAGAACGTCCCTGGGAGCCAGGCAGCGGGTGCAGATCGCGGCCTGCCGCACGGGTGAAGGGCAAAGAAGGGATGGAAGAAGCAGGCGTGCTGGTGCAGGGGACGGCCCCCAGATATTTTCTGTGCGAGCAAGATTGACGTTATGACTTCATATCCCGCCGCCCCCCCTGCGTTCCTAGTCCGGCCACATGGCGCACTTGCGCGCGCGCGCGGGACGGCTGAGAACCTGCCCAGGGTGGCGGACGTCGCGTTCTCACCCCCGCGCTATACGCGACGCCAGCACCAGCACACCAGAATCTTGTCTCTCGCCTGCCATGCCCATGCACGCAGCACCAGCACCCCGGGCCCAGTCGGGCCTGCGGTGATTCAGCGCAGAACGGGGAAAACTTAGCAGCAGCACAGGAGAACGCGCCCCAGTCGCCTGCCTCTCGGCCTTCGCGCTCAGCAGTGTGATGGTCTGCGGATCCCCTCGCGATGTGCGCGAGATGTGGTACAGGCTCCTGCCAG

>TU4312-GaLu96scf_31-203409-204043 AACAAAAACCGGCCTCTGCACGACCCGCTCTTGTGCTCAAACCGCCGACTACATCCCCGCCCCCATCTGACGCTATCTTTTGTTCTCGGCGTATTGTCTCCACTAATGTCTTACCGTGTATGCCTTCATTTCCTACAAGTCACCCCACCCCCATTGGTCCGACCGCCTCATGCTCGCATAGACTCCAGATAACTGGATGTTCGACTCTTGTACGACCCATCTCCGTGTCGCGCCCGTTTTTCCATGCAAGCTTCCGACCGAAAACAAAACATCGTTCCATCTTCCGGCGCCAATCTTTCTGGTAAGGCGGTGTCCCATGCGCCAATGATCCTCGAACTCCGACGCTCCATGCATCCTCTCCGTTCCATCCCATCCGGCGATGCGTCAGATCCTGTTCAATTTAATCCATCCCACCTACTGAGGATCCAGCATGCCTCGTCGAGAGCTCTGCTTCCGCTGGAAGACATCTGAAAGGTGAATGGTTTTCGTACAGCTTCCAGCCATCCAGGAACACCGATGTCCTGAGCTTCGTGATTTGTCCGCCCTACCCGACGGCCGAGGTCTGGAAGACCGCAACTTCCCCGCTCAACGTATACCGTACGTCAATACCTATACCTTCTCATCCAGATAGTACA

>TU4313-GaLu96scf_31-204927-205281 ATAGAGCACTCCATTACAGCAATCCAAGTTCAGCGGCGTTTAGCGAGCGGTCTGCCGACCCCCTTCCTCCATAACACGCTCCATTCTCTCGCGTACCCTACCCCTCCCCGCGCCTTGCGTTTCAGACCTAGGAAACGCGCTCCGAATCCGCGTTGCGAGCCCCGTAGCACGGACCCACAAACACCTACAATAAGGGGTCAGTGTTTCGCCTTCGTCCGCCTGCCAATTGCTCGCGTGGCCTTGTTCGAGCGATCGCGTCCGATCGACCAGGAACGGGGTCATCCCACGCATGCGCACACGCCAACGATTGTTAACGTTGACGACGTGACAATACCTCCGGACAATAGGCCCCTAT

>TU4314-GaLu96scf_31-206580-207165 CCAATATCTCTGCCATCTAATTGAGCAACGCTTTCGCGGGAGGTTCGCGCGTTTCGTCTTCAGATCCAGCGTGCGTGCCATACGTAGGGTCGTCGACCTCGTTTCGCCATGCTGCTGCGTACGCCTCCTGGACGTGCGTAGTTGTGCACCAGTTTCACCAGTTTCGAGTCCTGTCCTGTGCTTACATTCTGCAGATGTCTCGCGTGGACTTGGGACCGGCGCCAGGTGATACGTCTCGAGCCCACCTTTACCATACCGCTCGCTGTTGACGCAAGGGAAGACGTTATGCGCCTGACGTCGACATCGAGTGTCCGATCCATTCTGGCAGCGTTCTCGATCCTTGGTGGACCTTGCACCGCGTCAAGAGCTGTCGAGGCTAGGGTTGCCCGGTTGCGCCATCTTGCGCGTGTACCCCAGTCTTTGGACCTTGGGGTATATTCGTATAAATACGCTTTACCTTCGACGACGCTGGAGGGTCAGGTTGCCTAAGCTTTGCCCGTCTTCTGCGCCTCTCCTTGGAATCATTGCTTCAGAATTCGGAAGCGTGGGGTTTGGCGACTTGTCACCATCCTTCGCGTCCATTTCA

>TU4336-GaLu96scf_31-328224-328687 TATCTTCGCTCTCCTTCATCTTCTCCATCCATCTCTCACTGCAGTCGAATCGTCTCCACCGTCGTCCCCCGGCCTCGCTGCGATCACCCCTCCTGTTTCTCCCGGTACTTGAGACCCCTTTGCAGGCTCGACCAAGTCAATCAAGAGACCGCGTATAGAGTATATGAACGCCATGGCTCCCAGAAGTCCATTGGTGCGTCGATTGATGCGTCGAAGTGAACCTCGGCACGATCTCCGTGCTCACGCGACCCAGAGCAAGCTCCGGAAGCATCGACTTGACCGACGTGACCCTGACGGGTTCGACGCGCGTTGTTAACCCCCTATTAGGCCCAGCGAAGCCCGAATGGATCCCTCCGACATACGAGAAGAAAACGAGAAGAAAACGATAACGCGCCGCATTGAAGGAAAGCGCGTCGAGCGCGCCGCCCTCCCACTCTAGCTACGCATACGTCTTCGAGCGGATA

>TU4338-GaLu96scf_31-331412-332207 ATGGGTTGGGACCAATCATGAAGGGCCAGGGACTCCAGAGAGGAACCACATCTTTCAACTGCGGCAACGAAGACCTTGTGGTTACGAGGGCCCTTGTATAAGATGAGAGTCCTCAGCGATGCCACGGCGATGGCCTTGCCGAAATAGTAACCAGGAATGGCGAGAGTGTGAAGGCGGGGTGGATCCGCGTCCTCCCAGGACGGCAGCTTGTCGATAGAGAAGGGGGTGCCGCTCAATTAGTCTTGACCCTAGGGCATGCGTATGCGTAGGCTCTCTAAGTTCCGTATATGCGCGTGCACCAGTGGGAGAACCACCTTGATCCCGTCGGGGCAGGTCACTTCGACTGACAGGTGGGCCACCCTCGAAACGTGAGGCTTGAGGACTGCACTAGGGTCGAGCTCGGCGATGAGGCGCATATAGTCCGGGAGGCCAATAGTGAACAGAAACGGCTCCGACCGCGCGAGGAATACCGGTAGACACTGGGGCCAGTGGATGTGATCGCCACGATGGCACCACCTGCAGTGGCGCTGTGAATATAGATCGAACGGCTTCTGCCCACAAGTCCCGAGTGCGACGTCGCGCCAGTGCTGGCAGATGTGCGTAAGAGCGACCAGTTCCTGCCATCGGCTCCAAGAACCGCGACATTATTTCGACCACGAGTTCGGAAGGAAGGTGGTTGGCTGGAGCAGTGGCGTTGAAGGTGGACCGGAGGGCGACGACGGGTGTCCCGGATCCTAAGTTCGCAATGTTGGACGGGGATGGGCGGTAGGACGGGGTGTTCGGGCTCTTGGAAATA

>TU4368-GaLu96scf_32-18915-19379 CTCGAATAGCATTGGTGCAAACTAGTGTACAGACATCTAGTTAATTCTGCAAGACAGCTGCATACTACCGGGCATTGTAAAACTATGTACATGATAAAGTGAGAACGAGGGCATCGGGATGACACGAGCACGACCATAAAATGATTCGGTGCGCACAGCGAAATCATCCAAATATGGTAGATCTTGCTTCTGGAGAAATGTTGGATGCAACGCATCGCAAAAGCAACGATGCAAAGAACAAATAGGGGCAGGATAGAAGGAACGCCTGTAGAGTACGCACTGATGATCCCTTTTCCGTTACCAAGACGGGCGAAACCAGGCAGAGCCCGTGGAAACTGCTCCCAGCATGAGCAAGTATGTTTGTGCGATCGCGGAATATTGATACAGATCGGAAACTCCAGACTAGAGTGTAGAAAGAGGCGTCGCCGGGGGGAGAGGGTGGGTACGGAGTGGGTACGGTCGAGG

>TU4369-GaLu96scf_32-19527-20135 AAGACCAGACACCGTAGGTAGTGCATGTGAAGTTTGGGACGGACGCTGACGGTACCAGTGAGTGTTGTGAAAGAGCGAAAATGGTCAACTATGGACGGTTGGAAACAGGTACCTAGAAGCCGGGGGGTAGAGGCCTAGGTTCAGGAAAAAGACGGTATTGAAGGGTGCTGGTCACCGGTCAGAGCTGGTGCCGTGGTCAGAGTTGCCTGGCGTTGGTTACCGATCATGATTCCTGTAGGCCAAGGGATGGGCTGTGCTTGAGACGAAATGGGCAGCAAGAGCGGAATAGTACAGAATCCAGGTCGATTGGCAGCAGAGCAAGGCAGCTATCAGGATGGAGCTCGCTAAGAATCATATACGTTGTCAAGAGTCATCTGAAAACCGGGCGAGAGTTTGTGGATGATTTCCTGGCAGGAGCTAGTACTGGTGATGTTGTAGAAGATGGGGTCCGGGATGTTGTGTGATATACTCGGACCAGCTGGCAGGATCCATGGTCGACTTGGTCGGACCTAGCTAGCCTGCCATTCGCCAGTTGAATTTGTGAATGGATACATATGGCTATGAGAGAAAAGGGGAAATGGTCTAGATATTCCGGAGTCCTGGACGTGG

>TU4387-GaLu96scf_32-188709-189778 CCAGCTGGCTCGTCACCGTCGTGCCCATGGCCAGTCGTGCCCCAGGCCTATCGGATCTCGTCGACGGCGTCGCGCGTGCTATTCTATTTCTCGGGCTCTTGCTGTCTCGCACTCAGTAACAGTTGCCGTTGCTCTGGTTTCGATCGGAGGTTGCGAACCGATCGTCCGAGAGCGCGTTTGCAACCGGCGGGGGTTGGGCGGGGGAGCGGGAGCGGGGTGACTGCGCAGTTTTTGCGACGTCAATCGCGAGAACCACCAAGGCGGCCAAACAATGCAGCGCACGCCATCGTCCACAGGTCTCTGTGCATACAGTCCGGAGTAAGCTAGTAAGTTACGATTTGTACACGGTGTCACCCAATACCACAATATTCACCGGTCAGTTCCCATTAGAGACTACAACGAGGTGTGCACACCTCCCTCGTTTCCAAACTGCGGCCCAAGATGCGTCTCTGTCGGCAAGTAGGGTCAGCAGCTTCGAGTTGCTCGCCCAATCGCCTTGCCAAGATTGACTGCACGCAGGTCACAGATTGATCTAAACTCTATGCATGAACGCGGTGTCAAGTGGGTCCCATACGAGGCAAGCGATACATTCCAGAAGCATCCCAAACCTTCGCCGACGAATTGAAATGAAGTCTCATATCTGGACAGTGGACGCTCACCTGGATTCGAGGGTGATCGCCGTCGGTGGATAATATGGTCATGCGTACGGTGTCGGTGGAAGGCAGACAGAGTTGTGGCTCTCACGCATCCGGAAGTGGGCGATATTATGGTCACGGTGCTGGAACAACCACCTGGTCCCCAAAGATGTCAAAAACGTCGTCTCTGGACATGGCATGTGAGCATGAGCATTATGTACCGACCATGCGGGTGCTCACCAGGGACAGCTCACCGCCATCGTCGTCTCTTTTACACGAGGCGAGGAGGCGATAGCAGAGAAGAGCGTACGTATCGTATCGCCCAGGCCGCCGCGCGCGCCGCCGTGCACTCGGTCCTACCCAGGCGGCGGGTGGCACTGACAGTGACAGACTGACGGTGAGAGACTGCAGCTAAGTAGCAGAACCCAATGCAAA

>TU439-GaLu96scf_1-3538042-3538578 GACTCGTGCCAAGAGCCAACAAGTTTCAACTCTCTCTCCTCTCCTCTCCTTCGCCGCTCTCTCCACGCCTCTAACTTCCCTCCCCCCGGTATTTCCGTTTCAACAAAATCTTCCTCGAGCAGCTTTCAAATGTTTTATCCGAGCAACGCCGTCCAATCCCACATAAGCATCCGACTCCCATGGCCCTAGTCCCACACGAGGTCCTACGCACCGTACTTAGTGTAGTCCCGATTTCTGAATATCCGCATCTCAATCATATCAAACATCTCAAGAATCCCGGCATTGGTCCGCTGTCGTGCCCTGTCGTATGCCATGTCCGCATTCGTCCATGATGTTCGTCAAACCAACGCCACATCTTGGCGATTTAATGAACTTTGCCCGCAGGTCGCCGCCAGGTGGTCGTCGGCAGGCAAACCACTTCGTTCAATCGGCCGCAAGTGCACGCGGTCGCCCTGAAATCCCGATGCGTGACATTGACAGACATTACCCTCATCTCGCCGCAGTGCATCTGTAAGTATTTCCAGACAGACACCACCA

>TU4400-GaLu96scf_32-239298-240195 CCCCCAGCACAATCTGTGAGCACAGGGCGTCTGGTGCTCTCAAATTGCGGTACTCCGACTATTCAGCCGTACCTATTGACCCTTGCCAAGGGTGCTACTTACAGCCCCTTCTCGCGCGTTTTTGTTTTCATTACAGGGCCCGAGCGAGCGCCACGCATTGCGTTCTCGCACCGCACAACCCTCGACAATCCGCCACCCGTCGCCAACCGCGATGCGTCACGTAAACGGCCCACCGCTTCTTCTGCATTTCCATCCTCCCATCTGTTGGTGTGTGTTCCCTCCACCCTGGACTACCGGGAGCTGTGTGCCTGCTGAAACGCTCCCTAACAGGAAACTTACACCCCTGTCAATTGCGAATTGTGGGCGACGCGCCTACTAGTGCCTTTGGTGGCGCTCCATCGTCAGGGTGTTGGCAAGGCACTGGTATGGTATGGACGAGGGCTTCCTACCTTCAAATCCTCAGATTGAGGCGTAGGGCTCGCGAGTCCCTTCTCTGAAGGTGCGGCAAGGACAGGGGTTCTGTGCCAGTCATGGAGACGTTGTCTGGCACCCTGGTCCGCGGGCCGAACCGATCTCCAGCAGCAAGGCGGCCGGCGGATGGTGTGCCGTTCGTCAGGCATTCCCATGGGACAGAACGTTCTGCGTCCTTGACTACGACCTGCGCCTGCGAGTCCATGGTGGTCCAACTGTGCTCGACGGCACGGCGCAGCCGCTCAGTCCATTTCCGCTGTGGCCCATCACCCTGTTAGTATGGCTGTACAGCTGCGTTGGCAGCTGACGGCCCTACGGCATGCACGGAGTGGTTGGTTAGTGTCAGTGATTTTCGGTCCCATGTTCGCAGCGCGTTCGTCGCTGGATAGCACGCGCAGCTCAGTTACCTAAACGTATGGCTGGGGTT

>TU4411-GaLu96scf_32-347906-348918 GATGATCATCCAATGCTGCGTCCAAGCTGCGTTCACTGCATCCCGAGCTCTGGCGACATAGCATTCGGCGCATCTAAAATGATGTCCTTATCCTTCAACGAATGTCCGCGACACACGAGGGTGAAATGTGCCCGACGGTCCTCGTCCCCGTTCGGCGTTGCCCGCTAGTAGGTCGATTGACTCGGCGTGCAACTTTCACAGGTAAGCCTCTTATGTTGACCACCTTAGGCTTACCGTTTAAAGTCATGCCAAAGACGGTTTTGAGCCCGATAGCTCAGAAGTTCGACGCTTGCTCCAGCGGTATCGTGATACACACTTCCGAATGCTGCACGGCCACACGAACTTGAACGTTCTACTCTAGGCTTAACCACTCCGAAAAGGGACCTTGCTAACAGAATTAATGCTACCTATTGATAGTAAGTAATAATAAGCAATAGTTTCTGACAATTGGCTAAAGGGGGTTTTTCCTGATGACTAGCGCCATGCAGATCTTCCGGACGTAACAGCTACGAGGAGCACCTCAAGACTCAAGTACACTCTCTGCATTCCATTCACAGCTCCGACTGTGACCACGCCGGTCGCTTCAAGCTCGCCTTGAATGTCTACGGGAACGAGGACATCCACGCAGAAACTTAACGTTGGCGCTATCATTGGCCACGTCGTCGGGGGGCTTCTCTGAGGTTTGGGCCTGATCGTAACGATCATAATGTTTCTCGTCCTCCGGCATCGGAGGCAAGCCCGCAGAGAACAGAGCTCTCCAAGCAGTTCAACAGCTGTCCCATTCCGTGAAGGGTGCGGGCAGGGGTTCTATGTGCGTGGCCCAAAATTATCAAAGCAAAAGGGGCTGCCGGTGACACGCTATTTTTGATGTTCCGTAGGACCCCGACGATATCCTTTAGAGCTTGATTTCTGCTAGGGTAGCGCCGGGTTATACAGGATTTGCACAAATCATGTCATGAATCGGGTCCACTGCTCCGATATCGCTCCTTAGCTTCTTGCGGGCTCAGGAAATT

>TU4412-GaLu96scf_32-349092-349585 TTCCGCGCACTTACGTAGTCTGTTTCACTCTCGCATTCCCGCTTTCTACGTCATCGATCTATTAGTACTGTATATAAGCGCGTCCTAGTATGTAGTGTCCCAGGAATTCTCGTAGAATCAACGCATTGTAAGTAAGTACTATCGCTTCTGTTCGCATATCCTCGTCGACGTCCCTCGCCTGATCCTCGTCTTTGTCAGCCTCGCGCGACATCAGGCTTACAGTTGCGCTTTTGCTGATACAACTCTTGCTCCAAGTTGCACCTCTTCCCGTTCCCTTGCTGTGTTTTCCTTTGCCCGTGAACCCTCACGCTCGTATTGGGCCGGCGAGGTATCTGCTTTAAACTGGCTCACCTGAGCTATTTTCCTTAGCGTAGTCGGTTCTATCTAATCGGTTCTTCGTTCTTCGCCATTCAAAACCCAACCGTGCCCCTCTGGCTGACAAGTGACCCTCTGGCTGACATTACTCTGCCAACTGCGTCAACACATCAAATATG

>TU4415-GaLu96scf_32-356310-356570 CAACGCAACAGGCTTCAACGTTCAACGTGGAACCCGGGAACCGCCTCCTATGTTGAACACGTCCTGCTATCACTCTCGAGCTCAGTCTGGTCTCGTATCCCTTCTCACTCATGCTGTTGTCGTGTATCCTGAGATGGCCTCTACTTTTTGCATCGGCGCAGTGCCGCAATTGATGCGAGCGTGCCATACCTGAAACTCGGTGTTGCCTACATCCTACAGCCTGAGTTTCTGGATGCTTGTTCTGGCGTGCGGAGTAGTAAG

>TU4427-GaLu96scf_33-2407-3032 GCGTAAGGTCCGCGTGCCACCCCCAAAGTGCCTTGCCATACACGGACGCTCATGTCCACATATAGCGAATGATCTCCGCCCTTTTCTCTTGAATGAATGGGTGAGAGCCGGTACGCATAGTAATGAAACACGTACGGCGTTTACGTCTACTCCAAGGTTCACTCTCAGGCTGACAATCCCCCACGTACATCCCGACGTCAAAAGGTTCCGGTCCTGTCTACTTAAGTCTGCATTGACCGCTTGATCCGGCGGGGGAAAAGGGGGAACGAAAGTTGGTACGCTTCTCCTAGAAGTATTCATCGGAGGTCAGTTATATTTGAGGGTTGGACGTCACTGGATGGGCGGGAGCGAGACAATCGGGGATCGCGGGGCTTATCTTAAAACCCCATTCAGTGACATTCGCTGTCGGAGTCCAGAGGCTCGGGGTCATTTGTGAGCCATGAGCACTGAGCCGTGCAGAGTCTAGACGTGAGGTGCAAATATAGAGAGAATGCGGGGGTTGCGGAGGTGATCAGAGTATCCGAGTCGAACATCGTGCCGCCTGCGGACCTGAAATTTTACTGAGTTGCACCGCTTCGCCGAGCACAAACCAGAAGAGCTTTTATGTGATGTATAAACGCAGTGAG

>TU4428-GaLu96scf_33-3257-3767 TTCCCATGCTTCACCTGTTCAACTGTCCTCACTCGTAGGACCTGGTGAACAAAGGTATGCGGTCGAAGTGCAACTTTTCTCCCTCACTTAATGATTGAACCCTTCCATGGCGACCGTCCACCAGTCACTCGCATTGGCTACTAGGATCCGAGTCACGACTCCAAGGGACCCTCGCAGCAGTCTCCGAGACCATAATTCTTCCATCCCCAGACCAATCTCACACCTTGTTCTTTGACACCGGGTGGGATCCTGGCAAGTATAATCCCCGCAATTTCAGGTCCAGCGGCTCCTCCGGCGAGGGCCGCACCTCTAGTATGGACAAGCGGAAGCAGCGGCGGAGACCGAAGGCCCCATGACCAGGCACAACCCACGGTATTTGGTTTCATGGTGCACCGGCACACGGAAGCCACCACGGTCAAAGGGAACCTCCGAGGGTCGCTCTCAAGCTGCCCTCGTAAGTACAGACAGGTAGCAGCCGCGAGCAACACGTCGTGGTGTCTCGTGGCACCTG

>TU442-GaLu96scf_1-3545572-3546440 CCAGAAATCTGTGTCGTCTACACGTGGATCACGGAGTCCTGAGATCGGGTGTCTTGCGTGTCTACCAAGGTCTACTTAGCAGGGCACAGCCTGGGACTTGACAGACGTCGACAGTTCTCCTAAGTTACATAAGTACTCCTCAGGGACACGAAAACGAGGCTCGATCCAAAGCGGTGCGGCCGCTGCCATCGTGACATCACAAAAGGGACGGCTCGGAACTCGGGAATGTACCTACAAAGCACTAAATCAGTTCCAAACCAAGATGAGGACAAGCCCAACGCTCCAAGTCCGCATATCGAGAACAAGAGAGATGAAAGCACCAACGCACCAATGACAACATACGCCGACTGATGGACCACAGCATCTCCATCTCCCGTCCTCATCGCCTTGCCACTCGGGTGCGCCCACATCTGAGTCGGCCAAGATCGGCCATCCCTTCATGGGCTCGCGCAGGGGTTGAAACCTGTTCCCGCCTGCCTAGCCAGGTCACTGACTAAGTAGTACTGTAATAGTTGGTGGTACGTAGACCACCGGCAGCCCGATCATCAACGTCTCGGCCATCCGATTCACACGGCGCGTCAGATTCCCACTACCTTGAACATATCACCCTGACATACTGGGGCATATTGTCGGAGACCGGGAGGAGTTTGAACATGAGACGGTCGGCGCAAAGTTTGCCAAGGTTGCCAGCCGGGCAGGCCGGGGCTGGTCGTCGGCATCCACTGATCCAGAATTCGAGCGTGGAACGAGCCTTGGACCCCTGTATCGTCACCTTGCGCTGTGCGGAATAGAACGTCTTCTTCGCCGGCGATATCGAACTACCTGAGGTGCGCCAAGCGTTCGCACGGATGACCTACCCACGATCCCCC

>TU4433-GaLu96scf_33-42159-42747 AAAGGGTAAAGCGGTTCTCGTTGCGGCGTCGGATGCTACTGGTTAAGTTCGTGCCTGGTGTAGCGTAACCGATGGATAGAAGCGCGAATCTCAGCGGTTATAGCAATCTAAGACCGATACAGTGGGTGGCGATTGCAGTATGTACGCATTGCCTACAATTGGATAAACCGGGAAGTACACGTGAGAAAAGGTTAAAAACGAGGCTCCTGATACGAATCGATGTTGTTCAATGGCATTGAAAAAGCAAGATAAGTACTTGGCGCATGCCAGACCAGATGACCTGTGGGTCCATACAAGCCGAGGTGTCCGAGAACCGAGAAAACCCACCCTCGCCGTTTTCCTGCCGTGGGCCTTTGCAAATTGACACGCTGTTGACTGGCATGATCCTAACCGTGGTATCCGTTGTAGTACTCATTGAACCTTGGTGAGGTATTCGAAGTAGGTGTGTACAACCGATGATCTGGGGATTCAAGAACGATTAGTAGACGTAGAACTTCCGGCCGTGGTAGGGAAGTGAGTAAGGTGTACAAGTCAAGCACTCCGATTCGTGGGTCAGTGAATCTGAGGGCCAGGTGAGGGCATCGGAGTC

>TU4452-GaLu96scf_33-108822-109336 TAGCCCATGTTGTCCCTCTCGCTCCATCCGCCTCCATGCCTCCGCTGCCGCTACTGGATTCGTGTACTGCTTTGACGAAGATTTGGTGACGGGGAGACCTACATCTCCTTACTCGTGCCCCTTAATATTCGGGCAGTTGGTCTTCTCATGGTACGTTGGCCTGAATCTCCCGGTGAACCATCTTCGTCTCTTTCGCAAGCTGATGCAGGATACGTAGAGGCGGGGGGATCGTCCTTGGACGGTCCTGGATAATGTGCTCCTGAACGAGTTCCCGCTCCCGTTCCCGGAGCAATTCCTCCAGGGTTCCAGATCGCGTCCGCCGTCATCCCCCCACCTTTGGCCTCCCTAGGTCACTGAACGGCGAGGTCCATGCAGCGTCCGCCCGCGCAACGCGCCTAATATGGCATGTGATGACTGGGATATCGGGCCTAGGCTTGCTCTCGGTGCTCTTCATGCGGGAGGTTACTCTTCGCGACCATAGGGATGCGCAGTAGGGATTGCAGGAGGAAAAAAAA

>TU4457-GaLu96scf_33-120892-121162 GACTTCGTCTTGAATTCTGATTATTGACAGCATGTCCACTCCACCCGATCCGTGAGTACATGTCATATATATATCCCTGTGTAATAGAAACTAACTGTGGAAAGGCTCACTCAACTGAACGACGACGTGCTCGTTAAAATCTTCGAGGAACTCCGGCCCAGAAAAGGTCTCCGCAACCTGTCATCAACCTGTCGCTGGCTTCGTGAAGCATCAGCTTCCACTCTTTTTTGCTCGTGTTGTCAATTCCTAGGTGAAATACCGTGGAAGGTAG

>TU446-GaLu96scf_1-3581589-3582195 GCACGCTCACTTTGGCCACACGACCGGATCACCCTGCGCGATGCTTGGCCTCTATCCACCTTCACAACAGTCTATATGCAGATCACTTCCTCCACATGGTCTCCAAACGCATCTTGTCTCAGAGATTGGCCCTCGCCCCGTAAACCTCGGCCTTCGGCGCAATATATGCGGGTCTCGATTGAGGCTCTAACGACGACACGCCAGCGTGCAGGCGAGTTCTAGACATTCCTAAGTGCAGGCCTTCTGACCACAACCTGCGCCACACACGTCGCCATTAACCTTGCTCCAACCCTGGCCGACGACCGGGTGACGCGCAAGATTACATCAAGTGTTGGCGCTGTCATTGACCACCTCGCTCTCCACCGTCGTTCAAACCAGGCTCAAACCCACCCGCCTTATGAGCACTTGCTTGCCTCCATGTGGGAGCTGTGGTGACTGGTGCCGAATGGCAATTCCTTAAAGTCTCATGCAGGTCTCATGTTCGTAGTAGCAAGAGCCTTTTTCCTGCAAAGAACACACCAGCTACGTGCACCCGCGCTTGCCAGTGACGCCACTTTTATTCATTCCTTTACACACGCTCTACTGCGGTCTGCCCACGTCCAGTTGT

>TU4508-GaLu96scf_33-352323-352990 TGGATCACCAGTGACCACCTCCTAGGCTGGGGTTCTGCCTTCCAGGGAAAGACATGCTTATGTAAACGGTCTGTTCTTTCTACGCTTACCGAAACAATTACCGTATCACGCCGTAAGTTCAACTCCCTATAAGCTAGTATTCTCGCCACCTCTTACAGTAACTTTTCACCTAGCATCCGAGGACTGTATCATCGCGCTTTGCGACAGCCGTGCGAGCAGCTGAAAAGTATGCCACAAGCGAACGTAGTCCTGTAGAACAACGCGACGTCTGGATTCCTGCCTTGGGCTGCGCGTTGGCGAAGGCTGTGTCACCGATTTTCGGCAACTCCTCCAGGCAACTTGGCAAACCGAAGAATATTTTGCTGTGGTCACTGGTTACAGTGGGTTATTTATCTGCCTCTTATCATCTCCCAGTGTCGATGCGATGACCGCGACGAAAACAGGTTGCGCAATGGCCGCTAGCGTACATAGTATGTACATACCCCCAGTACAATACGCCTCGCCCGTTACGATCGTCGACGTCCCGGCGAATGGCTAGAGCTCAGGATCGAGCGCAGTCATGATACATGCACTGCGGACAAGGCCAACTGTACGGATCAATGATTTTGGACGGGGGCTTGCGCCGCAGCACCGAGTCTGCCCCAGACTTCAAGTTACGGTAATTTGAC

>TU4509-GaLu96scf_33-353063-353539 ATACCAGCTCCATACGATCCACTGCTCACAGACTTGTGGATGCTGTACGCGAGCTTCCGCTTCCAGGATTGACATTTGCTCAGTGAAGAGGCAATGGCGCGTTTTGGTGTTCGGTTTCGCGTGCCACCTTACCCCCCGGAATTTACGCGTTTGCGCGCGGTCTAGGTGCGTTCACGCCATATGGCACGCCGTGTTCGTCATGCTGAGCGGCCGAGAATAGGTCGCTGACCATGCGCGGGTGGCTTCGAGACCCTGCATGCTGCACAGACCGTATCGCGACAGACTCAATGCCGCACGACGACCCGCGGCCCGCAGTCGACACATTCATATGGGCATGGGACCGTGCGCGAGCACGTATGGAAGGAGGGTGAGAGGGTCGTTTTCTTTATTCCGTTCAGACATCATGATGTTGGTTAGACTGCCCGTGAAGAACGTATCTAGATGTCTAACCTATGCTTACGATTGCGTTACACCGTC

>TU4558-GaLu96scf_34-242410-243156 TCGCGAAGGAAAGAGAATGATATACACTGAAGAAGGGGCATCCCCAAAGGCTGCAACGCACCGCAAGTTCGATTGCGCTTGAGCTTAGCGTCACAGTATCGCCCCGCCTGTGGACAACATTCTTGGCTAGCTGGCAGGCGCGTCCTGGACGAGAGCAGGAACTTCGCTAGACCGTAGACACGTAGCGACAGGATAATTGATAAAGACGTACCTGGAGAACACCGTGCATTTTCCAGTTGGTCCATCCGATCCTCCAGTGCCCGCTTTCCGATAGGACTTGCAGCGCTCTCGGCTTGTCCCTAAAAAGCTTATTATATGTCGGCATATGGCGGACTAGAACACGCCCTGAGAAAGGCGAACAGAGCAGTCATTACAAGGGTATAGAAGGTGATGGGTTCCGGATGAGGCTTGAGGTATCGTGCGTGGTGAAGGCCAAGGGTTGTTGACGGCTCAGCCTCTAGACGAACGGAGAGACCGACCATATCCATAGTTCCGACGTCCCCTAGAGACCTTCTCGCGAAAATTCCGTCGCTCTTCTCCGAAGCATCGCGGACAGAGGTGCGTCTTTCTTTTTGTACCCTTCCGCGTGCCAGCCTGACCTGGCGGTTGTTTCTTCGCGGGCTTCAACCCCATGTTCACATAGAGTTCGAATAGCGGCCGAATAGGCGTGCGGCGAGTTTTCGAGCTGGACGAGAGACTAGTCTAGGATATTGCGCACAAGGATACAAGCAAGCTGTATGCGGGCGG

>TU4559-GaLu96scf_34-243275-243832 TATGGAGTTCCCACGCACGTTGTCTAATGTGACTTCTTGGGTACCACCCTGTAAGGACTTGTACTTTACCTTCTGCATCGTCCGCGACTTCTCATTTGTCGAGAGGTACTTATATCCCTCTGCCAGTACTTGCCTTCATCGCCCCTGCGTTCAGAAATCCAAGGCACCAGTCTCGAAAGCTTCACACAGGCGACAGTGAGGAATAGCAGTTCCCAAACCCTCGTGGCAGCCTGCAGGAGCTTGCGGCTTTTCAGGGCTTTTCCCAGTGCATACGAAGCTGACTCAGTTACCCATTATAGTGCCCCCGCCCCAACGTTCAAACTCCGGAACCATATGTGCTGCGGTAGCCTGACGACAACGATGGTGGGGACTCTAGACCAAGATATCTCCAAGTTGTCTTGACCTATCATTCCGCCTCACTTTGGCTGCTTTCTTATTATTATTATCCATATGACGTCGCTCTCCGAGTCCAATCTGTTCGCGCGATACATTGACCAGGATCTAGACGAGCTTGAGCAACTTGCACAGTCATTTCTGAACCAATGCAAAGCATCTTCG

>TU4579-GaLu96scf_35-133012-133907 AAAGCTCTGAACACAGAGACCAACGCGTCAGCGATCCGATGGATCGGGATGGTACGGCAGTGGTACAGATCAGGGGGAGATTTGGCTATGCGAAGGAATCACCATGAGTATGATCCTTCAGCATGCGAAGTTGAATAATTACGACACCGGTGTCGGCGACACGGCTACGGACTCCTTCTCCAACATCGAAGTTGTTGCACCAGAGTTTGAAAGCGATGCGTTTCTGTGGCATCCGGCAAGTTCCTATTGGCCACAGAGACGGGCGCTAGGACCCGAGCCTGCCCGACGGCAACATCTACGACTCCGAGAGAAAAGATGCCCGCTGAACTTCAACCGCTGAATGTGCACTGCGATCGTCCCAGCTTCTGTCAGCCTGTTACCATCGATGCTGATGCAAAACCACAGACACTGGCGTGCCCTTTCAGGGCAAGACGGAATGATGCTCCTCGTCGCCGTCGAAGCGACTCCTTGCTCCATCATCGACTCCCATCGCCAGCACGACACTTCGCCGCTCGCGTCAGGGTTAAATTTGGTCCAGCCAGAGGTCTGTGATCCCTGTCGTAACAGCTGTTAAGGGTAAGCACGCCTTAAAACTGCTTCTAGGGTTACCGGTAATCAGAGGTGGCACAGGGGTTCCCCGTAGTAGTCTTGCTCACGTCTGTCCCACAGGACATGCGCGAGCAAAACCGACTGTCCGTAGGACCATGGGGCGGATAACCTTGGTCGTAAACCGAGTTATGACATGATAATTGACGCACCCGTTTTGAGACATGGACGGCGGTCGACCCCTCATGAATGACGGAGTCCCCGGACTGCCCTCGGTTTCCGGTACTACGGTACACTGACATACGCCACTCAGCTTCCCGGCTCGGAGGGCTGTCGGTCAATCCTATCGT

>TU4580-GaLu96scf_35-134007-134558 TAGAGAAGAAGGAAGATGCGATTTGCTTCGATCATCCGCTCCTATGACCCAGCAGGCTTATGGATTTCCACGATTTCAGCGCCGCCAGCATATGGATGGCTTGGTCTGCATTATCACGCACTGTTGGTACGTTAATTGAACAGGATTGCGGTGGTCGATGCAATAGAAACTCACCAAGTTGAAGTGTAAACTATAGCGTACAGGCGTAAGGCCATCGCTTGGCTTGAACGTACCATACCATGACCCCCTAATCACCTTTGCAAATTGGGTACCAACAAGGAAGGTGACGTGCAGCGGAAAGCAAGGACGGTCGACGGCCAGAACCGAGGCTGCTTTGTTCATAATCTGTCACACGGCCGGCCCAGGCGCCCACCCAAAGCTGCTCAACCAGAGCGCCTGCACGCTACAACCGGTTTGTATTCTCTTTTGACCATCGACCTTCTCCTGTACAAAATTCCCTGGCCACTCGACGGCGCGATACTCGGCCCCAGGAGGTCCCTATTATTTCTGCGGCTCCCTCGCACCGACCGTGTCACCTGCCAGTGTGACTAT

>TU4610-GaLu96scf_35-270253-270588 GACTCGCACTATTGTCCCCCTGCGACGTACTTCTTCTTTTCTCGCAGGAGCCTACCGCAAGTCCCAGATGGACACATCGGCCCTAGCTGGAGCGCACGCCATCCCCGCGAACGTCGGAAACGGCGAGGGCGTGCTTTTCCTTACCAGGGTCGGGACATATGTCCCCCCATCCTCGTTACTCGTCTTGTCTGTATCTGCTCTGTTTTGTCTAGCGTCTGGTAGTGGGTTGCAATACATCCCCATTTACGCCTCCCCTGCTACCCACTCCGCAAATGTGACTTCAAAGTCCACGAGCGTTCCTCACCGGCACATCTCGCCTCAGGTTGTCACCGTCGT

>TU4615-GaLu96scf_35-283986-285038 GCAAGTCGTGGAACGTCGAGGAGGGCGAAACCCTATCCGTGCGCCTGCCCGTAACATGGAATGCCGCCGCATCGTGACTTCTCGTGTGTTCGCGCATCGGCGCAACGCACTCGGTCGTCTTCAAGGGATCCTTCAAGCCGCTACCCCACCACGCGCAGAACAGCAAGTCATCACGTCCGGAAAAACGGAGGGACAATCGCAATGAAGACATTTACTAACCCAGTGTCCGTTCATCAAGTACGTCTGCGTCCTTAAAGAGGTCTCCCGGACGCCCGGCCGCGACAAGCAGTGGCACGAGGATAGTGCAGAGGTCCCACATTTCTGCTTCCTGAAGGTCTTGCTTGTTGAACGTTGAGGACCCGTCCTTCATCCTGAACGTGAGTCCCTCCTTTCATCTATCTCCACTTTGCGGCATCGAACAGGCCAATGATTGCAAACATCTCGTTCGACTTTGAAACCCGTCGGTTCTGTCCACACGACTGGCGACTCTGCCCTCATCACGCACCCCGGCGCGGCCGAGACCAAAGGCATGTGCGTCTTTTTTAACCCATTTATTTGTTAGTTCTGCTCACCTTTGTCTTTTGCACCGTAGTTGTTAGCATGGCGGACGAGCCCGCCGGCCAGACGGTGCTTCATTCACGACACTCGGACACCACCATCCGCCTCCCGTGGATGCGTTCAGACAAGGTGCGTCCTCCATTTCCGTTCCCCATTTGTATTTTTAATCGTGCGTGTATGCGGCAGATCATGCGCGGTGCTGCGGAACACGGTCACTACTGAGGGTGACCTGCTGGGTGACCTGAGCACGCTCACTGCTGCAGATAGCATTCAAGAGGAGGTACACATCTCTGATGGTGTCGTAATTGAGCGCGTGTGGACAAGCCCTTGGCACCATGTCCGTAAACCCCTTCGGCACGGGAATCAACTGGAACGCTAGTAAGACTCGGCCTACGTCTACTCCCACCTCGCCGCTGGCCACAGGCAAGTCCGGCACCTCAGCATCAACACTGCTGTACTCTCCTAACCGGTCGCGAACGCGGCGTCGCTATCATT

>TU4628-GaLu96scf_35-338396-338965 ACCTATAGATTCCCTACCTCTGCGAGTGTTCCAGTTCTCGTAACTGAGCTCTGCGCGTCCCCTCCCCTCTCACACTGGCAACGCGGTTCTACTCGTCCAAAGACGTCCACAGCCTGGTGCGCGACACCGCCCTCGTCAAATACCCCGTGGCGAGTCGATCCATTGCTGACCGTGACATGAACTCACCACACTCCGGGTTTCGCTGTTCGCGTGAAAGTTGTTTGAAAGTGTATAGGAGTAGGAGACTTACATACCTTTCCAGTCTTCCGCGGCCACCGCGCCTGCATGTCCTGCCAGTGCACGCCTCTCGCCTCAGCTGTCCCAGAGCGTCCTGGAAGACATCGCGTTGCGCGATGCGATTATCGCGGAGGGCTGAGCGAGTTAGGGCTTCGGAATCGATTGTGTAGTGGCGGCGGCGGCGGCGTGTAGCAAGTAACCTCTCTCAGCTTCGTACGCCCTACATACTGGCCCACTGGCCAAGGCGCGGACACGGCCTACATCAGGGAAAACCTCCTTCTATAATGAGGTATCAATTATAATAACCGGGCACTCACCGAAACTAACCGAAAC

>TU4639-GaLu96scf_36-26726-27579 TTACTACACTACACAAGTACACAACCTCTTCCAGCCGCCGCAACACCTCTATGCTCCCAAACATGGCCCGCACCGAAAACGCGAAATCAAACCATTAGGGACCACACTACAGCGAAAGACTGAGCAGTCTAGCGACATTATAAGCAAGGAAAGTCACTCACCAATGGCACCGTAACGATGGGAAGCGAGCGGACAACGGAGCAACACCTCCTGGCAAGGGCGTCCCGGGGGAAGCAGAGTTTGCGCCTAGACTCGAGTCAACTGCGCGGCGGTCTAAAACAACGTGAGGCAATTAGTTCACCTTTGGTGATATGCAGGAGCGTGGAGAGAGAACATACCAGAAGCTTAATGAGGTAGCAACACTCCTACTGGTAGACCACGCATTGATGAAGGTATAACGCTAGGCGACGATGTAGCGAGATCGGGACCTTGACGTAGTAGAACAGGCAGCAATATCTTCATCCGAGAAAGAAATAAGGGCTCGAGGGGCAGAGTAAGGAGGACTCCGGTAGAAAGGATCTGGAAGGCTTATCAGCAGTGTTCTCTCCCTATGCCTGAGACGGTCGACTCAGAGGAATCCGACCGCACTCAGTATATGAACACTTACCTACGATGCTGCCTGCAGTTCAATCGAGGTGCGCACCAGGGTGATGGAGTGCTCACCTAAAGAGCTAGACTCGCCACGTCACAACCAACCGCCCGAGCTGCGACCACGGCAACAAAGCTAGAATAAAACCTACATACCGCGACACTTTGTCCAGAAGGCTGAGCGAGGAGACGGACTACCGAGCGGAGAGAGGGAGCGAGGACTAAACTGCACGGCAAGAGGTAGGGTGGACATGGTGGACGATGGA

>TU4643-GaLu96scf_36-60737-61013 CAGGTGTGCGCGGCGCGACCGGCACTTCCGGCCAATTAAGGTCTCCTGGCAACTAAGCTCTCCCTGCGCGCAATCCCCACAACCTTCGGTGGTTGGCCACATGTCGGACGGGTTGGGCGGCCCAATTACCTCCGTAACCTCGAGTTCAAAGCCCACCCAGTGGCCGGAAACGCAAGCGTCCAGGGTGGCAAGTGGCTGTCCCCCGCGCTCCGTCGCGTAGCCAGTCGAGGGCAAGTCGAGGACGGGACGCGTCGCGTGATGCGAGCAGGAGACCGCA

>TU4652-GaLu96scf_36-239340-239928 CCATCTTGCCCCTCGGCCCCTGTTATCATCTCGTGCATCTATCTGCCCACTGTGCTTTCATACCTTTTTCGACCTCGGGGCTCCCTCTACCATCACTCTCCAGTTTTCATTTACTGTCTCGGTCTAAGGTGCGTGCGCTCGTCATTGCTACGTCACTCTCTGCTTCTGGCCCAGGTGCGTACGCGATGTGGTTGGATCTAAGTTGACTGGTTTGCGGGATGGTCTGAGTCAGAGGTACATGGCGAGTGTTGGATATGCGCATTTAGGTACGTGCTACTGTATGTCGCTGGTCACCGGTCGCCGGTCGGCATTCAGGTACGTCCCCAGTGGTTGGTCATCGATCCGAGCTAAGATGTCGAGGCTACCACAGCCCATTCACGGGCCCTTCCTGTGAGACCGTACGTGGGACTGCCGCGCCCCCGTTGTTGGGCATCATACATACACACTCCGAGCTGCAACGCCACCCTGCTACGTCTCCCGTAACGCGCTTTCTCGGGTATTGGGTGTGGTACCCCTGTATTGCCAGTTGCGCTGTATGTACTCTATTGTCGGATTGGTATATAGCTCAATATATTTTCATTATGAATTT

>TU4675-GaLu96scf_37-146547-146798 GCGGCTACGCTTTTCCCCATGTTGCTCATGACTGCAATACTCTACGCGGTCGTTCGAGTGTCAAGCTGCGTCCAGATCAAACCCGGACATCTCAGCGCCCCTCTATGTCTTCTCCTCACAACACCCGACGTGGAGAAGATGGGCCGGTGGTTCAGGACATCTTCCAAATTTTCTGTCTTAGCCTTTAGGTACATCCCCAATATATTTTGTTGTTATCACGTCGAATGGGAGGGATGTGTAGATGCCGGTCTC

>TU4677-GaLu96scf_37-154191-155944 AGTCCCATCCCCCGTGGTCTTGGGTCTCCGTTCATCCAGCATAATGCTTCGTGTTCTATATATGTTACCTGATGTCTCAACTTCGAAAACGACGATGCCGTCACTGATCGACATGGGCAGAGACATATATCATTTCGACGACGCATGAACGGGCCCAGGCATGATTTAGAAAAAGCTCCTGACCTTGAACACGAGCCGTTCAGATGCGTTGTTAGAGAAAGGTGCGTCTGGGGCCGAGATTGCATAGGCTTCTTGACCATACATACATTGACTACGTCTGTTAGAGAACACCCGCTACGCGGCGCACGCGGCCCCCATCCCAGAGCGAAAAACGTCCGTGTACGCCATAACCTCCATGCGCCCCTAGCCCGTTATCTTACGCCCAAGTGACAATACTGACGTCCAATACATTGTGGTTTGTTAGCACTGCTTCGTCCAGCCTTCCCGTGCCTACTAGTCTGCCGTTCAATCGCAAGGGCTTACTCATAATAGCCAGGGTCTAGTTGCCATAAGGTGAGTAAGTTCATTGGCGTCTGTCATTGCTCGAAACAAACCTCGAACCGATCTTCTAGAAACGTTAACGTTTGTGGATCCTGGTTTTTCTGCGTCGAATGGCTTGTGTGACGGTCACGCATGGCTGTACTGAAGTCATCCACGTACAAAAGCTCGAGGCCTGGTGCAGTGCAAATCCCATAATTTCTCTTCAGTGCTCCAGGTTAACGTCTGCGTCATGACTCACTCAGACTCTGTCGCCTCCTTGGTCGTTCCTCCGCCATCCCGGCCAACCATTCCCACTGTTGCCGGGGGCGTTCTCTTTGGAGGCGAGTGTACTCGTTCATTCTTTCTTACCGACTACGTCTAAGTTGCGACGGGCAGGCATATTCATACTACTCATCGCGGGCTGCCTCTATTATGCCAATGTGTGCCGCGGCGATGCAAGTGCCTCTTACCTAAACCAGGGCCATAGAGGCGATTTCTTCCACAGGTTCCGCGACTTGCGCTCCACCTTCACACTTGAGCCTACCGCCTCCATCCTCCCGTTGAGTGTGCGCACGGACAACATTCTGAGGTCGTTCAACTTCAGCTCCCCGCCTGCGGACCTACATTCACGCAACGCGCGTGCGCAGCTGACCGTTCCTCCTCCATACGGTCATCCGTACGTGTATCCCCCTTCCTACGAAGCGTCGGTCAGCGACCACGGGGGTACACCACACTCTCCCATCTCGCCTTCTACTCCGGGATCTGCTGCGGGGGAGGGACCGACAGCTAGCTGTAATGTCTGAGATCCGACGTCCTAGGAGTGCCGTTCCGCGACATTGTAGCCTCCGCTCCCAACGATGGCGACGGAGAGCTTGAATGGAGGGGCGGAGGGTTTACGACAGTTAGTAGGAGCGGGGGCGTGGGCGCGGTCTACGGACGACGAGGGCTCAGTCCTGTTACTCTCTCTTCTTTCCCCTTGCCTTATACCCCACGCCCCATCTACGGCTATACCCTACATATAGCGACACAGCATGTGTCCAACAGCACAGTCGTTTTTCTGACCAAGTATGTCTGCGTGGCATGCGATGCAGCTTCTTTCTTCCATTTTTCCTTTCCAAGCGCCATTAACCCATCCTCGTCTCCACCGCATACTCGTGCCTCGAGGCCTGCTACGTCGAGGAGACATCGCCTTTTCGGTCGGACATTCCATCAAGAATTTCGGCGTACTGGCTCAGAACGCAGCTCGCGCACGAGCGGCTGCCAGTTGGTCAGAT

>TU4683-GaLu96scf_37-181093-182175 TGTAAGTCGCAGTAGAAGATGAGACTTGGGTGTTGAGTGAGGATAGGTCGCTCTTCAGTACGATACTTTATACACCATGGCCGCACGACTGTATCTTGAGAACACATATATGGATCCGAGTGACGTACTGGTATGACAGTCCTTGGTCCTGCGCTCCCCGGTCACCAAGACGGAGTCCGCGGTCTCTACATACAATTCAGCCCACGGTTTGTCGGACTGCAACACGTCAGCCGCAACGTCCGACACTGGCAGACCTTGGAAGCCGGAGCGTACTCACCGTGCAATCACAGTGCGATTATTTTCCCGGAAGGAATGTAACTGGTACCACCCATGCGGCGGCGTGCCGTTATTGAGGATAAGTGGGATACGGGAAAAACCGTCCATGTGGCAAACGTACGGAGGCGAGGAACCGGAGTTGGGGCGCTCTGCCGAAGAGCCCAGCGCGATGTAGGGTACATGATCCGACGTTATCGAAAAATACCGGCGCGATGCTGCTGCTTGAAGGGTCATTTCGGAAGAGGGTACATGCGCAGATCACCGCATTCATATGGTGTGACACGGGCCCGACCCTACGTCGGCGAGGTGCATATCGTTCGCGCAGCGTCGGCACCGACGTGATGACTGGTCGAGGTTAATCATCGGATTAGGAAAAGCCGGGAGCTAGCCTATGTCGATGAAGTGAAAAACGTCACTTCGGCCTTCCTGACGGCACCAAGCATGTCGGGTTCCTAGAGTTGACATGGTCAGGTAGATGTCGACCGGGCACCGGTAGGCAGCACAGAGACATACCGAATCACCCATCACCCGCAGGCCCGACCCTTGGGATGCCCAGTCGCTCGAGACTAGGTCCTTCACGCGCTGCCACACTTCTGCTGGCGAGAAGGCGTATGATTACGGTAATGACCTTCAACCCGTCACGTGAATTCAAGGATATATAACTACAAACGCAGCTCGCAAGAGTGGTTCCGTTCGGGTAGAATAACCTATTGCGAGCGGCTTTTCCATGGAGGAAGACAGCTCGTAATCATGTGGAAGGTATGACGGATCCCTTTAACGGAGTATGCTCACCTATGCAGCATGCAG

>TU4684-GaLu96scf_37-182329-182656 CACTGCTGACCATATACTATGCACAATGGATGACAGTCGATCCATGTTTAAGGGGAGGGCGATCGTGTGGCAAACAAGTCGAACACCCTTGGTGATGAGGTACCTCCTCGCCGGAGTTCAAGATACCTTCGTAGTAGTGGTCGGAGTGCTGCTGGCTTCCAGTTGGCTTCCCTACCGCTTTCAAGCTGATGTGTAGGTGTTAGGTGTATCCTGTTGATGTACATAGTATCGGTTTTGACTGAAATCAACCCTCTATTATTACTCTCCCTTCATATCTCTGCGGAGAGAAATGCCTACCGTGAAGGGGTGTATATATATGCGCTACGCT

>TU468-GaLu96scf_1-3795797-3796385 ACCTCCTCTCATCTCCGACGCCTACAAACCAGCCATGTTCTCCAAGCTCTACATTGCCTTCACGGCCCTACTTCTTGCGACGGTTATCACTGCCAGTCCCGCCAAACCGTCGCATGTCCCGCCCTCGGGTGTGGTGGGCATTGGTCCGGGCGTCGATACGGTTCTCAAAGGCGTCGCCGAAGTAGTCACCGGCCTGTAGACCTGCGCTAGCTGTAAGTGAACTGTGACAGTTTCGCAGCCTTTGCCAGACACTGACCATATCCTAAGGGCAAGGCCGCCGTGATTCTTCAAGGACTCGCCTGAACTGTAGCAAGTCCTGACTACGGTAGGCTGGCGTAGGGCCCGCCGACACTCTTGGACTGCCTTTACTTACCCTAGTGTCCACAGGAATCACCACAACGTGCCGAGCGATGGCCCTCGGACCGTGGATGGGTTTTCCTTCCCACATTGCCACCGTTATGCAGCATCTTCTTAATCGAGACTCGGAGCGATTCTGTGACGTTGAGCGAGATTGTAGGATCCGCATATACCCAGTATGAGCTATGTGTGTGCAATTCCATGCCATAATCGCACCCGCGATCCCTAGAAT

>TU4691-GaLu96scf_37-256939-257337 GGAGGAGGCACCGCGTAAGAATGCGTTCGGACCGCTGGGGCTAGTGGCGACGAGGGACTGCAAGCGTAGTCCGAGTCCGGAGCGGGGGAGTACTAGTGTGGGGGATCGCCTCGTTCCCGCGTAAGTGCAGGGTGTGCGAAGCGTGAGTGCCGTATTCCGGGGCCACAAAAGAGCAGGTTCCACATCCAGAACGGTCGTGCGGTCTCAGTCGGGATCGCACGGAGGGGACTCCTGTGAAGGGTGCAACCAGGGTTAGTACGGGTACTACTGGCGAGTAGCCGGCGTAATCGTAATTATAACAGGGCGCCAGCTGGGATCTGCCCACCAAACGCACGGTCAGCGTGAGCGAGACGCCGGTACACTGTGAAGAGAGAGGAGCGCACGGCGTACTCGAGGTCC

>TU470-GaLu96scf_1-3797776-3799321 TTCTGACTCTTGACCATATGTGAACCCCCCCCGTCCCCCATTGTGTCTATCTCACCAACCGTAACTCGCTAATATCAACTCCTGTAAGCTACTGCGAAGGCTCAACGGTGACGAAAGGCGCACCCACTAGGTTTCCATGTCTTCAGAGCGAGTCTGGACATCCTTCATAACTAACGGCCTAATCAATAGGATATCAAGCTTATCACGGCTCACCATGTGTGTTAAGTGCGCACGTAATCCAATGCACAGTACCGAAAAGCACTACAGGATGTTCCCGCCGTTATCATTGACCTCCACCTCCGAGCTGTCTGGATCGATGGCGGCGCCATGCATATCTCCGGTATGCAGCGACATTTGAAAACCGCTCACTGCAGGACCCACAAGCACAGTGGAACGAGATATACATAAGAAGTGAGTGGCTATCAGAACCCCAACGCCGTGAATCAACTGACCGTTGATGGCTGTCTGATACCCCCGCTGCTATCGGATACCTTTGGCCCCACTTTTTGGCACACTATCGTTGTAAGCAGGTCAGAAATTGCAACCACGCCGTGACGCGATAACACGGTAGTGATACTCGTAACAATTACAGCGTCGAGAATGCGCGAAGAGGGTGGCACGCCGTCAAGATCCGAACATCGAGGCGATCGCGGCGCATCGCGCCCATCTTCTCTCGTCTAGACTCCTTGTGAAGGTGCTGGTCAAATTTTATTTTCAAAAGGCATCCCGAGCGCCTCCGAAAGCCGTGAATGCAATCACACTGTGGGGACTCAGGGGGACTCACCACAAGCTTTAACTTTCGCCATTATGCCACTCCTCCCGATATCCAACCTTATCACACACGCAATGCCCCTTCAGACAGTCTTCAGCTGCGATTCTGTAGATTTCAGCTCTAGCGAAGGAGCGGTCCCGCCAATCCTGCCACCGCCGTGGGGAGTGACGCAGAGCGACCATTGGTCCGATCGATCCGTTGCGAAGGTCTCCGGATCCGTACGCGTGTAAGATCTCGACGGTTCCAACGGCCGTACGTATGCTTCATTCATGCCTCAGAGACTCCCTTGCGCTCGCGGCTAACGTAAAATTCGGCTTGTGGCAGGTCTGCTTATGATCGAGGTGGATGCTGCCCAACCGATTGTCTCCAGGGCCATAATGGACCGCTGGGAGATTTCCAGTAACGAAGTGCGCTTACACAGAATGCGCAGCTACCCCACGCTGGTCTAAGCCTTTCACGCAGACCACTTTGACTGTGACGTTGAAAGGGGCAGAACCAGAGGCACGCACAATACCGACCCAGAAATTCCGTTTTGGTCAGGCTAGGGACTTCTGGGCTTTTGTGTCCCAGGTCGCTTCGTGGCGGCTGCTTCCGCATGCCACCCCGCGAGTGGAGGCCTAACTTCTAAGGGTCATTCTCAATATTCTGGCCAAGTAGTACTTGAGGCTTGTTGTCGAGCGCTGTACAGAGTATATATAAATGGACAGGTCACTGCCGCGTCCGAAGACTGTTGAGTTTCAGACCCGGAGGACCGACCGTTGTTATCATAGACAT

>TU4729-GaLu96scf_39-22356-23082 GTAAGGTAACGCTCGTACCGCTCTCTTTCTACCCTCGGCGACAGCCTACCCTCCATGCACACTGCTCTCATCTGGGACTCCTCATGACTTTCATAACTGTCTTTCTGTTCGACACATCCTCCACCGTGGTCTCGTTAGGTCACTGCTCGGGGTGACAGCACTTATGCTTATGGGGTTCTGTCATGATTAGCGCCGTCCATAGAACACGTTGGCTCATTGGCTGCAGGCAGAGGTGCGCCTACTTAGTACGCACTCTTTGTGCTTACAACCTGGCCCGATTAACCGCTTATATGCCGTCGTTAGACGTGAGAGAATGACCGAAATGTTATGACGAGGTGCAGCACCCATTGGACGGTCAGGAGGCACTCGCGAAATAACAGGCAGAAACGAAGACTACGCCGGCGAACCGCGTGCGTACCTCGTGTTACGATCGCCTTTTCTCTTCGTATATACGTGTTTCACACGAGTGTGTGCTCTGTTCTCGCTCATATTGGTCATGTGTAGGAGGAACAGAACGAGATCGCTCTCACGGAGGCCGCTCAGGCCGCATTGAAGGGTCGTTCGCAATCGTCATGATCGTGGCAGAGCTACCGAGTGGTACGCACGTTTTTAGTGCTGTTTCTGGTGCGTCCAATTCCTCTACCTCATTTATGGCTCAACATTCGATGACTGGGCCCATACAGCTCTCGTCTTCGACATATTTGCATCTTGCTTTCGTTTTTCTCCC

>TU4736-GaLu96scf_39-35930-36324 GTGAAGACCGCGTCGCCCATGTCTATATTGGTAGTCGGATGTGGCGATGACATCATACCGTCGATGGAAGGACTTACTAGGTACTGCCCACTAAAGTCCCGTTGGGGTGCTCCGATTCGTTGGGGAGGGGTGTGCCTCCCCACGGTGAACAAGTGCTCAGATGGGGACGTCGGCGGGAGGAGGGCGTGGAGTGGCATCCGAGTCGGTGTGTTCACCCGTAAGTAAAATGATGACAGCAAATTTGATGGCGTTGATGGTGCATAAGGCAGAGGGCGGGTATGGAAAGCTGCTATGGAGGAAGAGACTCGGAAGGGTGCAACGTTGTAAGTAGCTGAGAGAAGGAGTGAGGTACTTAGGTTTGGCAGGCGCGCAAGCGCGCTGGTCCCATGTAATTG

>TU4779-GaLu96scf_39-151335-152022 GTCTCTCCGTCCACCGCTTACAGCCAGATCTCGCTGCTGCTCAACATCTTGACCAACTAGTCCTCGAAGTCAGCCGTATCGACTTTGCACCTCCCAGGGGGTACGTGAAGAGGTGAGCGCCGCCAGCTAGGCAGCGTCTTTCGACATCACTTGCATGTACGTGCTTCCCTCCCCCATTCTCTCCCCCTCTCCAAGGCATCCACACGCAAGGAGCTTACAGCCAGCCCCCCCGGAGATTAAGTCCCGCAAAAAGGCATGGAGGGCTATGGGTTCATGAGGATAGCATGTCTTCTACCACATCCTAGGAGTCGTTCGTGCGCCGGACAACCCTAATCTGTGCTTGGGGCTCTGTATCCTTTTGTATTCCTTCGCAAATCAATGATGTGAGTTTCGTCACATGCGTTGGGACCACTACCCCCGGACTCTTGAGTGACACTTATCGTTGTCATACAGCACGTCTTGAACCCACTTTCCGTTCAGTGCGGCCATGTCCTTGACTATTGTCCTAAGTCCTGATCAAGTGGGGGCTACACATGGCTGACGCGCTGACGTCTAGATCCTCGAAGTACATATCTTTCAGACGGTGTTCTTGATGTCGAGCATTGCCTGCAATCATAGCAGCTTGAGATGGATTCTGTCTCGGGCTGACAGGTACAGGCACTTACGGCTTACCCCTCAACATAGTTTT

>TU4809-GaLu96scf_4-170701-171255 ATGACGTCGAGGTCTGCAGGTGGGAATTATAACGCGTTGGTTGTTGGGTGCCGTGTTACCGAACACATCAGAGTCACAGGTGGAGGAGCTCTATGCTCGAAGCCTGTGGACACAGGTCATTCCGTGCGGCGGCCCGAGGCTTCCAGTGCTGTCGCAGCAAGTCGAAATGTAACGCCATTGCGAGCGGGCCGCCCATAAAGTAACACGGTGGCTGCGGTCATGGAGGGCAGTTAGTTCATCGCTTGGATAGACTGCATCCCCGTTCGTGAAAGCGAAATCAACCACCGGGTTGTTCAGAACGAGACAGGAGTGTGGCGGCGGAAACGGATGCAGTAAGTGGGTAGATGTTGAGAAAGTCAGTTGAGAGAGGGGAGGGTATAGGGCGGAGACAGTGAGTCGGGGGACGCCACCTGCGCAGCAGCGGAGGAGCAACTCTACATGTTTAGAACACTGCCTCTGGCGAGCCAAGGACCGGGCAATGTCATGAGGCTCTTGCGGTGGCGAAGGTTGGGAACGGGGGATTTCGCGGCGAGCGACGGGGTACGCTGGACGTCA

>TU4834-GaLu96scf_4-521130-521585 TGTTACCATTCACATACCAACCGGGGGGTACTATGCAATCTTCCTCCCGCCGCGTCGGACAAGAACGGCCCGCATGCCGTGAACTGCTACCACTACGCTTCGACGCAAACTGAAATGAACTTCCCAATCTCCATGCACTAATGTGCCTATCCACTGCCATCTATGTCCGCCATCCATCACGGGCCAACCGCGCACGATCTGGAAGTACAACACCTTGCAACACATTGTCTTCGAGCACATGGTCCGCGATAACGAAGGCAATCACTTACCATCTATCCCACCTGAAATGGTGGTCAATATGTACATCTCCTGGGCCGAGGATAAGGCGCTGGAAGTTGAAGAGGAGTTGACAGAGGGCTAGCGAGAGAAGTATGACGTTCCGGGCAGCGATGACTTTGCTGAGATGAGAGAGAGCCTTGATGCAGGGGTAAAGGGCTGAGAGCGGAAGAGGCGGGA

>TU483-GaLu96scf_1-3880882-3881311 GAGTCAGGCTAACGACTCGGAACACTACTTACTTACTCAACATCCTCACCGTATACCTGTTTGGGCCTTAGCCGCAATCATATTCTTCTCGGCGACGCATGGGCAGTAGCCGAAGACGCTGTTAAGCTGCTGAGCAACTGGCAATCATCTCGCTGCTCTGAGTCGACTCTGGAAGTTAACCGCAACCCCCGGCTTCTTCCCTCTCTTTTCTCGGCCACCGCAACCGGTCGCCGCACCCACTCATATGGAAGGCGTTGTACTCGTCGCATCTTTGGACGCTACCGCTTCCGAGTCGGTTGGTAAGTCTCTCCCTCGGTCGTCGTCCCATCACCTGGGACTGCTTCCGGCTTGCAAGCTCGTTACGGCACGGGCTGTGGCAGTCAGTAAGCAGTGGAGAACAGGGCCACCGACGGTACAGTCACATCACCAT

>TU4898-GaLu96scf_4-1138613-1138913 GAAGGGTGACCCCTGGCGGACGACGTTGTACTGTGCGCGGATACCTCGTCTTCCTCTCTCTATGCCAACGCTCGTAGGCAAGGTCAGTATCAGCACCAGGGGGTCTATGGAGAGAGCATCAGAGAAGTTTCGGGTGCGCGACAATCATGGGTGCTGTGGCGCGACTCACGGTCATAATGCCATGAGAATGCAGGGGGCTGCATGTGGAAAAGCTTTAGTTCTCGACGGCAACAAGCGAGGGTGATACGGCAACAAGCGAGGGTGAGAGCTTGAGACCGGAATGCCAGAACACGGAAAATCG

>TU4919-GaLu96scf_4-1344009-1344268 GTGAGTGGTTCACATAAAGACTGTTTATCGTCATTATGGTACCCTGTTCTGTGTGGTACTTGTGTACATGTGTTGGCGGTCGGTCCTGTGCCACGTACGGCGGTCCGCAGTTTTCTCCGACACTTTAATGCGGTCAACGCCATAACAAAACATGTCTTCCATATGACGACATACCTGGATGGCTTTTCTTCAGGGTCGCGAGCCTCAACTGCACAAACCACCACAAAGGATCGTATAAGTCACAAGTCAAGGATCCTGTA

>TU4923-GaLu96scf_4-1364901-1365250 CGCCACAGCGCTACCAGAGTTTTACGCCATGGCATACTTATAGCCGGGTAAGCCGTAGTCATGTGAGTAGAGGACTGACAGGTGCGCAGGAAAGCTGCAAAATCAAAACCGAGCAGTTTCTTTCACTCTTCCCAATCCGGCGTACCTCGGGGCGCCAGATTGCTTCCGACACAATCCGAGCCGTCGACCATGGCCAACTCCATTGAACTCTCTTCCTTCGTCCACACCTCGCAAACATACGCATTAGCCTTCACCTCACGTTGCCTTGAAAGAGCCCTCTGCGGCACGGAAGTCCAGGGCCGCAAGGGGCAGCAGGGGCAAACCCGCGCTCTTCCCAGTCTCTTCCCTCG

>TU4926-GaLu96scf_4-1379376-1379938 TTCGGGTCCGAAAATAGCTACAGATAGGTGTACGCTTTAGAACGCTACCCGCGGGCTCGTCAACAGCTCGCGCGCGGGACCGTACGGGCTCGCGCTCGACACACACCCCGCGCGCGCACTCGCGCGCGCCTTCCCGCTCGTCCTGCCATTCAATGCACGCCCTCCCTCCCACGGCGCGCTCCTCTGATCGAGCTCCGCGCACTGTCGCGTGCGGTCCTAGCGATGACGACCATTAGCATTAGCTGACGTCATAGGGCTTGCGTCCGCATCTTCACATACCCGTATTGGCGACGGCGCGCGAGGCGCGCACCGGAGGAAAAAAAAGGGAGGAGGCACTGGCAGAGGGTTGCACAGTGGGGAGATGGTGGGGGGAGGAAAGAAGGCGGATGACGAAGGGCGACCTTGTTGGCCCACCTTATATATGTCGCCGAAGTCGGGGACTCCGATGGTGTCGCGTTGGGGGCGGGATCGGGGGACAGTCTTAAAATGGACATGTCGGGCTCGGGCTGGTGAAGAGGCGGCTAGGGGCGGATGGAGGAACTATCTATGAACATGTCGGTACG

>TU4933-GaLu96scf_4-1417458-1418012 AAGTCCTCGGTGTCCGCCATTCCATGGCGCAGCGGTCGCTCTCCGAGCGCACCCGTAAAAGGTGGCCCGCCCGGGTCAAGCTTACAGCAGCGTTCGGTGAGGATACTAGTCTCCGTTGCCGACCTTCACTATCGGGACGAAAAGACAGTTCGATCTTTTCTAATAGGAGGACGCGGCGGCGCTCCACGGTGCTAAACTAGCGGAGCTGGAAGAGGGCCGTGACATCACGCCCAGCGAATTCAACGCTTGGGTTGTTGAGCGGTGACGACATACCTAAACGGTGCGCTCCGAAGGCCCCGTGGGCTTCTTCCTGGATACCTTTCAGAAGGTCACTCGGGTCACATTCCTATGCACGGACGTCTACGTGGAGGCTCTCGCGCTGCAAACGTTCGACGGACGGGCTGTGGAGTGCCTCGAATACGATCCACTCTCTCGCTTGGCGGGCGGGCTGGAGCGCACCCGTGCGTCGCTGCGAGGCGTGACACTGAAAGCTTATTTCCCCGCATTTCGATCTGCCTCCGTCGCCCGCCGCGTCACCAGCCCGTCAACCTGTTG

>TU4935-GaLu96scf_4-1422583-1423868 CAATTCCACTCCACCGTCACGCTTGTCACACCTCCCCCCCTTGGCAATCTCCACGCGAGATCCCACGAGATCCCCCCTCCAGACCTGGCGCCATCCGCCGACGCCGACTCCGACTCGCTCGCTCAATCCGGTCCAGTTCCCTCAAGCGCCTGCCACCACCCAGTCTCCGCCTGTGCACGTTCATACAATTCTCTTTGTCGCCTTTTCCACACTGTTCCCTACTATTCCTCTGCTCTGTATCGCCGCAAACGTGCCCTTCCTCGCCCCCCCGCCTCCAGATCAACGTGCTTCTTCCACCAAGTCCTCAAATTTTCTCCGCAACCTGTCATCGCATGCACGCCCTGTAGCCGACACTCTTCTACGCCGGGCATGCCGCCTGCACCCGCGGAGCACCTTTCGGGTCCTCCAGCGCACCAATAGCGCACTGCCTAGCCCTGCATGCTTCTCTTCCCTCTTCTCCCATATCACTTCCTACCTGCACCCGTCTCAGAACGCCGGTCCGAACGTGTCACAGCAATAGTCCCCTGTATTCCTGTACCTCCTTCTCTGCCACCTTCGATACATCCTCTCTTCTTTGCCGGAGTCTCAATACAACCTTAATTAACGCGCGCACTGGCACACTCCAACCGTTTCGCCGACAAATGACATGCAGATCACAGCACGCCCCCTCCGAATTCACTACCCACGTACGCACATCACTCGCACGCCAGATGCGACCTCACACGTCCGTCTTCGCGCGCGACGCGATTAGCAGATCCTCACGACGTGCAGCTTCCTTTCCCTGCTTGCGCGTGCCGTATCGCTCCCCGTGAGTGCGTCTCTCAAAATATCCGGTTATCCAGGCCGTATCGCCACCTTCCACATCATGCATCCCTGTCAATGGTGTCACCCCCAGGTCCCGGTCACCTCCCCCCCATGCCGTGTTCATCGAAGCCCGGCCTTCGTTCGGCCGCTAGCCTCATCTCGACTGGACGAGTGCCTGCCAGGCGCCTTGTCTCAATCTCGCCATAGTGATGTGTGCACCCTCCGAGTCATTTTCTCCGAATTCCGAATATGGTATGTACCCTACAAGTCATCGTGAACTCACACGTCGCGTCTCCTCCGATTTATCCTCAACGCGCGTACGCGCATTCGATGGACCGCGAACTCGGCCGAGGGCCCGCCCTATGTAGAGAGGAGGTACCCCATCGGTCACCGTACACGTCATCCACAGCCAAGCACTCTCACAGCTGGGGATAGCAACCCCGCACCGGGGGCCCAAGGTGACACCGCGTGAGGTATAGGGC

>TU4940-GaLu96scf_4-1440466-1440751 GTAGAGCGCCTTCGTCATGTTATTATGAGATGGTATAGCAACGCACTTGCGGAGACGATGTCGTCCTCCATCCCACCTCCTCTGCTGTGTACGCCCACACGTTCAGCGCGCACTAGTGTGGCACAATAACAGACCGTCGGGGTCTTCGGGATGCATTTTTGTTTTACCTGCACACTGATTCCCTGTAGATAGCGTATTCTAGTCCCGTACTGGACCACCTCCCTGGGCTACGTAGCAGCGCCGACCGTGGCGCGAGTGCCGTCTGTCGTGTTCGCAGAGCAGCCCT

>TU4953-GaLu96scf_4-1801084-1801741 CAAAGCTAGCGCTGTAGGAGCTTGCCTTGGCAATCTGTTGGAAATGATTTACGGCCGTGTGGATCGAGGATCCAGATGAGGGCGTCGATAGGGCTTTGAGCAGTTGACAATATCCCACAGAAAACGCGACGGCTGCCCCGAAATGTATGGTCGTCGAGACGTACAAAATGATCAGACCGCTGGACATCAAAGCGGTGGAACGTTGCCTGATATTTCGGCGGCTAGTAGAAGAGTATAAGACCACGATTACGGCCTTATTTTATAGAGGAAGAACTCACATCAAGTGTCCCGTAGAAACGATCGACAGGATTGTGAGGACGCCTCGAATGATCTGGTTATGTTGCAAACGTTCAGCCTCACCATAGAGGACCGCTGACGACACCCGATCAACAAGTACATTATAAATCCCAGGCGTGTAGAGTTGGAGTGCCGCCACGGTGGGATCTTCCATGGGTTCGATGGCAAGCGAGACTACAGTGACTCAAAGGACAAGGGTTGTCGACGGGCTGAGCCTGGAGTAGCAGGGGTTGTCCACGCTTATCTTGGGGCCCATGGCTTGAGGTTGAGGCCTTGAGGAGACGCGCTACAGCCTGTATCAGATGCGTGCCGAGCGGAGAAAGCCTCCCCAGAGTCCCCACTTCAACTTGACGCTGGCTGC

>TU5007-GaLu96scf_40-118044-118716 CGAATGTTACGCTACCCTCTATTTGACGAGCGCAGTACCTGGCACACCATACGCTCCGTGGCGGACTGAGCCATTGGCCTAACTCATGTGGTTGCCTTTGATAAGCTCCCTGCATGTGCGTAGCCTCCAACCTGCGCGTCACGCAACATACTAAGGACCGTAACGTACATGAGAAGACCGCTGGGCACATTGTCCGTAGCGTGGTCAATACCGATGGCGGACTCGTCACTGTGCCCGGCCCTTGATACGTGTCAGCGCACAGTAGTTTGAACTGAACTTACAAGATCCCTGCTTATCGCTTGGAGCTATGGATGAAAAAGGAGGTCCAGCGGGGGAGGCAAAAGGGGAAGAGAACCTGCAGCCCGAAGGAGAGCCGTTGAGCAGAGGGCTGGGGGCGCAGATGGTGATGAGTCGGATGGAAGACGCAAGTTTTTGGCGCCGGGACCCCAAGGACGTTAAGTCAACTGTCCTGTGCCAGCGTGTCTAGCGGCACTCATGGGATTCCACGGGGTGGAATGCAGTCCAGTGGAAAGCCCATGGGGCTTCGGCTGGCTTGCCAACAGGAATCCGCCTGGTTCCGAAACCATTGGATTCCGGGTGGCAGCCTCGGTGATTACCAATGATCGTGCTAGGGCATTTCGCTAGGCGCCTCTTCCCCTTGGATTCCGGTCGC

>TU5009-GaLu96scf_40-124132-124553 TTGCAAGCCACACGACGCCAACCAACCCACCCATACCCTATTTTCATCTCTTCTTGTCTCACCGTACTGCAATTCATCCGGATCGTGCGATGAGACTCAGAGGAGACTTCGACGGCCCACACGTGGCAAGATTTTCGTGGCAGTCAAAATCGACGATACCATCAACAATGGACGTATAGTCGACGTCGCAAACATCCGACGGCGGCGAGAAGGGGTGAGACCTCCAAATATTTGCCGCAACAAAGGTATTTGGCAAGAAGAACTGAGTAGTACGTGACGGCCGCTTGGATATCTGATTTCACTTGAACCCCCGAAATCATGTAAGTAGCGCTGCTTTAGTTGACGTCTCGTTTACAAACGGTCTTCTTGGCGAATGTCAACAGGGCCGGTTAACGTTGTAGGCTGGCTGTCAGTACTAGTGA

>TU5019-GaLu96scf_41-11048-11272 GCACAGAATCGAGCTCGAGAATGAGAGGATAGCCGATATATCGCTCGAGGGGTGGTCGTACGGGTACAGGAGTCTATCTACACAGGTCAGGGCCAGCGGCAGAGACCAGGACACCAAGACAAACACGAACACACAACAGCCACAGTGCTACAGCCACAACACTACACTAGGTACCATAGAGTGGAATCCCAGCACAGAACGGGCAACAGCCCCATCACCGCCAAC

>TU501-GaLu96scf_1-4012542-4013384 CGGGCGTCATCCCCCGCGCCATCGTCATCTGTATGTAAACGAAGCCCGCGAGTGGCCCCGCGACGCCGCCTCTTCTCAACCTGTCAGGGATAACGCGAATACCACCGGGCCTTGGCCGCGCTGACAACTTGGTTAGCTCGTGCATTGCGTCGCACAGCACCCACCTAGGCTGCACGGGGTTCCAATGCGTTCGGCGGGACGCATAAGCAGCACCCGCTCCTCGCAGCCGGCCTGAGAAGAGATGCCCAAGCGTGGGCATCGACGCATTCCAGCTCGTGGCCTTAATCAATGACAAACGCTGTTGGACCCTCAAAGAGGTTAGCCCCGCCCGCTCCCACCCAGCGCTCGCTCGCGTACCGAGCCCTGGCGAACACTTACTCGATGCGCATACAGGAGAGGCAGCTGAGTGCGGGCCACAGGCCTTAGTCGGAAGAGAACTCCTGAGCCCGGTGGCCGGCACTGCGCCGCGCGCGCCGTGGAGCAAGCAAAGGCCTCATGGCCGCTCATATCGACGGGAGGGCGTTCTGGAGCACAGGGAAGGTCACAAAAATATGGGCAATGCACCCTGAATAGCCATAGCCAGGAAGCACCCCGCAGGTCCAGCCACACGGAAGCTATTATGGCGCGCAAAATAGCTCGAGTCACCAACGACTAAACATTGCGTCGCCCGCCCGCCTCGTGCGGGGTGAAAGAAGGCAGAAGAGACGCAAAAAGAAGACGCGAATGGGCGGGTGGAGCGGGTGAAGATAATGGTCGGGAAATGAGATCTGGGGCGACGATGCAGATTGGGTAGTAGACTGGCATCCCAGTGGCCCCTGGTCCTGGCGCTGATGCTCCCACATA

>TU5030-GaLu96scf_41-45162-45513 AACAACCCTGAGGCCCGCCACCACTGGCACCTCTCAGTCATCGGGCAGACACCAGTTCTGCTTCCCACTCGAAAGTGTTCTAAGCGGCAGACGCATTCTTCGAGGTCCCGGAGACTCTTGTGTGCTGCTACTGATCTCCACAGATTTGGCGTGCTGACTCGGTCAACTCACATGCGCCAGCTCCTTTCCCACAGGCCTCTCCGCTATCTACGTCCAGCCATCTCCAGTCCCAGTGCATTTCCGGATCCGATCCTGCGATGACAATTAATCTCAACTTGGCACGGCCCCAATCACCCTTGATGTCATCGAATTCTGGCCGTGCTCCGACTAAGTACCACTGACAATAGGCCCT

>TU506-GaLu96scf_1-4027268-4028060 GATTAGTTACTTTAACTTATCACGCAGCGCAGGCACGCAGACACGCTGGCGTGCATGGATGTTGACGTCGACATGCCCAGAGGACACGAACTTCATCACATTCTTCACGGGCATCGGAACATTCATAATATGTATCACACGGACATTAATGGACACAGTACATGCATGGTACAAAATCACTAACAAAATACGTACCGCGCAACCCTGGTGATGATACATGAAATACGCAAGGATCCTGGAGAAGATCGATGGTTAGCACTCGAACGAACAAAAGAGTTCATGAGCTTCAAAGTTTCTTCAGAGACTTGAATGGTTCCCGAAGGAATCGCACCCATTGTGCGGAGCACATCGTAAACGTTTGCAGAATATTTTCATCATAGGAGGGTAGGACTTTATCAAGCGTAGTAGATATTGAGGCTTACCTCGCTGAACCAACGTCACATGAAGGCGCGACGACAGCAAACACACGTCACGCGCCTAGAGATGCCTGGGAGATAAAATGAGTCAGTTCTCTGCCTCGCAGATGTATAAAGAATCGTTTCAGTGGAAAAAATGACATGTTATTCTCATGACCAGGAGGTAGTCGAAGTGAGCTGAGTCCAATTCTCATCTCGAGACCAAGGGGCAACTTGAAGGAGACTCACCTTTCACGTGTACAAAGAACCTCCAGCAACCTGTCCGCAATGTCTCGCGTTTACGCACGCTGTACCCCAAGCCTCCCCAGAACGCGCAACAGCTTGCTGTGCGCTAGACAAGAACCGTGAGTGGATGAGGGAAACAAGTTCAAGTTCAA

>TU5106-GaLu96scf_43-72411-73348 CGATGCAGAGGAACTTCGCCGCCAGGAAACGGACGCTGCGCAGGGGTCAGGCAGGCGGGAGAGGTGAATCTGTGGACAAGATGGGAGGGCAATCGTGACTAACCTCCACTGACGCGAAGCTTCAAGCTTGAAGGCGAGGGGGGAGGGCAAGACCGAGCTGAAAAGATGGATGGCGACGATGGCCGCAGAAGCCAGCGAGGTATAACGGAACCAGACCTACCACAAGAAACAATACATGCATGACAGCATTGCAATGCGCTTCCCCCCGGCGAATGAATGAAAGCAACAACATTGACGTACCTGCGAACCTCACAAACACAAACCCTCACGTGCACCGTGAACATCTGCCTGGTGGAGCATCAAAGCGCATATAGTCTGTAACAGTATTAAATGCGGAGAGAAGGACGCATACAATGACTTACCGGCAGCTGTAGAGGACCGCGCTATGGGGGGCCACAAGGGGAAGCTTGGGGATGTGCGTGCTGGAGGGAGATGGCTGACCGCGCCGCTCATCTGGTGAGGGCGGTGCCCGGATGGCTTGAGGCGAAGTAGGGTTATGGTGGGCGAGCGAGTCCAAGTGTTGAAGTCAACAGCGAAGAGGGCGAGCAGTCTTTTATCAGATGGGAAGCCGAATGACAGCTCATGAACCAGGACTCGGAGCCCGTGATAACGCACGGACCCCTCACAGAAGCGCACGGACCTCGTCCGATTGTAGCAGATAGCCCACTTGATAATCCTCGTATCCTTCTCCTCTTCGTGTTGCGCGGCCTACGGAGCCCAGACAGATACAAGAACAAATGAAACCATCACTCACCGCAGTGTGCGTGCCCGACTCGGAGAGGGTTTGATCTGGAGGCGCGCGGATGCTCCTCCTATTGATATGAGGCTATATATATGAGCATGAGAGCGTACTTTGGAGGGAACGACCAGGGACGGAG

>TU5111-GaLu96scf_43-157498-158542 CCACCCAGGTCTCTTTCTGTGACCACCCGGTACGTGTCCTCCTTCAGTGTCGTTTTATATTCGGTTCTCCGACTGTAACGCGTTATTATACGGTGCCAAATGCCTCTTTGACCCATGCATCGAACGACGACCCGCCGTTTCGGAAAGCAAGATTGTCAGACACATACCGTCCTCAAGTTCTTTCTCCGGCTTGGCTCTCCGCTCTGGATACTGCTGCATACATCCAGCTATGTACGTCGTCCCACATATAGCACCTCCCGAGCTCTTAATTTACTAGCAAAGTCATTACTAGTTCCGACATAGCGTCTACAAGAGCCTCTCCCCGGGAGATGGGCAAACTGCACAAGGAGGGCACGTTATGAATTGCTCACACCGTACTGCGTATGGAACGCCTCGCTTCCCGAATGTACGTCCTATCTCTTCCAGAGAACGGCTGTCCTTGGTTGCTGTTTGGCAGAATGTTCTCCCGTATATGGCCTTTGCGTACGTCTCTTTACCGAATGTTAATCCGTGAACAATCACTGATCTGTCATTATCGCCGGTTTGTATGTAGGATGAGCAGCCTTCTGGACCTCAAAGTGAGTGTGTCCGTCAGTGTCGCCAGAGCGTACGCACATATGCTCAAGTAGCATTCCTCCGTCGACGTGACATTTCCGCCAGCTCCTCCACCAATCCCTACTCCTTTGGTGCACCTCTTTCCATTTAGCCCGAGTGACCCCCAAGAGCATTGCAGCATCAGGCACCTAATGGCTCGATGCATGCATAGTCGACCGGAGAACCGCGAAGACTGCGGCAGCACGAAACAGTGCCTCTCACCACTGACCACGCATCTATGACCACGGGCTGCGGCGCAATCACCAGACTCGCAGTGAGGCTCACGATCCAGCTGCTCACCATTTCCGTTCTCCGCGCCGAAACGCCTGGATAGGATCACGCTCAACCGAAAGGTTCACCTAACCGCAACGTGCAAAACGGCTTCGGCTACATCTCGTGAAGCAACTTGCTGTTCGAAGGAGGGCAGGACCTAGCTCAGAGCTGTCGCCCT

>TU5152-GaLu96scf_45-40077-40631 ACTGCGCCGACTTATGCACAGGTTACTTACGGTCGTCGGTGGGGAGCTCGAGTGTTAACGTTCGATCGGGCGGCCTAGCGGCGTGCTGCCGCCTTGCATGCAATCCGCCCATCTGTGGCGTCGCACGTATACCGTATACAATGTACATACACAACCCAACTTCCAAGTCGGCAGACGGCCACGGGCCCGCGCGATCCCTCGTGATCAAGTCGTATACGTACCTCCAACTCGCTTAAGTTACGTACGACGTTAACTAGCACTGCGGTACGCCGGGTCGGATCCGGAGAGGGGGAGCAGCTGCACGCGCTATACTGTATATCGGGTACGGGGGATCCAGGTGTGGCCAAGGCCTCCGTCGACGACGCTCGAGCCTCGACCAACGCTTGCTTTTATGTAAAATTGGTTGTTTCTATCATAGTCCTCGAGCGTCGACGTGCGGTGCTAGCTTCCTAGGCCTCCTCGGTGGAACGTCGCGATCGCTGTGGGTGGGGATGTCGATCTCCGCGGACCATGCATGATGGAGGCGCTCGGAGCGGCGGCGGTGTCATGATCAGC

>TU51-GaLu96scf_1-303493-303754 ATGGCCCCTCTGAAAAGTCGAATTATCATTATCTCGCTGCTCAACGCTGTCAGAGCACTACCGCATGGGGGACCTCTATCCTTGGACTGCATCTGCCAATTTCCCACCGCTGCTCAGGGTTTGGCGACGTGCCAGCTGTTTTCGCCAGCAGGGCCGTTCCTGTCGAATCTTATCAGGTATTTGAGAATTACCACTGTATCTGACTCTGGATGCTCTTTTGATTGCTGACATCGGCTCAGTTTCCCGTAGGTACCCGCTTTGT

>TU5218-GaLu96scf_47-134753-135154 TGGGAGGGCAATCAAACGCTGTCCTAGCCAACGACATGTGTGACGCGTACACACCATTTTCCTTATGGCGGAGGGCATGCGCTAAACTTGGGTCCTACAGGGTCACAAGCATTCTCGTCTCTCAATTCCTTCTCGAACTGCAAGCGGCGGACGCGCGGATTCGCGCCGGAGGGTCCCAGCTTTCAATCACGAACTCGCGGATCAGCTCCGTTGTCTTCCATCGGGTGGTGGGGTCGATTCGTCACGCATCGGTCACTCCGGAAGAGGCAGACCATGACGTTGGCAGCGGAGGGTCTCTGTTCGAGGACTGAGCAGAAGAGTTCGCGCAGGAGGAGCTGGGCGGGGATCCTGGCAAGGTTGTGCCAGATGCCCGAGAGTGCAGCTCCCCTATGCAATTAGTCG

>TU5227-GaLu96scf_48-24480-24747 CGGACACGACTCGGCCCCTCGCGTACTCGTACAGATGGCCGTACCGGGTCGCTCGCTCGCCTCGTATGCTATCATACACAGGCCGGACCGGATCTCTTCGTGCGCGTGTCTCCCTACGTCGGGAGCTCAGAAGCTGTGGGCTGGGTGAGCCCGCGGGGCCTGTCCGTACCCGACTCCGCTGTCGAGGGCACGGGTCAGGGACTGGATGGCGGTCTTGATGACCACGCGATCCTTGCCCGTCCAGTGCTTGGGCGGCCGAGCGAGGATC

>TU5289-GaLu96scf_5-170550-171277 AAACAGGAGGATCCCTGTCTTTGCATATGAGGGACCTCGCATTCACCAACAGCACCGTGTCGCATTGGCTCGATGGAGGGGTTCCGAGTGCTTTCTCGAAGTGAGCGCATACCCGCAGCGAATATCAAATCGGCAGTCGGCCGTGCGGAGTGCGGGCGGCGCTGGTATGCAGCGTGTGTTCGCGACTTCTCAACGTTCGGTATCGTCCGAGAGGGTCCTTTCTGGACGACACGACGTTGCGCGCGGTCTCGGGCTTGCCACGGATCTCGACCCGTGGTACCGACGCAGTGACGCCGGGCGCACTGACGACCCTGGAATTCTGTGCGGCTGTCGCTGAGAAACGTTCGGGACCTCGGAAGATGCCCTACGTCATACTCGGCAGATAGCTGTGCAGCCGCGCCTCGGATGTGCCTGTCCGGCAACCAAAGGCGCGCGTCCGTCGCTGCACAAGGCCGTGGCCCGGTGGATCATGTAAATTTACCGTACAGTCGCCGTCTCCGAGTCGGTCTCGGGAAACCTTGTACATAGCGTTTGCTGGACACCGAGGCTCTTCGGCGATTGTCGCGCTTGCACCCTAAGTACTGTACAGCACGTTCCCGTTTATGACCTACTATATCGTTTCATCACAAATATTGAAGTGGAATACGTTCCCCTACACACACCCAAAAAACCCTTCCCGTTTTGGCGGGTATAGCATAAATGAAAATAATGGCTTCTGTGACTATTGT

>TU5308-GaLu96scf_5-249349-249602 CCGAAAGCGCGAGGAATATTCGAAGCGAGTCACCTCGGCGGTGCACTCCTTGTACATAACATGCGCGCGGGCCATCCTCCCTGGCGGCCTGAGGACAACTGACCAGTACTGAAGTACTGACCCTCCACCGCGCTCTCCTCGTAGGAGGGACGGCCGGGTCACATGACAAACTCTGGAGTCCTCTGGAGTGCCTCAGAGTTGCTCGGAGTTGATAGCCGACACTCCAGAGAAACTCCAGCTCCGCTCCGCGGCAG

>TU5321-GaLu96scf_5-345650-346325 CCGCGGCCAAGCGCCGCAGCCTGAGCCTGACCAACTGCAGCAAGGAGCAAGGACTGGGATGAGCGCACGGCCAAGGCACAAGCATACACGTGGCACAAATAATCACAGGGCGGCATGATAGCGGCCTAAAATCCCTCCTCCTCCGTAAGGCGGAGACCGGCGAGAATCAAAGGGAGGAGGTTTAGGATCGAAGGTCCCCGGGAGGGGCCAGGAAGGTGAGCAAAGTTGTGGGCTGAGGTCACGGTGTAGTGGGCAGTGGGAGCGTTCCTGCGATTAAAAACTGGGCCGTGGGCGGATCTGGAGTAATAAGAAGCCTGTAAAAACACATCGAATTGAGACGGACACGTAGAACAGTGGGAATTCCGAATCACCCACATTGCAAGGCATCTCCGGTCCTTCTCGCTCCTCTTTACAACCGTCACCCTTGGTTAGGTTTGAAAGTGTCTCTTATATAGGAGCTGACGGTCATACTCGTGGACAATGGAGTTGAAAGGACGGATGTACAGTGTCCAGGGACCTCGACCGTAACATCCGAGTCGTGACTGGTGACCGTCGGTAATCTTCACGTTCGTTCTGCAGTGCTTCGCGCCGTGTCTTAGTGTAGGTATGAGGACTGCGAAGGATTGCAGGGGATGAATGTCGAGAAACAGAAGTTTTTGATACAAAGCCATACAGG

>TU5329-GaLu96scf_5-388017-388623 GGGTCTCAGGAGCGGTCCCTGTAACTCAAACCCCCATACGCTCTATGGCGACCGCGCCTAGGTGACGACGGCTCGGGATGGCTATATGCGTGCGCGTTCTTCGCCACGCTGCGTGCTATACTATTAGTATTACTTGCGGCACCATGCACGTCGTGACCGTATTACGCCCACCGCATTAGCCTCGTACGACTACGGCGCTGCGGTAAGGGATGTCCGCCGCCAACAAGGGTCGGATTGGACTCTGGAGCCATAGACCCGAAGGAGTCGGGTGCCGGCATCCCATCACATGTCACCAGTCACTGTCAGGGTCACAGTTGACATGAAAGCCTGTTTGAAGGGACCCAGATCTAGAATTCGGGGGGCGGAGGATTAAGGAAGCCGGGCTGTGGCGCGATGGAGGCTGGCGCGAAGAGGGAGCGAGCCAAGGCGGGGGAGCGGGTTTCCATGGCCGGCTTCGGGATGTCTGGCGGTTCTTGCCCCGAAGCGCGGGGAGCGTATCTATGCATGTTTCTGTTTCTCCGGCTTTTCCACGTCCGCGTCGCGCATCCGGCATCCTTTGTGTAAATGCCTGTCAGTACGGTCGAAGTTATTGGGCTTCCCTATGAGT

>TU532-GaLu96scf_1-4151014-4151342 TGCAGCGTAATCGGGCACAGGATGTTGATAGGTCGGGGGTTGAAAGTGGTGTTAGATGGACGTCGTATGCTCAAAAGACGACGGAAGCTTGATAAGAGGAACCAAGTTCTTTAGATGACAAGTGGCAGGAAGCAAAGAGAAGATATATTTGAGCGTAGATACGGGGCAGAAGATGAGCGTAGTGCGGCAGAATTCAACGTCGGGGCGTGCGTGGCGGAGGGCGGGACTCGGAGAGAGACGCGCCGTGCAGGGCCTCACGTAATGTTCATGACTGCGCTCCAAATGGAAAAGTGGGCGGTGTGCCCAAAATGGACATACAGACGCCGGAT

>TU5334-GaLu96scf_5-403955-404387 TTTGACACAGTGGCATATCCCGTCGAGGGTCGTTCAAATTGCTGCCGCTTGGGAGGTGATGCATGATAATCCAGTTGTCGATTCTGGGTCACCCTGCTGAAACACCATAGGGTGACGGGTAGTATAATACCGCTACCGAATTTGAACGGGTCCATGGGTTGATCGCATTTCAGAATTTTCATGCGCATGCGATGTTACTGAGTATTGCGTCGCGCCCCGCCGATGGGCTTCCCAGCTCCTGACCGTCATCACGTCATACATGCAACGGGCTTCGGGGCATCTCCATCATTGCACCCCCTCACGCTCGTGCATCTGGCACCTGAGTTGTTCGGATGAGTTGCTCGTGCCCCATATGGTATTCCGGACATTCCAGTTTGCCACACAGAGGGCACGAGTTCGAGCTCGCAGCAACAGATCAAGTTCCCTGCCCGTC

>TU5341-GaLu96scf_5-432326-432852 CACAGCCTTCTTGGCCACCAATTGTAAGTGCCTTCCTTCCCAAGTGAGCCTGCACCTAGCCTGTCCTCCTCATGTCTACTCGAACCAAACTCTACTACATACGTAGGTTACGGACCGTTCTCATTGAGACAGACCCAGGTTTGGACGGGCCTGCCAGGGACGCAGATTACAGTTGTCAGCTCCCAGGGGGTTAAGACCAAAAAACACTGACAGACGGGACGTAGAGCCTCCGGACAGTTTAGGGTGAATATAGCTGCGGATGCGGAATCAGTGCGGAAATCCTTCTTGCGCCCTCAGTCTACGTTGATCATCCTTGTCCGCCACTGGCAGTCTGGCCATGGTTTAGTCGATTGTGCGACCGAACCTGTCATTGTCAAGCAGTGTCCCAATAGTGTCCTCTACTTATTTTAGCGATGCCGCCTCCGTCACAAGGATATAGTCCAGACCCGCACTTGCCAGGCGCTGTATAGTCAATCCAAATTGCTACAGTACTTATGATCTCAGAGTCCTTGGAATCTCGGCGAGTT

>TU5347-GaLu96scf_5-615750-616586 ACCTGTAGGCTGTGCAGGGCGTGAGCCACCTGGATCGGAAGACATCCGGCTGCACCGGCTGGCACGGGTTCGATGTTCAACCTCCGCCACTAGTATAGTGTCCGTGCGGTCTGGAGGCGGACTCCCAACTTGCCATCTGTGAGATCTGTGAGAACGGACCCTGGACGTAACTCATCTTCTGTTCGCAGCTTGTATTACTCCACATTGTCAAGGCCAAGCGTTGCGTGCCACCTACGCGATTCTCCAGGCGCATCACAGGGTCAAGAGCGTCGAGGAATGATCCAGAGGCTGCGCAACACTACAGTAAACGTCTTCGAGATGTCCTGAGATGTCGCGACTCGCGGTGATGGTCCCGCGCGTCGAGTCGGCCATGGATAGATAGCCGATCGCGTCACCCGGGCCTGCTATTGGGCCGTCGGGGCGGCGCCCGCTTCCTCCGGTTATTGCCAAGCGCACAACCTATTCTTGCTCGGGAGTAAGTAAGAACAGAATCAGAATGTGGTCTAATGCGGTATGTAAGAATAACTGCACATACTCAAGTAAGCATCGAGAATCGATTTTTCATCTGTTACGTTGTGTTCCAGCCTCCTGGGTGCTGCCCACGCAGCGCCGTTTGATCAGCGACAGCTTGGCAGTCGAGTCGCGCTGGGCTGCCAGAGGACGTACCTGAAGAAGCGACCATACCCATCGGCGGTTCTCACCATGAAAACACCCTTTTTGTCGCGCGCCAAGAATTTCACAAGGCTGGTTGGCACCACCCGGTGTTCATGAGCATCGTCGATGGCCCGAGTCGGCGTGTATGTGCGAGTACCTCCGATGTCGATATCGCTGCCAATT

>TU5349-GaLu96scf_5-619450-619938 TGTGTACGTTCTGATACCACGCCTCGCTCCGGCCCTACCTAACGGCTGAAAGGCGGTCAGAATGTGTACGATGTAATGCGGTTTATCGAACGACAATCGCAGGGCCGCCGTGCGCGCGAAATTAGACAGGGCTCAAAGACCGACCCGGATGCTCACTAGTCCGGCAATGATCTGTACGTCAAGCGACCCGGAATTTTCATCGGAGGATTTGCAAACAGACAGGAGATGACCAACGTGGAAGCGGCCGCTCCGCCGGATAGTAGCATGCGCGCGTTGTGAAGCCAAGCTCCGAGGTTGAGACCCAACCGTAGGATCAGGGCACCAACAGAACGGGTCTTACTCTCGGCGATGTTCATCTTGGGCTCGGTGCTGCCTAAATCTGCCAAGAACGTTTTATGAGCGTCGTGAGGACCTTCTCGATATCTGATAACTCACCATGAGAGGTAAGACTTGCCCATGCCCACTGATCCTTGTCGACCACGGACCTTC

>TU5356-GaLu96scf_5-675828-676696 CTAGTACTTACTGGTTACTTTTGACTTACTTTAACTTATATGCTTGTAGTACACGCCACATGGCAGGTTGAGAATCCGTTTCACAGCCACTGTGGGGATGCGAACTTGAATCGGACCCCGCTGCCTACAGCCTTGGGATGTTCCTACTCGTACTCGTCAGTATCCGCACATGTCATCAACAAACACCCCAATACCCCAATACTCATACTCGAGAGGTGGTACATGCGCATCACCACGTCCTCTCGCTTGACCTCCGGACGCACGACGCTACTGGCCTCCGAAATCGCGGCGTAGACTCGGCCCCGGACTCGGAGTCAAGCCGTCAGTACTAGTGGACTCGTATTACAATATACAGTACGATGCCGAGAAGAGGCCAGACGCGCGATAATGTCGCTTGACTTGCAGAGCGTCACGCTCATCATGAAGCCTAAGGAGTCGTGTCCTTAGCGGGGTCCTGGTCCTTGTGGGGAGCAAGTCGGTCCTCCCGTTCAGTCACTATCTGCGGGAAAGAGACGGAAGGTCAGTATCTCCCTGTCTTTCCTTGCCAACCCTGCCCGGTTGCAGTTTAGTCGGCCTTTATGACTTGCAAGCAGAGTTCTACGGTCTACTGACCTCAATGCGATCCTCGGAACCGCAGGCTTCAAGGCTTCTTATCGGTCCTAGCGTCTTCAAGTTACGGCAAACATCTGGGGTGCGTCCTTTGAGCTGCAATGAGCCGCCAAGAGACTGAAGCCCGACCATCGCAGCCCGAGTTCACATTGGAGCGAGGTTCTGGCCGCACCGGCTCACGACAGTGGGCCCGTTCAGGTGCCCCGCGAAAGTGCTTGGGGTTGAACATGACCACTGTCAGTGACCCCATTCCCCGATCC

>TU5359-GaLu96scf_5-738180-739184 GGCGATGAGGGGGACCCGAGAAATGATCTCATGTACCGGATGACCACGAGGCCGTATCCAATGGGTAGTCGCCGCTCGAGACGCCCCTTGCGCTCGACCTTTGTGTCGGAGGAGAAGGGGTCTCCAGATCATATCAGAATACATTTCCAAATGTCGCCATCAATGCAGTAGCAGAGGCTTCTGGGTTCCTGGTTGAATGCCGATAACAGAGCCCTTCATAATGAGACTTGAACGGACCGGCACCGACCACTTGCCTATCAAGAAGGCGGCGTATTGGTGCAGAGGAGGGTTGCGCGCAGCCAACACCGGGCCTGCTCCCAAGCCGCGACCATATGGGCTAGTGAACGTCTCGAGGTAAGGCGCGCGATAAACGCCTAGCACATCCAAGTTCCAGCCGGATTTCCAGGCTGGAATGCCTTCGCCAGGCACACGCATACGCCATGGTGGTGACCAATGGAACTCATTCTCACCCCAGCCACTGACAACGCGCTTTGTCAGGCCCTCAGCGATGCCGGGTAAGTCGGGGGGAGTAGGCGTGGGGAATGCGGTAGGGCGAGCGGGACAAAGGGAGGCCGTACGCCCTACCAGGCGGCGACGTCGAAGGCGGCGACGTCATTGACCGGGGACGGAATTGCTGCAAGCGGGAACGCTCGCTCGAACCGGCAACCCTACGCCGTTCCTACGTCAAGTCTAAATAGCGGTGAAAGCCGGACGCGGCGCGCTACAGCATAGGATGGCGTACCCACACACCTTCGGGAGCGACGCCTGGGAGTAGTGGGTTGGTGGGAACACAGAGATACGCGTGAGATGCGGGCCAGAAGCTGCAGCCTGCATATGCCACCAGGGTCGCGAGACGATCTCCTGGCCGCACGGGCAGATCACGACATCGACGTCGCGGTTGTTCGGTGACAGTAAGATCCTCTGCGCACCATTCCGTCGCTTGCCCCTTGGGCCTTCTGACGGTGCGCTGACCGGAGAACGGCTCGCGGGTTCTCAAGGGTGTGC

>TU5367-GaLu96scf_5-856392-857018 ATCTGGCATTGCTCTCCAACACACTGCGTATCTCGCTGTTGTTCGCCAAAGGTCTTCCATCCACAAGGATGGGATTTCCCTGCATTTATATCTACCGCATCCTACATACTGGGGTACAGTAAGCTCAGGGCTTCTGGGCCATACGCCGGACCAGGGCAACCAGTGTAGAAACGTCAAATGTCACCACCAAGGGTGAGTCGATAGATCGTCTCAGTGATCATGGTCGAGAGCTGATGCAGATGACGCTTAGATAGGACACCTCCTTGGCTGTGGCGCCGCCCACATCGAGATTCAGCAGCTCCTCTGTTCTGATTCGCACATACGCTCGCTCAGCGGCACACGCCCGCCGCCAGAGCACGAACTGACAGCCCGCCGGGTATCGGCGGGATGATGTGCGGTCAGGGGGCAACCGCTCGGTGGAATTCCGAGGGCAACCCCTTCATCGTGGCCGCCGAACCGGTGTCCGGGGTCTTTGATGGCAGAATTCTACCAATCTTCCGACAAACTGCTTCTCGCAACTTCTCACACCGTCCCCACGTCTCAACACTTTAGATTTAACATGTATGGGCCATTGTCTAGCACATGAAGTGGTTTTTGTATCATATTACTCTGATAGTACTCGAGATG

>TU5374-GaLu96scf_5-902659-902936 TGAATTTAACTATCAAAGGTAATTGGAGCACAGATAGAGTGAGCTTGGATCTCTGGCATGCACGAGTACGATGGTACAAGGGAGCGGGAGCGCAATGCAGCAACGGCTTGAGTAGGCAGGAGTACTAGTAAGTACATGGCAGGATTGGAGGCTGTGATGGCAGGAGGCATGCAAGCGGAGGAGGCCATCATCGTCTAGGTGAACGGGAGAGCTGAACTGGGGAGGAGGCAGGAGGAAGGGAGCCTTAGCCTGGCCTTAGCCTCAGGGAACGTGTTACG

>TU5376-GaLu96scf_5-906876-907648 CCACGACGCTCAGTTGCATGCGGTTGCAATGAGGTACGTACATTCGACGAGGAATTTCTCAGGACGCTCACATACCAGATCATTTTGCGCTGCGCGCGTCCTGGAGGTCTTAACCAATCTTGCGCAGCGCGCAGGCGCCTTCCGGTCGCCCTTCGAAGGCCCTTCTTGATTAAGGTTCTCCGACGGCGGATTCTCCGGACGTGGCTCGACGGCTTCTTCTCACTACGCCGTGACCCTTGGCATTTCCGTCTGCCAGGAGGCCGCTGGGGCGTGCACGCAACATTATCATATGGCCTCAGGGGACAGGGGCCGCCTCAACGCCGCGCGTCTGGCATTCGAGGTAGACTTTTTCGACGTTCTGATCAGATTCTATCAGCTAAAGAATAAACTTTAGAATCATGTCTTGGTGTGTCCAACGCCCCTCTAACACGCTTTAGGGCTCAGGGTCTTTCACGGAACTGCTTGACTTATGAGACGGGTTTGGCAGGGCGACCGCCGTGAGTGCCTTTAACTCCGTCGCCGTCGGCGTTTTCACCTCGTGCATGGTGGCCGGGTCTTGTCGCAGCGATCAATGCTGTGGAAAACCTGCACGCAAACGTCAGGGGCTTTGCTTTGGGGAGGGAGCACGAAGTCGGCCTTGTGCCCTTCCCTGCTAGGCCCTCGAGGAACAGATCTAGACTCTAGAGACATTTTTCGAGCCGCGCAGGTCGCCCAGTTCGGTTTAAGGCTCGGATTGCTCGAATGAAAGAATTACAAGGTTACTACTAACAACA

>TU5378-GaLu96scf_5-913468-915032 TGGGGCCTCGGGGAACGCCATGCCCGCCCCCGAAAATGTACTTCACGGCACGCCATGAGGCCACTCCGGTTGGATGCTATAAGGCAGGTGAAGTGACTACTCGTGGGGTTTTGGGGCGGATGTATTAGGTCTGTGGGTGACTCAAGTCACAAGTGGCGTGAGCTGATGACATGTGGCGGACAGCGCCAGCACGGAGTCCACACACGCCTCCCGTTCTGATGCAGGAGGCTCGTCGAGGCCTTGGGAGATAGAACCACAGAAGCGGGCCGCTTGGCAGATACATAGAGCGCAGAACGGCAACCTCGGCGATAGAGCAATGTTCATATTTGAGTTCAACAGACGGCGACTATAGGACTCGGGAGAAGCAGCGCGGTGGGGCTGTATTAATTTTGGAGCATCGCACGGACGCAGGTTGCGTGCGTGGAGGAAGCAGGTTGTATTGCGAGTCCTAAGGTAATGCGAAGGCGAAGCCAGGGCTGAGGGATGGAATCGGACGTGTAAGAGTCGAAGATCGCACGGATATGAGGGCGTGAAGCGGCTGTATCGCAGGACAAACGCCCAACGGGGCAACGTCGACACGCCGATCGTCGCGTAACCGACAGATGTGCACAGTTGGCGAAAGCCCTGGGCGGACGCACGTCGCAGCAAGGAGCCGGAATGCCAGACCGAGGAAATAACACACTGCAGGTGAGCCTCGCGAGGTGCGGCGCGTGCACAGCAGCAGCGGTAGAGGTCTCGACGTCGACGCTGTGGTCGAAGTTCTGGGCAATTGCCGTAGCGTAAGTTATGAACAAACTGTCCAGGCCAGACAGTATTCAATTATGGACTGGTGGTGCAGTTGCACAGTAGAATACGGATCATGGGGTGGTCCACACGAAGCCCTAGATGAGTACGAGGCCCGGGACGACGATATTTGACATTTTGCGCTGTTCAAGCCGACAGCAACGATTGTTGGATGCAGATGGACGCGGAACGCGAGGGTTGGAATGCAGTTATTGGATCCCAACGGCAAAGGAGATGGCCAGACAGCAAAAAGATGGTTGCCTAGAAGGAGAGACCCGAACAAGGCAGGCCATGATAGCGACGATATGAAGCGGGTCCGGGAGACAGCCAAAAGGAGGGAACGATAGGGTGAATGGAGACACGCGGGAACAATGGGCAAGGTGAGGCGATACTTGGACGGAGGGCGGATGGACGGAAGGCTAAGTCAGGATGGGGAATCAGATTCGCACATGCATGAGGGAGCGCACGGATTGCGTAGGGCCGAGTCATGAGGGAGAGTGAATGAGACGCGGGCAGGCGATGATATGATGAGCCTGATATGGGGATGTCCCGGATGTGCGCCTGGGCTCATTCCGCAGCGGACAGAGAGGAAGGGGCCCGGGGCAAGCCTGGGGTGGGGAAAGTGGGCGAGCGGTGGGCACAGTAGGGACACAGCCTGAGAGGAAGGGAGAGAGGCCTGTGCGAGAGATCCGGGCGGGAGAACGGCCACTGCGAGTGCCCGCTAGGGTGCAGCATGGGCGCTCTTGGGCGAGTGACTCCAGCATTGTTTGGTCGTGTTTACG

>TU5383-GaLu96scf_5-937208-937780 GGAATGTTTGATTGACGTAGGAATTCCGAGTCAGGCATTCCCCAGTGGCATAAGTACATGCGCACCCTTGCATATTGTAAGTGTTCGTCTTTCTATGTATTTGCCCGGTCACCTTCGATCGTAGGGTCCACCGTGGGGGTACTCACATTTGAACGAAGTAGCGCGAAAGATGAAGATGTTGCGTAGCTTGTTATATCGAAGTCAGCTTGATTACAGACAGGAATAGTCCGGGCTCGATGGGGGATGGTGAGACCGACGTGGACAGCGAGTCGGATACATATCGGAGACACGATCGCGAGATGCGACAGTTTTTCTGGTTTCAAAGCGTGACAAAATGGCCGGAGTAGGCTGCTCATTGTATGCCCGAAAAATGCGTGATACCGATGGAGCCCTGTGATTTTAATTTAGGGGGGATAAGTATTAAGCTCCATGAGGACGGAGACTGGCCGGGCGGGACAGCGTCTTCAGATGCAGGACAGTACTAGTCAATCGACTGGTGCCGAGATGGCCTTGGCGAGACCGGGCTGGCATGTTTGAGTGACTGGTTTGGGGCGAGGGGCAGTAAGTACTGCG

>TU538-GaLu96scf_1-4172331-4173054 TGCACATGACTGAACTCACCCCATTCTGTATTCACCCCATGACAAGCTTCGTATCGATGTATGCACTCGATAGTGTACACTTCTGACGGCGGGTAATGGAGTATCGGAAGATACCTTCACAAAAGGACAATATCATATGTTCCACGGATTTCAACCCGGGAGTCTGGCATCTAAGTAAAGTTCCGAGTTCCTGGCTTCTCCCACAGACTCGACAATTGACGCGACATAAAAAATAGTGAGGATAACTAGGTTTTTCAGCATACATACATACGGCGACGCACCCTCAAGCTCCTACTCTGTCGAACATTCGCGGCGGACCTACAGAAGTAGCGTTTCAAGTAACGTTACAGCTGTTTTCATAACGATAGGCCCCTCGGGCGCATTTTACACACACGGGCGGACATTCGACTCAGGCGACGGCAGTAAGCAGTTCCGATTCGTTGCGCAATGTCGCGGGGGCGGCGCTCCATTCGAGCCCCTCACCCGACGCTGCCGACTTTCGGATATCACCGCTCGTGTGCGCGTAAAACGCGTCCCAGGGGCCTATCGTTATGAAAACAGCTGTAACGCCGGCGCCAGGCTCAAGTCTCTTCGCAGGACACAAGGCTAAGCGCCGAGTGGAAAGTCTTCGCGGGATACAAGCGCCGACTCGGAGTGGAAAGCTCTCAAAACTCTTCGACGTGTCTGTCAGACTCCCTCTATTACACATCGTCGGCACCCTCAG

>TU5391-GaLu96scf_5-1021841-1023370 ACCGGGGGTACTGGACCGCATCATAAGGGTGTGAAGGCTACCCTGGCCGTTGCCAAGAGCAGACATTGCTGAAGGTTAACGTTCAAAACGGAGTCGGGAGGCGCGAGATGGGGGGTAGCCTTGTCGTGCAATGAGGTTGATGTAGAGATAGAGAGAAAAGAGGCAGGTATCCAGAGGAGCACGGGAGAGGCTACGAGCGGAGAGAGTGAGGTCACACAGCAAAGGAAGGAGAATATGAACTTGGTTCAGGGCTTGCGACCTCGAGGCAGGCGCATGTTCGACTTCCTGTTGACGCCTATTTGCCTCCCATCCAGCGCTACGTATACCTTCACCCCCCGGGCCCCCATACCTGAGGCTGCTCCCCTGAGCCTCACTGCGCAGTCTCCATCGCTCCAGCTACGACGAGACGACTGAGACTCAAGTCCTTGGATGTATGCTGCTATTATGTACCATCTCTCGTCAGGCAGCCATGCAACATTTGCTTCCGTGGCAGCAACCGATCGCATATATAGGGGTCATGATCGAGTCGACACCGCGCCACACATGCACAATCAGCGTCCTTAAGCTTCAGACTTGTCTCTATGTGGCCCCTCCTCAGTGTCTACTTCAATAAGCCGATGTTTCCGCCTGCAATAATGCGGATATCGACGGCCTGCATTGCACGCCAGAAGGAGGGCGTTGACCGAAGATGTCGGCCGAAACGACTTTTTCAGACGAGAGGTGAGCAAACACGCAACTTCTACGGAGCTACGTGTGCCGCGGGTTTTGGTCGATCCGTTTGTCTACATGAAATGCACGCGAACATTATGCGGAACGATGCTTCTGGCGCAAATTGCTGGACGAACTTCATATTCAGTAGGCGGACTCTTGCCTGACTTCAGGTGCTGACGCGTACGGTCTGCGCCGTCTCCTGTGTCGGGACGCGCGCGGACATTCTCTTTTCATCCGCCCTATTCTGGTCTGACGGGGTCATGGATTTCCATTCGAGCCTGTGTGTTTCTCAACTTTCAGCGTCGGTTCTGGAGCTCAATCTGTCTTATGCTTACAATGACCTAGGTTCGACACAAACCCTTCCCTGACCGGGAGAGGACGGTCACCTTGGTATGAGACTAGCAATAACCACGGATCTCTAGTTGGGCAGACATCGTTGTGAACTGAGCGTTCCACGGTGGTCAAGTACAAGTATTTGAAGGTTAATTCGCATGCCAAAGTCTAGTTCAACGAAGGATATCAACAACAATGCAACGCAAACATAGCAGGACAGGAGGTCATACTCAAGATGGCGATATTTGTCCCATCATGACCATCCTATTGCTAGGCATCCTCGATTGTAGTATGTATCAGAGCGGTTAGCGTTGTTTTCTAGGCCGCTTCTGGAGAAGTTGGGACAAGGGAGATCGGGTACTTAGGGAAGGTCGATGCTCAACGTTCAACACTGGCACAGCAGGCCGGTCACATAGTTCCGTTGTATCTGGACTGCGCGAATGCTAACTACACAAGTCTCGTTGGGACACGGAGCAGGAACACGTT

>TU5397-GaLu96scf_5-1044015-1044536 GGAAAGCGGATTGTTTTACACGCGCACAACGCCGCCGCGCAACTTCTTATACTTCCCGCAGCATTAGTGAGGTCTCCAGCTAGCCCCCCATGGCATGCAGAACGATGTCATGGCCCAGTCTGGCTCGTTGCTGAAAGCCGCAGTCGAGGAGCCGTGGAGGCCCGCCGTTTCAGCCGCCGCGTCGAACCCCTAGATTGCCAGCGTTCGTGACAGGGGTGGTTACTCCGCATGGTGGTTCGCTAACCTTCCCTGACGTGCCTGCGGCAACCGATCGAACCGCTGAGCAGCATGGTTGAAGAGTCGAAGGCTAGACGTGTTCTCACGGGGAGTCATAGAATGAAGGTATAGATGTGGATCCGGTATGGACTGCGACTGGTTTTCGACCGGTCAGTATGAGTGTATCGTCCAAGAGAAACGGACCGCAGTCGTCTAATTATTCTGGTATTTTTAATTAAAGCACAGAAATCCCAAAACACGGACCTGTAAGCCATGTACGATCATGTGTATGATGAAATGTTCTGC

>TU5416-GaLu96scf_5-1189260-1189860 AGTACTACTACTACTCTCTCCGTTCTCAGACTCTCCTCCGAGTCCAATCAACTTCCAATCGCAGCTTGGGCATCCTGAATACACCCTTCCTTCAGACATCAATCAAGCTAACACCCTTCTCGTTACTCCCATCCTCACTTAGTACTTACTGTACTGGAACTCAGACAGTTACCATACTTAAGGTCCGACCTTATCAGGACTTTATCCGAGCCAAAGTCTGATCCACATTTCCAGGGGTTCGTTCCAGCCACTTTGTCGCATTCCACTCGACGCCCATCCACTGCGATCTGGTTGTCTGTGATTTGCACGCCATCGCATCGCCGTCCTATTGAAAGTAGTCAGGCGATATCAAGTTCCGTCCCAAGCCAACCTCGCCCCCTCATACCCGACGCCCATTGCTCAATGCCTGTTGTTCGTAAGCTTGTGATTAGTTTCGGTCTCGACAAGTCCAGAAGGAGCAGCATTCTAGAGTGTCGCACCATGTCAGGCGCACGCCCGCATTAGGTGAGTCTGCACGCCGCTGGAATGCATACGCATACTTATACTGCTTGGGCCAGGTCCGTACCCTCTTGTACCCCAGAATTTTAACGCCATCATGATT

>TU5458-GaLu96scf_5-1423141-1424134 AGCGCAGTCTGCAGCGTATGCTCCCATATATGCACCTTTGCTTTCTCCAATCCAGTCATCTTCTTCAAGTCCTACGCAATCACATTAATTAATACAAGAGACTTCTAGATACGGTGTCAAAGATGCCGCACGGGATAGCAACTGGCTCCCCTCTCCAAGAGAGCATACCGCAAGAGCGCACCTCTTCCGCTGTCTCGGTACAGGTACTCGTTATCTCCACTTTCATTGTTCTGATGTAAGCGAATTCATATTTATGTCGTCGTTCTCATAAGATAATCTCACGACGTATTGCCATCCTATAGCACGTATGGTCTCTCCCTAGACCAAACATACCGCTACTTTAGCTGCTACCCGAAGGTTGCATTGAGCATACATATTGTGGTGAGTGCTTGTGCTGACTGCACCCTAATATTTGTCATTTCAGGCGATTTCTCTGCTTTGATCTCCCGTATGCTGATTCATTACGCTTCTACAGCCTCGTCAGCACCCTGCACTCTGTTTGTATAATGCAAGTGTGGTGCGCGAATCGTATCATTCTGTTCACAGAGATGCTCACATCCAAGTTGTTCCAGAGTCACTCTAGTGGCGCACTCGTACTTTCCCCAGGAGGCTGTTACTCTACCGACTTGATCCTGGGTCTGCCGTGAGTAGGCTGTTCTGCCTAGCACACATCAATTTGGCGTTCATTAACTTTTATTCCAGTTATTTCTGGGAGGGGCATCGCACGAGTTCTTTCCCCCAGCATACCAAGCCTCGAAGAATACTGCCACAGCTCCTCCCCATCACCACAGTTAGCCCCTCGTCATGGCAGGACCGTTGCAGTTCGTTCACATTAACTGTTAGACCGCCGTCAGCGTCATAGTTCATTAGTTTGTTTCCATGCTATCAAGCTTTTTCGTTCGGCGGGTATACTTGAGTAAGTGTTGTTTCTGCCGACCACTAGCCCCCCGAATGGACGGTGAATGGGATGCCGGCTTCTTCAGTCTCCGTAATA

>TU5486-GaLu96scf_5-1590396-1590746 GCTTTCCGTCCCCAGAGTTCAAACCTTCTGCATTGTGTTGGTGGTAACCCTTCTCTTATGAGTGCTGGTGAAGCTGTTGAACAGGACAGAAGTTAACGTAGATTGCCGAGCAGGAGCGCTGGAGATAAGTGAGAGTAGACGTGTGAAGTGGTAACGCGACACCCTCTAGTTCCCTTCCACTGTTCACCGACACGCGTGAACGCACTTCAGCTGCATATAATCCCCCCCCCAAACTCTTGCTCCTCAGAATATCCACAGACCCATTGTACCAACTCTCCCCAACATGGAACAGAAACTCGCGAAGGTCGACGACGTTGCCCGCAAGTCCTTTGACTTTATCGTAGTCGGTCC

>TU5492-GaLu96scf_5-1685421-1685678 AGCGACTGACCCTCCTCTGCCTTCCAAACCCCACTTACTACTCACTGTTTCGTTTGTTGGGCCTTCAATGTCTTCGACACCACTCCCCGCATCCACCAGGCTCTGAACACCACGTCATCGGCCATCAACGTGATGGCGCGGTTGTGCTTCAAGTTGAGCCTTCCCCTGAGTTGAGCTTGACATGGATGTTATCTCAGTCCTGCGTTTGAACTTTGTAGTGCCAGTGCTGGGGTGGAGGAGGAGAGATGCTTTGGTGGT

>TU5515-GaLu96scf_51-75594-76346 GTGATAGTCGTGCATTATTGTGAATGGTAAATTATTTCACATCGACTGCGTATTGAGTAGTCTTAGTCCTGAGGCTGAAGACGGATTACAACGAGGTTAATTCGGAAAACGAATTGTTGACGTCTACGGGAAAATGTGCCGCTACACGTGAGCAGAGTAATCGAGTGGGTGGACCGCACTATGTCTCTGCTAGGGCAGACGTCCATCAAAACAAAGATCGAGGGACCATATAGCTACCCGCCAGCTAAGGGGAAACGTCGAACCGCTCCTGAAATAAGTTAGAGAGGACGGCCGCCGACCACTTCGCTCCGGGTCCGACTCGGAGTGTGTGATAATCCAATGGACGACGCACGGGGGTTGAGCCAAGCCAAGTCGTGGCTTGGTATGCCTCCGAGTACGTCAACGACTCGTCGGAGTCCAGGTGCAGACGGTCATGACTGCGCAGCACATCCAGAGGGAAGGAAATCCGTTCCGAATGCAGCGGGGGACAGACGACCGTGATCCAGGGCTGGAGGGAGACATTTGTCTGGCATGGGCTGATCCTCTCACGCCGTTGGGACCGTGGGCATGCGAGGAGGATTGGCGGGGCCAATACCGTATCGTGTGGGGCAAGAGGGACAGCGCACCTTTTGAAGTAGCGAGCGTCCAGTCGTCACGACCCCATTGACCCCGACAGGGCTACACCCAATGGTGGGCTCAAAGTGCAGCCGATCCGCCCCATGTTATAGAGCACAGTTTCAAAATAGCTAGGTG

>TU5518-GaLu96scf_51-83103-84348 GATTCACAAAGTGTCTATTCTACTACGTGCAGGGTGCTGAAATACGGTCGCTCTAGCATTTTACTCTCCCACTCAGACCGTGGTCATTGCCTTCTTCCCCCTCGTCCTCGCCGGAGCCATCGTCTTCAAGTGCGCCGATTATGCATCACTGACTCCCCACTCATCCCCTCGAGCTCCCTGACGGACGTACAGATTTCTGCCATGTATCCGTCTACCATGTTTTTACCTCGCCTGGCGTGGTTCAAGCTCCCCAGGTGTCGTTAGGTGTCGTTGCGAAGAGATCCCTTGGTGCGTATCGATCACATCGTCACCGCTAGTCTAATTTCTGCGTCCCTGCCCTCGCGAGTTTGTCCAGCATCCCCCTGGACCTCCGTCGGCCGCCCCGGGTAGCCTACATCTTTGAAGCTCCGCGTCTTCCTCGGCGGAAGCCTCTATCGAGCCATCGAGAGCCCAGAGCGCGCTCGGATAGACTCGGCGCCTACCAAGCTTCAAGCTCCTCGAGAATGCGGCTGATCATGCATCATGAGGCACGAGACTCACGTCGCGAAACTAGGGCCGGGGTGGGTTTTTTCGATGCCACCCTAGAGCCTCTTTCAGACGTGCACCGCTGACTTGCAAAGCCCCTCTCTAGAGAACACCGGCGGTGCACGTCGGCAATTAAGACTACGCCGCCCAAGCCGTCGGCCGCACGTCCCCTGTCTTGCCCCCCCCCCAACGGCGGGCTGTCGATCATGTCCGCCGCCCCCGTCAGCTTCGAATTCGCTTTCCACAGGATGGGATTGGTCCGGGAAGAGAGAGGATGCTGTCCAGCACGCCGTTCTCATGACGGACTGCCGCATTATGTACTGAGGCAGAGCGCCGAAGAGTCCGAGGGCCTTTCCTGGGATATGCCGCATACGTATACGTATGCCGCGGCGGCGAGGGGGAGGAGGGGCGGAGCGGACCAGTGACCAGTGTGCGCATCACTGCTTCTTTTCGCTTCCTTGCTTACGACTATGTATGACGACTTGCGGGTGATACAGACAGCCCCGGCTTCTTTCATTCCGCCCTGTTCATGCCCCGTTCTATATATCTATATCGCGAGCACAGCACACTGGGCGGCGGCGGCGGCGGCCTCGTCTATCGTCCGATTCCAGGTATGTGGGAGACGGAGGCCAGCCATCACGAGCACTCTCACGACAACGTGAACGCGAGCTCGTCCGCGGCTGGCAGAGGGGGCTCTGCGTCTGAAGCTTGTGTATACGGG

>TU5520-GaLu96scf_51-86325-86841 GTGCGTAGGGCCGCGCGATTGGATGGGAGGGGATTGGACGGCACGGGGTTGAGGCAGCGTACGGCGACGTATTGTGCATGAAACGGGATGGTGAATTGGATCCCATTGGATCTCGACGCAGTCCTCGGCGATTGTGCTGGATAAGAGCCGTGCGGGACGAAGACCTCGGAGCAGGGAGAGCGTCTCAGAGGTGGGTTGGAAGCCACTGGAGGTACGTTGTGCTGTGCGAAGCTGTAGAAACACCTGATAGTGCACCCCTCTATTGAAATCAAAATGCGCGCGGCCATATGTGCTCTGTCGAAATGTTAGGATTTCGATTGGATGAGACGTCTACGCATACGGAATCTGGGGTCGATCAAGTAGGTTGAAAACATATGAGCGGGTGCGAATAGGGATTCCGGGCCGAAGAAGCCATGCCAGGCGCACCATACACGGGAAGGTCAAGGGATGCGGTCGTCGTATGCCATGTGGGTCCGGCTGCATCGAACGGTGGAGACGTTCAAGGAGGGATACCATG

>TU5532-GaLu96scf_52-59491-59711 GCGCAGTCTGTCGAAACCAATTTAGGAGGGTTCAATGAAAGTGCGGCTCCCATGACTCTCTCTGACATATCCGAACCCATGCCCACGCGATCCACCCAGCCCCACCTCACTACTTCGAATTTGAAAGCTTTACTGCCGACGCGCTATGACCGGTGAACACCCAGGACGGCGGTGAAGCCTGGGACACAATGCTTTTATATTATGCGACTACGCACATTGCC

>TU5613-GaLu96scf_55-49399-50404 TCGGCTCCTCCCCATCAATTCCTGACCCCCCGGCCCGCCTCCCATCAACCCTAGGCTCTCAGGGTGCCCCCCGCCATCAAATACGAAGCTCCCGAGGCGTACTGGCCCGATTTGGAAGCCTCCCGCCTGCCTGGGGTGGCCCACTTTGTTTCTACTGTTACTCACCCATTTCTAGGCCGATTCCCTTGGTTCTGGTGGCGTTCTATTCGCATTTAGAACGCGAATAGAACGCACATAACGCGCCACACCAAAGGTGCGTACTAACAATTCAGATTGGCCCCCTAAGATCGCCCTAGGGGGGATTGTTTTGTCCATTGTGTGGCCATTTCTCGACGGATCTGCATGGGAGAGGGCACGTTCTGTTCGCATTCACAATGCTCACATAACGCGCCCTTTCGCGCCCGAATCCGTCCAGCGTTCTATCGCCACCCATACCCCCTCCTCCTGGTTGGGTTAGTACACATATCTCCAGAACGAGTTGACCAATTCTGATGCTTCTTTTACTGATGTATAGCCTCCAATTAGGAGATTCAGGGCGCACTATTCGCATGGCATGCGCCCTCTATGCCATAGAATGGCTAGCTGATCAATGTTCAGTCACAGCCGAGTTTGTGTGCCCATATCTCGGCCGGACGACCACCGATTTGCATGGTAGAGGGCTTGTTGGAAAGCGACAAGCCATACCTTTCTAACGAGCCCTCATACACCCAGGTACGTCCGCTGGAAGGCCCGTTATGCCAACCCCATCCTCCTGACCCCTTCTCTCCTCTTCAGGAGCCCATATCTCGGTCAATTTTGTATAGAATTACATGATCTCTTTTCTATTCGCTACCAGTTATATGGCTCTATGACACTCCAAGGTGCAATAGCTATCGCTGTTCGCAATCACTGTTAAATGGCTTATCAGTTGTAGACAGAGGGCCCCCTATGACGATTTTTTGAGCCCTTAGGATGATCTACAGTAGAGTCCAGTAGATAGTATTGAAAAGGACGAAGTGAATGCAAA

>TU5614-GaLu96scf_55-50601-51602 AAAGGTGTATATTGGCGGATTCCAATCGAAGTAACCATCTGTACATATTGCTACTATATACTGCTATATAAGAAGCAGAGTAGGGAGTCTAGTAGAATACTGATAAGCATATTGTGCTTTCAGGCATTGAGCGATAAGGCCCATACGTGGCATACATGGTTGTAGGTACACAAACAACGGACTGCGAAAATTGGAAGTGGAAGTACGGACAATTGCCCAAGATATGGGCTCCTGAAGAGAAGAGAAGGGGTCAGGAGGAGAGGGTTACCATAGCGAGCCTTCCAGCGGACGTACCCAGTCGTATAAGGGCTTGTTAAAAAGGTATGGCCTATGGCTTTCCAACAAGCCCTTTACCATGCCAATCGGTGGACGTCCGGCCCAGATATGGGGGAACAAAGTGGGATGTGGCTGAGTATTGATGTGTTAGCCATTCTATGGCATTGAGGGCGCATGCCATGCGAATGATGCGCCCTGAATCTCCTAATTGGAGGCTATACATCAGTAAAAGAAGCATCAGAATTGGTCAACTCGTTCTGGAGATATCTATACTAAGCCTTCCTCGAGGAGGAGGTATGCATATCTCGGTGGCCACTGGACGGATTCAAGCACAAAAGGGTGCGTTCGATGCGCATTTCGCACGCGAATAGAACGCGTCCTTTGGCATATAAATCCGTCCAGAATCCACTGAGATATTAGCCAAACAGTGACCCCTAGGGCGAACCTAGGGGGCATTTTCAAACTGTTAGCACATACCTTTAGGGTGGCGCATTAGGCGCATTAGATTCGCACTTGAAATGCGAACAGAACGCCGCAACAATCAAGAGAATCGACCTAGAAATGTGTGAGTAATGAGCAAAACAAGGTGGGCCGCCCCCGGGCAGGCGGGAGGCTTCCAAATCAGGCCAGTACGCCTCGGAAGCTTCATATTTGATGGCAGGGGGCAGCCTGAGAACCCAGAACTGATGGAAGGCAGGCCGGGAGGTCGGGAATTGATGGGGAGGA

>TU5624-GaLu96scf_56-20229-21207 CTTCGTCGCGGAGATCATCGGTAAGCCTGCGTGCTCCGAACCTTGAGCAACCCTTCGACGCACTGCGGCCACCTGGCATCTTATCACGTTAACCCTCGTACGATCGTTGTACCCTATCAGGCAAGTAATAACACCGTTTTCACCGCGATGAACGCTTAAATGGATCTTAGGCGGGGCTTCAGGACACCCAAGGAAAGGGGCATGCCTCTGCCGCCGGATGTGTCCATCTGAAATTGACGTTCTGACCGTGGGGTTCTGTTAGTGCGCCCTGATGCTGATGCTGGCAGGCAATTCTTACCAGCTACGCTGGACCGCAGTCGACGGACGTCGCAAACTCCGATGGCCTGCTCCACTTTGCGTGTCCCCGGATGTCTTTGAACGTCACACACATGTGGCATTCCACGGGCAACTGGACACCCAAGTCTCCCATACGCGGAGTGCCACACCGGTGCGTCTCACGCCCTTCACATGTACCGTTGGCTAAACCACTTCTCCGCGCCGGAGCAGCGGCGTCCATCGAACGGAGCCAGTCCCAGTTGAATGAAGCGCTTCGTTTGAATACCAAGCGCTCGGGAGCACGCCTGGCGGGTTTTGCGGGGCCTATTTAGACTTCCATTGAGGCGGAGAATACAACGGAGGTCACAAAGGTGGAACAATGTTGTGTGTGCGACTGTCCAGGCACCTGCTGCACACATCCCAGGTACGCATATGGGCGACGATTCCCAATTCGAGACAAGGTTTGCTCACGGCAGTCACGGTAGGCCTTCAGATGCGTTTCGACGCTCGGATCGTCGTTTGGCGCCGCTCGTTTTGTGCCCCTTCATTCATTGGCATCGGCTTCATGCAAGGCTCGTTTGAGAAAAGGGCTTGCAAGGCAGACTAGACGAAGCAAACCGCTAAAAATGCACCGATGTTTCTGCGATCGCAGGGTTGATCGCGTCCGACCGCACGCGGAAAAACACACAGTGACCCCACCCTG

>TU5649-GaLu96scf_57-41204-41476 CCTTTCCAGCACTGTCCACGGAGCTCGCCACAGTGACGGCGAGAACGAATACGACTAGCAGGGAGCTCGAACTCTAGCCAGGTAATTACCAATGTCTTGTCTTCATGCATTGGGTGCCCGGAGCTCAAGAGGGCGAGGGAATGAATGGACGACGAAGAACTCTGGGTGGGAGGAATGGGCGTGGTTTGAAGGCACGGATGAAGACGTTGTCGGGATCGGAACAAGTAGAGACTGGGGTATCGAGGCGAGGGAATCGAGGACGCCCGGGGGAGG

>TU5687-GaLu96scf_59-3763-4037 AACAGAAGTATATCACCTGAGCATCCATAAAAACCCTCATACTATTCACCATACATACCAGTAAGACGACTAATTTGCGATATATAAGTATGAAGCTCGTCTGAATGACACCAGTGTCAAAACCTGGCCGACGAGATGGGTTCTGGTGGGTGTTAGGCGAAGCGGCGTAACTACCGTACATGGTAGGGTGACATTAGGCGCCCTTGGTGACAAAGCGGAACCTCCGGGGCAGAACCTGGGTTTTGGCTCAGAACCCGGTATCCGAACGGAGCCTA

>TU5706-GaLu96scf_6-30646-31402 ACATGTCCATTTTCGCCATCCCCCGATCCCGCCCCCAACGCGCACCCGACATGTCCATATTTGCCTGTCCCCCGATCCCGCCCCCAACGCGAGGCCATCGGAGTCCCCGACATATATAAGGTGGCCCAACATGGTCGCCCTTTGTCCCCCGCCTTCTTTCCTCCCCCCACCATCTCCCCGCTGTGCAACCTACACCACCCTCTGCCTCCTCCCTCCCTCTTGTTCTCCCGGGTGCGCGCCTCGCGCGCAATACAGGTATGTGAAGATGCACGCCCTATGACGTCACCTAATGGTCATCATCGTTAGGACCGCATGCGACGGGCGGCGCGGAGCTCGATCAGAGGAGCGCGCAGTGGGAGGGAGTGCGCGGATGAATGGGCAGGACGAGCGGGAGGGTGCGCGCGACATATGGGGGTGCGCGCGGCGGGTGTGGTGCGCGCGAGCCCGTACGGTCCCGCGCGCGAGCTGTTGACGAGCCCGCGAGTAGCGTACGCCCGTCTGTGTATGTAGCTATTTTTGGACGCCAATGGAGCTGCCATTCAGTGTACCGAATAAACGCGGTAGAGCGGCGCAATCAAGGCTTCTTTTGAAGATATCGAGCCTTTCCTATCTTTTTGGGGCCAGCGCACAGAGGGGAACAGGCTCATTTCGAAGGAAAAATATTTAAATATATTGCAAAATGTGATAGTGGACAACTGGGGTTTGGAGTCCATATGTACCCATTCGCGTCTTTTCCCCCCAATACGCAGGGAAATCT

>TU5713-GaLu96scf_6-170979-171260 CTTGGCGTTCTTGTCCATTCGTGAAGGGGGATGATCTTCTTATTAGCAGCGAGTGGCACTGTTATCGATGCTGCAGCATCGATGTCCTCTTCGGGAAATGTTGGGTATCGCGCGCTTTGGCAGGTCGCTGGCCGGGATTTTGACTGGCTGCATATTAGATATGGAGAGCCAGGGCAACGTAGCTACGCGATGCTGTTGTCTAGCAAAACTTCTTGGAGTGGCAGAGCACTGGGCGGGTGGACGAAACAGCAGGCGGGACGTGGGCGATCTCGCTGAGAGCAA

>TU572-GaLu96scf_1-4308762-4309313 TTGACAAATGCATCATCCTGACATCCACCATATGAAATAATCTGCGTCCCTGTCCCTCTGACCGAAACACATCATGTCACTAGTCCGATCCATCATCCACGGCTGCGTGACAATAGCACTTTCCGCCATCCATTGTCAAATGAGCGGCTGGGTTTTCGGCAAGGATGCCCGGTGACCCCGCGCGCTTGGCGGCACCTGTGTGTCTATCAAGTAGGCGCATCAGTCATTGATGCGCCGAAGGACAAGTCTCAAGTCGTGACCTTACCGACGCGATTGGTTTCTTTCTGTCCACGACGGCATACTTAGCCTGAGGGTTCGAAGGCTGTTGCTTCGAGGGTAACGATGAGGATGTTCGCCGTCCATGAAGTGCGTGGGCCAACGTGTGGTAGGGCGCATTGGGGACATGGGCGCGCTGTCAGATACGGGATCAGGTCGGCGGGCGATACGGCCGCGCGAACAACTTGGGTGCATCAATGAATGATGCGCCCAAGTATCCGTCCACATGGATAAGGTTGGCGGCCGGAAGGGATTGTCTCGGCGGGTTACGCCACG

>TU5735-GaLu96scf_6-360082-360596 CCTGAACCCTCCGCTTTTTTCACCGTCGGCTGCCCGCAGCACGTTCCTCCATCTTACATGTCGGTCCATTTCTTACCCAGGCTACCTACACGCTCTTCAGACGTCGAGTGGCTAAATGGCAGGACCAAGAGAAATTTCGACAAGATGGAGGTTATAATGCTTGTACTTCAGCTTCCTAGGATACGAGCTTCGCGCCTCCTAACATCCCATCCAATGCATCTGATTTCTGTGACGAACATATGTGGCGCGATGATATTGATGTCTGTAATAGCAACGGTCTATTCTTTTTGATTTGTTCTCAGACGAAGCAGCGCGAACCTGAAGGCCGCCCCGCCTTGAACAGATTATTTATTCACCGTTTTGTGGAGTCATCTGGCAGGAACCAGGCCTGTGGCCCCCCGGGCATCTTCGATGTTCCTTAGTCCGATGACTGATGACTGTGTAGTCAACGTCCGTCCGATACACGGACAATGTCGTGAACGAGGTTGAATTTCGAATGCGGACCACAGCGGGAA

>TU5805-GaLu96scf_6-1129550-1130209 ACCCTCGAGTCCATGATGCGCAAGTTATTCTCCAACCTTGAAATCATGTTATATCACACATATTGACCAAGGTGAGCAGGGTTCGTTGTTCTTTTAATTTATGCTGTAGACTGTGAGGGTTTGCGATGAGGAAGTTAAGCAACTCACCGTATAGCTGGCGGTGGGCTTGAATAGGTTCCTTGAATCGAGGAGCCATTCGAGAACGTCGACTTTGTGGCTGGATGACGGTCGTAATTTGCAATCTGAGCATGCACATATGCAGTCATGGGAGTGGCTTCCTTCACCCATAAATCAGTACTGATATCCATCTTTCTACCTCTCCGTTGTAGGTGCAACTATTAACGCCATGCATCGTACTGGTTGAACGGCGGCATCACTTCACGTCTTAGCCACTGTAACATACAGTATGTTATCCCTGTATTTCTCAAAGGCCTCAGATATCGTGAACGCGGCATACTGGCATACACATCAGACACATTGCTTAGAGCTCGACCCCTGCGACGCCGACGTCCGTGGAGTGGCTTCCGGGTTTCGGTTTGCTGTGTACAACTGTCCTCCCTCCACGCAACAAGGACAGACATCTATCGTCTGCCTTGGGAGACAAGGTTTGCTGAGGCTTCCACTTGGTGCAGTAAGGCCGTGGACGCAATCGAGGCACAG

>TU5813-GaLu96scf_6-1163018-1163760 GCGACTCACATACAGTACTTATTTCAGGGAAGCACTCACCAGTCCCGGGTGACCGACCGCATCTAACGTGTCTCGGGACTTGGAAGACGCTGCCAGTCTTATGTCGGAAGACTATTCCTCCTCGGAAGGCGGGAGCGACCGACGTGGACCCCTTCGTGAGTGGAACCAATGTTGCATCCGCACATACGTGCATGTATGCTCCCCCGGTCATAACCAGGGCAGCAGGAGCATCGTGGGCTCCATTTACAAACCACCCACGGCCACACGAGCGTACATACGGCGCAGGCAACGCGAGCGACGCACATGCGTCCGCACAGCATACTTACGAATCTAGGCTTCCCTGCACGTCGGTCGAAACTCCCTCGTGAGCGAGTTCGCCACAATGCGAGCGATCGCCCCGGACCCAGGGTACAGGATCTAGACGGCGCGATGACCGATTGCACACGTCCAGGCCTCGAAAACGCTATCACTCGATGCCCAGATAGGTAGCAGATAGGTTCGAATTCTACGACGTGCGTGGAAGTGTGGAACCCCGCACCGCGTGTGCGCGTATTGCCCCGACGACTCACGAGGCCTCCATGGCATGGACCCCGACTCTGCGCTGACACCGTGTGTGTTGCTGTCAGCGCACTGGCGGTGGTGGACACTGGACCCGTCTTGAGTCGCCGGCACCTGCGCTCTGCCGCCAGAGTGCATCATGGCCTGACATTTAAACATAGTGGCGGCGCGAAGCCAAGATCTCG

>TU5829-GaLu96scf_6-1336625-1337022 TCCTCCCTACCCGCTGTTCTTTTCACTCCCATTATCCACTCCATCGGCAACACACCGGTCCCAAACTCAGGGCAGACAGACAGACTTACTCTACCTTGCCAATGTGAGTAGTTATACAACTAGCTACAGGGGGGTGCGGGAAGAGACATCCAGTAGGTAGAGCAAGGACAGGTCCGACGACTAGTACTGTACAGTGTAATACTGGCGATCATCCCGCGGATACTCCGTAAGTACGATATGAAATCGGACTGGGAGGGAAGCCCAATGGCCAATGAGTGGGCGCGGCAACGTGCTAGAGATTACTTGCTCCGAATCACCGATACCAGTATGGCCCCTGAGTCGTACTGCACGTATAAATACGGCCCGTACAGTTCCTGCGATACCATGCTCAGCCATAC

>TU5839-GaLu96scf_6-1387892-1388527 TCCTCCATCTTCTTGAAGGGATCCAGTTCGCTACGCATCTCACAGCCCCAAACTGCAAGACGGGACGCTGTTCGCGAGAACCCTAACCTCCGCAAACTCTTCGTTCACCCCGACATCATTCCCCCTCCCCCTCCGAGAGGTACCGGTTGTAGCACAAAGTTGGACATCTTTCGCGGCATGGACAACGAGGTCAACAATGCGGCGCATGGGCGCATGGAAGACATCCGCCACCTCAAGGAGAAGTTCAGAGCCGCCGACGCTGAAAGTGTCGAGCGAATCGCTGTGAGTAGTAACCTGTCATCATTATCTTCCCGCTAACTAATGCACGGCGACAGGCTGCTAAACGGTTTCTCGACCAAAGGATGGCGAAGTTTGAAGCCAGGGAGGCGCAAGCTTTTCCCGTTCCGGAGCCAGGAGAGGTGTGCTCGATCATCCACCTTCCAAATCGTATCCTAGCGCTAACCCATATCATGAGCAGACGACAGCCGCCACGCTCAGGGATCGACTTAGTCTTGACGATTACGCGGATATCAACAACGTCGACGAGACCCTTGAGGAGCAAGTAACAATGGTCCGCATGCGGTTCGAACGGTCCTACGTGGCGACAGCCATTCGAAGGCTCAAGAAGACGGTCGA

>TU583-GaLu96scf_1-4357479-4358260 CTGTGTAAGCAGAGCTACCACAGTCGTCTGAAAGGGGTTATATCCGCTGCGGCACTCTTACGGGGTGCAGTGTCGGGCAACAGCCCCGATGACCATGCATGCGACTGCGGACTCTTGAAAAGGACTGAGGCCCATTTCGAGTCAGCTCGCCGTCACGATCGAGTCTGTCACAAAATGGTCCTCGGTTTTTATATCATCTGCGCTTGAAATTGCTGTCCATCCAAGGGTGTCCTAGATATCCGCGTCGCAAGGACAGTGGGAAGCAAATAAAAATATCCTTGCTGTGGACTATCACCACAAGATACAACGTCTGGACAGTTTTCCCCAATACAGCAGCGCATATAGTGAGTGAATGGCTTACGTTATGGGGTAAAGCCTTTAGTTGACCATGAGATTGGCCAGCACTAATTGAATTTGACGAGATATCCATGCCCACTCGAGGAGGCTCCTCAATCGACCGCGCGGAATGATCATAACATAAAACACACCTATTGCAGTAAACGTAAAGTTGGGATTCTGAGATGTCTCCGTAGGCCGGAGGCAAATTTTCGGAAGATAAATATTGACATGAATATGACTTTTGCTATTGTGTCTTGGTTGGCGTCCGAGTGGAGGTTGACTGGGTTCAAGTTGGTCGTCAAGTCGTAGTACTGAAACAAGCTAGGGAAAATGCCATGCGTAATACTCATGGTATACGGGAGGGTTCTGTGTTTTCAGAATCTCCGCAGTAAGACTAATCGTGGGCCAAGCAGGCAGGGGACGAACAAGCAGAGAAATGCTGG

>TU5842-GaLu96scf_6-1457432-1457882 GTCCGAGTCGAACATACACTCCCGCTTCGCCACGGTTTGTGGCAACTTCGAGTGTGAATCACCTTCACCACATGACTACAGTTATCACTCGAAACTCGAAGCGCCGCCAACGTGCGAGCCTTTCCCGTCCCGCACGAGCGCTCCGTACGGGTATGTCCCTAGACCCTGGCTCGAGTAGATGCACTATTTCCTGACCTCACCTTTTCCGGCAGAGTATTACGGAGTGTTTGGCACCCCGACGCGGGAGTGCGCCCGATTTCTCTACGAGGACATGAAGGCGAGGTTCGGGTGTGACGGATACACCCTCAAAGCCCATCGGAACTGGTTTTATAAACAGGCGTAATAGCCTTGGTTCTATCGGCAGAGAGTAGGGTATGGGCGAACGTAGCAAGTTGGCTCTATGTTGTTGTTGTCTTGAGAAAATGCTCGAATATGTATAAGCGCGCAGTAG

>TU5845-GaLu96scf_6-1461289-1461514 CATTATCCTGGAATGCGCCATATTCACCCTCACGATGGTCTCAATTCGGAAATTTGACTTCAACCTCCATTTAACATGGCAACTCCCTCTTGTTAGAGTTATAATACGCGACGGTCAGTGAGCTCGCGGAGACCCCGTTGTTGCTTTCGTCTGACATTGTCAGCTTGTAGCTATCCTGTATTTTGGCCTGACGCTCTTCAGCTCCCTGTTTAATATCTTCGTATGG

>TU5846-GaLu96scf_6-1461657-1462098 AGCGTCCACTGAACATCCTCGGCACTACATTCACCCTCTGCCTCATGATCGCCGCTGGCCAGCGCCTCGTCCTCGACCTCCGCAAGGTCTCCCCAGACGATACGCTTTCCACGACGCGCGTGGGCCGCGAGGTCGAACGCGCTATAGAGGCTATGGCGCACTCCCGCTCGCCCTCGCCAATCGTTTTCGCCGAGCGTTCCCTTGCACTGCAAGATCCTCAGGTACCTTTAGCACGGTGAAGACGCACAGCTGGGTGGAGCTCGTGGGGGTCTTGGTGTTGAATTCGGCCTCGGACTAGAACACCTGACGTCTGCTCCTGCGGTCTCAGGCGTGAAGGAGGACGGGTACGTGTTGTTGTGTTTTTACCGCCATACAGGTTTCGTGTATGTGGTACTGCGCAGTTATTTCAATGGTACCGGCGTGGCGGCGTTGACGCTGAGGC

>TU5878-GaLu96scf_6-1653433-1653669 CTTCCTGCGCGTGGTTTGCGCTGGAGTGCGCGGAGGAGTACCACGAGCGGCTTGCGCGCGGGCCGTGGGCGGGGGACCGGTTTTGCATCACTGGCTTGGAGGGGATGCCGGCGCCGAAGGATGGAGGGGAGTGGGTGGACGTCACGGCGGAGGCGGACGAGTTGTTGTGCCACCTCTGGGAGATGAACTGCAGCGAGGATGTAGACGAGGACTAGGCTATGCTAGTGATTGACTCCT

>TU5886-GaLu96scf_60-14257-14667 GGCTGACATGGACATCATCCACCTTGGGCTCCTCCCTTTGTCCTCCTGATCTTTCTGTGCATGAAGCATCTACAGTCTGCAAGTCTACACTGTCTCTACTGGCGACCCTGTGCCTTACCTCATTGCGACGCTCTCCACTCCAGCGACCTCGACTGGTGACACCTCACAGGGTTCAGAAGCGGTTGAATTCGGAGGAGAGGTGGCCATTGCTGGGAACACTAGGTAAGTGCCTCCTCGGTGTAATCATGAGCATTGAAACTGGAGAGTACCCCTCTGCTACCTACCACCACCCATTTAGTCTTTCCGTTGTATTGTATAGCTCCATTGTATTCCCTCGGCGGTAACTCGCCCGTCATTTCCTTCAACTAACTGTGCAGGTAACATTCTGTAGTTCACGACTATCTATATAGG

>TU5907-GaLu96scf_62-9485-9901 TATGTTGATGAACAAGCCATCAACTCCGGTGTGGGAAGCTGACCAGGCCGTCCGAAAGGTGCGAGGGTGCTGGGTGCTCGAGAACTGGAAGGACCGTCGGGCGGATAGAAGGAGGTGGGGAGGTAAGGGACCGGGTAAGGACCGGATGGCAGCTCCGCTGCCAGAGGCGACGGTGTGGTGCAGCCGCGTGCTGCAGAGCACAGAATCGAGCTCGAGAATGAGAGGATAGCCGATATATCGCTCGAGGGGTGGTCGTACGGGTACAGGAGTCCATCTACACAGGTCAGGGCCAGCGGCAGAGACCAGGACACCGAAACAAACACGAACACACAACAGCCACAGTGCTACAGCCACAACACTACACTAGGTACCATAGAGTGGAATCCCAGCACAGAACAGGCAACAGCCCCATCAATG

>TU5909-GaLu96scf_62-15813-16168 CCCTATCTCCTATCCTCGGATGCTACATTCCCCCCTCCCAGATCCAACAGATTTTGCCAGCTTAGGCAAGACCTCTTCCATTCGAGATGCTACCCCGCGTATTAGGTCAGTCCATGCTGCATCCGGTCTCAGGTGAGTCCCATTAGGGTACTTCGGAATTTTAGGTGCTTTCATTTTCTATTAAGTTCACGAATTCTCGCTGCGGACCAAAGGTACGCCCAATTCCTTTTCTCTTATGCATGCGACAACGGGGACGATGTGGAAGCGCAGCCGAAAGCGAGTTGGGAGGCCAAGGTATTCAAGTTTGGAGGGCGTGGCGGCGATGTTCTCTGGTGGAAGGCATGGCGTGGGTTCGG

>TU5947-GaLu96scf_68-455-804 GAGGTGTCTCCCGTCGGAGAGGTTGTCCAGTTGGGTCCATTGGAGTGCCGAACCGGAATTTTGTTCCTGTTGACTAGTTCCTAGTGATTCTAAGGGTGTCGCCGTAGAGTGTCGGTAGGGTCTCTTCGAAGTAGGTGAGTCTGCGAGTGTCGCCTAGTGCATCGAGGGTGGGGTCAGTTGAGTTCGGCGAGTCCACTGGAGTGTCGCTGACAAACGATGAGCCCAAAAGTTGTTGCCTAGATACTTAGATAGAGGTGAGTCAAAAAAGATGTAAAAGCACGTAAGAGATCCTCCGAAGGCCCGCAGGTGTGACTCGTCCAAGATTCTGGTATTCAGGTGACAAGTAACAT

>TU5955-GaLu96scf_69-7673-7928 TAATACAGTCTTTAACCGGTCCTCGGTTCGCCATGGGTCCTCGGTTCGTTGCGAATTTTGTCCCTTGCGCTCCTTCCTGTCGGCCTCAATTTTATTACATTGTCATGGTGAACTGTACGAGCTCTTCAGATATCGAATTTATAGGGCGCACTCAAAATATTGCGCTCTCCAGTTCGTGGCCTTTGGGTAGATTACAGAGAAACTGAAATTTCGCCGCAATCCCCGCTGAATATGTCTGGGACACTATGATAGAATC

>TU595-GaLu96scf_1-4526379-4526638 CGAACATGGGTATCGCGCGGCGCGCCCTGTCCATCACGCGAATAACATGACGGCGGGTTTTGGAGAGAAGGCTAGACAGTGGTGCACCGGGCCGGGTTGCCTCCACCTATGCCAGTACTGAGATCTTGAGAGGGGATGCGCTCCTAATCGTATACATGGTTTGGTTAGACTTAGTAAGTTAGACGTGCGCGACGGGGTTTCGGGCTCGGGCTCGGGCTCGTCACCGTCGTGAGCCCATATTAATCCCCACCCGCGCAACG

>TU5968-GaLu96scf_7-47997-48581 TAAAATTACAGTACAGTAATGTTTCGAAGCCTCTCGAGAGATGGCGACGCCCCGAGACTATGGAACGCTCGCGTGGACCGTCTGCGCTAACTTGGCGACGGGTGTGTCAGACTCTGCGTGAGATTGGGTTCCAACAGTCGATCCTCCAGGCGCACCGGCGAGCGGGCACTTCCCCGGGCTGAGCTTGGACGAGCAGCAAGGCGGGGCGTGTGGGCGAAGGGGTGGTGGGGCACTGGGGGGACAATGAAGGGTGAGAGAGGGTGGAGGAATGGGAGCATAGGAGGCGGGTATGGATGAATTGGAGCGGGGATGATTGGCCGGAGTGAGAGTGACAGAGGCGTGATGGAGGACTGAGGCGTCGGCGTCGAGTGCCGGACGGTTGGGTGCCGGCAGGCGGAGGATGGCCGGGACCACTGGAGTCTGGCAGTGACTCGGAGTCGCTCGAGGGACTAGTAAGTAAGGAGTACTTAGTACTGGGACTCTGGGAGGCTAGATGGTAGACGCTGGATTGGCGTAGGGCGAGTGCCACGTTCCGCAAAATCCGAGAGGCAGGAAGGACGAGGACGGGCCCGGGCAGGAACGCCC

>TU5997-GaLu96scf_7-155797-156385 GTCTTCTAGCAGTTGTAGCATTCCTCCTTGAGCATCCAGGAGAGAACGAGGATGAGGAAGACAAGGAGGACCCAGAGAGGGCACATGCGAGAGATGTGGAGGACCAAAGGAAAAGGCGAGGGCAGCTAAGTCGTGCACTTTCACGTGACAAACACATCCAATAGCACGGATGATCATGTCACCCAATTTGACGTGCCTATAAATGGACCTCCTGGGAAATGAATGTAAAGTCCCCAGGTTTCCCTTGTACCACCGTCTCCCAGCATCTCCCCTCAACCTTCCTCCACCCTCTGCAGCACATACTTGCCCTCTATGCCGGGGACCCGAACTGAAGTAAGAGGATTCGGACAACACCTCTAGAACCAGGTGCAAAAGTCCCTATGAGAACCACACCCTGTTAGTATATCTTGGTGCACATATATCCATATCTATGTGTCACAGCCGATAGACTGTGCCACCAAACAAACAAACAAGGGGCTCACTGATGGAGGACACAAGTGGTGACAACAGAAAACAGTGGATAGATTGTCTAGACTAGAAGACGCAGTGGGGCAGGGGCTGTTAAGCTGCCTACAGTGGAAGGGATGGA

>TU6008-GaLu96scf_7-180475-181133 ATATTCAAGTTGCAGTACCTAGTATATACGAATCTACATGGACTTCATGCTATCTACACGCTACACGGCAGTTGCCAGCGCCAGCACGCCTCCAGACACCATACCCGCCCAGATGACCAGTAAATGCCATCCTAAATTATAGCTATAGATAAAGCAATATCAGTCAGGACTCACCTTCACCTGTACCTGGGCGCATCAACTGTCTAGTGTTCAGTCCGGTCTATGCATGCACGACACCGCAGACACTCACCTGTGCAGAAAACCGGGCTGAGGAGAAGAAAACTCACCTTGCACACAAACGGGACACATATCCATGGCAGAGGGCATGCCACTTGCCGATAGAGGCTCGGAGGTGAGTGGTGTCCACGCAACGGACTCCCACAGAGGCCCCGAGCAACGTGGATGGACACTCACCGGATATGTCATTCCAAACAATGCCAAATGCCGCACGTACCGAGGTTCAGAGGCTAATGGACACACCTCGCTCTTGAGCTCGAGTCCTACGCCGTTGCAGCCCGGATTCCTGTTGGTGCCGTAGCAGTTGCGAGGTCGTATGGGAGGCGGGAAAACCAGATGTCTTGCAGAGATAAGCGTAGAGACAACCGAGGCTAGTGAGACAACGAGCAGCGCAGAAAGAAGAGAGCGATGTCATCCCGGAC

>TU6023-GaLu96scf_7-235080-235938 CCTTTGGGAGACTTTTGAACGACTTTTGTCTGGTTTAGCCACGTGGAGAGAAAAGTCTTTCACCTGCCTCTCCTTTTCAGCGACTTTTCTCCAGTCGAAAATAACCAAATGCCCGCCGTGCCAGCTGGACGGTCAACCGGTAACGGGCCACGGAGAGTTCATGTTGCCGAATGTCGGCGGACTAGCGACGGCGAAAATCATTGCGATGACGATGTCTGTCAGCGGTGACCTGGTGGTGACAAGACGACCAGCGTCCAAAGTCGAGGTGGACGTCGACCAAACGTCGACTGGTGCTGGCCAGCCAACGACGGCGAACTAAGTCAGGACCAGTCAGTGGGACTCCACTTTGAGCTCACGTAAACCCCCTGTGAACCCCCCTCGATTTCGGGGAGGAATGAGGAGGCGAGACCCTACGCGCGGAGTATACATATGCATCGCAAACGCGAACGACGAATTCAAGACGGATGGACGGGCGGTGGGCGAGAGACGTAAGCAGACGACGCGAGTAGGAGGGTGGCGACTTGGGGGAGTCTGAGAGTGGATAGATACGTACATAATGATATACAGAAGCTGGACGAGTGGACGTCAGGGAAACTGAGTACACGGCCAAAAGCCAGTTTGAGTGAATTTCAAGTTTGATGGCAAGACGAGGGCCCTTAAGAGCCAAAAACCTGTAACTCACCTAAAATCCAATTATCAGAAATTCTGATGACAGGGAGCGCCCATCCGGACGTCCGCTAACCAGCAGCCGAGATGCTAACAATCCGGCGCCGATGGACTTGAAGTGGACTTTAAAACTCGGGCCCAGCCCAAAGGGCGGTCGGGAGGGTTGGACCGGAGGTCCGCCCGACACGAGAAT

>TU6028-GaLu96scf_7-285481-285810 CGTGGTTTGGGGTCCTTGGCTATCCCATCCGCGCATCGGAATTCATATTGACGGTTCCATTGAACATGATATGATTTGCATGCACTCACATTCTCTAAGTGGGCGAAATTAATGATCCGCGAGAATCGATGCGGAGGTATCGCATAAGGTCTTTATGAATGGGGGGAGCTCAAATTACGCGACAGGATTTCAGGTTCCCTATGTCTGTCATGTTCTTGGAGTTTCCTGAAGCCATACCTCGATGATAATCTGTGATTATGGTTCGTAATCAGTCGATGGGTACTTATTGTTATGTGTGGATTGTGCTCACATACCTGTGAGGTCTATGAT

>TU6059-GaLu96scf_7-529231-530103 TCTGGAGTGTTTCGAAGACTGATGTTCGTCGCCCATTACGTCTACGGCGTGCGGAGTTGGTGGGCTTTCTTTATTCTGAAGAGCTTCCGAGCATTTGTGCCGCTCGGGAGCAGGGCGATCGACGGGTCGGAGTGGCTGTCGCCCCTGTGACTGTCGTCCTAAGGCGCGCCTGCGAGCGTGGCCGGCACTTATCACCTTCATCTCCACTCTTCGTTGCCAACCGTCTTTCGGTTTCAGCTTCACCCCGTCTTGCCGAATCTTATCGATCTCATTCCGGATCACCAGCACCCAACCTGTGACATTCCGCCGCCTTGTCCGTCTTCAACGTGTTCCCCGTTGGAAACTGCACGCTCTCCTTTGATGTGGCCATACCCCCGTTCCCTATAAAGCCTTCCCTCTTCTCCCCTCTCTACCCTAAGCTCTCAGCGCTCTTGTATAATTTGCCTCGAGCTCCTTCTCCCCTCCTCTAATCCTCCCTCGTAACCAACCACTACGTCCCCTAGCCTCCCCACCACACCGTCCCCAGCCCCCAGCTCGCCGTTCTCTACAGTTCCCGCACTCGTTCCGCACTCTCGCCGCAACCTTCTTGTCTGTGCTTCCCGCTCCCGTAAGCGCATCTTCCTCCGTGTCCCCGGCTTCTCCGATCGCTTATGAGGTCTTTAAGTTTTAAGGTTCCACACTGCTGTGCTAGCCAACATGTGCTGCCTCGAAACGTAAGTCCGGTCCCTCGCCCCGTTGGCCTTTTTGTCTCTTCAATATTCGCTCACAACGGTCTGATCTGCAGCGAAGGAATCAAATATGGTTGCGGCGTACGTAGTCTGAATATTTGTTGCGATTCCGGCGCTTCACGTCCAGCTGACGTCACCGCCACTG

>TU6066-GaLu96scf_7-556468-557270 CACATATGGCGTCCAAAAGCGAGTTGGGGGTTTTTTCTTTTGAAATGTTTTCAGACTGTTTAGACTCGAAATAGATGCATTGCTAGCGCATCGCGCGGCACAAAAGGATACAATTGACTGGAACGCGACCTAGAAGTCTCCTGGTAGTAATAACCGACCCATTCCGCGCACCCACCCACTACCCGCCCGCTCGCGCGCGACCGCAGATCTCGCCCTGCGCCTTGCGATAGCACCCGCGCGCGCCGCACCCAACCCTATCGCGCCGCACTGCATATCGTACTTACCCGCGCACCATGCCCCTTTACCACCTCACCCACCCCACCACGACGCGCGCGCCTCATACCCGCCGCGCACCAGTTTCACGCACCTGTCATGTCCCGCTGCCCTCTGAGCGCCTCCTTCCTTCCTCCGAGTCACCCGCTCGCTCGCCGACTCGCGGGTAGCGCTTGCCGCTACATATGTTCAGCGTCACGGTGGATCTGACGGGACACTGTCTCTGTTAGCTAGTAACTGCGCGTCTCCTCACGCTCGGCGGAGACGTACCTATATGCGCCCCGAGTGCGCATATATGGCGCATATATCCTCCCTTTTCCGTTGGTTGACGTAGGTGAGACTCTGGGGGCGGGAAAGGGCGATCTCGGAAGGTGCCAGGAAGGTGATGGCAGGAGGCAAGAAGCGGGGGAAAGCGGGAGATGGCGCTGCCCTTTTAAGAGAACGTCGGGACCTTCGATGGCGTCATCGACGGTGGGACGGGGTCGGGATCGGAACACGGTCGAAAATGGATGTGGTGGGTCGGGCCCCGA

>TU6085-GaLu96scf_7-652621-653092 ATTATACTTCCCGAACTCCGAGAGCTACAGCATGGTAGGTGAGCGCGGAGCAATGGGAATGGAGCTATTCGAGTGGCGGGTAAGGAAGTGGGTGTGAAGTAGAAAGGCGTAAGGGGGTCAAAGGTGCAATGGTCGGCTTTTGTACACGGGAGAACGCGTCAGCACCGCGCAGGCGAGGAACAATAAGAATACTCACAATTGGCTTTCCCCTTCAGTACCACCTGACTCCAAGCACGCGTTTTAGCTGAGGACACGCATGAAATGACCTGAGCCTCTGATCTTCAACGAATGGCGTCGGAGGCGGCGGCGAGCTGGCGTGCACGGTCGCAGTGAGTTGGACCGATAACGGGGACAGTCCAAGCCCTAGCTTCTAGCTCATTCAGTGAAGTAGGCCAGCCAAGAGAGCGTCCCCGGCGCTGCGAAATCGGAAAGGAATGGGTAAGGTTGTAATGCAACAGACAGATTGCAATAG

>TU611-GaLu96scf_1-4577269-4577767 CCTGCGTCCAAGTACTTAGTAGCTTGCATTCAAGTTGTAGCAACTACTTAACGCAGGACAAGCGGGGGACTGAGGCCTGAGGTGCTGGAACTCGAGGTAGCCACGCAGCCCGGCATGTCTCGTCAAGTTGTTGCCACGCCGCACGGTGAGGAAGTCTTCTTCCCCGGTCAGGCCACGGTGAGCGGGGTCTAGCACAGAGACTCTCCAGAATCGCTCGTACTCACGCATTGACAGGCGATTCGGAATACCTGCCGGGTGGCGACGTCAATACTACCAGCTCCGAGCCGACTGCAGTCACTTGCAAGAGGCAAGACAGGGTTCATTACGCCTTGACCCTCTTCTCCTCTACCGCTTCGGTATAGGTCGCAGTGAGTCGTGCAATGGAGCCATTGTTCGTGCGTCACGCCGGGCCACGTGCCTTCTCATGACAGGCATTTGCTGCGCACACCTGAAACGAATATTAAGACCGAAATTGTTCTTGGTCCGCTCATAACCTGCC

>TU6238-GaLu96scf_7-1408983-1409928 CATGAAAAAGCTCAGTTACAGTTGACATTTTGTGGACTCGGAAGGCCGCGGCAAGGACCGGCGCATAGCGAACCGCATATATCAAACCATATGCAGCTGGGCGGGGCGGGGCGGGGCGGGGCGGGGCGGGGTCAATCAGCGGTGAGCCTGTCTCTGTCCCGCGTAATCTTGCGCATGGATAAGGAGCTCAACCCTCTCCGGTTTCTATCAGCCGTCGTTAGAAGTAACCGTGTAGCGTCCTCACCCATTCAACAACGGCCTAACAAGTAAAGAAGTGAAGGCTAGTGAAGGCCGCGAATTCCCCAAATGAAGCCGACGCTCGTACCTCACACTGCAGTGTCAGACATCAGGCACTGACCTCGGTGATGGTAGGGGAGGGTAGGGGCCTAGGCTAGAGAACGCACACCGGACGATCGATGACGTTCACGAATGGTCACGCCGCGTATGAACTACGGGGTGGCGCAGTGTCGTAACAGCGCTGTCCCCCTTGAATGCGCGTCGCTCACTGGGAACGCTGTACACGCAGGTGAGTGTCGCAAGACGTTATCCGCGATTTTCTGCTTACCTTGTATGTCGTCATCGAAAATGAGCATCCCTCCACCCGTATGCACACTTCCTTTGGGGAAACCGCGATGCGCCCCATGGCAAAGACTGTGTAGATATCCGCTGTTCTCACCTTTATAGCGCGACGTGTGTCAGCCTTATCTTCCGAATAAACTCACCGCATCTCACGCTTACTATGATAGGGTCCGATGACAAGGTACGTCGAACGGCGATCTTTTCTATCCGGCTGACGCACGGATTGCGAAAGACAACAGATACGATGACCATCCGGAGGATGTAGTAGGGCTGACGAAGGAGTCAGGTCCGGTAAGTGCAAGCGAGGAATTCCTTCTAGCGTGATTCTTTCCTTGGACCGTATATTAACCTTTGCCTTTGCCTCGCC

>TU626-GaLu96scf_1-4774118-4774504 AGAGAATCCATGAGGCGTCGTTTGCAAAACGATTTGCGAGCTTTTCGGGGGGAAAGGATACGAAAGAAGGAACAGCAATAGGCCTTATAACTTGTTGTTCCGGGGGATTAGGACACGTAAGTTTATCTTCTCCATCCAGCTTCCTTAAGCTTCGCATTGAACCTTCTGTGTACAGGTCGATGGGTCGGGATTCGAGCTCGGAGAAGCATGTCGGATCCGAGGAGCGCCAGGCGCAGTGGGCCAGACGCGTGCCAGACGCTGCCGATAATATTCGGCGCAGAGTGGCAGGGCATACTAGTATAAGAGCGTTCAACAGCTGCGGAATCCACTCCTCATCAGATGGTCGCACATTTCCCGCATTCGCTCCACGAGCCGGGGGGGGGGGAT

>TU6279-GaLu96scf_7-1533368-1533628 ATCCAGACGACCCATGTCGCATCCAGTGTGCAGATACTTACTCATAATCGTGCCTGCAGCAAAGTAAAGATGAGCACAAGAGCGGCGAGGTGCTCACCGGGACAGCTGGCCATGCCACATCATCGCGCGACCGACCTGCGTATGCGATACTAGCCAAGCTGGGCCCCATACGCGCTTGATCCTGACAACCAGGTAGAAAGACGGACTGCTTAGCTGAGAGGGAGAGCGACGAATGATGTCGCGACACTGAGGCAGGCAGGA

>TU6307-GaLu96scf_7-1652731-1652980 GAAGAGCTGCAGGACAGGGGCGCGACGACCTGCATCTTCACTCGCTACCGGACGGTGATGCGCGGACGACGTAGTGCTTAGTCACCCGGCTGCTCGCCCAATGGCCTCCGCTGAGCTACGCCAGCACCAATCGGGGAGCGAGTGACAGCAGGTGGCGGCGCAGGTCGTGCGCCGAATGAGGGCGCCTTCGCGTGGCAGAGGCCTCGCTCACTGCTCCCTACCCTCGGAATCCGCAGTGAAGCTGACCTCT

>TU630-GaLu96scf_1-4781222-4781427 AGGCAGGATATCGAGTCAACGCTCTGGACCTGTGGTACGAAATAAACCGGCGTTAACCCCGCTAATGTGCGAGTCGTCTTTGTGGCAACTCGTTAGGACTCCGAGTGCACGAGGATCCGCAGTCACCGGCTAGCCACCCCACAACTCAAGCGGCTCCTTGGTGGGCGTACTACACGTGCTGGTTGTATCCGAGTCTCTTGACGGCC

>TU6360-GaLu96scf_8-133352-134088 CCTGCGCACAGGAAGTGGGCACACGACAGGACGCGTACGATGATTCGGATGCACACTGAGAAACGTCCGAACAACGTCGCTGTTGTCTACGTTTTTCGCTAGGTGAGATATGGGTATGTCAGCCGTGCTTCCCTTGCAAGTCAACCCTTTGGCAGTCGAGATGCACCGTCCAGCTCCGCCGGTGTTGACAGGCCCATGTTCCCCGGCTGCTTCCCAGGTCGAGTCACTTTTGCCCCGCCTTGGCTGGGTTTGACGCTACTGTTCCCTGCAACACGGGGCTTACCAGGACGCATTCGAGAACGAGAAGCCGCGGCGCAGCGTCAGCAGACAATGGAACCGGTGAGTTCGTGAAACTTCGAGCAGTCTCCATTCTCACCAGCTTAAACCTGTGGGACGAAATTACGATCATCTTTAGCTATGGCATAGCGTCACCAGAAGCCTATGACGCGGACAGTCCAGGCCTGATGTTTACTGGGGCGGGGGTAGAACGGGTATGGTGTCGACCTCAACGTAAGTGATGCGTTCTTCTCGTACTCACGCTAGGACGACTGTTTGCAAATCCTCACGGGAGATGTGTCCGGGAATCCGAGCGAAATGTCCAGACTGTCGAACGACCGGAGGCGCAAGAAGCCACGAGGAAACTATGGAGGACCAGTCGGGTGCGTTCGGGGCTGTAGGTGGATATAAGGTTTGCGAGACTGAATGCACGAAGCCGTTTGCAAGGGGCATGTGAGTAG

>TU6375-GaLu96scf_8-221655-222191 CAGGAATTCATACTAAACAACGCCAAGCACGCTGTCTTCTCCTGCTGATGACTACACCAGCATACAGCCGTAGATCTACACAACATTTGGCAGCCGACCAAGGCTCCCACTACCACGACGAGGTGGCTCCGTGATCAGCTACTTTCCGTGTCTCCGTCACCTTGCCGTCGGAAGACGTGTAGCGGAAGTTCTGGTAGTAGTAGCTACCATCCCTGCGAGCGTGGTCAAATGGAGGGAAAATAGACAAGATGGACCGACATGCTTGTTGCTGTAGTGGTATCCGTGGTTGCCATTCTCGGATACGCGATGGTCCTATTTGTTCCCCTGGTGTGCAAACAAACAAACGCGTAAAGAGCTGAGCGTGGGGCACACTGAAGAGGTCCTGAGACGTCTCAGGCGCACCCATTGATTCGTCCCGCAGGACGTGATATCGTACTGTTTGCCGGTGTACCCGTCAGTAGGCATCAACTTTGAAGAAAGAGTTGAACAAAGTGAAGCGGTGGAAGGCTTAGGACCCGACAGACACTACTGAGGCCG

>TU639-GaLu96scf_1-4803896-4804176 GCGTGGCGGACATTCAAGTGGCGCTGAGGAAGATGGGCGGGTTCGGGTGGAAGGGGAAGTTGGTGGTTGGATGGGCGACGGGGAGAGATGTGTTGGATGCGCTGGAGGTTGCGGATAGGTGGGGTGGGCGGAGGGTTATCACTGCGGTGAAGGGGCGACATGAGCCGTTTAATCGGCTCGTTTCCGAGTCGATGGGTGCGCAGAAGTGGGAGTGTCTTTGAGGAGACTAACGCGTGACGTCGCAACCATTGTTCTATATCTACAGAGACAATTCTTGTCCT

>TU6502-GaLu96scf_8-1215228-1215663 TCCTAGACCTACGTAAACTAAGTCCACCATCCTTCGTTATCTTCTGTGGTGAGGGTAGTTAGGAGCAAACTCGGTGAGCGTCCTCGCACTTCTCATATACCCAGCGCTGATGGGGCTCCGCGACGAAGTTGGGCTCGCAGAATCATACGTATGTTCGACCAATATAGAAATTATCTTCCATTTGTGTGTAATTGCCCGCACTTACTTACTCCTTTCATACGTGCAGCATACACGACATTAGGTGTCCTCGTCAAATTATGCCGCCACAGTCGTGGTGTCGCGATATGGCCTCATTCTCCAAACTTCTAGTTTTCCTCCAAGTAATGCTAACAACCCAGGTATCATAGTAGATCCGCGACATCTCGTACTATAACACACTTTAGCAGGAAGCAGCCTCACACTACCCTACTTCTTGCGTAATGAGGTTCGTCTGTCA

>TU6508-GaLu96scf_8-1236085-1236507 GTCGAAAAAAAAAACGACGTGTTCGAATCTTCCGAGCAACCTTCCGACCCAATCTTCCAAGCCTAGACGCTCGCCGGTCCTGCCCCAACCGGCGTGGCATGTTTGGCCACCTGCCTAGAAAGGCAGGGAATGACCCAAATTGGGGCTGGGTGACGTCGGGGAAACTTCCGGCACACCATTTTGGGCAGGACCAGCCAGCGCCTAAGGGCTGGAAGATTGGTGCGGAAGGTTGGTCGGAAGATTCAAACGCGTCGAAATTTTTGGAGTCGCGTCAACCATTTGTGAATCTGCTGTAATATTTGGTGCACGGCCGCCGTGGTAGGGTGGGTATGGGGTGGATCCGGCGACAGGCGGTGGGACATGGATAGAACTCGGCTTTACATCGCGCGTGGCTCGAGCAGCGGGGGCTTGTCGGCGATAGTG

>TU6512-GaLu96scf_8-1246120-1247036 TCTCTATCTCTCCTCTTTACGGTCACGTCGAGAGCTGGCAAGTGCGTCTGGACCATGCGTCGCGGTTCGCCTCCTCATCCCCCTCCGTCAGTCCAGCACGTAACTACCACCTCGAGGCGCACACGACCACCCCACACCGCAAAGGCCGCGAGGCTCACCCCGAACAGGATGGCGGCTTCGGCTTCGTCTCCGAGGGCGTATGCGCATCCGTGCTCGTGAGCGGGCCAAGGTCTGAGTGTTCCAGTCCCCTATGACTGACTCTAGCCGAGTTGGGTCCGGGACGACCCCGCGACGCACCAGACATCCGCCGCTGACACCCCTCGCCACCGAGCGCCGTGACCGGAGCCCAAAGACTCTGACCGTGTGCGGTTAGCTGTCTCAGGACCGTCATGCGGCCACGATGAGGGCTCGGGGTTCGGCAGCTTGAAAGCGTCGTCGGGACCGGGCGCGCTGCCTTCAACCTCGCTCTGCTTCGGGACTATCGCGTCTATCGCGTCGGTGGCCTTCGTGGCACGGCACGCATGGCGTTGTTCGTTAGTTCGCTAGTCCTTAGACGACGAGGTGTACGAATGCGGAGACAGCTCTTTTGGCAGACGTCCTTGAGTGCACTTCGCGGCTCGCTGACGGCCCTTTTAGTGCCCTGCGAAACTCGATTCAGACTACTCTTGCGCTGTGGTTCTTCCACGCCAATTTTAATTTTTCGCCTTGTTGCTCTTCGTGAGCCCATTTATCTCTTCGTCAAAGATTGTCCAAGTCTAGTCAAACTTCCACCTGATCAATTATTGACAGTGTTGTGCATGTATGCGTAAATGCAATACATAGTAGATACTTCTTGCAAGCGTGGACCACAGCGTCTCAAACTCTGAGCAGGATGGTTTGCGAATCAGCTACGCACGGTAGGAGGCCTCGGGCGAGCT

>TU6538-GaLu96scf_8-1472173-1472788 TCCATCGTCGTCGCCCCGCCGGTACACAGTCTTCCGGGCTGCGCGAGAGTACCAATGACTGTGGGTGGCCAGCTCCGTCGGGCACGAGATTCTCATGCACACTCGAGTCCAGAGGAACATTTTTGTGCCAAACCTAGCGCGTAGCGCAAAGGACGGGATTTAAGTTGTTGAAGCGGCCCGCGGGTGTTCTCGGAAAAGTCTCTGCCAGTGTATATCCTAGCCTACGCCGCTCGCCAGGATTCCCCCGCGGAATGGGGGAATGGGTGAATCCCTGTTGTACGGACGTTCTCATGAACTTCGTTTTCGTCCCACTGCTTATGTGCTCAAAAATATCTAATCTATTTCTATTACTTATGTAGGGTCAAGCAAGACGGTTTCCGCTGGCGCAGCCAGCCCTTCCGAGTACAAACTCCACTGTCAATACCTCTGAATCGTGTTCGTATATGCACTCGTTGACCTAGCAACTTGCCAGCAGCTCACCTGAGGCACAATTCCTTCCCCCATCTTTCCATTTTATGGATGATCTGTGCCCGGCGGCGGGCGCAGCAACTGGTTGAAAACATGGCCCGAGCCTTCCAACAAGAGGTCGTCATGACATTGACATGGATGAATTACG

>TU6575-GaLu96scf_9-74236-75250 ATCAGTCATGCCGCCCATAACGTAGTCTCAGCACACGCCGGTCGAAGACGTCACTAGTGATCTCTGGATCCAATGTGTTGGCAGGCTATCTATCCCCCTACAAGTATCACCTGAAGACCCCTTCGACTCGTCCTCTGGCGCCTGACCGGTGACCATCGAAGCCGCTCGTCGAGGTCGGCCGGTCAAAGAGCCTGCTAAGCCCACCGACGTCCCCTGATAACTATCATCGCTGCCAATTTCGAGCTTCATTTCCACATCGCCGTCCTCTTGCTCCGAATCCGACTCGATCTCTATGGGTGTGCGCTTCGAACCCGTCCCTGTCCCCTAAACCCTTCTTATTGGGTTGGCCGCGCACTGCTTTCGGCGTTTGGGCTGCGAGTCCGAGTTGTTGCTCCCGCCTTTCCTTTTCAAGCGCCTCGACACTTTCACTCGGGAAAGTGCTCTGTCGCCGTGAGGATGGCGCTGGGCCTGTGGCTCAGGCTGGGGCTGAGGTGAAGGTTGAAGCGAAGGTCGGCGGGGCGAAAGTCGAGGGGAAAGGTGGGGCGAAGGAGCTTGGGGCGAGAGCTGAGGCGGACTCGGAGGGGCTTGGTCTTCTTGGGGCAAGTCCTCCTGCTCGATGTGAAAGTCCTATGCGCACTAGCGATGCACACTCACTTTTGGCAGAGTCGTCTGAAGGTTCCTCCTTCTTGAATTAAATGACAGATGCGGGAAGGATCCGTGTCGTGATTGGTTCCAGATCGAGTCCCAAGGGGATCTTCATCCCCGAAGCTTTGAGCTCATCCTGTTTTCCATCGTTGGTCTCGTCAGTGCAAATACATAAAGCCACATCGCTAGCACACCTTATCATCGACTTCCTGATCCACCCCCGCCCTACCCCCCGCAGAAGACTTTCCCGACTCGGACTTTGCCGCGCGTGCGGACATGCCAGAACTTGAAGGCGTCGTAGGACCTCCTGACTGTAGGAGACGGCGATTATCGATGGTAAGTAGTTGAGGTCGGATGCTGGCAGTCTAAT

>TU6592-GaLu96scf_9-174185-174460 TCAAACGATACATGGCGCCATTTCCAAACGGAGGGAGCGCATATAGCTACTCCCCACGACATTCAACGTCGACGGCTTATCGCGAACGGGTCTTCCCCCTCACCTCCTCCTCCTACTCCCCCTCCCCTCCTACTCCCAACATCCGGCAACCCTCGCCTCTATATGCGCATATGTTGAGTGCTGGTGATTGACCGAAAGATGTAGAACGAATGAGATACGTTTCAAAATGCTGCCGGTGAAGTCGCCAGTGAGGACCAGCAAGTCTCGTAGCGCATC

>TU6603-GaLu96scf_9-240286-241242 TCCTGGTGCCATCCTCATTCTATCTGACGGGTATTGGCCTGTGATGCTCTAGACGACGGATGTCAATGATGGTCTGTTGTACTGTGTCTTGACTCGTTGTTTCTCGGGCACAAGCTTCGCCTTTGAGCAACGTGGCAAAAATGGCTCTCCGTGTCCACCACCCATAGCATGTCCGACCTCTTCCCCCAGAATGTCTCTATACTTCTGACGGCCATAGTAACAGTTACTGAAACTTCAATCCCTTCCCGTTGTCCCCCATGTCTCATCCAAGGAATAGCGATCTCACCTCGGCCGACACCTAATCATGCTTCCGCCACACTTGCCCTCGGGTCCGTTTACAGCAGGACGCGGAGCATAGCAACGCTGAGAGCGCGTGGGCTGTACAGACGCATAGACAAGGATTCCTACGGTATGCTGTTCATCTCCTCCTTCCGAAGCTGTATCCAACTCCGATACTGAATGCTAGCATTCTTAGATGATCATCGTGGCTGGCAGGTCGCGAGTGGCGCCGGTCCGCTGCAGGTGGCTTCGTTTCCAGCCTGATCGCACCCGCAGTGGCGAGTCTAACTGCGGGTGTACTAGTATTCCTCAAACCGAAACGCTTTTTTTCGCCCAAGCAGATCCTGCTGGAATGGCGACGGGAGGGATCGCCAAGGTAGTGGTATGTTCCTTGAATCGGCGTTCCTCGTTGCAGCGGACCGGAGTGCCCTCAAGATCTTGCAAATAGACATGTCGGCTGTGGCTGACGACGACGGCAACGTGTCGGGCCAGATTGTGACTGGGTTCCCTGAGCTCTCGCCTCGCTGGACCCGACAACGGTGGTGGCTTTGTGATGGTTTCGTGAACCGAGTTTCCAATGGATTCTGTCTCTGATATCTGCATCTAATACCCTCCACCCCTTGCCTGTAGATCTTCCGAAGGCGTGTTATGGATGCCCCTCTGTTGTGGATGTATTAC

>TU6607-GaLu96scf_9-256015-256492 CGACGGAGACAATTGATTTACTGTTCGAACAGAGTCTGGAGATGGGGGAAAGAGTGAGCGGAAGCTGGGGCCCAAGTGGCGAACCCAACGGGTATCTAAGAGTGTGGTCACGGCAATAACTAAAAGGGGAAGGACCGAGACATTGAATCAGTGTGGCGCGTTACAGTGCGACGGGCGCCGGTCACAAGGCCTACGGTACCTATTTATCGGAATGTGGCTAAGGGAGAGAGAGAGAAGGCGAAAGGGAGTGCACGATGGTGACGGAACGAGAGACGGGAGGTATGAAACGATAGTACGTAAGGGTATCTGCGTTCGTTACGCTACGAGTGTCGTACTCGACACTACCACAACGTCCCCTATGAAAGACGCTCCCACAGGGATCGAGATGAGCCATTACGATGTTGTCACATGAGCATTCGAGGACGCGAGGCGAGTCAGCTAGTTACCCCAATACTGGAGGTTGAGAGGGCAGAAACCG

>TU6608-GaLu96scf_9-257579-258095 AAACGTCTTGTCGAGTGGTAACCACGTCGCAAACTCATCGGCCGACATACTGTTCGGTGTCGTGGTAGAATTGATCATGAGAACTTCGGTCGAACCAGGAAGCCTGGGAGCAACGAGGCTTATTGAACGCGCGGAGAAGCACTGGACGCTTGGTGAACTTTGGCGACACGAGCAGTGTCTTTATATGCTACCAATGTTGAAACAGGGTCCCGTCGGAACATGGAGTGGACCGCGATGTTGAGCGACGACAAGGCTGGGAGCAACAAACTCCATTCAGAGCGCAGTTGGGGAGGTGTGCTCAGGTGGATCTCAGTCAGACGTGGTTGAGGACATGACATCCGATCAGCACTACTACTCAGCTGAATCTCGTAGTTGTAGCCGCATATGGTCATGGGAAGGCTCATGCATCCCTTTAACGCTATCTCCAGGACCTCATACTGGTGATGATGTCGGTATCGCCTCGCTAGAAAGCTTCCGTAGACGTCGTCACACTTGAAGCTTGAAGCTGCCCTCGGAG

>TU6614-GaLu96scf_9-333319-333552 AGCATTATCCTCTTATGTAAGTCACAAAGTACTTAAGTCGGGCTTAAGCACTGTTAGATCTGGCTAATCCTGGCAAGTCAGCGCCGCTTGGCGCAACTGCCAATCATTGGTTTGCCGACTTTGTATCTCCCGAGCTGTGTTACGACTGAACGTCGAGTAAGGATCAGGGATATGCACGAGACGAGCAGGAGCGCCGACGAGCACTTGTGCAGCAAGAGCGGAGCAAAGCTCGAC

>TU6644-GaLu96scf_9-461000-461375 TCCCCACTGTGCGACCTCTGCTACCCTCTGCCTCCTCCCTCCCTTTTGTTCTCTCGGGCGCGCGCCTCGCGCGCAATACAGGTATGTGATGCGGACGCATGCTCTATGACGTCATCTAATGGTCGTCATCGTTAGGACCGCACGCGACGGGTGCGCGGAACTCAATCAGAGGAGCGCGCGGTGGGAAGGAGCGCGGCCGGATTGAGTGGGCGCCAGGGCAGAACGAGCGGGAAGGTGCGCACAACACTTTGGTGTGCGCGCAGCGGGTGGGTCGGGCGCGAGCCCGTACAGTCCCGCGCGTGAGTGCGTAGCATACGCCCGTCCTGTGTAACTATTTTTGGACCCCAATAGAAGTATTCAGTGTACCGAATAAACG

>TU6648-GaLu96scf_9-476894-477136 CCTGAGAGCTGCTTGTGCTTGTGCTTGTGCTTGTACGACCAGCGACTGGCCAGGTCTCGCTCTCCTGCGTTTCTCCTGCTCGTCCATCTCCCACAGCGTCGGGCACGGTTGTTGAAGCCGCTCGCAAGCAACGCTTGCCACCCTTCCAACCGTCTTCCCCGGTCTTCGTCCCCCCTCCCCCCTTGATTCGACACACTCCCGACTTTCCCTCGGGCCAGCACAGTTCCTTCCTGCTCTAAATAG

>TU6660-GaLu96scf_9-565663-566006 TGTAGACACGTGGTGCGTGGACTCACTGCCGCTCATGTACTTCATCTGTCCCGCGACACACACGGCCAGACTGCCTTCCGGCAGGCTCTCCATCAGCTCCCGCCTCCGTCTGTCATATTCCGCCTGCGGGATCCCCGGTGTCGTCTCTTCCGAACGAATCAAATGACCATGCGAGGAGGCCAGCGGTTGGCCATAGAGGGTGGGTTTCAGTTGCGAATGGCCGTTGACAGTCAGCGAGGAAGCGTAGCGGCGCGCGGGGGTGGGCAGAGCATGAATAGAGGTCAAGGCAGCTCTTGAGGAGGAGGCTGCCCGTGCTGCCGCTCTTCGACAAGATGGCAGAGAGC

>TU6707-GaLu96scf_9-1029608-1030322 TTCGCTATGCCACCTCCCGATGATACGCTATTCGCATTACCAGTCTCAAGATACTATGCGATCGATATTCCGGATAGAATCGCGTTCTGCTTCCTGGTCAGGTCAGGACAGGGTCGAGGGGGGACAATGACGAAGTAATCACCCAATTATGCTGCTACTGCTAGTCCGGTCGGGCCTTTTACTGACTTCTGGAAGTCGAATGTCCCAGCTGAGCGGGCATGTGTGCGCGGTGTGGCCTCCCTGAACGTCGAAACTGACGTCGCTTGGCGATATTCACGGGTCGGTCTGTTGCATCTTGGCGACTGTAACTGCATGTTGCAATGCCTGAGGACTGAGCGCTGAGCTATCGGCCGGGGGCAGATGATCGACAGAAATACAAATACAGCCGAATGTTAGAGTTGGTGGATGACGACAAAGAAGCAACAGAAGGCAGGCCCAAGGACAGGGCATGGGAACCTCATGGCAATCTCTAGGGCGGGCGCGCTGGAGCGGTGACTCGCACTCAATGGCACGATAAACCGCGGTGGCGGAAATAAGGTCGCGCAGCATACTCGAGGCGCGTCGCTGCACCTGGCGATATTCAGAGCGACGTAATAAGAGCCTTGGCCAGTTGCGCACTGGTTGCGCACGAGCCTGGACCGTCTATAGCACAATGACGTGGGGGACCTGGGTTGTGGGGGTGAACATGAATATTCATATTCATGCGTTCATGAAG

>TU673-GaLu96scf_10-127098-127399 GCTGCGACCCGTCAAGCCTCTGCCTCCCACCAAGTCCGCGATTGAGGACTTGACTTCGCGCGCAACCTGAAAAAGGTCCCCGGAGAACTTCCGATGACCTCGTCCTTGCTCCGGAGCAGGCGGACGAAGAGTTCGAGTTCGAGGAAATCGCATATGACGAACTACGTCTACCTCTTTGTCCTGCCGACCCTGCGCACTTCGCGCCTGTCTTCGACTACCAGAGGGCATACCATCCCTCTTTGCTCTCCCGTTCTCGCACATCCCTGTCTCGACTGAGGAAACTCTCCCAAACCTCTCTCAAG

>TU6750-GaLu96scf_9-1432835-1433342 TATTATAGATCCTCCATGCAACCTCCATGAACGGGCTTCACAGCGGGCGGGTTTAATGAGACACGGCTACTACTTAGGACGCCATCAATGCAGGCAGGCATCAGCAAGCACGGACATTGCGCAACCGTATTCCCGGACGAGGGGAAGAAGGGGGAAGATCCAGGGATCTAAGATGAACATGGTGTTACAATGGGTTGGGACAAGGTTGGGATCAGCGAATGTGGCAGGTGACGACAGAAAGCGGTGAGGATGGGTCGAGCGGTGGTAACGAATTCAAACTAGGTAAAAATGGGGCACGCAGACAAAGTCGGCGGTGCCTACGATGACTCCTCCGTAGCCGCTTGAACGTTACGGTGATGCTAAATGCATGGTCCGGAGAAGCACATGGGGGGCATGCTTGAGAAGGTTGGCATGGCATTTGCGAGTGATAGCCAAGTATGTACGAGGAGAAGTAGCCTGAGAGACGGAGGGTGAAGACCGCACATACAGCTTCAGACGAATAGTGGCT

>TU693-GaLu96scf_10-198294-198880 GCTGGCAACGGCGGCAATGGTCCCATTTTCCTCACTCTGGACATGCATGTGCACTGCTGCTGGCTGCATTCGTCTAATAAAATGCCCACCTCGGGTATTTTTCCTACCTTGCTTCAAGCTATCGCCTGAGCCTCTCCAAGTGTCGGCGGCCTGTTACGGATTCTGACTCCCGGCGGGAGTGTTCGCCTTCGACCACTCCTCTTCGCAATCCCCACGCCATATCGAACTTATACACCACTGTCGAGCACACAACACTCGATCATCTTTGCCTGAGGTACTCTTTCGAAGGGTCGAGTGAGATTCAAATGTCCGTACCGTTCCGAGTCCAAGTCACCCCGGGCCCCCACGTAGGAAACTCCGAGCCAAGAAGGTGAGTGCGTTCAATTGCCAAGTACTCATTGTCCTCTGCTCCACCGTTATACATGTGAGTCCCCATCCCATCACACTTAATGTCTGTCAGTCCTCATCTTCTTTGCGCCACTATTGCAGATAACGCGTCCCACGATGAACTTCGGCTAGAAGCGGATGTCTCCAGACAAGTCCCCCACTCTCTGTCCCGAGCACTCCTTTGGAGACTTTTTAAAGGT

>TU694-GaLu96scf_10-199075-199521 ACATGGGCTGACGCTAACTCGCTACTTTCTGTACACTCGCAGGGTATGTTATCGGAAACTTATCGAAATGCCAGACGTCACTTTCTCACAAGACACCTCTACAATGTGGGTGGCGCCAGTCAGCTACATCCTGTATGTGCCGATTTTGCCTCCTGCGACGCTTCCTCTCACCGTTCGTGTCTGTCCGGTGAGGATCAGGGCGGAGAAGAGTCGACATTTGAAGAACACGCACTGGGGCAATAGGTGTCATGTGAATGTCAATCAGGATGGGAGCAGAGGTGTCCGCTGACCGTGTTGAGAAGATTTTGGGATGAGAAGACAAGCACCGAATGCTTCTGATCATACAACTGTGCTTGATTGTTCCTGGAGAAGGGGTTGAAGGCATCCAAGGTGCGTGTCGCCGCTTCGCTAGATGCAGTAGGCCCAGTGCACCTGAATATTGCCACT

>TU6-GaLu96scf_1-16623-16895 TCGAGCTCGTCCCGCCTCTTCCTTCTTACCACAACAATTCCCATCCGGATCCCCCATCTGCGGATCCCACTTTTCGCTCTGTATCGTATTCAACAGCATCGAGCTCGACTTCAAGTTCATACAAGTCTCATGATCGAGTAACCCAGAGGTAGATTGCAATTCATATGACGTCCACATGTTCCGCCTCGTCCTTGCGCCGGTCTCATCGATAGCAGGTCCTCATCGTTCTTCTCCATCACAGTAGTTGATTGGCCGGACTGGGACCTTCGAGCT

>TU718-GaLu96scf_10-356045-357414 CGGCTGTGATACGCACTCGAGCTCGAGACCGGGTTATATCCACGCATACACATACAGTACTTGCACGAACGCATATGCCGGCAGGGCTGCACAGCACGCCCCCTCGTTGCGCCGTGAGCGCAACATGAATGTGTCTCGCTTATGGTCCACGGCGGGCCCGGGGAGGAATAGGACTAGTACTGGGAATCACGACGGCGCGGGTGGCAGGCGACGTTAACCATACTGTAAGTGAATGGGGCTCTGGACGTGGATAATACAGCGCGCCGAAAATTTCGCTCCGCAGCGAGCTCCCGTCCGCTAAGTTAAGTAAGTAGCGTGGTACTCGGTGGCTTCGAATTCAGGCTCGCTCGCTGATGAATGACGTGGTTTGCAGGATCGGGTTGCAGTACGACGTGATTTGAAACTGGAGGGCCGGGCTGCGTTGGGATGGTGCTGCGCGTAAGTAGACGCGTCTGCACTTGGCTCTGCCTCGCGGAGAGGTCGCGGTCGCTCAGCGCTCAGGCCTCAGGGACGAAGAGGACGATATCGAGCTCGAGACGGATCGTCGGCGGCGGATGGTGTGCCGCCGCCGCTGCTGCTGCTGCTTGTCGATAAGGATCGTCGTCCCGCTGTACGCGCGGATGAACTCAAAACTCGTCGGCCGGACGTGATCTCGCATCTTGCGGATCCAAGGATTTGCGCTCCTGCCGCGCAATCAAGCTCGGGAGGTGTGTGCCATTGGAGCTGGATTATCGATATCACTTGGCTTCGTTCAGGTGTACGGCACAATCTCAGCAAGTCGGCATTCGGACACGTTCTGTCTGGTGGACCGCCTCCGGCTTCGTCGGCTCTTCACACTCCGCTGATCGCTTTACCTTTTCCGTGACACCGGAGAAAGGGAGGGACTGGCGTGGGAGTCTACGCTTGCACTGATTGTATTCTTGATCGACACTCAGCGCGACGTGCCAGCTGGTTAACCATACTCTTGTTGCACCTCCTCCTCCTCCCCCCCGCCGACGTCCCCTCGTAGGCTTGACCAGGCGCTGTGCTGGACCACATCTGGCGCAGGATAAGTCGAACCGTGGCCGGGTGCCGTGCTTTGCGGTCGGACATCGCGAGAAGTGTGTGATCTGGCAGGAAAGTTCTGGGCTGCGATGGCGTCTGCGTGAATGAATGAATGCTGGTTTGATTCCCGGGTTCTCGCAGTCTTGACTCTTATTAGGGGGCTTTCTGACTGCATGTCAAACGAATCCTTACGGGATCTGGGCGAGTAATCTTATGTCTATGTGGTCTCCGTACGACATCCGGTGCCACCATCCGAGTTTCCTTGACGGGACATCGATGGCACGGAAGATTCGAAGATTGGACGACAGGTATCAGTGACCGTAGGC

>TU735-GaLu96scf_10-556835-557435 GAGTCCCAATATAGCCGCGCGCAGCATGGACTTGACCCCAACACGACTTCAACCCCATCCACCATCCACCATCTCTCCTCCCCGCCCTGGCTTCAGTCATCACTTCCCGTCTCTCTGGTCCTTGAGCTCACTGGAATCTGAGACTTGCCTCAGTTACAACCTTATACGGAGCACGAGTGTCGCCTCGGCTGTCCCGCTCTAGTAGATGAATATCAGGTGCGTATATTCTCGCCATCTACCCGACAGTTATGCGTGCGGCTAGTAGGCCTGTTGGGCTGGAACCCGGAGTGTCTTTTGAAGGTACGTGCCCTCTGCGATGCCCCTGCTTTCAGGTATGTGCGCAATTTGTGTCCCCATTCAGATGTCAGTGCGTCGTCGGTCGTGAGTCTCCAGTCCAGGTCCAGCTTGAAGATACGTGCTTACCCTTCATTCTCGGGGCTTCCGAGTAAGTCAGCTCGCTAGTGCGCCTATCGCCAGGCGCAACACGTAGGTCCATCCTCCAGAATCCCCCGGGCCCCAAACCCTCCTTCCCCCGACAGACCGGCCTTCCACCCGTCGAATCATTACACATTAGGTGAGTAGTCTGTTCATTGCATCGCGT

>TU76-GaLu96scf_1-432071-432734 GTACCATGCTCTCTTCCCCGTACCATTGACATCCCAAATCATCTCTCTGACAGAATCTGAAAGCGTTCTAAACTGATTTGGCACAAATTTCTCGGTGCAAAAAACGGATGGGGACGAGAGTGCGTACCGATTACCATGTTAACGTTATTGACCGCGCGTCTCGCCTGATGTCTAGGTAGCAAATGCGATATCGCACCGTGGCGGCAGTCTCAGAACAGGTGGTTGATGCCTGTCAACAGATATCGAGATCGAGCGTGTGGCCTCCGTTATGTCATGGGCTTCTTGCAATCGGAAACAACAGAGGCCAAAGTTGCTAGGACTCCGAGGAGAAGCCCGGATGAGGGTAAGCATTTGACTGTTTGTGTTTTCCGGTGCCAAACATCGTTACTTTTCGTATTGCTGATTTGCGATGTTATAGGACTCACAACGTAACATTGAACGATTTGGAACGGTGACTTGATTCCCCACACGGTCCTCGATGGATTCTAGAGAGGATCAGCGCTCGAGGAGAGTAAGTAGACCGCGGTGGTGCTGCGGCTTGAATCAATGTGGGTCGCTGTGAATCAGCCTCATAATGAGTTTACCTTCGCTTTAAAATAGCCACCGATAGATAAACTGAAGACTGCGGGGAAAAGTGGCCCAGGTTGTGCCAGTGGTGGATTAT

>TU773-GaLu96scf_10-953686-954365 CTGACCTTGGACATACGTCTTGTGGCATGTGTGAACATGGACACGCGGCCCACGATAGCGAGAGTGCACCATGTGGTGCACACCTCGCACCCTCGACACCTGACAGCACCCAGTTACGTTTCATGGTTCCAATCGTCCTCGAGGCTCGGTCCATCCTTCGCCGTCAGCACCCAGGCTCCGGCTTTCCTGCAAGCTAGGTGAAGGCTCCCACTCCAGGAGTATCTCCTGAATAATGTTTCTTACATCCTGCGAAGATATTTTAGTTCAAGGCTGTGTCATATTTTATACCACATCGCAACCCTTCCGAGCGGTCAAGGGAGAGAGGTAGGCCAGTGTTTGGTTCACATTGATCGAATGAAAATTCCTATCTTTCCGGATGAGAGAGTTAATGTCCCAGCTCTAATGCGACCCCGACTGCTAGGGACCACAGGTCGTTCCGCCCACGGTCGACGGGTTCCTTACGCCCTCCTAATTAATTCAGCATCTGTGCCTCACGGTATGTAGTCACCTGACGCCAAGTATAGCTCTATGGCATGCAACTCGTCAGTAAATTAGACTTTACAGGAGAAGCACCGTGCTTCGGATGTCTTCCGTCTTCCGAACCTGGCTCGGTGTGAGTCTCGGGGTCTGTTCTATGATTCGAGTCGGTGCCCCTCAATATATAGTGTGCGCATGCTGAC

>TU781-GaLu96scf_10-1241744-1242998 GCAATGATTCAACATCCCCGCCATCGACGACGGCGTTCAACAGCCCGGCCTCGGGCCCCGGTGGGGATTCCATGCTGTCACTTTGAAACCTTAGCTCATATTGTTTCTGTGTTCCGCTCTAGACTCAGTCGCGACACGATTGCACATGTCTGACGACCTCCTCATCCCTTCCGGGCGACTACCGTATCTTTATCCATGCCATGTCGCTTTGCCTTTGCACGTGAGTACACATTCAGAGTGTACATGGGAGACAGGAACGACATATTACCAAGCATAAGGCGCAGGGGGAACTGGACTCTGAATCTTGGCACCGCGAGGACTGTGCATCGAGTGACCATTCCCGCCTCGGCGATTGGTTCCCGGCAGGCAATTCCTCGATAGCCTCCTTTGGTGAGGGGGTGTGGTTCTCTTGAAGTGCCAAGCTTATAGATTTTGTCCAGGAACGGAAAGAAACCTGGCTCCACTGCTTACTGCCTCTCGTGCGTCTCTCTCGCATCTACCTGGAACCAGAGATTCGAGGATATCCGCCTGCTCTCTCAAGCCTCGAGATCTATTTTCTCAAGTTCCTCTGAAGAATTTCCCCACAGGGCCGATGCCCATTACACGCAACGTCGGTACCTGCATGGCTTTGATCGCCACAAGACTCATCACCTCCGTATTGCGGCGCGCAACATTCGATCCTGTCGTCTTCCATGTCAGATCCTTACCCCACGACGCCGTGATTTTTGCGCCGTCCGAGTCGCCGGAAGCGACGAGATGTCACGGCGCCTGATTCCCCGGTGCTGTCCACAGGTGGTGACCCGGGTTTGCGAACGGCGCAGAGCTCCTCCGGTGCGGTGCGCTCGCCTGCGATGGAAACACTACAGCCATTTATGAATCGTATATCGGAACGTTCGGGTCGGCATTGTCGCTGGGATGTCGATCTCCCGGATCGCGAGCAAGGTGGAGGGGGTGGCGCCGCTTGCCCGCTGCAGGTTTCCGCACTATGGCGAACGCCATACATAGCATCATTAAGAGTGCCGATGGCGCCGTAACTGTGGCCCTAGGTGCGTGTCATACTGCTTTGTTTACCCGTCCGGCGTGCGACGACGCTGAGCTAGTTGGATGTGTGTCCATCAGGCCTGTCTTATACCACCATGTAGTGCGCGTCGGCGTACGAGGCCGGCGATGAGAAGTGGGTGGTACATGACGTCTCCGGTTCGGCTGTTTCTTGTGCAGAGGTACGTGTACAATGATACCGCCTCTCCAGGACGAC

>TU838-GaLu96scf_11-71512-71855 GATAGGTGTACGCTACAGTGCGCTACCTGCGGGCTCGTCAACAGCTCGCGCGCGGGACCCCACGGGCTCGCGCTACCCTGCGCGTGCACCCATATGTTGCGCGCGCCTTCCCGCCCGTCCTGCCATTCAATGCGTGCCCTCCTTCCCACCGCGCGCTCCTCTGATCGAGCTCCGCGCACCCGTCGCGTGCAGTCCTAGCGATGGCAACCATTAACATTAAATGACGTCATAGGGCTTGCATCCACATCTTAACATACCTGTATCAGCGATAGCGCGCGAGGCGCGCAACGGAGAAAACAAAAGGGAGGGAGGAGGCACTGGCAGAGGGTTGCACAGTGGGGAGA

>TU842-GaLu96scf_11-83674-84481 ACTACCACGACGTCTCTCCCCTATCAGGCCATCTTTGACTCGATAACAACTTCCTTTTAATCTGTTATATACTCCTGCGCGCACCTGTGTAATCAGCGACATCAAGTTCGTCAAGTCTCAAAGCCGCCACTCTGCAGTAGGATTACGTTTACAGCCAACCGCCGACATGGCAGGTTTACAAAGCCCAGGTGCGGCTTTGAATTGTACCATCTGAACCAGTAAATCTGACATGGGTTCTCACGGGATTCTGACCTCGTCATGCGCATACAGCCGCCAACGTCAAGCCAATTTTCCTTTCTCCCACCCATAATCCCGCATCATGCACCGCGTCCCCTTCGAAGGGCTCGAAGGACGATAGCGCGCAACAAATTGGTGAGCATTTTCAGATCCACTCATGTGAAGGTTTTTGGTCAACATCGCCCGTCACAGAACGTCCTGACTTCCTCGCAAAAATGGTCAGTAGCTGACTATATGCGCTGCATTCTGACCCTATAGTCTGTAGGCTGCCGCTGCCGCCTTGATTCTTCTAAGGAACAGGCAGCGCGAACGGTTTTCTGCGGCGGGACCTACAACGCAAAGTGATCTCTGTGGGCTTCAGCCTCTCTCGAAGTTCTCCGGACCGCGTATTGCTCCTGGCGGCTTGTCCCACTCTTGCACTGGGTCGACTTCTCAGAGGTATCCAGCACCTTCCGCTCCCTGTCAGCCTCTGGAGTCAGTTTCAGAGGAAAGCGCTTCACGGCTTTCCGAGGACGAGTTGCTGATGGTGCTTACGGAACCGTACCTTATGGATTGGTGTTTCCGATAACTG

>TU84-GaLu96scf_1-524998-525540 ACATACGACAAAAATTTGGCCTCAAGCTGGAGCCGTTCCAGGAACGCGTGCGCGCTCTCTTGTGTTGCTGAACGTCCCATTTTTCATCAATTGCGACTGGCGAGTCCTTGTCAAAATTCAATGAGCTAGTGTTCAAGAGTTTTACGGTTGTTAGCAGGGCGCGATGTGACTACAGGTTATAGCAGAGAGAGTCGAGATGATAACAGTACCTGAATGCGCTGGCAGGCAGCATGAGGAGAAAGTCATACGAGGCTTGATGAGGCGTCGCATTTCCAACACGCTGTTGATTAGCGGTTCGAGACTCGTGACGAATTCGTGCAACATGGATTGGAAAACGTCTGACAGAACAATCATACAATACAAGGTATGTAATGGCGGCGGGGACCGTCGAAAACGTGTACCCCGCTAGTAGTCTTCTTCGCCGGGTAGTCAAGACCCGAGGAACAGAGTAAACGAGGAGGATGCTCTTCGTCCCCGCGAGCGCCTGCAGGTTCCCTTGAGACAAAGAATTGGGAAGCAAGTGACTCTAAGGCAGAGACGGTG

>TU866-GaLu96scf_11-211813-212104 GTGAAAGTCGCATCCCGTAGTCGTTCGAGACACTTCGTTCTCCACGCGTCCGGATCCCGGTTATCTTTATCTTTGGCGGAGGGTGGGAGAGGGAAGGTGGACACCACCATTGACGGCCAAGTCGTCGACTCAGAGGGAGGACGAAGTGTATCCCTTACCGGTCCACGATGGACAGGCCAAGACAAGACATGTCTGCAAGGCCGAGCGAAGGGTCGGTGGACGCCGCGCCACTGGGGGGCGGCCTACAAAGGGCCTCGCACACCTGCAGCATCACCAGCTCGTGGCCGCTCGC

>TU868-GaLu96scf_11-213487-213984 TGTCCAGTGGTTGTCAAGATATGCATACGCCTTCCTCGAGGGAGGGTGGTATACATATCTCAGCAACCACTGCATGCATTGTCTTCGTTTCTTCACTCACTTATAGCTACTTACTAGCGTATATAGGGTATACCATTCGTTTTACAGCTCTCCTGCATGCCATAGAAGGAGATGGCTATCAGTACTCGATATGGGGCACCTTCGACGCCCCATATCTCGAGCGTTTCTCCACGCCCCACGGCGCAACACACATCCACGGAAAGCCCTAGGCCAGGGCTTTCCAACGAGCCATCATGCGCCCGGATCCGACCGCTGGAACGTCCGGGGTAGGGACCCCTTCCTCCTACCCCCATGTACCATTAATATGTCTATAGTGGCTTCAATTCCCAACGAAATGTCTTCGTTCCTTTTGTATTCTCTTCTGATTATCTCTGTATAGCTGTTAAATGAGACGATTCGCTTATCGCACACCAGGATCTTAGTGCAGTTGCTTATCAG

>TU869-GaLu96scf_11-214300-214875 CAAGGCGTCAGATGTGCATACATTAATCTAAGGATATATGGGAACATGGGAGAGCATTAGACACCTTTTGTACCAGACTGATAAGAGCATAATGCCAGAGTACAGGCTCTGATAACGATGATATTACAGAGCAGCATATCAGTATACAACCAGAATACAAAACAAAAGGAATGATGAGAATAAGTGCTGTATTAGTTTAGATATAAGGTATACAAGCAAAATGAGAGGCAGGAGGAAGGGGTCCCTACCCCGGACGTTCCAGCGGTCGGATCCGGGCGCATGATGGCTCGTTGGAAAGCCCTGGCCTAGGGCTTTCCGTGGATGTGTGTTGCGCCGTGGGGCGTGGAGAAACGCTCGAGATATGGGGCGTCGAAGGTGCCCCATATCGAGTACTGATAGCCATCTCCTTCTATGGCATGCAGGAGAGCTGTAAAACGAATGGTATACCCTATATACGCTAGTAAGTAGCTATAAGTGAGTGAAGAAACGAAGACAATGCATGCAGTGGTTGCTGAGATATGTATACCACCCTCCCTCGAGGAAGGCGTATGCATATCTTGACAACCACTGGACAGA

>TU87-GaLu96scf_1-534476-534720 CACCTAGGCTGTCGATTGTGCATTGGTCCATCCCGAGTACGTAGCGCATATAGCAACGATTACGTGCACCTCTGACATGCATGTGGCTTGACAGCTTCGGACGTGGACCACAAAGCTCTTTGAAGGTCTGCGGCGCCTAATGACTCCGACTACCACTGGCACTGGCATCATGGCACAATCCGAGTCCAAGTCCGGATCCATCATAACGGAGCTCCAGGAGACATTGAGAAATGAAGTGGACAATA

>TU912-GaLu96scf_11-457098-457475 TGCCATGGGCCTTCAGGAACACGATGCCAGTGAGTGTCCGATGCCATTGTAGTCCATCCTACGCCAACCCTCCCTTCCTCCCGCGTCGTTGGCAGCAAGCTTTATCACGCATGAACCGTCATCTGCGTCGCACATGCCGCTCCCTAGCCGCACATCAGCCTGCATGGTGGCCCAAAGCATCGTACCAAAAGTCCTTCGGTGCCGTCCGGATCCGTGGCCAAAAACTGCAGAACAAGTTAGTACGTCGCCCGCGCGTCATGGGCAATGTTGAAGTCGGCGACTAGAATGAATGGAGAATATTGAGGGGAGGTAGGGCGGTCCTAGCAGAGGGCAACCCCTCGGTGGATAGGACGAAAGTTGGTCGATGGAGTCCAACGG

>TU966-GaLu96scf_11-886406-888371 CGTGCGTCCGTCTCCGATGCATCCGAATCTCTCTCTTTCAAAAACACACGAATGTAGCTCGGAATCTTAGCAGACTCTTCCGCGGCCATTTCTGCATCAAGTTCGTCACGCCCACGCCGCCGGCAACTCGCCAGATGGACTGGAATGTCTTTACTCGTTGAACTCTCGGGCACCACCCGTCGGTGAGTGTATGTCTACTATCTACCGCGGCTGTATTCAATGGAACACTCTGAACGTGCTGTATTACAGAGACAGGGTTTTACGACTACGACACGACCCCAGCCCGCTATTCTGGTCGCTCTGCCATCACGCAGGGCCGCGCTCAGCAAATCTTCTCCAGAGGGTCCTCCTTCTTCCGTCGGATCTGCGACATCCGACATATTTGGGGTCGACGCAATGTGAAGTATGATCTAGTCTTAACCATGCCTCATTTCACGGCGATGGTTGGTCGCTGGCAGGGTCAACAGAGATAGCGGACGGGTCTTCAACTTACAGCACCGCTCCGCGCCACGAGTCAGTACAGTCAGCCCTGCCGCAGTGCTACGGTTATACACTATGCAGACGCCTCCCAGCATCCGCTCGAACCCTCCGAGTGCCACCCGGTATTCAAATTTTCATAGTTTTCGGACACCACGAGGCGTACGGCACTGACGACCCGGAATCAGGTCCCCTGACTGAACGAGGACTGCTGCGTCCTCCCGCCGGGCGTGCGGAAGCATCATGCATCACATACCGTGCTTCGTCGCTAGCTTCGCACATCGGAGCTCGTTCGAGTTCGAGGTACTTGGCGTTCGTAGCCGGGGAGTAGGCGGCGCACATGCGAACTTGAATCTTAGCTGACGTCCCTTCCAGTTTTGCGGCCCTCGATCGCTCACGTTCAGGGGGCGCGGTGGAGAACAGACTCGGGACGGTTGGTTGTGCCTCCGAGTAAGCAGGCGGCTCGGAACGTGCCTCGCGCCACTGCCTGTCGGCTGGCCTCCGGAGGCACGCCGCGATGGACGGTCGACGGTCGGGGTCACGGTCATGCGCGCATGTGATCGCTTATGATTCTGTCTCTGTCTCCCTGCAGTTCGGGTCCCTGAGATCTGGGGCCGCTCGCCTTATCTCTGTCTCCGGGAGATAGCGCAAGGGAGAACTTAAGTAGAAGCAGGCGGCTTGCCTTTGGGGTCCAGCCCGACAGGCTGACTAGTGGTGACCGTCGGCGAACTAGTGCGGAGGGCCCCGCGGACCGGGACGGCGAGCTTGTTCGGGAAGTCAGCCATCCGACTTGCGAGCCTCCTCAGTCCGAGATCCTGTCACACAATGGAGCGAGGGGGACGACTCCGACTCCGACTCCGATCAGCCACGTTCAGAGGTTGCGTCCGCACCCCGAGGACGAGGACTAAGCCAGCTTCAAGGCGTCGCGCATATATTGAACTTTGACACACAGCAAGGCCTGGATGTGGTGAGTCGAATGGCTTGTCTACTCGGGGTGAATTGGTTGACTAGACGGCGAGCAGCGATGTATCGGCGGACACTAGTCGTCCGCCGCCCTCTGCTGGCACAGCGACATGGTTTTTTGAGATCTCCGGAAGGATATCTATTCAAATCGTCCGGTGGATGTGAGTGAAGATCAACCTGTCTCGCATTCGCCGGACCCTTCGGCTTCTACTCCATTCAAGTGCGCAGGGTTGAATCGGCCTCTCAATTCTTGCGGGCCACCGTACGAGCAGAAGGTGGTCTGGTTGCGTGACACTGGACTCGAGGCCGGCTTCGAAGCAACTCCGGCTCTCTCATAACCGCGAGAACGGGGTATGTGGTACTTGCCCGAAACTCGCTATTCCTCGACCAAGATTATCTAATTGCTGATGTTTCCCCACTGCAGAGATAACACGCCGCTCTCCGAGGCGGATGGACGAATTGCGAGTGGCCGGACCTGTGAGGCATCAGTGATCCCAGGCCATCGATCCCGGAGCCATGTTTGGAA

>TU969-GaLu96scf_11-896780-897543 TTGGTGTCGAGTGGTAATGATCTTGCATTTCCGCCAACCACTTGGCGGGTCTCAGCACTTCACGCCCACATTCGAGTGACAAGACGACGATGAATCCACCACGCTTGCTCAATCTCGGTAGGTTCCCCCCAAGCCGCATTTATAATGTTTAGATTTCTCATATAGTATCTATCAGAACAGCGACTAGGGGCGGATTTCCATCTGGAATCGTTCTATCCAGCTTCCACGAACATTCGGATGGATGTCTGAAAAATCCTGTGAGTCCCCAACGGCATGCTTCTGGTAAGGAGGTCCGGGTTGGCTCTCAGCTAGGCGTTCGATCCGGCCAGTGTGGTGAATTTTCGTCGGCCGACGACACATTACGTCTGGTTTGTCTATGGTGTCGATGGCGTTATGAAGCCCATAAAGCCATCGATAACGTGGCACTCGGGCGGTACGCTCATCCACCCACAGTCGAATTCCGATGGCTTAGGGCCGTAGGAGGGCCTAAATTGCAAAGTTCAAGCGGTTAAACCCTTCTTTCCTGACACGGAGACGGGAGTAGTCAGTCCCAGTTCTGGCCGCAGCATCGTCATAGAAGCGTTCTCGCCAAGTGAGGGTGTCGACGTTGTGACGGGGACTCGTTATTTCCCTTATTTTGTAACTCAGACCGTTCTTGGCATCGATCGACAAAGCTCGTTCGCAGAAAGTCATCCGCGGACGACCTTCACCGCTGTCGGCCGGTTTCTTGCACTCATGGCTAGGTTGCTACGAGACCAAGTATG

>TU973-GaLu96scf_11-912660-913209 TTGTCCGTCAGAACTGAACCATAACGTTCTCACCCTAGTCAAACCCTAGTACCAACTTTGCTTGATGGAAAAAGTCTACTATGTACTAGCATCAGTAGTGCATCCGCGATCCGACACAGAGCTCGGGTATGTGAGGACTTCACACCCTCCCTCGTCTAGCCGCACCTGCGCAACAGCTGGACACACTGGTCAATTGCCGCAGTCGCAGATAGCCCTGGAAAGGGTTTGGAACGAGTCGTTACGACATGAAATTTCCAACCTCCGTAGTCCTGCTGTCAGCCAATCTCCGTTCCATCGGGGGCGAAGAAACTTAACTGTGTAGCGTTGGCAACGGTGAATCCGCTCGCGGACGAGGTCTAATGAGTTCCTATGGATTGGTCGCATGGCTGGTAACAAGTGGTCAATGGGACAGGCGCTGCTCATCCTTTAAACAAAACAAACGCACCGTGCGGCGTGGACCCAGCTGCAGGGTAAGAGCACCGGGTCAAGTATCCTCGGTGTGGCACGGCTATTCGGTCTATCTTGCGTCAAAGCCTTCGGCAAGGATCGC
